# Transient Au–Cl adlayers modulate the surface chemistry of gold nanoparticles during redox reactions

**DOI:** 10.1038/s41557-025-01989-4

**Published:** 2025-11-13

**Authors:** Sarah May Sibug-Torres, Marika Niihori, Elle Wyatt, Rakesh Arul, Nicolas Spiesshofer, Tabitha Jones, Duncan Graham, Bart de Nijs, Oren A. Scherman, Reshma R. Rao, Mary P. Ryan, Alexander Squires, Christopher N. Savory, David O. Scanlon, Abdalghani Daaoub, Sara Sangtarash, Hatef Sadeghi, Jeremy J. Baumberg

**Affiliations:** 1 NanoPhotonics Centre, Cavendish Laboratory, Department of Physics, University of Cambridge, Cambridge, UK; 2 Centre for Nanometrology, Department of Pure and Applied Chemistry, Technology and Innovation Centre, University of Strathclyde, Glasgow, UK; 3 Melville Laboratory for Polymer Synthesis, Department of Chemistry, University of Cambridge, Cambridge, UK; 4 Department of Materials, Imperial College London, London, UK; 5 School of Chemistry, University of Birmingham, Birmingham, UK; 6 Quantum Device Modelling Group, School of Engineering, University of Warwick, Coventry, UK

**Keywords:** Organic-inorganic nanostructures, SERS, Surface assembly

## Abstract

Controlling surface chemistry at the nanoscale is essential for stabilizing structure and tuning function in plasmonic, catalytic and sensing systems, where even trace ligands or ions can reshape surface charge and reactivity. However, probing such dynamic interfaces under operando conditions remains challenging, limiting efforts to engineer nanomaterials with precision. Here, using in situ surface-enhanced Raman spectroscopy, we identify a transient Au–Cl adlayer that forms during electrochemical cycling at gold interfaces. The adlayer exhibits significant charge transfer between gold and chlorine, generating an outward-facing dipole that polarizes neighbouring atoms and modulates the local potential. This dipole stabilizes nanogap interfaces and directs oriented ligand rebinding, enabling reversible reconstruction of subnanometre architectures. It also alters interfacial charge distributions and mediates electron transfer between gold oxidation states, acting as a redox-active intermediate. These findings show how transient surface species shape nanoscale reactivity and stability, offering strategies for designing catalysts, sensors and nanomaterials.

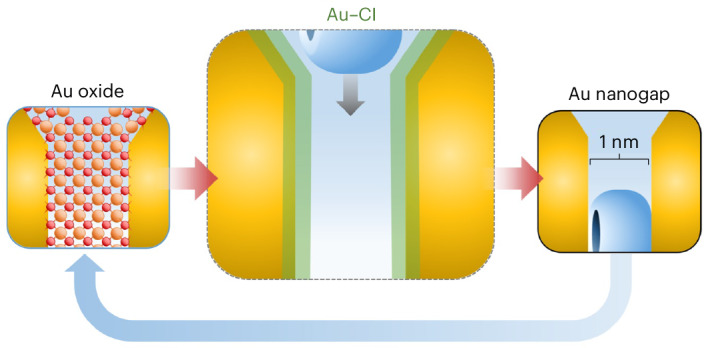

## Abstract (web summary)

Controlling nanoscale interfaces is key for ensuring stable plasmonic and catalytic function yet remains difficult to achieve under operando conditions. Now it has been shown that transient Au–Cl adlayers function as redox-active Au(I) intermediates, modulating interfacial electrostatics. This modulation stabilizes gold nanogaps and directs ligand rebinding, thereby enabling reproducible regeneration of subnanometre architectures.

## Main

Gold nanoparticles (AuNPs) are essential in modern healthcare diagnostics, serving as substrates in sensing platforms such as lateral flow assays, where their nanoscale interfaces enable selective biomolecular recognition and colorimetric detection^1,2^. Their functionality in these assays depends on local surface chemistry, which dictates ligand attachment, aggregation and overall performance^3^. This dependence on surface interactions underscores the broader importance of understanding nanoscale interfaces, which govern critical processes such as electron transfer^4^, molecular adsorption^5^ and colloidal stability^6^. Even subtle changes in the interfacial environment can dramatically reshape morphology^7–10^, alter molecular binding^10,11^ and reactivity^10,12,13^, and ultimately determine material functionality. Thus, understanding how ligands and ions modulate nanoscale interfaces is essential for designing better sensors, catalysts and nanomaterials.

However, probing nanoscale interfaces under operando conditions remains challenging. Techniques such as X-ray photoelectron spectroscopy (XPS)^14,15^ and X-ray absorption spectroscopies^16,17^ provide valuable insights into chemical states and coordination environments but often lack the resolution needed to resolve transient intermediates at the nanoscale. NMR^18–21^ and mass spectrometry^22^ offer molecular-level insights but primarily detect reaction products or bulk-phase intermediates. Surface-enhanced Raman spectroscopy (SERS), on the other hand, offers high sensitivity and molecular specificity by probing molecules within electromagnetic hotspots at nanostructured metal interfaces^23^. This surface sensitivity makes it well suited for real-time monitoring of interfacial processes, especially when coupled with electrochemical techniques^24,25^. However, conventional SERS substrates prepared by electrochemical roughening exhibit ill-defined morphologies that suffer from instability and poor reproducibility, making it challenging to systematically probe dynamic interfacial transformations^26,27^.

To overcome these limitations, we have developed a multilayered AuNP aggregate (MLagg) platform stabilized by cucurbit[*n*]uril (CB[*n*], *n* = 5–8) scaffolds, which define sub-1-nm gaps between gold facets^28,29^. The CB[*n*] scaffold not only establishes uniform gap spacing, which is key for achieving reproducible strong SERS enhancements^30,31^, but also provides the structural stability needed for systematic studies of interfacial dynamics^32,33^. Building on this platform, we introduced an electrochemical regeneration protocol (EC-ReSERS) that oxidatively removes adsorbates and restores the CB[*n*]-defined nanogap structure in situ, enhancing reproducibility and extending substrate lifetime^32^. During EC-ReSERS, the oxidative step also oxidizes the gold surface, disrupting the nanogap architecture, while a subsequent reduction step ‘rescaffolds’ the interface by precisely reconstructing the nanogap through CB[*n*] rebinding. This reversible transformation between an oxidized and reconstructed nanogap makes EC-ReSERS a compelling model system for probing dynamic nanoscale surface chemistry.

In exploring the dynamic transformations underlying EC-ReSERS, we now identify a transient Au–Cl adlayer that plays a crucial role in this regeneration process. While chloride ions are often regarded as background electrolytes, our findings demonstrate that even at submillimolar levels, they play a crucial role in stabilizing nanogaps and modulating the electronic structure of the gold surface. This highlights how seemingly innocuous species can profoundly influence nanoscale interfaces, impacting both surface properties and reactivity. Here, we systematically investigate the formation and role of the Au–Cl adlayer in EC-ReSERS and extend these insights to chemically driven regeneration (Ch-ReSERS)^28^. Understanding these transient phenomena offers broader insights into nanoscale interface engineering, with implications for plasmonic sensing, electrocatalysis and the controlled design of nanomaterials.

## Results and discussion

### Electrochemical rescaffolding (EC-ReSERS)

**Fig. 1:**
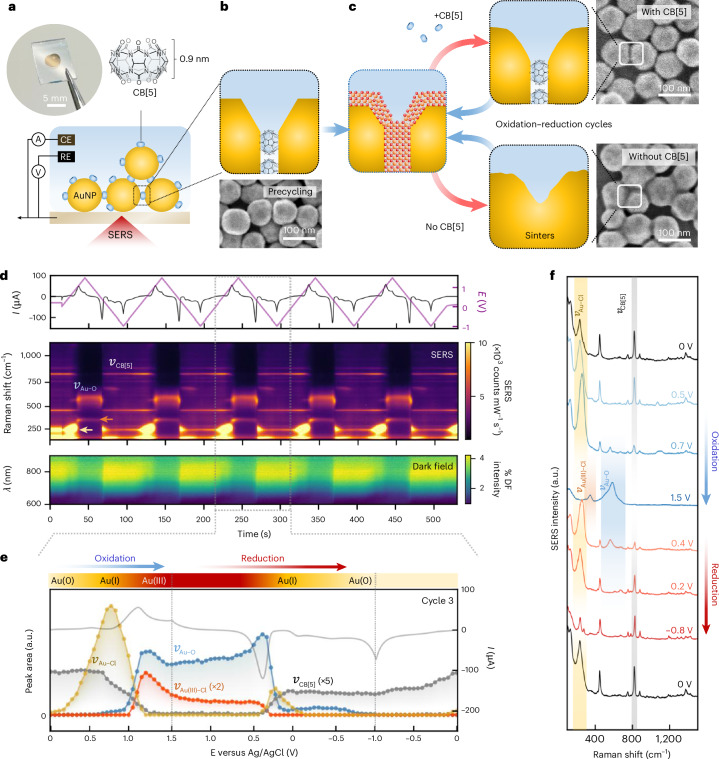
Evolution of gold facets under repeated electrochemical ORCs. **a**, AuNPs aggregated with CB[5], deposited on FTO-coated glass, and assembled into a spectroelectrochemical cell. CE, counter-electrode; RE, reference electrode. **b**, SEM of MLagg-CB[5] with schematic cross-section of a nanogap scaffolded by CB[5]. **c**, Schematic of ORCs, showing MLagg-CB[5] oxidized (blue arrows) to form gold oxide and reduced (red arrows) with or without CB[5]. SEM and cross-section schematics show morphologies after multiple ORCs with (top) and without (bottom) CB[5]. **d**, Applied potential (versus Ag/AgCl, purple) and current (black) versus time, together with time-series SERS and DF scattering spectra during 5 ORCs (50 mV s^−1^, +1.5 V to −1.0 V) in 0.1 mM CB[5], 0.5 mM Cl^−^, 50 mM potassium phosphate buffer (pH 7.0). SERS features of CB[5] and Au–O are labelled; yellow and orange arrows mark Au–Cl and Au(III)–Cl modes. SERS and DF spectra were measured on separate samples subjected to the same treatment. cts, counts. **e**, Extracted peaks for cycle 3 of **d**: Au–Cl (240–265 cm^−1^, yellow), Au(III)–Cl (345 cm^−1^, orange), Au–O (520, 590, 660 cm^−1^, blue) and CB[5] (826 cm^−1^, grey), plotted with current (grey line). **f**, Representative SERS spectra during 1 ORC: blue spectra from the oxidative sweep, red spectra from the reductive sweep, and black spectra at 0 V showing initial and final states. Key vibrational features are annotated to highlight gold surface transformations.

Unlike electrochemically roughened electrodes with an ill-defined nanoscale geometry^27^, ligand-stabilized nanogaps survive multiple oxidation–reduction cycles (ORCs). Here, 0.9 ± 0.05-nm-wide nanogaps are defined^30^ by aggregating 80-nm-diameter AuNPs (Supplementary Note 1) using rigid scaffolding molecules of CB[*n*]^29^. CB[*n*] binds AuNPs through its *n* = 5–8 carbonyl groups (Fig. 1a,b). We use 1–2 monolayers of these aggregated AuNPs (MLagg)^28^ assembled in a spectroelectrochemical cell^32^ (Fig. 1a and Supplementary Fig. 1), which provides direct control of the electrochemical potential while enabling real-time monitoring of the nanogap surface chemistry.

**Extended Data Fig. 1:**
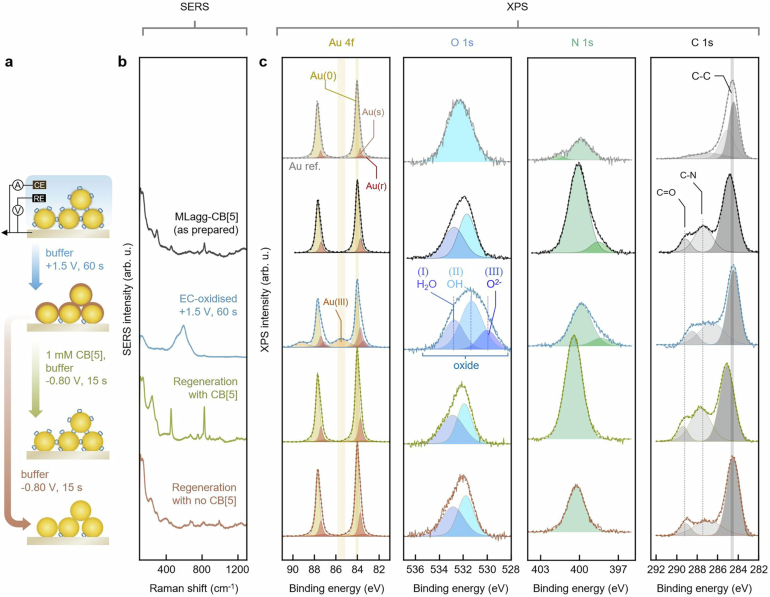
SERS and XPS of MLagg-CB[5] during EC-ReSERS. (**a**) Schematic of the EC-ReSERS protocol. A MLagg-CB[5] substrate, with CB[5] and other ligands such as citrate on the AuNP surface, undergoes electrochemical oxidation (blue arrow), followed by reduction either in the presence of CB[5] to regenerate the nanogap (green arrow) or in its absence as a control (brown arrow). (**b**) SERS spectra of the as-prepared MLagg-CB[5] (black), after oxidation (blue), and after reduction with (green) or without (brown) CB[5]. (**c**) Au 4 f, O 1 s, N 1 s, and C 1 s XPS spectra (solid lines), peak fits (dotted lines), and fitted components of a planar Au reference (grey), compared with MLagg-CB[5] samples as-prepared (black), after oxidation (blue), and after reduction with (green) or without (brown) CB[5]. All XPS spectra were collected at 700 eV and normalized to maximum intensity. The Au reference also shows O 1 s, N 1 s, and C 1 s signals from surface contaminants.

Time-resolved SERS tracks the evolution of AuNP nanogap chemistry while sweeping between oxidizing and reducing potentials in 0.1 mM CB[5] and buffer (Fig. 1c–f). An anodic sweep from 0 to +1.5 V leads to the gradual desorption of CB[5] and the formation of gold oxide above +0.7 V, as confirmed by SERS and XPS (Extended Data Fig. 1 and Supplementary Notes 2–4). Beyond +0.9 V, vibrational modes of the gold oxide lattice emerge at 480, 560 and 630 cm^−1^, in agreement with density functional theory (DFT) calculations (Supplementary Note 5). As the potential increases further, these peaks shift by +30–40 cm^−1^, reflecting increasing oxide density within the nanogap, which remains limited to ∼1–2 monolayers, as estimated by XPS depth profiling (Supplementary Note 3). A cathodic sweep then reduces the gold oxide and readsorbs CB[5] from the bulk solution into the nanogaps, restoring the CB[5] peak to its initial intensity.

**Extended Data Fig. 2:**
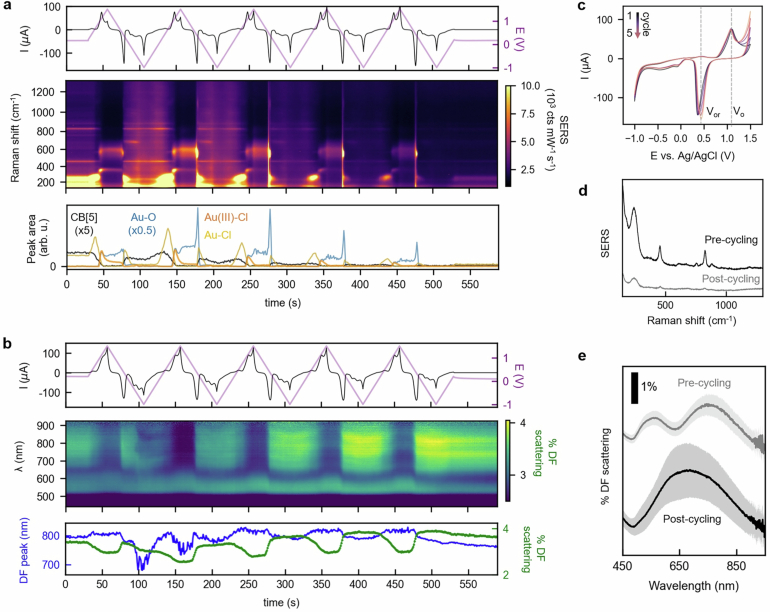
Cyclic voltammetry (CV), SERS, and DF of MLagg-CB[5] in buffer. (**a**) Applied potential vs Ag/AgCl (purple) and current response (black) for a MLagg-CB[5] cycled between +1.5 V and -1.0 V in 50 mM KH_2_PO_4_/K_2_HPO_4_ (pH 7.0) at 50 mV∙s^−1^ for five scans, together with a colourmap of time-series SERS spectra during the potential sweeps and extracted peak areas of CB[5] (826 cm^−1^, grey) Au–O (520, 590, 660 cm^−1^, blue), Au(III)-Cl (345 cm^−1^, orange), and Au–Cl (240-270 cm^−1^, yellow). (**b**) Applied potential vs Ag/AgCl (purple) and current response (black) for an equivalent MLagg-CB[5] sample cycled under the same conditions, together with time-series DF spectra during the potential sweeps and extracted chain-mode peak amplitude and position. (**c**) Overlaid cyclic voltammograms of the five scans. (**d**) SERS and (**e**) DF scattering spectra measured before and after cycling, where solid lines represent the mean and shaded regions the ±1 s.d. of *n* = 150 spectra across the MLagg-CB[5] area.

With CB[5], the nanogap structure remains highly stable over multiple cycles. Repeated cycling shows no degradation in SERS signal. In situ dark field (DF) scattering spectroscopy also shows repeatable changes in peak wavelength and intensity of the coupled plasmon mode (Fig. 1d), while scanning electron microscopy (SEM) shows intact nanogaps (Fig. 1c, top) even as the outer AuNP surfaces exhibit some facet restructuring (Supplementary Note 6). On the other hand, SEM after cycling without CB[5] (Fig. 1c, bottom) shows clear evidence of sintering, where the AuNPs merge and the gap structure collapses^34^. This is further reflected in spectral data (Extended Data Fig. 2), which show decreasing SERS intensity and blue-shifts of the coupled plasmon, consistent with the loss of electromagnetic field enhancement and the weakening of chain mode coupling across the nanogaps^28,32^. On alternative SERS substrates, such as electrochemically roughened gold (Supplementary Note 7), SERS signals also degrade over multiple ORCs, yet remain stable when CB[5] is present, highlighting its essential role in maintaining hotspot integrity.

The reintroduction of the scaffolding molecule is crucial to stabilizing the nanogap. However, how CB[5] returns to restabilize nanogaps during gold oxide reduction remains unclear. A key question is how nanogaps resist sintering during reduction because the CB[5] molecules must diffuse from the bulk and effectively rebind to the freshly reduced facets within the nanogap, which is precisely when the structure is most unstable. Understanding how this dynamic reconstruction occurs without irreversible collapse is essential for revealing the processes that enable repeated EC-ReSERS.

**Extended Data Fig. 3:**
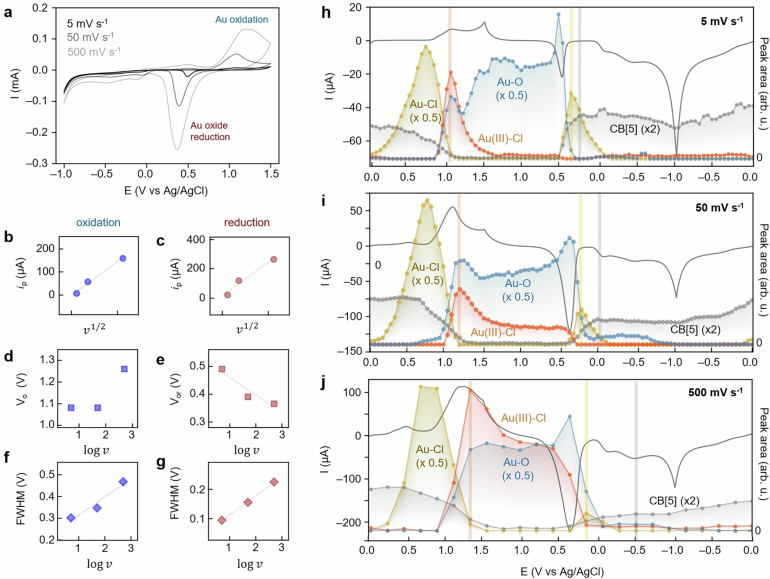
EC-ReSERS at varying scan rates. (**a**) Overlaid cyclic voltammograms of MLagg-CB[5] in 0.1 mM CB[5], 50 mM potassium phosphate buffer at different scan rates (*v*=5, 50, 500 mV·s^−1^). Effect of scan rate on the oxidation and reduction (**b-c**) peak currents (*i*_p_), the (**d-e**) peak potentials and (**f-g**) peak widths (full-width half-maximum, FWHM). (**h-j**) Overlaid voltammograms and extracted time-series SERS peak intensities of Au–Cl (240-270 cm^−1^), CB[5] (826 cm^−1^), Au(III)-Cl (345 cm^−1^), and Au–O (summation of 520, 590, 660 cm^−1^) at different scan rates. Orange line marks potential where Au(III)-Cl intensity reaches its peak, yellow line marks potential where Au–Cl intensity reaches its peak during reduction, and the grey line marks potential where CB[5] intensity starts to plateau.

Closer examination of low-wavenumber SERS lines through a single ORC yields useful insights (Fig. 1e,f). We particularly note the evolution of Au–Cl (240–265 cm^−1^) and Au(III)–Cl peaks (345 cm^−1^; Supplementary Note 5). The Cl^−^ required for the formation of these intermediates originates from the CB[5] reagent itself, which introduces ∼0.5 mM Cl^−^ in our experiments. During the anodic sweep, Au–Cl forms first, followed by the growth of Au(III)–Cl and gold oxide features. On reversing the potential, Au(III)–Cl and gold oxide are not directly reduced back to Au(0). Instead, their reduction is accompanied by the transient reappearance of Au–Cl, which closely precedes the restoration of CB[5]. This temporal alignment highlights Au–Cl as an intermediate during reduction, one that may create favourable conditions for CB[5] to re-enter the gap. Notably, kinetic studies show that slower scan rates promote stronger Au–Cl formation and more robust CB[5] rebinding, suggesting a tight association between Au–Cl formation and nanogap restabilization (Extended Data Fig. 3 and Supplementary Notes 8 and 12).

To probe the influence of electrolyte composition, we tested sulfate, perchlorate and phosphate buffers at both neutral and acidic conditions (Supplementary Note 9). In all cases, Au–Cl bands consistently appear during ORCs, indicating that residual Cl^−^ is sufficient to drive Au–Cl formation. EC-ReSERS remains highly reproducible, with effective CB[5] rebinding and stable SERS signals, underscoring the strong affinity of Cl^−^ for gold over other anions^35^.

**Extended Data Fig. 4:**
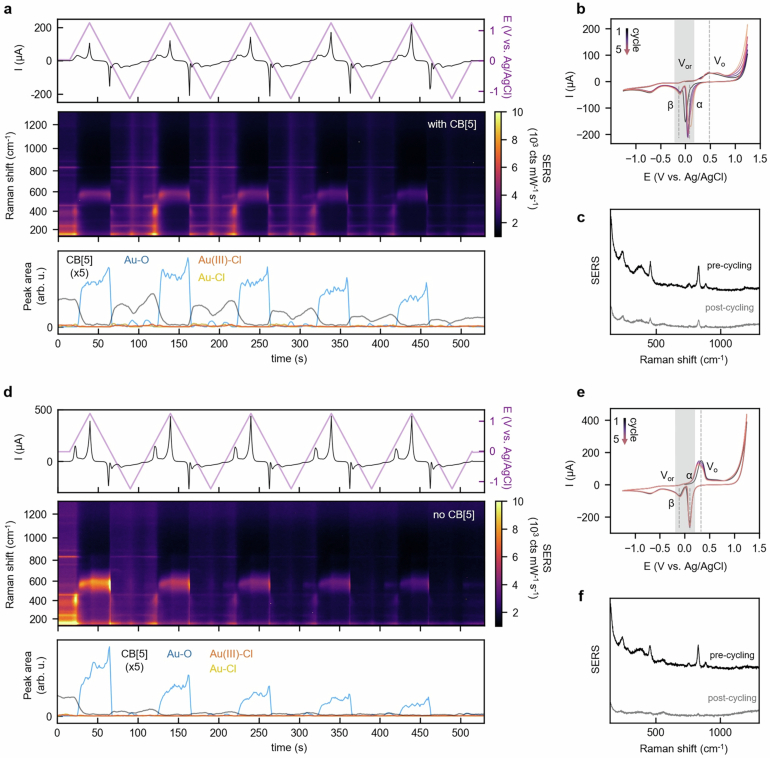
Cyclic voltammetry (CV) and time-series SERS of MLagg-CB[5] in 100 mM NaOH (pH 13.0) with and without CB[5]. (**a,d**) Applied potential vs Ag/AgCl (purple) and current response (black) for a MLagg-CB[5] cycled between +1.5 V and -1.0 V at 50 mV·s^−1^ for five scans, with (**a**) 0.1 mM CB[5] and (**d**) without CB[5]. Colourmaps of time-series SERS spectra acquired during the scans are shown, together with extracted peak areas of CB[5] (826 cm^−1^, grey), Au–O (590 cm^−1^, blue), Au(III)-Cl (345 cm^−1^, orange), and Au–Cl (240-270 cm^−1^, yellow). (**b,e**) Overlaid cyclic voltammograms of the five scans corresponding to (**a**) and (**d**). (**c,f**) SERS spectra of MLagg-CB[5] before and after cycling, measured (**c**) with and (**f**) without CB[5].

**Extended Data Fig. 5:**
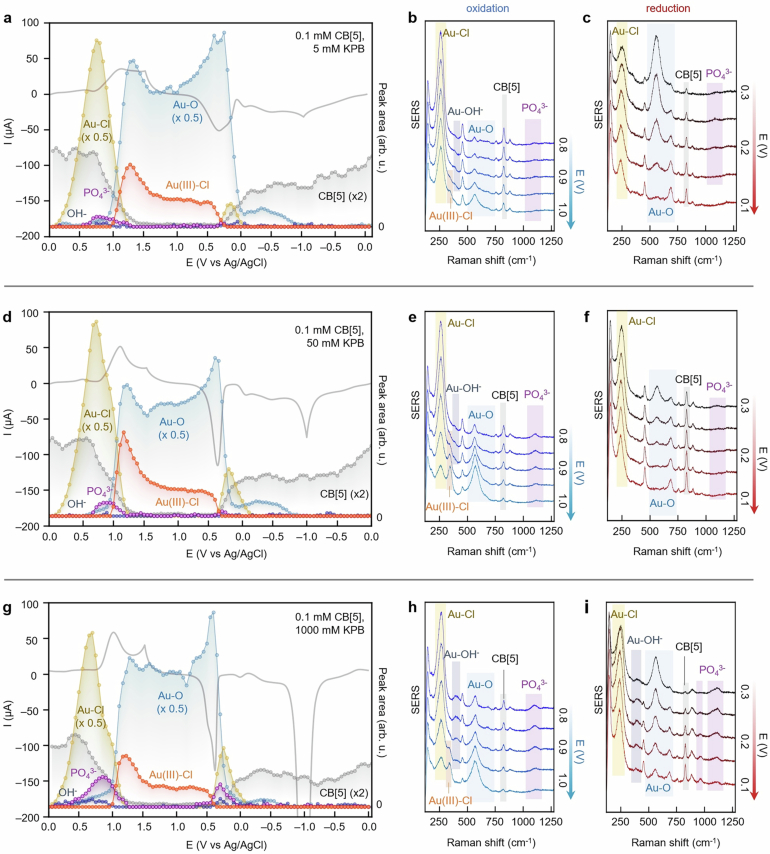
Surface transformations of MLagg-CB[5] cycled in different buffer concentrations. Voltammograms and extracted time-series SERS peak areas of Au–Cl (240-270 cm^−1^), CB[5] (826 cm^−1^), Au(III)-Cl (345 cm^−1^), and Au–O (summation of 520, 590, 660 cm^−1^), PO_4_^3−^ (1100 cm^−1^), and Au–OH^−^ (400 cm^−1^) of MLagg-CB[5] cycled between +1.5 V and -1.0 V at 50 mV·s^−1^ in 0.1 mM CB[5] and (**a**) 5 mM, (**d**) 50 mM, and (**g**) 1000 mM potassium phosphate buffer (KPB), pH 7.0. Corresponding offset SERS spectra during (**b,e,h**) oxidation and (**c, f, i**) reduction with key SERS features highlighted.

**Extended Data Fig. 6:**
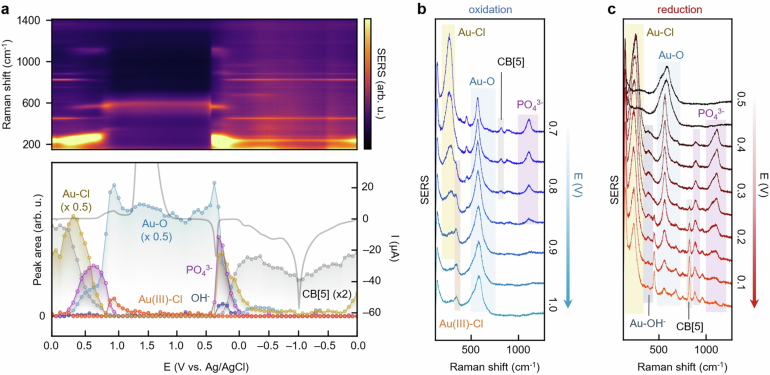
Surface transformations of MLagg-CB[5] slowly cycled in 1 M phosphate buffer. (**a**) Time-series SERS spectra of MLagg-CB[5] cycled between +1.5 V and -1 V at 5 mV∙s^−1^ in 0.1 mM CB[5] and 1 M potassium phosphate buffer (pH 7.0). Overlaid voltammogram and extracted time-series SERS peak areas of Au–Cl (240-270 cm^−1^), CB[5] (826 cm^−1^), Au(III)-Cl (345 cm^−1^), and Au–O (summation of 520, 590, 660 cm^−1^), PO_4_^3−^ (1100 cm^−1^), and Au–OH (400 cm^−1^). Corresponding offset SERS spectra during (**b**) oxidation and (**c**) reduction with key SERS features highlighted.

By contrast, NaOH fails to support Au–Cl formation, instead favouring the formation of Au–OH (Extended Data Fig. 4). Under these conditions, CB[5] rebinding is hindered, and performance deteriorates over successive cycles. Similarly, at high buffer concentrations, the compressed Debye length and excess anions (OH^−^ and phosphate) screen surface charges, leading to inefficient CB[5] rebinding and loss of nanogap integrity (Extended Data Fig. 5). Notably, even when using slow scan rates, CB[5] rebinding remains delayed until the excess anions are cleared from the nanogap, confirming that the interfacial environment plays a more dominant role than diffusion kinetics in governing the efficiency of rescaffolding (Extended Data Fig. 6 and Supplementary Note 8).

The presence of Au–Cl during the reduction step is strongly associated with successful CB[5] rescaffolding. In contrast, conditions that suppress Au–Cl formation or cause excessive charge screening result in destabilized nanogaps and performance degradation over time. This points to Au–Cl as a critical intermediate that enables effective nanogap regeneration. Given this central role of Au–Cl, we now focus on deeper examination of its properties.

### Au–Cl adlayer

**Fig. 2:**
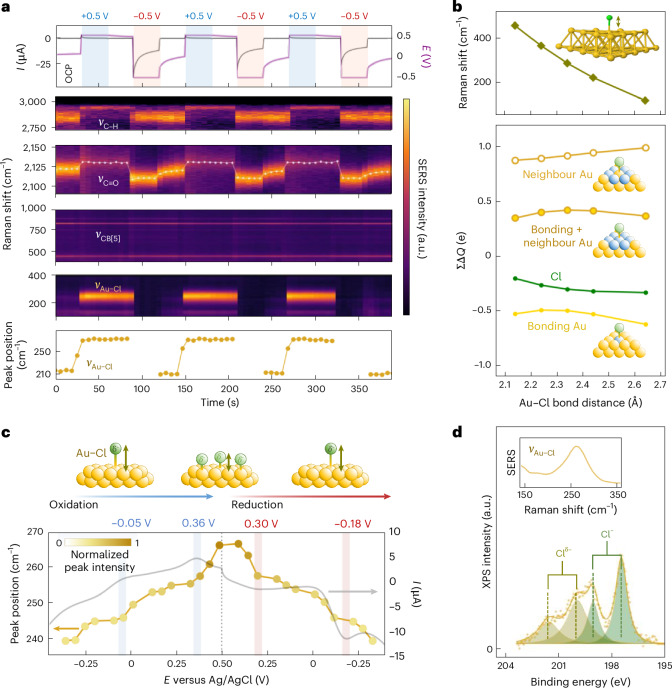
Formation of Au–Cl adlayer on MLagg-CB[5]. **a**, Real-time SERS during potential step experiments in 50 mM potassium phosphate buffer (pH 7.0) containing 0.1 mM CB[5] and 0.5 mM Cl^−^, alternating between +0.5 V and −0.5 V with OCP relaxation between steps. Separate panels highlight the Au–Cl peak, the CB[5] fingerprint region, C≡O stretch and the CB[5] C–H stretch. For the Au–Cl region, underlying CB[5] modes at 240 and 280 cm^−1^ were subtracted for clarity. Intensities are scaled, and the C≡O peak position is overlaid on the SERS spectra; the Au–Cl peak position is tracked in a separate panel. **b**, DFT analysis of the Au–Cl stretch as a function of bond distance. Lower panel shows Mulliken charges (Δ*Q*) on chlorine (green) and gold (yellow) atoms at different bond distances. The summed charge of selected neighbouring gold atoms (blue) is also shown. **c**, Spectrovoltammogram (50 mV s^−1^) showing currents associated with Au–Cl adlayer formation, with Au–Cl peak position (points) and intensity (shaded points) versus applied potential. Shaded blue and pink bands mark oxidation and reduction peaks associated with phase-like transitions. Accompanying schematic illustrates the relationship between adlayer density and Au–Cl bond distance. **d**, Chlorine 2*p* XPS spectrum of MLagg-CB[5] (450 eV photon energy) featuring a prominent Au–Cl SERS peak (inset).

To study the formation and stability of Au–Cl systematically, we turn to controlled anodic potential step measurements (Fig. 2a). When the MLagg is stepped to +0.5 V, the open-circuit potential (OCP) relaxes to and stabilizes at +0.5 V. Concomitantly, the Au–Cl vibrational band intensifies, shifts from 240 to 260 cm^−1^, and remains prominent even after OCP relaxation. Upon applying −0.5 V, the Au–Cl peak disappears but re-emerges weakly at 240 cm^−1^ after returning to OCP ≈ 0 V, consistent with partial Cl^−^ rebinding. These observations suggest that Au–Cl forms a stable reducible surface adlayer and invites closer examination of the bond chemical nature.

**Extended Data Fig. 7:**
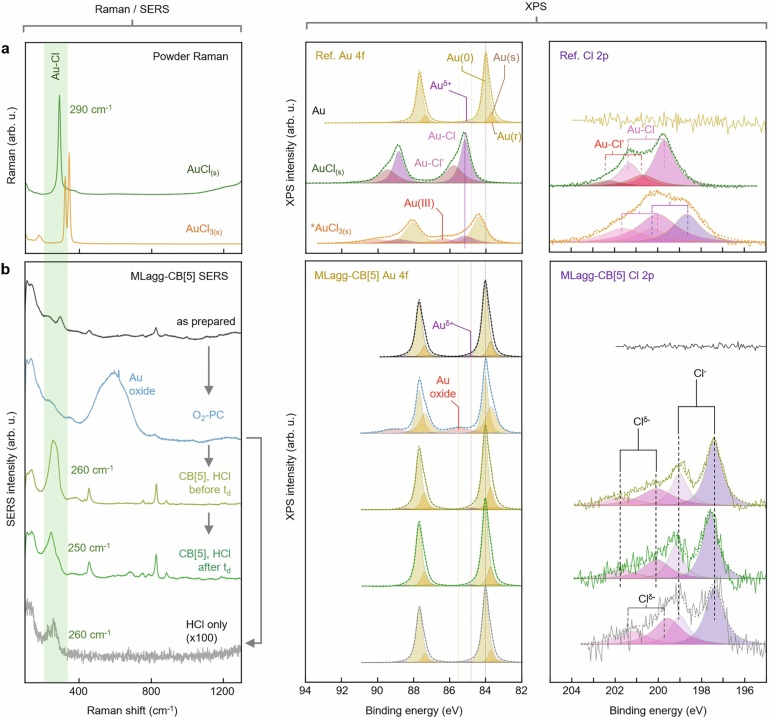
SERS and XPS of reference and MLagg samples after various treatments. (**a**) Raman spectra and XPS spectra (Au 4 f and Cl 2p; solid lines), fits (dotted), and fitted components of reference samples: AuCl_(s)_ powder and AuCl_3(s)_ powder. The AuCl_3(s)_ powder shows signs of in-situ reduction during measurement, forming AuCl and Au(0). The XPS spectra of planar Au are also shown for reference. (**b**) Corresponding SERS and XPS spectra of MLagg-CB[5] samples are shown as prepared, after 45 min of oxygen plasma cleaning, after treatment with CB[5]+HCl before and after transition time t_d_, and after treatment with HCl only. All XPS spectra were acquired at 700 eV and normalised to maximum intensity.

Metal–chloride interactions are generally considered to be ionic, as in the cases of Ag–Cl and Hg–Cl^36^, but a growing body of evidence suggests that Au–Cl displays significant covalent character^36–42^. DFT calculations and complementary spectroscopy confirm the covalent nature of Au–Cl. Upon formation of Au–Cl on Au(111), the chlorine atom becomes only partially negative (−0.3*e* Mulliken, *D* = 2.34 Å), while the bonded gold atom becomes electron rich (−0.5*e*; Fig. 2b), reflecting significant electron sharing rather than full electron transfer^36,39,40^. Notably, the formation of Au–Cl electronically perturbs the surrounding gold lattice. The six nearest-neighbour gold atoms gain a total charge of +0.9, creating a delocalized polarization field characteristic of covalent interactions. Spectroscopic evidence aligns with these predictions. Low-energy synchrotron XPS (Supplementary Note 11) shows a subtle shift of the gold 4*f* peaks to 84.8/88.4 eV, slightly above Au(0) at 84.0/87.7 eV, reflecting partial positive charge redistribution in gold. Chlorine 2*p* spectra further differentiate between weakly bound Cl^−^ (197.6/199.2 eV) and the presence of Cl^*δ*−^ (200.2/201.8 eV), which is strongly indicative of the covalent Au–Cl interaction^40,43^ (Fig. 2d and Extended Data Fig. 7). Supporting this interpretation, DF scattering reveals stable and reversible approximately nanometre redshifts and ∼5% decrease in scattering intensity upon Au–Cl formation (Supplementary Note 10), indicating reduced free-electron density at the gold surface^44–46^.

The covalent Au–Cl bond is not static but evolves dynamically with applied potential. SERS reveals a shift of the Au–Cl peak from 240 to 265 cm^−1^ during anodic sweeps, reflecting transitions from a sparse, disordered adlayer to a denser, more ordered one^37^. Two discrete anodic peaks (Fig. 2c and Supplementary Note 12) mark these transitions, where enhanced interadsorbate repulsion drives electrocompression and increases charge transfer between gold and chlorine^40,42^. DFT shows that greater charge transfer shortens the Au–Cl bond and upshifts the stretching mode (Fig. 2b), linking the observed peak shift to previously reported trends relating increased adlayer density to enhanced Au–Cl covalency^39,40,42^.

**Extended Data Fig. 8:**
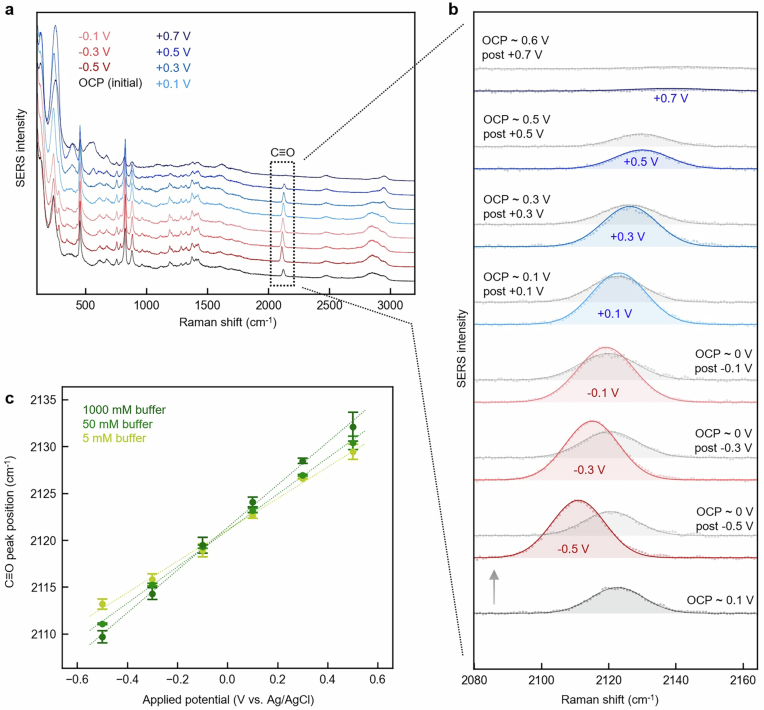
Electrochemical Stark shifts of adsorbed CO on MLagg-CB[5]. (**a**) SERS spectra measured on MLagg-CB[5] in 0.1 mM CB[5], 0.5 mM Cl^−^, and 50 mM potassium phosphate buffer (pH 7.0) at the initial OCP and different applied potentials. The highlighted peak at 2110-2140 cm^−1^ is assigned to the CO vibrational mode. (**b**) SERS spectra (red and blue) within the CO peak wavenumber region at different applied potentials. Also shown are the spectra (grey) measured at OCP after the corresponding applied potential. Spectra are offset for clarity; the arrow indicates the chronological order of measurement from bottom to top. (**c**) Fitted peak position of the CO vibrational mode at varying applied potentials in 0.1 mM CB[5], 0.5 mM Cl^−^ and different buffer concentrations (5 mM, 50 mM, or 1000 mM) and their corresponding linear fits. Points are plotted as mean ± 1 s.d. from measurements of CO peak position on *n* = 3 different MLagg-CB[5] substrates.

The charge redistribution resulting from Au–Cl adlayer formation significantly influences the interfacial electronic environment. Formation of Au–Cl leads to a stable OCP of 0.5–0.7 V, depending on surface coverage (Supplementary Note 7), consistent with an increase in work function from the formation of an outward-pointing surface dipole (Au^*δ*+^ → Cl^*δ*−^)^47,48^. This implies that Au–Cl modifies the local electronic potential, generating an electric field at the interface. Complementary CO Stark shift measurements (Extended Data Fig. 8 and Supplementary Note 10) reinforce this interpretation, showing a stable +13-cm^−1^ shift in the CO stretch mode upon Au–Cl formation (Fig. 2a). This directly indicates the presence of a local electric field^49^, supporting the conclusion that the Au–Cl adlayer establishes a stable surface dipole that shapes the interfacial electrostatics.

**Fig. 3:**
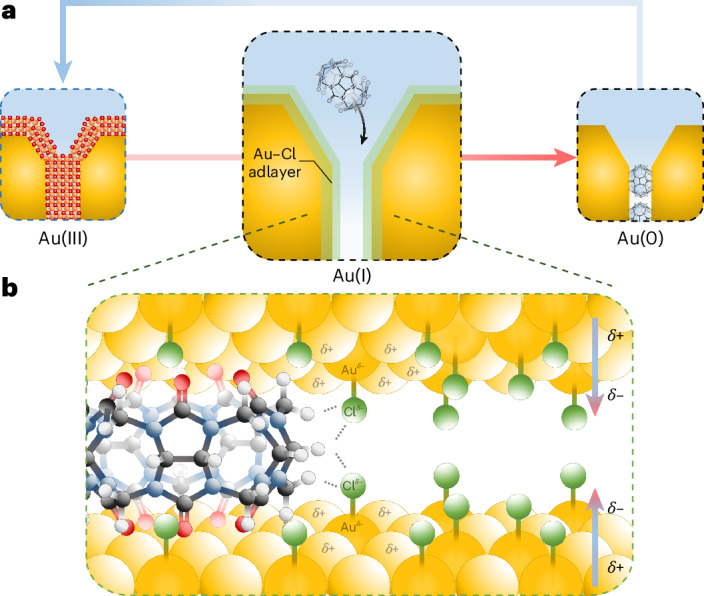
Schematic illustration of Au–Cl role in EC-ReSERS nanogap regeneration. **a**, Electrochemical reduction (red arrow) in the presence of Cl^−^ transforms the gold oxide-coated nanogap (left) into an intermediate state featuring a Au–Cl adlayer (centre). Further reduction enables regeneration of the nanogap (right) with CB[5] stabilization and enhanced SERS sensitivity. The regenerated nanogap can undergo oxidation again (blue arrow) for continued cycling. The primary oxidation state of surface gold atoms is labelled per stage. **b**, Close-up of Au–Cl-coated nanogap showing partial negative charges on chlorine (green), partial negative charge on bonding surface gold (dark yellow), and partial positive charges on non-bonding neighbouring gold atoms (light yellow). For clarity, charges are indicated only on selected gold atoms, and countercations and other electrolyte anions are omitted. Charge redistribution generates an outward-pointing surface dipole (Au^*δ*+^ → Cl^*δ*−^), and favourable interactions with CB[5] carbonyl portals and C–H groups, illustrating how the Au–Cl adlayer modifies the local electrostatic environment to support efficient nanogap rescaffolding. Atoms not drawn to scale.

The stable Au–Cl surface dipole creates an interfacial environment that is ideally suited for nanogap regeneration (Fig. 3). By suppressing surface reconstruction^37,41^, the Au–Cl layer stabilizes surface atoms and limits atomic diffusion within individual gold facets. At the same time, outward-pointing Au–Cl dipoles on opposing gold facets generate electrostatic repulsion across the nanogap, countering sintering forces and preserving the nanogap architecture prior to CB[5] reintroduction as a steric stabilizer. Meanwhile, the partial positive polarization of the gold surface attracts the electron-rich carbonyl portals of CB[5], facilitating oriented rebinding. High-wavenumber SERS also reveals that C–H stretches of CB[5] respond sensitively to the Au–Cl adlayer (Fig. 2a and Supplementary Note 10), supporting the role of previously established CB[*n*] (C–H)–(Cl–Au) dipole–dipole interactions^50,51^ in further attracting CB[5] into the gap. As CB[5] re-enters at this stage, continued cathodic sweeping reduces the remaining Au–Cl species, facilitating displacement of Cl^−^ and allowing CB[5] to dominate the nanogap interface. This ligand exchange completes the regeneration cycle, re-establishing the CB[5]-stabilized architecture.

In contrast, the Au–OH adlayer formed under alkaline conditions exhibits different interfacial properties that fail to support nanogap rescaffolding. Unlike the covalent Au–Cl adlayer, previous DFT analyses confirm that Au–OH is predominantly ionic^52^. Consequently, the electron density remains localized on OH^−^, and the surface potential remains strongly negative (−110 mV)^53,54^. This environment is electrostatically unfavourable for CB[5] rebinding because the carbonyl portals of CB[5] would experience repulsion rather than attraction. These contrasting interfacial properties highlight why Au–Cl, but not Au–OH, supports efficient nanogap regeneration and ligand rebinding in EC-ReSERS.

### Chemical rescaffolding (Ch-ReSERS)

**Fig. 4:**
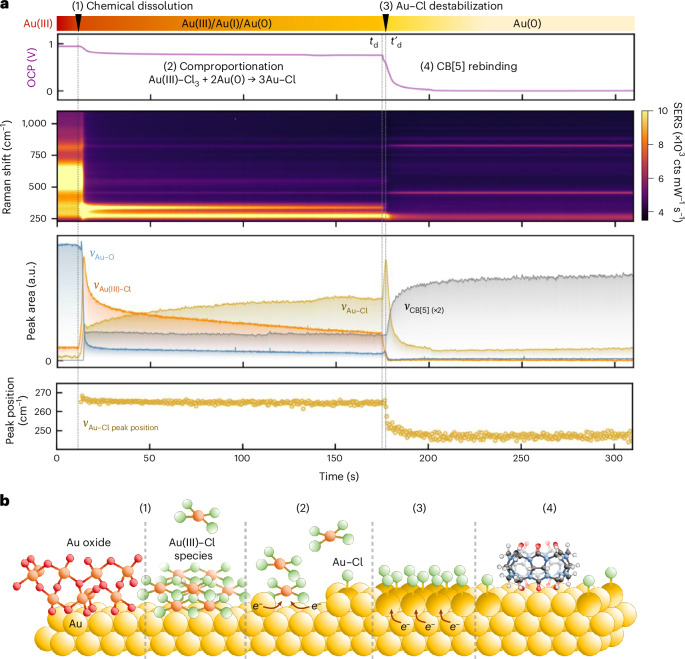
Time-series SERS and OCP of an oxidized MLagg-CB[5] undergoing Ch-ReSERS. **a**, Time-series OCP and SERS spectra (0.1-s integration time) from MLagg-CB[5] initially oxidized by oxygen plasma, followed by incubation with 100 µl 1 mM CB[5] in 0.5 M HCl at point (1). Extracted peak areas of Au–O (590 cm^−1^), Au(III)–Cl (340–365 cm^−1^), Au–Cl (240–265 cm^−1^) and CB[5] (826 cm^−1^) are shown, along with the Au–Cl peak position (bottom panel). **b**, Schematic of surface transformations during Ch-ReSERS illustrating (1) chemical dissolution of gold oxide to form Au(III)–Cl species, (2) comproportionation of Au(III)–Cl with Au(0) to form Au–Cl, (3) Au–Cl adlayer destablization and (4) CB[5] rebinding. Arrows indicate conceptual electron flow during redox processes. Note that the structures of gold oxide and Au(III)–Cl shown are intended to illustrate key transitions in Ch-ReSERS.

The Au–Cl adlayer plays a critical role in stabilizing the nanogap environment and enabling effective CB[5] rebinding in EC-ReSERS. Au–Cl plays a similar role in an alternative chemical rescaffolding protocol, Ch-ReSERS^28,32^. Because Ch-ReSERS is driven by spontaneous chemical transformations rather than externally applied potentials, Au–Cl is found to also modulate surface redox chemistry. Here, the MLagg is first oxidized with oxygen plasma (Supplementary Note 4) and then treated with CB[5] and HCl (Supplementary Note 14). Time-resolved SERS and OCP measurements capture the sequence of surface transformations (Fig. 4): the gold oxide peak decreases as the Au(III)–Cl band emerges, indicating chemical dissolution of the oxide and generation of a high local concentration of Au(III)–Cl species. A rise in the Au–Cl peak suggests rapid comproportionation between Au(III)–Cl and surface Au(0) to produce a Au–Cl adlayer as the OCP stabilizes at +0.7 V. A weak CB[5] signal also appears at this stage, consistent with initial binding at the Au–Cl interface, further evidenced by characteristic high-wavenumber C–H lines (Supplementary Fig. 54).

Ch-ReSERS proceeds through spontaneous comproportionation without the addition of external reducing agents, thus relying solely on the redox interplay between Au(0), Au(I) and Au(III) at the interface. As the Au–Cl adlayer grows denser, it maintains an elevated work function and progressively depletes electron density from the surrounding Au(0), making further oxidation thermodynamically less favourable. This slows comproportionation and stabilizes the intermediate state, effectively gating further redox transformations.

At a critical point, however, the system destabilizes and initiates a cascade of redox events. The start of this transition is marked by a sharp OCP drop from +0.7 to +0.6 V (Fig. 4a, *t*_d_). We propose this indicates a local destabilization of Au–Cl, where electrostatic repulsion and strain exceed the adlayer capacity to accommodate further compression. Typically, this instability would induce adlayer phase transitions to relieve strain^43,55,56^. However, under oxidizing conditions, electron transfer from Au(0) to Au–Cl becomes favourable, causing a local collapse of the surface dipole and a drop in work function. This local collapse then rapidly propagates across the entire gold surface, triggering a rapid final burst of comproportionation, reflected in a sharp Au(III)–Cl decrease and a simultaneous rapid increase in Au–Cl. As the Au–Cl line reaches its maximum (Fig. 4a, *t*′_d_), the adlayer achieves its densest configuration, driving additional strain. This state becomes even more unstable, initiating a second reduction stage: Au(0) transfers electrons to the Au–Cl adlayer, leading to a rapid OCP drop to 0 V, a decrease in intensity and shift in the Au–Cl peak from 265 to 240 cm^−1^, and replacement of Cl^−^ by CB[5]. Notably, this reduction process leaves the underlying gold substrate transiently electron deficient, thus enhancing the electrostatic attraction between gold and the carbonyl portals of CB[5], promoting rapid ligand rebinding and nanogap restabilization. This sequence of events emphasizes the central role of the Au–Cl adlayer as a dynamic modulator of surface redox processes, actively impacting electron redistribution and interfacial restructuring during Ch-ReSERS.

**Extended Data Fig. 9:**
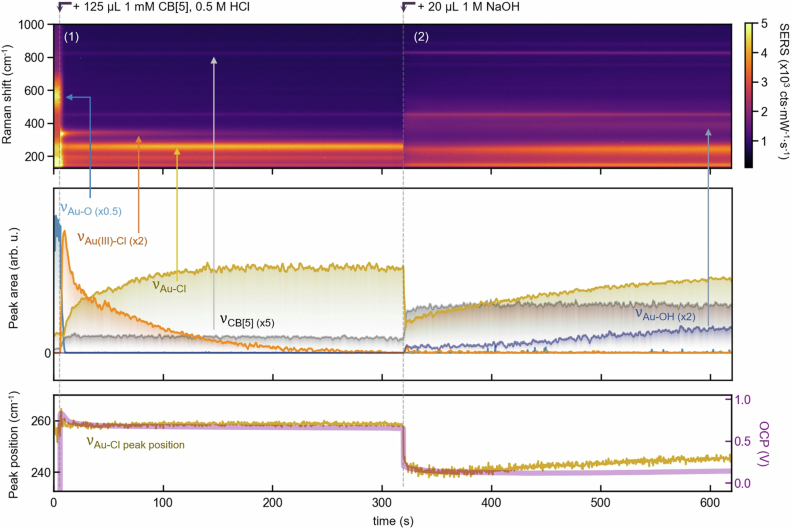
Au–Cl adlayer formed during Ch-ReSERS and destabilised by OH^−^. Time series SERS spectra of an oxidised MLagg-CB[5] treated with 1 mM CB[5] and 0.5 M HCl at point (1) and allowed to equilibrate. At point (2), an aliquot of 1 M NaOH is dropped into the electrolyte solution, resulting in shifts in the equilibrium. Extracted peak areas of CB[5] (826 cm^−1^, grey line) Au–O (sets of peaks centred at 590 cm^−1^, blue line), Au(III)-Cl (345 cm^−1^, orange line), Au–Cl (240-270 cm^−1^, yellow line), and Au–OH (400 cm^−1^, dark blue line). The evolution of the Au–Cl peak position is also plotted together with the measured open-circuit potential evolution (purple).

This redox-driven collapse of the Au–Cl adlayer illustrates its dynamic and metastable nature. We also observe that the adlayer is chemically labile: the addition of OH^−^, even in trace amounts, can displace Cl^−^ from the Au–Cl interface to form Au–OH, collapsing the surface dipole and inducing OCP drops similar to those triggered by redox-driven collapse (Extended Data Fig. 9 and Supplementary Notes 13 and 15). These observations highlight the central role of surface dipoles in governing interfacial properties and demonstrate that the electronic behaviour of AuNP surfaces can be modulated not only through applied potential, but also via subtle changes in the solution environment.

**Extended Data Fig. 10:**
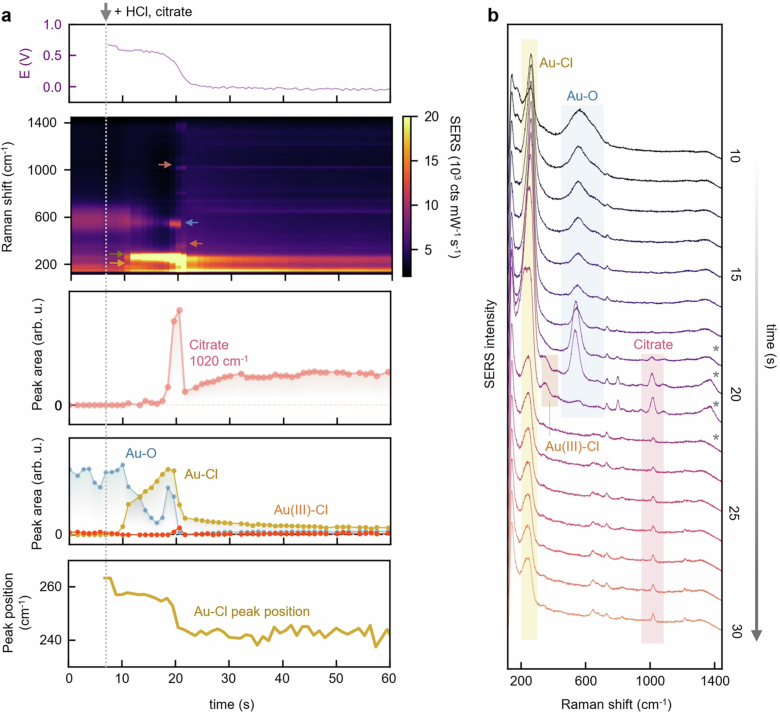
Ch-ReSERS with citrate. (**a**) Time-series SERS spectra of an oxidised MLagg-CB[5] substrate incubated with 100 µL of 0.5 M citrate in 0.5 M HCl. From the onset of incubation, the open-circuit potential (OCP vs Ag/AgCl, purple) and SERS spectra were recorded, with coloured arrows indicating the peak areas extracted below. (**b**) Time-series SERS spectra near the OCP transition, offset for clarity. Spectral features of interest are highlighted. The time axis (10-30 s) corresponds to the time window in (**a**), with spectra marked by asterisks (*) captured during the OCP drop from +0.5 V to 0 V.

The insights from Ch-ReSERS extend beyond the MLagg platform, offering a mechanistic window into dynamic redox transformations at gold interfaces. The stepwise transformations between Au(III), Au(I) and Au(0) mirror redox pathways proposed in colloidal AuNP synthesis^15,22^, electrodeposition^57,58^ and dissolution^59,60^. We show here that rather than adsorbed [AuCl_2_]^−^, the Au–Cl adlayer is the key Au(I) interfacial species that bridges Au(III) with the Au(0) surface, consistent with recent high-resolution X-ray absorption spectroscopy evidence of surface Au–Cl species^61^. To probe the relevance of these redox transformations to AuNP synthesis, where citrate serves as both a reducing agent and a stabilizing ligand, we substituted CB[5] with citrate in Ch-ReSERS (Extended Data Fig. 10 and Supplementary Note 16). The results show that citrate accelerates the reduction of Au(III)–Cl species to Au–Cl, with the final transformation to Au(0) marked by an OCP drop that mirrors colloidal AuNP growth dynamics^62^, signalling the completion of reduction and subsequent citrate binding to the metallic surface. These findings further highlight that Au–Cl acts as a key redox-active surface species in chloride-containing environments, governing electron redistribution and interfacial reactivity in a broad range of gold-based processes.

## Conclusions

Surface adlayers, such as Au–Cl, play pivotal roles in modulating the electronic structure at nanoscale interfaces, influencing the local potential and ligand interactions. In EC-ReSERS, we show that the formation of a transient Au–Cl adlayer stabilizes nanogap architectures by facilitating CB[5] rescaffolding and reproducible SERS regeneration. In contrast, conditions that favour Au–OH formation lead to poor regeneration. In Ch-ReSERS, we find that Au–Cl adlayers act as key interfacial Au(I) intermediates that bridge redox transitions between Au(III) and Au(0) species, directly participating in surface redox processes. Linking adlayer formation with redox dynamics and ligand rebinding highlights the importance of interfacial chemistry for stable, reusable SERS substrates. More broadly, these findings advance our understanding of how adlayer chemistry shapes interfacial properties and reactivity, paving the way for engineered nanoscale interfaces with enhanced performance across electrochemical, catalytic and sensing applications.

## Methods

### Materials

All chemical reagents were used as received. Citrate-stabilized AuNPs (80 nm, optical density 1.0 at 555 nm) were from BBI Solutions. CB[5] hydrate (∼20 wt% H_2_O, CB[5]·*x*HCl·*x*KCl), AuCl (99.9% metal basis), AuCl_3_ (99% metal basis), ascorbic acid (≥99%), NaBH_4_ (99%), sodium deuteroxide solution (30 wt% in D_2_O) were from Sigma-Aldrich. Analytical grade chloroform, H_2_SO_4_ (98%), HCl (37%), HClO_4_ (70%), H_3_PO_4_ (86%) and deuterium chloride (1 M in D_2_O) were from Fisher Scientific. NaCl (≥99%), K_2_HPO_4_ (≥98%) and KH_2_PO_4_ (≥98%) were from Alfa Aesar. NaOH (≥98%) was from Acros Organics. Polydimethylsiloxane (PDMS) slabs were prepared using a SYLGARD 184 kit from DOWSIL (Dow Silicones). Fluorine-doped tin oxide (FTO)-coated glass slides (TEC 10) were purchased from Ossila and were cleaned and cut to 10 × 15 mm^2^ or 10 × 7.5 mm^2^ slides prior to use. All aqueous solutions were prepared using deionized water (≤4.3 µS cm^−1^) or D_2_O (99.9%) from Sigma-Aldrich.

### Multilayer aggregate preparation

MLagg substrates were prepared^28,29,32^ by mixing 500 µl of citrate-stabilized, aqueous-based 80-nm AuNPs with an equal volume of chloroform, and initiating aggregation with the addition of 25 µl of 2 mM CB[5] or 50 µl of 1 M NaCl. Aggregation was facilitated with 1 min of vigorous shaking, after which the aggregates settled at the liquid–liquid interface. The AuNP aggregates were then washed with deionized water by repeatedly (at least three times) concentrating and diluting the aqueous phase, followed by a final concentration step in which the volume was reduced to microlitres. The AuNP aggregate was then transferred via pipette to a cleaned FTO-coated glass slide and allowed to air dry. The deposited MLagg was further rinsed with deionized water and dried with compressed nitrogen.

### Cleaning and rescaffolding

Organic molecules can be removed from the freshly prepared MLagg surface with oxidative cleaning procedures. Two methods were used to perform initial cleaning: oxygen plasma cleaning using 90% radiofrequency power and 30 cm^3^_STP_ min^−1^ (Henniker Plasma, HPT-100) for 45 min, and electrochemical oxidation via application of +1.5 V for at least 30 s in 1 M potassium phosphate buffer (pH 7.0). After cleaning, the scaffolding ligand was reintroduced by incubating the oxidized MLagg in 1 mM CB[5] prepared in 0.5 M HCl for at least 5 min., followed by rinsing with deionized water and drying with nitrogen. Alternatively, the oxidized MLagg can also be rescaffolded by applying a potential of −0.8 V for at least 5 s in 1 mM CB[5] in neutral electrolyte such as potassium phosphate buffer.

### Raman and DF measurements

Raman spectra were collected using a custom set-up^32^ with an Andor Newton 970 EMCCD camera coupled to a Shamrock 168 spectrometer and a Matchbox 785-nm diode laser. Excitation and collection were collected through an Olympus LUMPlanFl/IR ×40 W numerical aperture (NA) 0.80 water-immersion objective at 1-s integration times with 1-mW laser power. Additional Raman measurements were taken on a Renishaw inVia Raman microscope with a 785-nm excitation laser using a ×20 NA 0.40 objective.

DF scattering measurements were collected using a modified Olympus BX51 with an Ocean Optics QE-Pro spectrometer. Excitation and collection were performed through an Olympus MPLanFL N ×20 BD NA 0.45 objective with 500-ms integration time. A white light scattering target (Labsphere) was used as a reference to normalize white light scattering.

### EC-SERS and EC-DF set-up

Spectroelectrochemical cells were designed and fabricated to measure time-resolved SERS and DF scattering spectra with simultaneous electrochemical measurements. An EC-SERS cell (Supplementary Fig. 1a) was fabricated from PDMS to accommodate a three-electrode electrochemical system: a platinum wire (Sigma-Aldrich) counter-electrode, a leakless Ag/AgCl/KCl reference electrode (LF-1-45, Innovative Instruments), and a MLagg SERS substrate on FTO-coated glass as the working electrode. The electrolyte compartment was defined using a 6-mm-diameter biopsy punch. Using custom 3D-printed stage holders, the EC-SERS cell was sealed and mounted onto the stage of an inverted Raman set-up, with SERS probed from below the cell. To measure time-resolved EC-DF spectra, another spectroelectrochemical cell was machined from Teflon to accommodate the same three-electrode electrochemical system (Supplementary Fig. 1b). The EC-DF cell was mounted below the objective, with DF scattering measured through a 0.13- to 0.16-mm coverslip and a thin (0.5-mm) layer of electrolyte. Electrochemical measurements were conducted using a portable potentiostat (CompactStat) from Ivium Technologies in static conditions. All potentials were referenced to the Ag/AgCl reference electrode.

### SEM measurements

SEM imaging of MLaggs deposited on FTO-coated glass was conducted using a FEI Philips Dualbeam Quanta 3D SEM (dwell, 3–10 µs; high voltage, 2 kV, current, 50 pA; working distance, ∼2.0 mm).

### XPS

XPS was conducted at the I09 Beamline of the Diamond Light Source synchrotron facility. XPS spectra were recorded using four different X-ray photon energies of 200, 450, 700 and 1,100 eV. MLagg samples of various treatments were prepared on a 100-nm gold layer with a 3-nm chromium adhesion layer on a conductive silicon substrate. Spectral data were analysed using CasaXPS^63^. Binding energies of gold samples were calibrated using both the Fermi edge and the gold 4*f*_7/2_ peak at 83.96 eV. For powder samples, binding energies were calibrated using the carbon 1*s* of adventitious carbon at 284.8 eV as the internal reference, which is commonly present in powder samples exposed to air.

### Data analysis

SERS spectra were background corrected, where applicable, to remove the broad glass signal centred at 1,400 cm^−1^ from back-side optical measurements. SERS peak areas were determined by first defining a spectral region, fitting a polynomial background, and fitting Gaussian curves to the peaks of interest. To extract Au–Cl vibrational features from overlapping CB[5] peaks in EC-SERS spectra, a spectral subtraction method was used. A reference spectrum of CB[5] was acquired at −1.0 V, where Cl^−^ was desorbed and no Au–Cl signal was present. This spectrum was scaled according to the peak area of the CB[5] scissoring mode at 450 cm^−1^ and subtracted from spectra at other applied potentials, with the assumption that low-wavenumber CB[5] modes (240, 280 cm^−1^) scale proportionally with the 450 cm^−1^ mode. CB[5]-subtracted spectra were used to assess Au–Cl peak evolution as a function of potential (Supplementary Fig. 55).

DF scattering spectra were analysed by fitting a Gaussian curve to the MLagg coupled plasmon mode. Peak wavelengths and intensities were determined from the centre of the fitted Gaussian.

### DFT methods

We constructed several structures, each formed by a gold nanoparticle connected to one or several chlorine atoms, as shown schematically in Supplementary Fig. 36. We used the Gaussian g16^64^ implementation of DFT to find their ground-state geometries and calculate their vibrational properties, including Raman spectra. For this, we employed tight convergence criteria and LSDA functionals. We then displaced chlorine atoms from their optimum positions and calculated Raman spectra and Mulliken charges on each atom. DFT calculations on Au_2_O_3_ were performed using VASP^65–67^, employing the projector augmented wave (PAW) method to describe the interactions between core and valence electrons^68^. The calculations utilized the Au and O POTCAR files from the PAW_PBE_64 set. The PBEsol functional^69^ was used for all calculations with a Hubbard *U* correction of 0.702 eV for the Au d states^70^. A plane wave kinetic energy cutoff of 600 eV was used, increased to 780 eV for calculations involving variable cell volumes, with a *k*-point sampling density of 0.32 Å^−1^. The structure was relaxed until the forces acting on each atom were less than 5 × 10^−4^ eV Å^−1^. Calculations to determine force constants and scalar-averaged Raman intensities were pre- and post-processed using phonopy^71,72^ and the phonopy-spectroscopy packages^73^.

## Online content

Any methods, additional references, Nature Portfolio reporting summaries, source data, extended data, supplementary information, acknowledgements, peer review information; details of author contributions and competing interests; and statements of data and code availability are available at 10.1038/s41557-025-01989-4.

## Supplementary information

Supplementary Information Supplementary Notes 1–16, Tables 1 and 2, and Figs. 1–55.

## Data Availability

The underlying data that support the findings of this study are available from the paper, its supplementary information or the Cambridge Open Data archive^74^, and from the corresponding author upon request.
