# Supplementary material for: Transient Au–Cl adlayers modulate the surface chemistry of gold nanoparticles during redox reactions
Source: Nat Chem. 2025 Nov 13;18(2):294–301. doi: 10.1038/s41557-025-01989-4 (PMC12872461; doi:10.1038/s41557-025-01989-4)
Supplement: Supplementary file 1 — Supplementary Notes 1–16, Tables 1 and 2, and Figs. 1–55. [file 41557_2025_1989_MOESM1_ESM.pdf]

nature chemistry

Supplementary information https://doi.org/10.1038/s41557-025-01989-4

Transient Au–Cl adlayers modulate the surface chemistry of gold nanoparticles during redox reactions

In the format provided by the authors and unedited

Table of Contents

List of Supplementary Notes

Supplementary Note 1. AuNP size distribution and nanogap uniformity 9
Supplementary Note 2. Surface chemistry of MLagg-CB[5] during EC- and Ch-ReSERS 11
Supplementary Note 3. XPS depth profiling of Au oxide 13
Supplementary Note 4. Au oxide growth from EC oxidation and O_2-PC 16
Supplementary Note 5. Surface transformations during EC-ReSERS 22
Supplementary Note 6. AuNP facets after ORCs 32
Supplementary Note 7. ORCs with other SERS substrates 34
Supplementary Note 8. EC-ReSERS Kinetics 37
Supplementary Note 9. EC-ReSERS in varying electrolytes 42
Supplementary Note 10. Electronic and interfacial properties of Au-Cl adlayer 53
Supplementary Note 11. XPS and SERS characterisation of Au-Cl 64
Supplementary Note 12. Au-Cl formation and reduction in EC-ReSERS 67
Supplementary Note 13. Au-Cl adlayer destabilisation with OH^- 72
Supplementary Note 14. Surface Transformations in Ch-ReSERS 75
Supplementary Note 15. Ch-ReSERS destabilisation with OH^- 87
Supplementary Note 16. Ch-ReSERS with citrate 91
Supplementary References 94

List of Supplementary Tables

Supplementary Table 1 | Au-O and Au-Cl vibrational frequency assignments from literature. 5
Supplementary Table 2 | Summary of Ch-ReSERS stages. 79

List of Supplementary Figures

Supplementary Figure 1 | Spectro-electrochemical cells for in situ SERS and DF measurements. 3
Supplementary Figure 2 | EC-ReSERS with different reference electrodes. 4
Supplementary Figure 3 | Raman and SERS of Au-Cl species 8
Supplementary Figure 4 | Characterisation of AuNP size distribution 10
Supplementary Figure 5 | SERS and XPS of MLagg-CB[5] undergoing Ch-ReSERS steps. 12
Supplementary Figure 6 | XPS depth profile of Au 4f before and after oxidation. 15
Supplementary Figure 7| Characterisation of Au oxide formation of MLagg-CB[5] from electrochemical (EC) potential step treatment. 18
Supplementary Figure 8 | Characterisation of Au oxide formation of MLagg-CB[5] from oxygen plasma cleaning (O_2-PC) treatment. 20
Supplementary Figure 9 | SERS spectra of MLagg-CB[5] after oxidation with O_2-PC and electrochemical potential step. 21
Supplementary Figure 10 | Different chemical transitions in SERS and DF of Au MLagg in an oxidation-reduction cycle. 25
Supplementary Figure 11 | Evolution of Au-Cl in other AuNP aggregate systems. 26
Supplementary Figure 12 | Progression of SERS and OCP after potential step oxidation and reduction of MLagg. 27
Supplementary Figure 13 | Potential step oxidation and reduction of MLagg-CB[5]. 28
Supplementary Figure 14 | Main vibrational modes of Au_2O_3 from DFT. (........ 29
Supplementary Figure 15 | Cyclic voltammetry (CV) and time-series SERS of MLagg-CB[5] in 100 µM CB[5] and 50 mM potassium phosphate buffer (pH 7.0) in H_2O or D_2O. 30
Supplementary Figure 16 | Raman of $AuCl_3$, CB[5] and CB[5]·$[AuCl_4]^-$. 31
Supplementary Figure 17 | MLagg-CB[5] morphology after multiple ORCs. 33
Supplementary Figure 18 | Cyclic voltammetry (CV) and time-series SERS of MLagg-NaCl in potassium phosphate buffer (KPB, pH 7.0) with and without CB[5]. 35

Supplementary Figure 19 | Cyclic voltammetry (CV) and time-series SERS of EC-roughened Au electrode in potassium phosphate buffer (KPB, pH 7.0) with and without CB[5]......36
Supplementary Figure 20 | EC-ReSERS with oxidative and reductive step potentials......40
Supplementary Figure 21 | EC-ReSERS kinetics at different buffer concentrations......41
Supplementary Figure 22 | Cyclic voltammetry (CV) and time-series SERS of MLagg-CB[5] in sodium phosphate buffer (pH 7.0) with and without CB[5]......44
Supplementary Figure 23 | Cyclic voltammetry (CV) and time-series SERS of MLagg-CB[5] in 100 mM Na_2SO_4 (pH 7.0) with and without CB[5]......45
Supplementary Figure 24 | Cyclic voltammetry (CV) and time-series SERS of MLagg-CB[5] in 100 mM $NaClO_4$ (pH 7.0) with and without CB[5]......46
Supplementary Figure 25 | Cyclic voltammetry (CV) and time-series SERS of MLagg-CB[5] in 100 mM H_3PO_4 (pH 1.0) with and without CB[5]......47
Supplementary Figure 26 | Cyclic voltammetry (CV) and time-series SERS of MLagg-CB[5] in 100 mM H_2SO_4 (pH 1.0) with and without CB[5]......48
Supplementary Figure 27 | Cyclic voltammetry (CV) and time-series SERS of MLagg-CB[5] in 100 mM $HClO_4$ (pH 1.0) with and without CB[5]......49
Supplementary Figure 28 | Cyclic voltammetry (CV) and time-series SERS of MLagg-CB[5] in 100 mM NaCl (pH 7.0) with and without CB[5]......50
Supplementary Figure 29 | Cyclic voltammetry (CV) and time-series SERS of MLagg-CB[5] in 100 mM HCl (pH 1.0) with and without CB[5]......51
Supplementary Figure 30 | Cyclic voltammetry (CV) and time-series SERS of MLagg-NaCl in 100 mM NaCl......52
Supplementary Figure 31 | Electrochemical control of Au-Cl and Au-OH- adlayer formation......57
Supplementary Figure 32 | CO adsorbed on MLagg......58
Supplementary Figure 33 | CO Stark shifts with Au-Cl adlayer stabilisation at OCP......59
Supplementary Figure 34 | Calculated Raman scattering spectrum of Au-Cl......60
Supplementary Figure 35 | Charge transfer on Cl and selected Au atoms......60
Supplementary Figure 36 | Calculated Raman scattering spectrum of Au-Cl configurations......61
Supplementary Figure 37 | Vibrational modes of Au-Cl with D = 2.24 Å (left) and D = 2.44 Å (right)......61
Supplementary Figure 38 | Formation of adlayers in different electrolytes......62
Supplementary Figure 39 | SERS spectra before and after adlayer formation......63
Supplementary Figure 40 | XPS depth profile of MLagg-CB[5] samples with Au-Cl......66
Supplementary Figure 41 | Formation and reduction of Au-Cl adlayer......70
Supplementary Figure 42 | Formation and reduction of Au-Cl adlayer during EC-ReSERS at varying scan rates. 71
Supplementary Figure 43 | Electrochemical formation of Au-Cl (in buffer) and its destabilisation with OH^-......73
Supplementary Figure 44 | Electrochemical formation of Au-Cl (in HCl) and its destabilisation with OH^-......74
Supplementary Figure 45 | Replicate time-series SERS and OCP measurements of MLagg-CB[5] during Ch-ReSERS with HCl and H_2SO_4......80
Supplementary Figure 46 | Replicate time-series SERS and OCP measurements of MLagg-CB[5] during Ch-ReSERS at pH 2.9 (0.5 M H_3PO_4/KH_2PO_4) with 0.1 M or 0.5 M NaCl......81
Supplementary Figure 47 | Replicate time-series SERS and OCP measurements of MLagg-CB[5] during Ch-ReSERS at pH 1.0 (0.5 M H_3PO_4) with 0.1 M or 0.5 M NaCl......82
Supplementary Figure 48 | Time-series low wavenumber SERS and OCP measurements of oxidised MLagg-CB[5] during CB[5]-HCl treatment.......83
Supplementary Figure 49 | Time-series SERS, DF, and OCP measurements of oxidised MLagg-CB[5] during CB[5]-HCl treatment......84
Supplementary Figure 50 | Time-series UV-Vis and OCP measurements of 1 mM CB[5] 0.5 M HCl solution during Ch-ReSERS. (......85
Supplementary Figure 51 | Ch-ReSERS of MLagg-CB[5] with CB[5] and reducing agents (ascorbic acid and $NaBH_4$). 86
Supplementary Figure 52 | Au-Cl destabilisation by OD^-......88
Supplementary Figure 53 | Au-Cl destabilisation by OH^- with oscillatory behaviour......89
Supplementary Figure 54 | SERS spectra of MLagg-CB[5] during different stages of Ch-ReSERS......90
Supplementary Figure 55 | CB[5] SERS spectrum correction for Au-Cl peak extraction......93

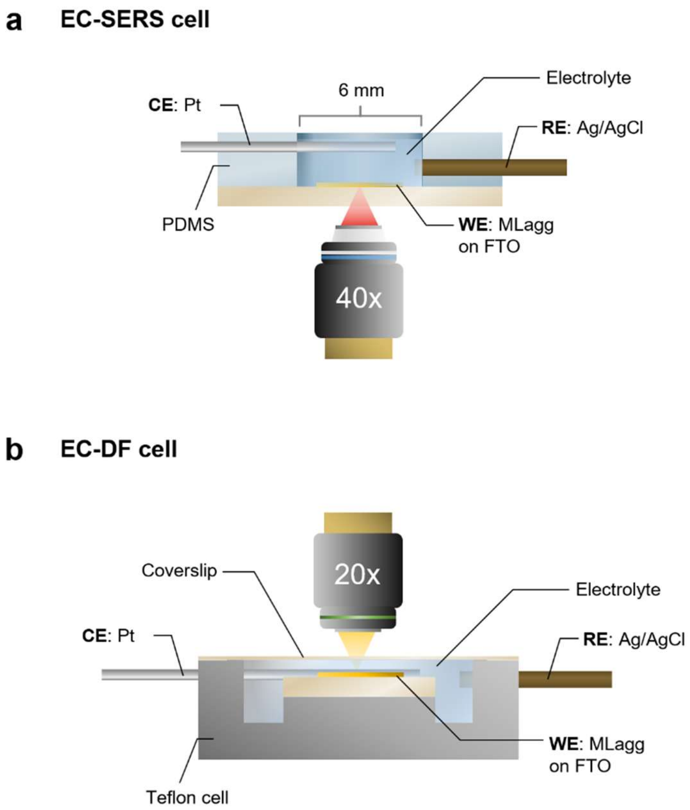

Supplementary Figure 1 | Spectro-electrochemical cells for in situ SERS and DF measurements. Cross-section diagrams of the spectro-electrochemical cells for (**a**) EC-SERS and (**b**) EC-DF measurements. CE = counter electrode, RE = reference electrode, and WE = working electrode.

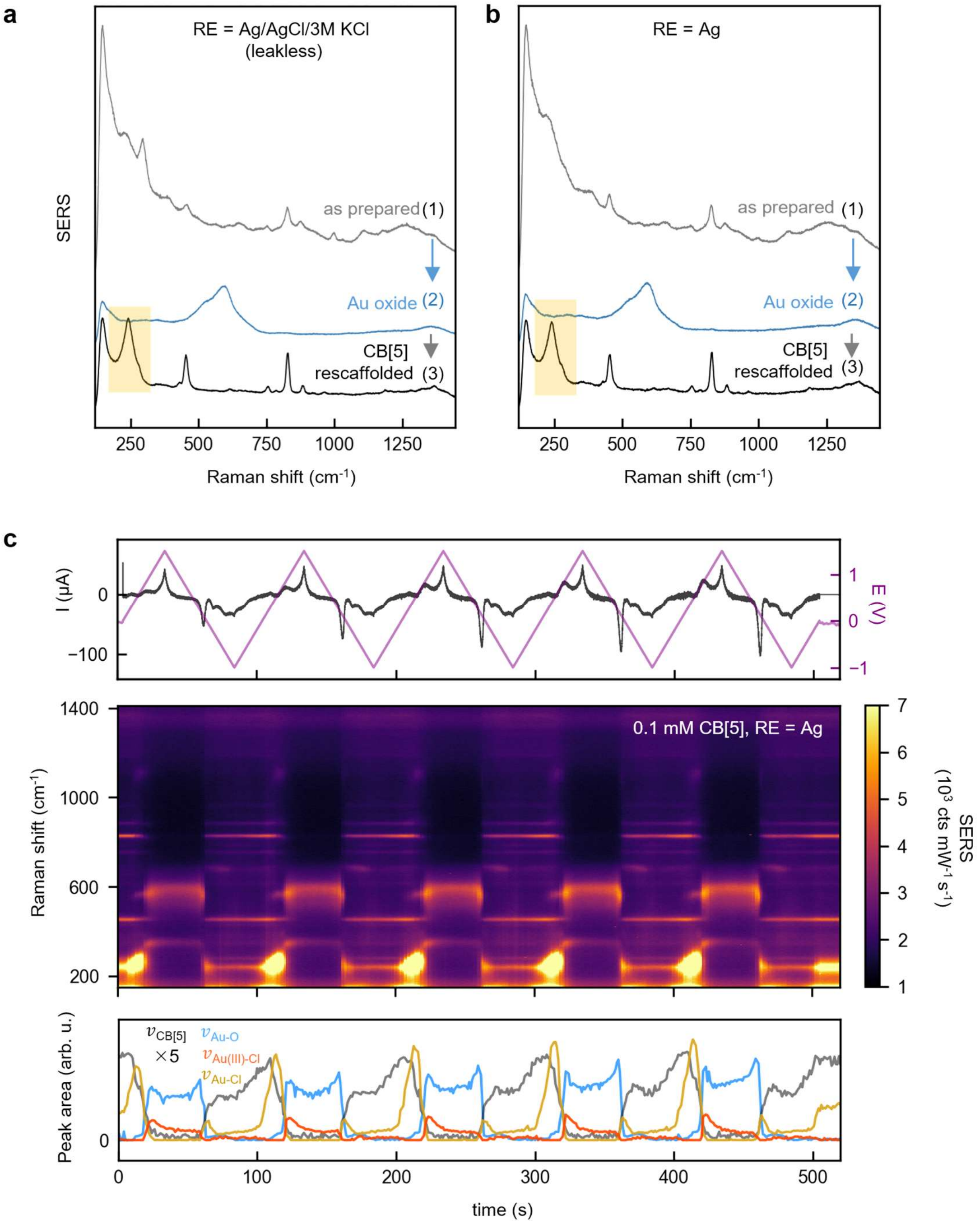

Supplementary Figure 2 | EC-ReSERS with different reference electrodes. SERS spectra of MLagg-CB[5] treated in an electrochemical cell with (**a**) a Ag/AgCl electrode, and (**b**) a Ag wire pseudo-reference. Spectra are taken (1) as initially prepared, (2) after electrochemical oxidation, and (3) after CB[5] re-scaffolding. Yellow region shows Au-Cl peak. (**c**) Continuous cycling with Ag wire pseudo reference electrode. The presence of significant Au-Cl and Au(III)-Cl SERS signal while using a Ag pseudo-reference electrode suggests most Cl- in system is introduced from the CB[5] solution, and not significantly from leakage from the Ag/AgCl reference electrode.

Supplementary Table 1 | Au-O and Au-Cl vibrational frequency assignments from literature. Calculated vibrational frequency assignments are in parentheses (). Vibrational modes from spectra also measured in D_2O, where available, are in brackets []. Where a broad peak is indicated, the authors refer to the peak position with maximum intensity (no peak fitting). For assignments indicated with an asterisk (*), assignment is tentative.

Peak position (cm^{-1})	Assignment	Notes	Refs.
173,<br>327, 350	$[AuCl_4]^-$,<br>δ_{ClAuCl} (bending modes),<br>$\nu_{Au\text{-}Cl}$ (stretching modes)	Raman of $KAuCl_{4(s)}$	1
173,<br>325, 347	$[AuCl_4]^-_{(aq)}$,<br>$\delta_{ClAuCl,}$<br>$\nu_{Au\text{-}Cl}$<br>(0.9-1.2V vs SCE)	SERS on electrochemically (EC)-roughened Au electrode in 0.1 M KCl,	2
178*<br>180*<br>185*	Au--oxyanions*<br>ClO_4^-<br>NO_3^-<br>SO_4^{2-}	SERS on EC-roughened Au electrode	3
235	$Au\text{-}Cl^-_{(ads)}$<br>(no applied potential)	SERS on EC-roughened Au electrode, 1 M KCl, adjusted to pH 1	1
245	$Au\text{-}Cl^-_{(ads)}$<br>(-0.4 V vs. SCE)	SERS on EC-roughened Au electrode, 0.1 M KCl	4
247	$Au\text{-}Cl^-_{(ads)}$	SERS with NaCl-aggregated citrate-stabilised AuNP	5
252	$Au\text{-}Cl^-_{(ads)}$	SERS with Klarite SERS substrate incubated with NaCl	5
(257)	Au_2Cl_2	Calculated	6
270	Au-Cl<br>(0.3 V)	SERS on gold electrode, 2 M HCl	7
275	Au-Cl<br>(0.5 V)	SERS on EC-roughened Au electrode, 0.1 M KCl	1,4
(269, 301)	$[Au(I)Cl_2]$-	Calculated	8
(285)	Au-Cl in<br>$[Au(I)Cl(OH)]^-$	Calculated	8
289	Au_2Cl_2 (ring stretch)	Raman of solid Au_2Cl_2	9
289<br>96, 168, 341,<br>367	AuCl	Raman of solid AuCl	10
(317, 322, 328,<br>317)	$[AuCl_2(OH)_2]$-	Calculated	8
324, 329, 334,<br>353	$[Et_4N][AuCl_2]$	Raman of solid	11
326, 329	$[Bu_4N][AuCl_2]$	Raman of solid	11
327	$[Bu_4N][AuCl_2]^-$	Raman of solid	1
323.5, 347.1	$[AuCl_4]^-_{(aq)}$	25 mM $[AuCl_4]$-, 2.5 M HCl	12
323, 348	$AuCl_4^-$ on Au,<br>(0.4 V to 1.0 V)	Au electrode in 2 M HCl	7
325, 348	$[AuCl_4]^-_{(aq)}$, pH < 3.5	0.02 M $HAuCl_4{\cdot}4H_2O$ solution, pH < 3	13
325, 335*, 348	Au(III)-Cl in<br>$[AuCl_3OH]^-_{(aq)}$, pH 3.5-5.5	0.02 M $HAuCl_4{\cdot}4H_2O$ solution, pH 3.5-5.5	13
(323, 353)	$[AuCl_4]^-$	Calculated	8
324, 341, 353	Au(III)-Cl in<br>$[AuCl_3OH]^-$	Calculated	8
328, 366, 379	Au_2Cl_2<br>(terminal $-AuCl_2$ stretch)	Raman of solid	9
335, 355	Au(III)-Cl stretch in<br>$[AuCl_2(OH)_2]^-$, pH 5.5-7	0.02 M $HAuCl_4{\cdot}4H_2O$ solution, pH 5.5-7	13

332*	$[AuCl_2]^-$	Formed from 25 mM $AuCl_4^-$, 2.5 M solution at high temperature and low dissolved O_2	12
335	$[AuCl_2]^-$ (~0.9 V)	SERS on electrochemically (EC)-roughened Au electrode in 0.1 M KCl,	2
357	Au(III)-Cl stretch in $[AuCl(OH)_3]^-_{(aq)}$, pH 7-11	0.02 M $HAuCl_4{\cdot}4H_2O$ solution, pH 7-11	13
360-420	Au-OH stretching, 0.3 V	0.1 M NaClO4 + NaOH (pH 9) - SHINERS on Au(111)	14
400-450	$Au\text{-}OH^-_{(ads)}$	SERS on EC-roughened Au electrode, 1 M KOH	15
425	$Au\text{-}OH^-_{(ads)}$, 0 V	SERS on EC-roughened Au electrode, 1 M KOH	16
470-475	$Au\text{-}OH^-_{(ads)}$ at high potentials	SERS on EC-roughened Au electrode in neutral (0.1 M Na_2SO_4) and basic conditions (0.1 M NaOH)	15
489-524	$AuO_{(ads)}$, 0.5 V	0.1 M NaClO4 + NaOH (pH 9) - SHINERS on Au(111) Further oxidation of $AuOH_{(ads)}$ to AuO	14
(510)	$Au\text{-}OH^-$ in $[Au(I)Cl(OH)]^-$	Calculated	8
510, 538	$Au\text{-}OH^-$ (1 V vs. Ag/AgCl)	Au electrode in 2 M HCl	7
530-560	$Au\text{-}OH^-$ in oxide layer (>1.35 V vs RHE)	SERS on EC-roughened Au electrode in acidic (0.1 M H_2SO_4), neutral (0.1 M Na_2SO_4) and basic conditions (0.1 M NaOH)	15
557-593	Au-O (gold oxide on surface) 0.7-1.4 V	SHINERS on Au(111), 0.1 M $NaClO_4$, pH 9	14
560	Au-OH (pH 14, 0.5 V)	SERS on EC-roughened Au electrode in 1 M KOH	16
560.4 [545.4 – D_2O]	Au-OH (stretching)	SERS on EC-roughened Au electrode in 0.1 M H_2SO_4 DFT	17
565, 579	Au(III)-OH in $[AuCl(OH)_3]^-_{(aq)}$, pH 7-11	0.02 M $HAuCl_4{\cdot}4H_2O$ solution, pH 7-11	13
566	Au(III)-OH in $[AuCl_3OH]^-_{(aq)}$, pH 3.5-5.5	0.02 M $HAuCl_4{\cdot}4H_2O$ solution, pH 3.5-5.5	13
568 (broad)	Au-O stretching of Au oxide (1.1 V)	SERS on EC-roughened Au electrode in 1 M $HClO_4$	18
568, 580	Au(III)-OH stretch in $[AuCl_2(OH)_2]^-$, pH 5.5-7	0.02 M $HAuCl_4{\cdot}4H_2O$ solution, pH 5.5-7	13
580	Au-O, surface oxide	From HREEL measurements on Au(111) at high oxygen surface coverage	19
(broad) 584 [562] 586 [564] 589 [567]	Au-O of Au oxide	SERS on EC-roughened Au electrode, 1.0 M $HClO_4$	20
590 (broad)	Au-O(H) stretching mode	SHINERS on Au(111) electrode, 1.0 M H_2SO_4	21
610	Au-OH- (1.5 V vs RHE)	SERS on EC-roughened Au electrode in neutral (0.1 M Na_2SO_4) and basic conditions (0.1 M NaOH)	15
612.6 [617.3]	O-Au-OH (oxide)	SERS on EC-roughened Au electrode in 0.1 M H_2SO_4 DFT	17
623, 614, 640, 617	Au-OH in $[AuCl_2(OH)_2]$-	Calculated	8
624	Au-OH in [AuCl3(OH)]-	Calculated	8
635	Au-OH- 1.1 V vs. RHE	SERS on EC-roughened Au electrode in neutral (0.1 M Na_2SO_4) and basic conditions (0.1 M NaOH)	15
635	Bulk phase $Au(OH)_3$	Prepared from precipitation of $Au(OH)_3$ from $KAuCl_4$ with an excess of OH-	16
635, 616, 593	$Au\text{-}OH^-$ in $[AuCl(OH)_3]^-$	Calculated	8

645	Au_2O_3	HREELS of Au oxide from oxygen DC reactive sputtering of Au(111) in UHV	22
650 (shoulder)	Au-O from Au oxide	SERS on EC-roughened Au electrode in 1.0 M $HClO_4$	20
(674.9)	AuO, with O on top of a single adatom	Calculated	23
675	AuO (gaseous)	IR of AuO prepared in rare gas matrix at low temperature	24
676.6	O-Au-O stretch of $Au(OH)_2$	IR of $Au(OH)_2$ molecules prepared from laser-ablated gold atoms with H_2O_2 and H_2+O_2 mixtures	25
751.7 [749.7]	O-Au-O*	SERS on EC-roughened Au electrode in 0.1 M H_2SO_4, No close matches with DFT	17
771.4 [783.7]	O-Au-O*	SERS on EC-roughened Au electrode in 0.1 M H_2SO_4, No close matches with DFT	17
788	Au-OH bending mode (0.5-0.9 V Ag/AgCl)	SHINERS on Au(111) in 0.1 M $NaClO_4$ (pH 9)	14
790	Au-OH bending mode of OH^- adsorption on top sites (0.4-0.5 V Ag/AgCl)	SHINERS on Au(111) in 0.1 M $NaClO_4$ (pH 9)	14
790	O-O in Alpha-oxide (Au-OOH)	SERS on EC-roughened Au electrode in acidic (0.1 M H_2SO_4) and neutral (0.1 M Na_2SO_4)	15
807	Au-OH bending mode	SHINERS on Au(111) electrode, 1.0 M Na_2SO_4	21
810	Au-OOH (Au with low coordination number), > 1.4 V vs RHE	SERS on EC-roughened Au electrode in 1.0 M $HClO_4$, DFT	20
822	O-O in Au-OOH (1.2-1.4 V vs)	SERS on EC-roughened Au electrode in 1.0 M $HClO_4$	18

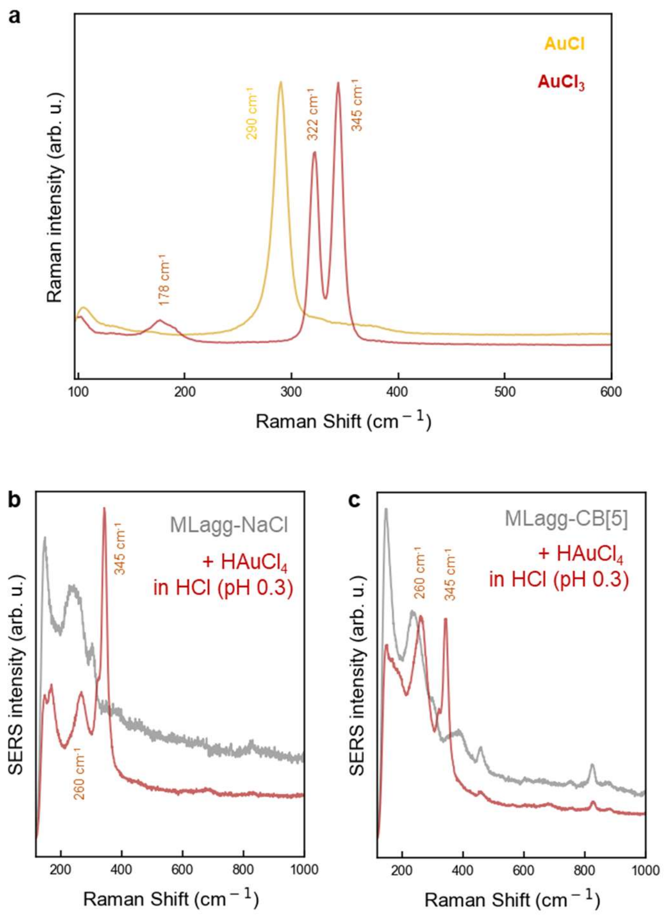

Supplementary Figure 3 | Raman and SERS of Au-Cl species. (**a**) Powder Raman of $AuCl_{(s)}$ (yellow line) and $AuCl_{3(s)}$ (red line) collected at 20 s integration time, 785 nm excitation laser with 12.4 mW power. Solid $AuCl_3$ exists as Au_2Cl_6 molecular dimers, whereas AuCl consists of molecular Au-Cl-Au-Cl chains. (**b**) SERS spectra of MLagg-NaCl, as prepared (grey line) and after incubation in 40 mM $HAuCl_4$ in 1 M HCl (red line). (**c**) SERS spectra of MLagg-CB[5] (pre-cleaned and CB[5]-rescaffolded, grey line) and after incubation in 40 mM $HAuCl_4$ in 1 M HCl (red line). Au(III)-Cl and Au-Cl peaks are labelled. SERS spectra were collected with 1 s integration time and with 785 nm excitation laser with 1 mW power.

Supplementary Note 1. AuNP size distribution and nanogap uniformity

This section addresses the size and shape variation of the AuNPs used in the MLagg-CB[5] substrate and its impact on SERS performance. While the AuNPs exhibit moderate variation in diameter and morphology, this does not significantly affect signal reproducibility. Instead, the key factor governing reproducibility is the uniform nanogap spacing established by the CB[5] molecular scaffold.

The AuNPs used in this study are commercially sourced (BBI), which are size-controlled to ensure batch-to-batch consistency (with any variation in ligands removed using our cleaning protocol). The AuNP size distribution measured by dynamic light scattering (DLS, **Supplementary Figure 4a**) shows an average diameter of 82 nm with polydispersity index (PDI) of 0.1, indicating a relatively narrow size distribution[26]. Additional SEMs over a larger number of AuNPs better represent the size and shape distribution, as expected in practical colloidal synthesis (**Supplementary Figure 4b**).

Despite the modest variation in AuNP size and shape, the MLagg-CB[5] substrates demonstrate excellent signal reproducibility (<5% RSD local signal uniformity and <5% RSD substrate-to-substrate reproducibility[27]). This reproducibility originates from the controlled nanogap spacing, as the local electric field enhancement that drives SERS is known to be exponentially sensitive to gap width but only weakly dependent on AuNP diameter.[28] Previous finite-difference time-domain (FDTD) simulations confirm that atomic-scale variations in gap width have a greater effect on SERS intensity than 10 nm changes in AuNP size[28]. This underscores that gap precision, not AuNP size uniformity, is the key parameter for achieving reproducible enhancement. In our system, the nanogap spacing is defined primarily by the CB[5] scaffolds, which bind between adjacent Au facets and set a consistent spacing of approximately 0.9 nm[29]. Thus, even when AuNPs differ in size or facet area, the CB[5] molecules enforce uniform nanogaps between neighbouring particles. This separation is governed by the molecular geometry of CB[5], as established in previous work, where CB[5]-mediated AuNP assemblies consistently formed plasmonic junctions with well-defined 0.9 nm spacings, regardless of the particle diameter[29].

The reproducibility resulting from the uniform nanogap spacing was further demonstrated experimentally in previous work[28,30], where the same batch of AuNPs was used to form aggregates using either CB[5] to establish uniform gap spacings or using salt, which results in non-uniform gap spacings. The CB[5]-aggregated AuNPs showed far superior reproducibility compared to salt-aggregated controls demonstrating that controlling interparticle spacing with CB[5] scaffolds effectively eliminates one of the major sources of variability in SERS substrates.[28] Moreover, compared to CB[5]-aggregated AuNPs, salt-aggregated AuNPs exhibit much broader plasmonic modes due to the inhomogeneous distribution of gap sizes[30]. This further underscores the critical role of gap uniformity in achieving reproducible and high-performance SERS.

It is also important to emphasize that mild structural disorder in the arrangement of AuNPs can actually enhance SERS performance, provided that the interparticle gap spacing remains uniform[30]. Prior work using the same MLagg-CB[5] system has shown that its disordered architecture support more efficient light coupling into the nanogaps. In contrast, perfectly ordered superlattices may suffer from symmetry-imposed constraints that suppress field confinement in the nanogaps.

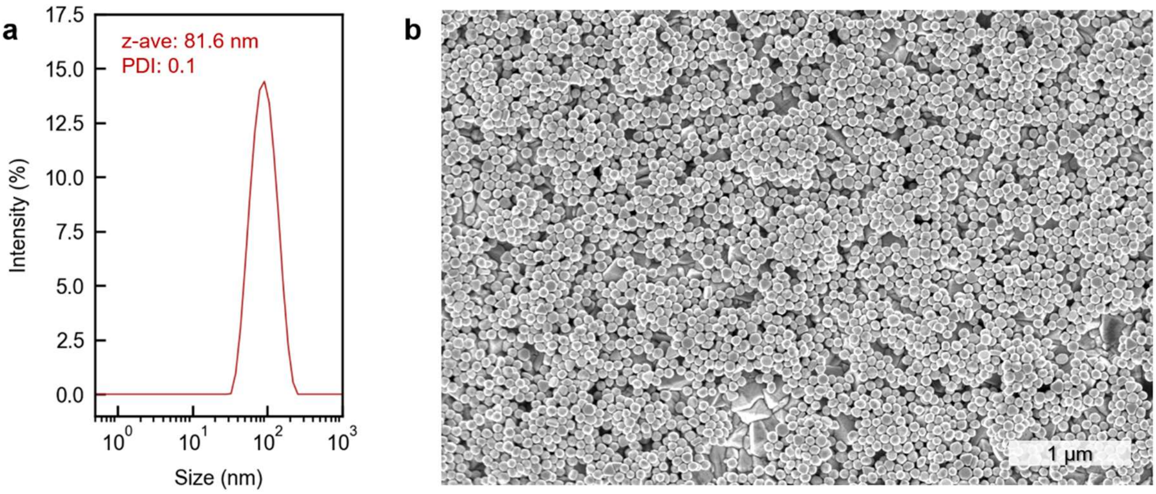

Supplementary Figure 4 | **Characterisation of AuNP size distribution.** (**a**) Dynamic light scattering (DLS) size distribution. (**b**) Scanning electron microscope (SEM) image of aggregated 80 nm AuNPs deposited on a fluorine tin oxide (FTO)-coated glass substrate.

Supplementary Note 2. Surface chemistry of MLagg-CB[5] during EC- and Ch-ReSERS

When the MLagg-CB[5] undergoes the ReSERS protocol, its surface chemistry undergoes several transformations, which can be probed with SERS and XPS. **Extended Data Figure 1** and **Supplementary Figure 5** show the SERS and corresponding XPS spectra of the MLagg-CB[5] after undergoing each step of the EC- and Ch-ReSERS protocols. Initially, when the MLagg-CB[5] is prepared, the AuNPs are coated with native surfactants as well as the added CB[5] aggregating agent. The SERS spectrum shows characteristic CB[5] peaks at 826 cm^{-1} and 450 cm^{-1}, and a relatively high background signal corresponding to other native ligands. The O 1s, N 1s, and C 1s XPS spectra also show peaks consistent with the presence of CB[5] molecules (532 eV component in O 1s and 289 eV component in C 1s corresponding to C=O, and N 1s and C 1s peaks corresponding to the C-N bonds from the glycoluril units).

<u>*EC-ReSERS*</u>
In the EC-ReSERS protocol (**Extended Data Figure 1**), the MLagg is electrochemically oxidised in neutral buffer conditions, resulting in the oxidation and/or desorption of organic ligands and the formation of Au oxide. The SERS spectrum is now dominated by the broad Au-O vibrations corresponding to the formation of an Au oxide layer in the nanogaps. Consistent with the SERS spectrum, a new component in the Au 4f XPS spectrum appears at ~85-86 eV and 88.7-89.7, corresponding to Au(III) $4f_{7/2}$ and $4f_{5/2}$ from Au oxide[31–33]. The O 1s spectrum also features three oxygen components consistent with Au oxide: components at 532 eV, 531 eV, and 529 eV signify the presence of adsorbed H_2O, OH^-, and O^{2-}, respectively[32,33]. Although the SERS spectrum shows only features associated with Au oxide, the N 1s and C 1s XPS spectra still contain peaks associated the CB[5] scaffolds, suggesting there are still CB[5] molecules in the system. During electrochemical oxidation, the CB[5] molecules are likely only desorbed and/or partially oxidised. Since oxidation is rapid, some CB[5] molecules can remain close to the surface and can get trapped within or can re-adsorb onto the oxide surface (see schematic in **Extended Data Figure 1a**). The change in environment of the residual CB[5] molecules is reflected in the N 1s and C 1 s spectra which feature peaks shifted to lower binding energies.

Subsequent reduction of the Au oxide layer in the presence of an excess of CB[5] scaffold leads to the regeneration of the nanogap hotspots. The SERS and XPS spectra now show loss of the Au oxide features, and the return of peaks associated with CB[5]. The SERS spectrum of a control in which CB[5] is not added during the reduction step shows a decrease in the characteristic CB[5] peaks and the appearance of small features associated with adsorbed oxygen species. The XPS N 1s spectrum still suggests some residual nitrogen-containing species in the system, although the C 1s spectrum does not match the spectral features associated with CB[5].

<u>*Ch-ReSERS*</u>
The surface chemistry of the MLagg undergoing the Ch-ReSERS protocol was also tracked with SERS and XPS (**Supplementary Figure 5**). Here, the MLagg is treated with O_2-PC, after which the SERS spectrum is again dominated by the broad Au-O band, and the XPS spectra of Au 4f and O 1s similarly show peaks characteristic of the formation of Au oxide. In contrast to the results from electrochemical oxidation, the N 1s spectrum exhibits a much weaker signal, suggesting most of the CB[5] is now removed from the MLagg surface. This is consistent with the mechanism of O_2-PC cleaning, where organic molecules are oxidised to volatile compounds such as CO_2, which are subsequently withdrawn from the plasma oven via vacuum.

The oxidised MLagg can then be regenerated via incubation with CB[5] in HCl. This treatment results in the loss of the Au oxide, and the reintroduction of CB[5], as confirmed from both SERS and XPS spectra. Treatment of the oxidised MLagg with HCl only similarly results in loss of Au oxide with no trace of CB[5], as confirmed from the weak N 1s spectrum. Peaks appearing in the XPS C 2p spectra appear to be from residual carbonaceous contaminants, while peaks in the O 1s spectrum can be attributed to residual bound oxygen species remaining after Au oxide removal, consistent with previous observations[34]. Further discussion of the Cl 2p XPS spectra is detailed in **Supplementary Note 11. XPS and SERS characterisation of Au-Cl.**

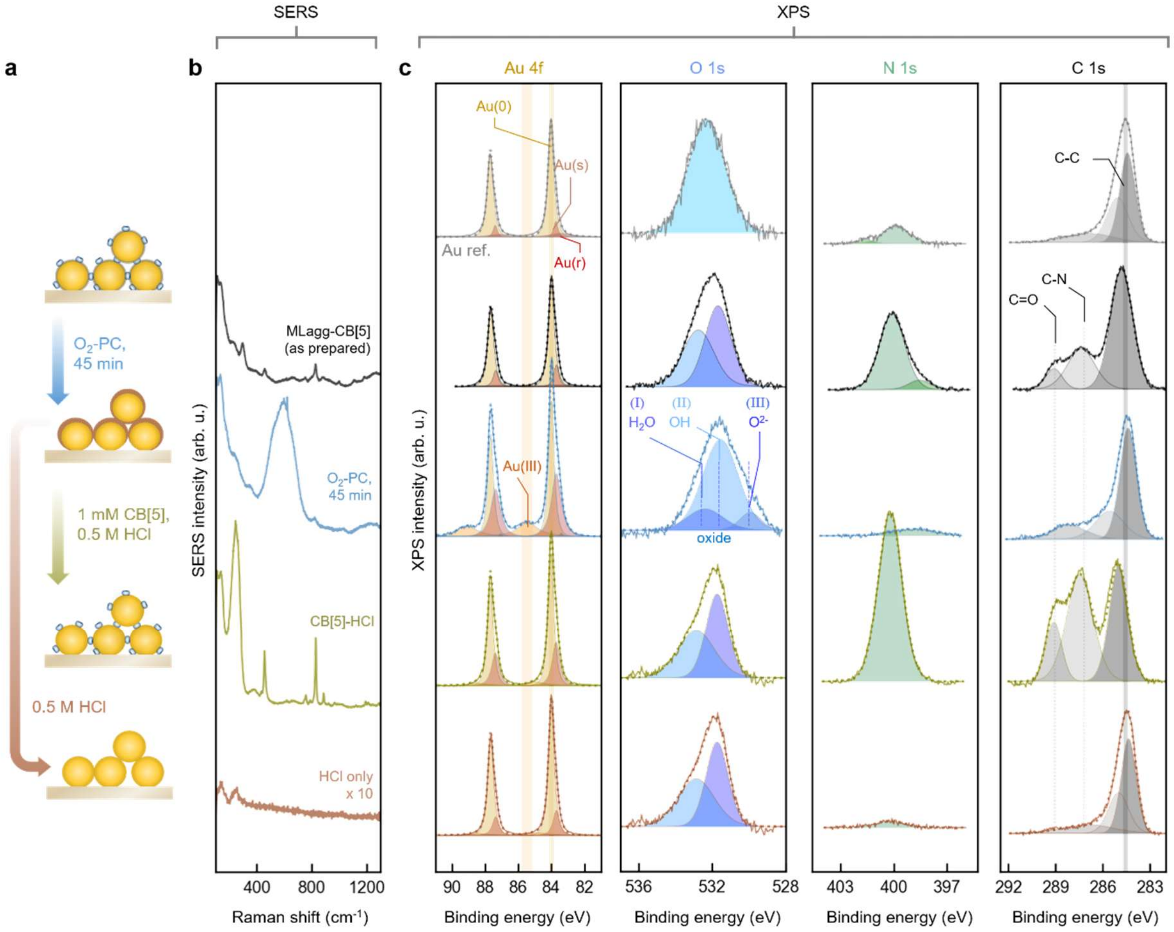

Supplementary Figure 5 | SERS and XPS of MLagg-CB[5] during Ch-ReSERS. (**a**) Schematic of the Ch-ReSERS protocol, where MLagg-CB[5], with CB[5] and other ligands such as citrate on the AuNP surface, is oxidised by oxygen plasma cleaning (O_2-PC, blue arrow), followed by regeneration with CB[5] and HCl (green arrow). A control is shown where the oxidised MLagg-CB[5] is treated with HCl only (brown arrow). (**b**) SERS spectra of MLagg-CB[5] as-prepared (black), after 45 min O_2-PC (blue), and after regeneration with CB[5] and HCl (green) or without CB[5] (brown). (**c**) Au 4f, O 1s, N 1s, and C 1s XPS spectra (solid lines), peak fits (dotted lines), and fitted components of a planar Au reference (grey), compared with MLagg-CB[5] as-prepared (black), after O_2-PC (blue), and after regeneration with CB[5] (green) or without CB[5] (brown). All XPS spectra were collected at 700 eV and normalised to maximum intensity. The Au reference also shows O 1s, N 1s, and C 1s signals from surface contaminants.

Supplementary Note 3. XPS depth profiling of Au oxide

In the ReSERS protocol, the surface of the AuNPs can be cleaned after exposure to oxidative conditions such as oxygen plasma cleaning (O_2-PC) or electrochemical (EC) oxidation. These treatments result in the oxidation and/or desorption of molecular ligands with the concomitant formation of an oxide layer[27,35]. The Au oxide layer can be characterised with X-ray photoelectron spectroscopy (XPS), a surface-sensitive technique that probes the chemical composition of the top-most <10 nm surface of a sample.

Depth profiles of the Au oxide layers formed from O_2-PC and EC oxidation on both planar Au (evaporated Au on silicon) and a MLagg-CB[5] were obtained using increasing X-ray photon energies (200, 450, 700, 1100 eV), corresponding to decreasing surface sensitivities (**Supplementary Figure 6**). Before oxidation, the Au 4f spectra (**Supplementary Figure 6a,d**) show the characteristic metallic Au(0) $4f_{7/2}$ and $4f_{5/2}$ peaks at 84.0 eV and 87.7 eV, along with a surface-component, Au(s), shifted ~0.3 eV towards lower binding energy (BE)[31]. Another component, Au(r), shifted ~0.6 eV towards lower BE, corresponds to low-coordinated Au atoms on the rough Au surface[31]. After oxidation, a new component appears at 85-86 eV and 88.7-89.7, corresponding to the Au(III) $4f_{7/2}$ and $4f_{5/2}$ from Au oxide [31–33]. The Au(III) component and the other surface-related features, Au(s) and Au(r), are more prominent in the spectra obtained from the lower photon energies, as a greater fraction of the emitted photoelectrons originate from the first few atomic layers of the sample[31].

The depth-dependent Au 4f spectra were used to estimate the thicknesses of the Au oxide layers. Assuming a planar semi-infinite substrate (Au) with a uniform overlayer (Au oxide) with thickness d_{AuOx}, the ratio of the intensities from Au(III) and Au(0) is given by

$$\frac{I_{AuOx}}{I_{Au}} = \frac{F_{AuOx}\rho_{AuOx}\sigma_{AuOx}\lambda_{AuOx}\left(1 - \exp\frac{-d_{AuOx}}{\lambda_{AuOx}\cos\varphi}\right)}{F_{Au}\rho_{Au}\sigma_{Au}\lambda_{AuOx|Au}\left(\exp\frac{-d_{AuOx}}{\lambda_{AuOx|Au}\cos\varphi}\right)} \quad (1)$$

where I_{AuOx} and I_{Au} are the intensities of the Au(III) $4f_{7/2}$ and Au(0) $4f_{7/2}$ peaks, F includes various instrumental and experimental parameters, ρ is the atomic density of the analysed species, σ is the differential cross-section for a given shell of an analysed atom, λ_{AuOx} is the inelastic mean free path (IMFP) of electrons through Au oxide, $\lambda_{AuOx|Au}$ is the IMFP for electrons through the Au substrate and the Au oxide overlayer, and φ is the emission angle with respect to the surface normal. This model can apply to various scenarios, including a planar metal. or metal oxide surface[31,36,37]. For this system, $F_{AuOx} = F_{Au}$, $\sigma_{AuOx} = \sigma_{Au}$, $\rho_{AuOx} = 11.34$ g·cm^{-3}, $\rho_{Au} = 19.32$ g·cm^{-3}, and $\varphi = 0°$. It was also assumed that $\lambda_{AuOx} = \lambda_{AuOx|Au}$, which is a reasonable assumption for overlayers on a substrate[38]. As IMFP is dependent on energy, λ_{AuOx} was estimated from the NIST database[39], yielding λ_{AuOx} = 0.55 nm, 0.87 nm, 1.17 nm, and 1.62 nm for the 200, 450, 700, and 1100 eV photon energies, respectively.

To estimate the parameter d_{AuOx} for the planar Au samples after undergoing O_2-PC and EC oxidation treatments, the Au(III):Au(0) intensity ratios as a function of IMFP (λ_{AuOx}) was fitted to the model[31] described by eqn 1 (**Supplementary Figure 6b,c**), yielding d_{AuOx} = 0.89 ± 0.04 nm (R^2 = 0.970) for the Au oxide from EC oxidation (potentiostatic oxidation at +1.5 V for 30 s in 50 mM potassium phosphate buffer, pH 7.0), and d_{AuOx} = 0.92 ± 0.04 nm (R^2=0.942) for the Au oxide from O_2-PC (45 min). Previous work[40] studying Au oxides grown potentiostatically on planar Au in acidic conditions estimates from ellipsometric and electrochemical data that an oxidised atomic monolayer (ML) of Au (forming a place-exchanged Au^{3+}– O^{2-}/ Au^{3+}– O^{2-} layer) is 0.37-0.4 nm. This suggests that the oxide layer is approximately 2 ML of oxidised Au.

The model described was also applied to the oxidised MLagg-CB[5] samples. Fitting of the model described by eqn (1) yielded moderately reasonable fits, with d_{AuOx} = 0.73 ± 0.04 nm (R^2 = 0.899) for the Au oxide from EC oxidation and d_{AuOx} = 0.65 ± 0.04 nm (R^2=0.925) for the Au oxide from O_2-PC. These thicknesses correspond to 1-2 ML of oxidised Au. However, it is noted that the model assumptions, including a flat surface and uniform overlayer, are not fully applicable to the MLagg-CB[5]. The presence of the surfactants on the AuNP surfaces prior to oxidation as well as the nanogap morphology can result in non-uniform oxidation over the surface of the MLagg-CB[5]. The XPS signal intensity can also be affected by shadowing effects from the curvature of the

nanoparticles[36,37], and signal attenuation in the nanogaps. Most of the signal intensity may be preferentially weighed towards the top areas of the MLagg.

To account for the spherical morphology of nanoparticles, we applied the model developed by Wu *et al.* to estimate the thickness of a uniform oxide layer on a nanoparticle core[41,42], given by eqn 2:

$$\frac{I_{AuOx}}{I_{Au}} = \frac{F_{AuOx}\rho_{AuOx}\sigma_{AuOx}\lambda_{AuOx}\int_{R_1}^{R_2}\int_0^{\pi}\exp\left(\frac{r\cos\theta - \sqrt{R_2 - r^2\sin^2\theta}}{\lambda_{AuOx}}\right)r^2\sin\theta\, d\theta dr}{F_{Au}\rho_{Au}\sigma_{Au}\lambda_{AuOx|Au}\int_0^{R_1}\int_0^{\pi}\exp\left(\frac{r\cos\theta - \sqrt{R_2 - r^2\sin^2\theta}}{\lambda_{AuOx|Au}}\right)r^2\sin\theta\, d\theta dr} \quad \textbf{(2)}$$

This model assumes spherical nanoparticle cores of radius R_1. The total radius of the nanoparticle with a uniform oxide layer is R_2, thus $d_{AuOx} = R_2 - R_1$. As with the assumptions used for the model described by eqn. 1, $F_{AuOx} = F_{Au}$, $\sigma_{AuOx} = \sigma_{Au}$, $\rho_{AuOx} = 11.34$ g·cm^{-3}, $\rho_{Au} = 19.32$ g·cm^{-3}, and $\lambda_{AuOx} = \lambda_{AuOx|Au}$. R_1 was simplified to 40 nm. The intensity ratios of Au(III) and Au(0) were fitted as a function of the energy-dependent IMFP by numerically integrating the model over polar coordinates r and θ to estimate the parameter R_2. This yielded d_{AuOx} = 0.46 ± 0.04 nm (R^2 = 0.901) for the Au oxide from EC oxidation and d_{AuOx} = 0.40 ± 0.04 nm (R^2=0.887) for the Au oxide from O_2-PC, suggesting an oxide thickness closer to 1ML. However, as the model assumes uniform oxide thickness around the AuNP and does not account for increased attenuation in the nanogap regions, these calculated thicknesses are likely underestimated. A best estimate from XPS for the thickness is thus ~2 ML of oxidised Au.

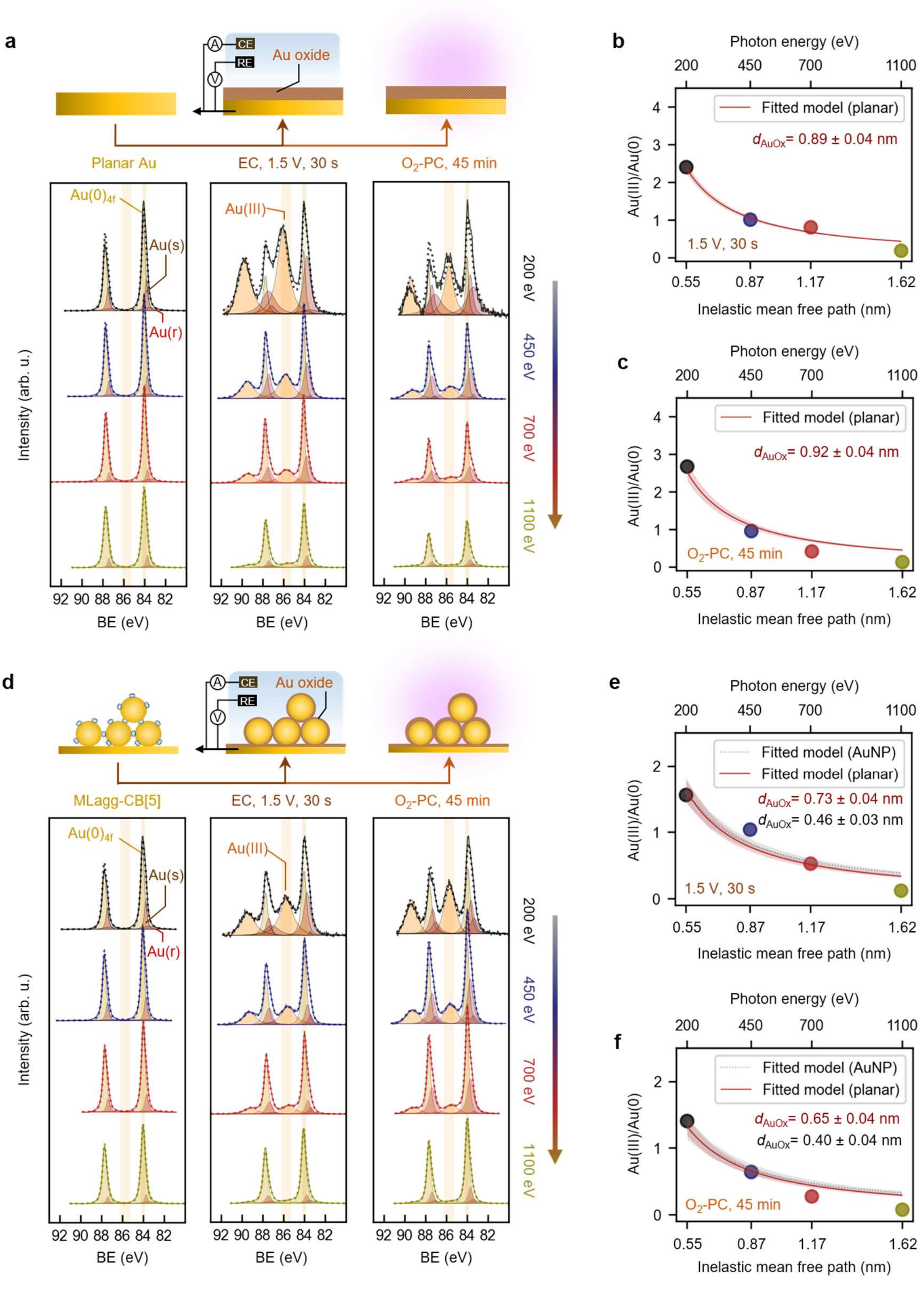

Supplementary Figure 6 | XPS depth profile of Au 4f before and after oxidation. (**a**) Au 4f photoelectron spectra (solid lines) and peak fits (dotted) from planar Au before and after the formation of a Au oxide layer from electrochemical (EC) oxidation or oxygen plasma cleaning (O_2-PC) treatments. Spectra were measured at 200 eV (black), 450 eV (dark blue), 700 eV (red) and 1100 eV (green). Peak area ratios of Au(III) $4f_{7/2}$ and Au(0) $4f_{7/2}$ *vs* inelastic mean free path (IMFP) for planar Au after (**b**) electrochemical oxidation and (**c**) O_2-PC. (**d**) Corresponding Au 4f spectra of MLagg-CB[5]. (**e,f**) Peak area ratios versus IMFP for MLagg-CB[5] after EC oxidation (**e**) and O_2-PC (**f**). Data were fitted using models of a complete oxide overlayer on planar Au (red) or a uniform oxide shell on spherical AuNPs (grey). Lines represent the best-fit curve with fitted Au oxide thickness, $d_{AuOx,}$ while the shaded region corresponds to the models recalculated with $d = d_{AuOx} \pm d_{error}$, where d_{error} is the 1σ standard error from the regression, representing uncertainty of the thickness estimate.

Supplementary Note 4. Au oxide growth from EC oxidation and O_2-PC

The presence of ligands and the close-packed arrangement of the nanoparticles of the MLagg-CB[5] can result in non-uniform Au oxide growth rates over the MLagg surface. In O_2-PC, for instance, bombardment of the sample with reactive oxygen species (ROS) can lead the preferential oxidation of the more exposed top regions of the MLagg compared to the nanogaps. In potential-step electrochemical oxidation, on the other hand, variations in potentials and local concentrations along the surface of the MLagg can similarly result in non-uniform oxide growth along the surface.

To track the Au oxide growth with time, the MLagg was characterised after treatment with various O_2-PC exposure times (**Supplementary Figure 8a**), and oxidising potential step hold times (**Supplementary Figure 7a**). After an oxide was formed from each treatment, the oxidised MLagg was characterised with SERS and DF spectroscopy, while representative samples were further characterised with XPS depth profiling at various X-ray energies to estimate Au oxide thickness. Electrochemical characterisation was also used as a complementary approach to estimate the amount of Au oxide from the charge required for reduction. From previous coulometric studies of Au oxide on planar Au[43,44], it was determined that the reduction of a monolayer of Au oxide requires 400 $\mu C\ cm^{-2}$. However, since the MLagg Au surface area is larger than its geometric surface area, each surface area of each MLagg was estimated using cyclic voltammetry (CV)[45]. After the reduction of the Au oxide formed from oxidation treatment, the potential was ramped to +1.5 V to form a monolayer of Au oxide on the MLagg; this was followed by a cathodic sweep back to 0.0 V to reduce the monolayer oxide. The CV cycle was repeated until a constant voltammogram was obtained. The charge required to reduce the monolayer oxide is proportional to the surface area of the MLagg and was therefore used to normalise the charge from the initial Au oxide reduction.

Au oxide from O_2-PC

Characterisation the MLagg after various O_2-PC exposure times suggests that the Au oxide layer grows logarithmically. **Supplementary Figure 8b** shows representative linear sweep voltammogram for the reduction of the Au oxide formed from increasing O_2-PC treatment times, with a representative monolayer Au oxide cyclic voltammogram for reference. The amount of Au oxide increases with increasing exposure time, growing beyond an equivalent monolayer by 5 min exposure time (**Supplementary Figure 8c**). Growth continues until a plateau is reached by 15 min, where an equivalent of ~1.5 ML Au oxide is formed (note that the Au oxide may not be uniformly distributed across the surface area, thus the actual thickness of the Au oxide layer may be greater than 1.5 ML). Notably, the Au oxide reduction potential (E_{red}) also shifts to less positive values (compared to the E_{red} for monolayer Au oxide reduction) with increasing O_2-PC time, consistent with the formation of a thicker oxide [43]. Au oxide thickness estimates from XPS depth profiling (**Supplementary Figure 8d,e**) also show similar Au oxide growth kinetics.

Tracking the SERS spectra of the oxidised MLagg, on the other hand, suggests slower Au oxide growth at the nanogaps (**Supplementary Figure 8f,g**). As the highest SERS enhancement is primarily concentrated at the nanogaps, a SERS spectrum reflects the chemical environment at the nanogaps. O_2-PC for 30 s shows an immediate decrease in the CB[5] peak but minimal increase in the Au-O peaks. By 5 min O2-PC, there is a sharper decrease in CB[5] peak and the appearance of a broad Au-O band centred at 590 cm^{-1}. Notably, 5 min O_2-PC also corresponds to more than an equivalent 1 ML of Au oxide on the MLagg. The SERS spectra, however, suggests that the nanogaps are only starting to oxidise. These results suggest non-uniform growth of an Au oxide layer, with >1 ML oxide already growing outside the nanogaps at 5 min O_2-PC. At 15 min O_2-PC, there is sharp increase in the Au-O band, followed by a plateau; by this point, the MLagg has an equivalent of ~1.5 ML of Au oxide.

The DF scattering spectra of the MLagg before and after oxidation was also tracked with time (**Supplementary Figure 8h,i**). As the DF scattering spectrum is sensitive to changes in the refractive index around the nanoparticles, changes in the spectrum can suggest the presence of Au oxide even outside the nanogap. With increasing O_2-PC time, the MLagg chain mode exhibits increasing red-shifts and decreasing scattering intensity. The red shift in chain mode peak wavelength[35] implies the growth of the Au oxide layer (characterised by high refractive index) on the surface of the nanoparticles with similar kinetics suggested by the electrochemical data.

Au oxide from EChem oxidation

The effect of hold times on the potential step oxidation (+1.5 V, in potassium phosphate buffer, pH 7.0) of the MLagg was investigated using the same characterisation methods (**Supplementary Figure 7a**). Potential steps to +1.5 V show the rapid oxidation of the MLagg with an equivalent of ~1 ML of oxidised Au formed by 15 s. Continued oxidation to 30 s and 60 s shows a minor increase in oxide, with a plateau of ~1.1 ML Au oxide (**Supplementary Figure 7b,c**). Au oxide thickness estimates from XPS depth profiling also yield roughly similar trends (**Supplementary Figure 7d,e**). The reduction potentials of the oxides are shifted 220 mV to less positive values compared to the monolayer Au oxide reduction peak, suggesting a more stable oxide formed from potentiostatic oxidation. Additional XPS analysis of the oxidised MLagg suggests that there are residual CB[5] ligands remaining on the MLagg (**Extended Data Figure 1**), possibly re-adsorbed onto the surface of the Au oxide or in non-oxidised crevices. This indicates non-uniform oxides greater than 1 ML are formed on the surface of the MLagg and/or residual adsorbed CB[5] molecules have a stabilising effect on the Au oxide layer. The prominence of the Au-O band in the SERS spectra, however, implies that the nanogaps are already being oxidised at this point, suggesting a relatively more uniform oxidation of the MLagg surface compared to O_2-PC.

The estimation of oxide layer thickness is consistent with literature reports showing that Au oxide growth is generally self-limiting under aqueous electrochemical conditions, typically logarithmically growing up to 2 ML of oxide, although thicker oxides beyond 3 ML can form under prolonged polarisation (>100 s) at highly oxidising potentials (>1.7 V)[46,47]. In this context, our oxide thickness estimates of ~1.1 ML after 60 s at +1.5 V, supported by both electrochemical and XPS data, fall well within the expected range.

The DF spectra before and after electrochemical oxidation (both spectra measured in air), show minimal changes in the chain mode amplitude (<2% decrease) and peak position (< 4 nm blue shift) (**h,i**). These results contrast with the DF spectra from MLaggs oxidised with O_2-PC, which exhibited a 16 nm red shift and 10% decrease in scattering intensity with 30 s O_2-PC treatment. The differences in DF spectra suggest that the two different oxidising treatments produce oxides with different properties, such as in uniformity, thickness, and structure. Specifically, electrochemical oxidation forms relatively more uniform and thinner oxide layers.

Comparing the SERS spectra also reveal chemical differences. **Supplementary Figure 9** presents the SERS spectra of MLagg samples oxidised with 45 min O_2-PC and 60 s of potential step electrochemical oxidation (**Supplementary Note 5**). Both oxides feature similar components (520, 590, and 660 cm^{-1} peaks), but at different ratios (520/590/660 peak ratio of 0.30 : 0.43 : 0.27 for O_2-PC Au oxide versus 0.30 : 0.60: 0.11 for the electrochemically oxidised MLagg). The MLagg treated with O_2-PC also features a higher background signal, possibly from increased roughness or defects generated from the plasma cleaning process.

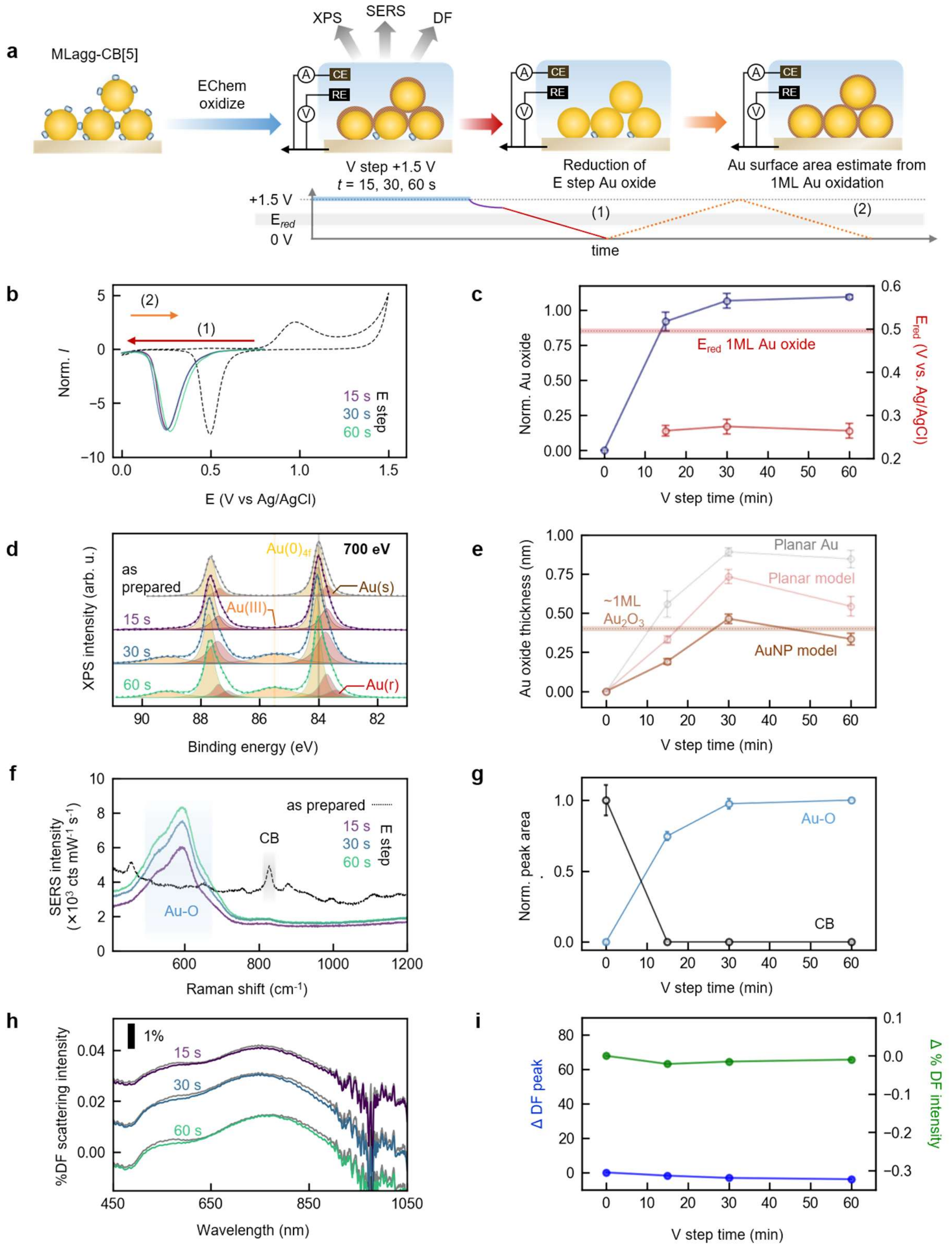

Supplementary Figure 7| Characterisation of Au oxide formation of MLagg-CB[5] from electrochemical (EC) potential step treatment. (**a**) Schematic illustrating the EC potential step (E step, +1.5 V, blue line) treatment of MLagg-CB[5] at various times, followed by XPS, SERS, DF, and electrochemical characterisation of the Au oxide-coated MLagg-CB[5], starting with (1) a linear potential sweep to reduce the Au oxide formed from EC treatment, followed by (2) an oxidative and reductive sweep (0.0 V to +1.5 V and back) to estimate the MLagg-CB[5] surface area from monolayer Au oxide formation. (**b**) Reductive linear sweep voltammograms (1) from OCP ~ +0.7 V to 0.0 V at 100 mV s^{-1} in 1 M potassium phosphate buffer for the reduction of Au oxide formed from EC treatment at varying treatment times. Also shown is a representative cyclic voltammogram (2) of the monolayer Au oxide formation and reduction of the MLagg-CB[5] (0.0 V to +1.5 V and back at 100 mV s^{-1} in 1 M potassium phosphate buffer). Currents were normalised with respect to respective current density of the monolayer Au oxide reduction. (**c**) Ratio of the E step Au oxide reduction current density to the

monolayer Au oxide reduction current density (normalised Au oxide, blue) and E step Au oxide reduction potential (E_{red}, red) vs. E step time. Data points represent mean ± s.d. (n=3 different MLagg-CB[5] samples). Red dotted line highlights the mean E_{red} from monolayer Au oxide reduction. (**d**) Au 4f XPS spectra (solid line), fits (dotted line), and components of MLagg-CB[5] (as prepared) and after 15 s, 30 s and 60 s E step at +1.5 V. Spectra were collected using 700 eV X-ray energy. (**e**) Au oxide thickness estimated from XPS depth profiling data sets on planar Au (grey) and MLagg-CB[5] fitted using planar model (pink) and the AuNP model (brown) after different oxidative E step times. Data points represent the fitted Au oxide thickness, while error bars represent 1σ standard error from the regression, representing the uncertainty of the thickness estimate, not replicate measurements. (**f**) Representative SERS spectra of the MLagg-CB[5] as prepared (black dotted line) and after various oxidative E step treatment times. (**g**) Normalised SERS peak areas of CB[5] (~826 cm^{-1} peak, black) and Au-O (summation of 520, 590, and 660 cm^{-1} peaks, blue) of MLagg-CB[5] after various oxidative E step treatment times. Data points represent the mean ± s.d. (n=3 different MLagg-CB[5] samples). (**h**) Representative DF scattering spectra of MLagg-CB[5], as prepared (grey line), and after various oxidative E step treatment times. (**i**) Change in the chain mode peak wavelength and intensity before and after various oxidative E step treatment times.

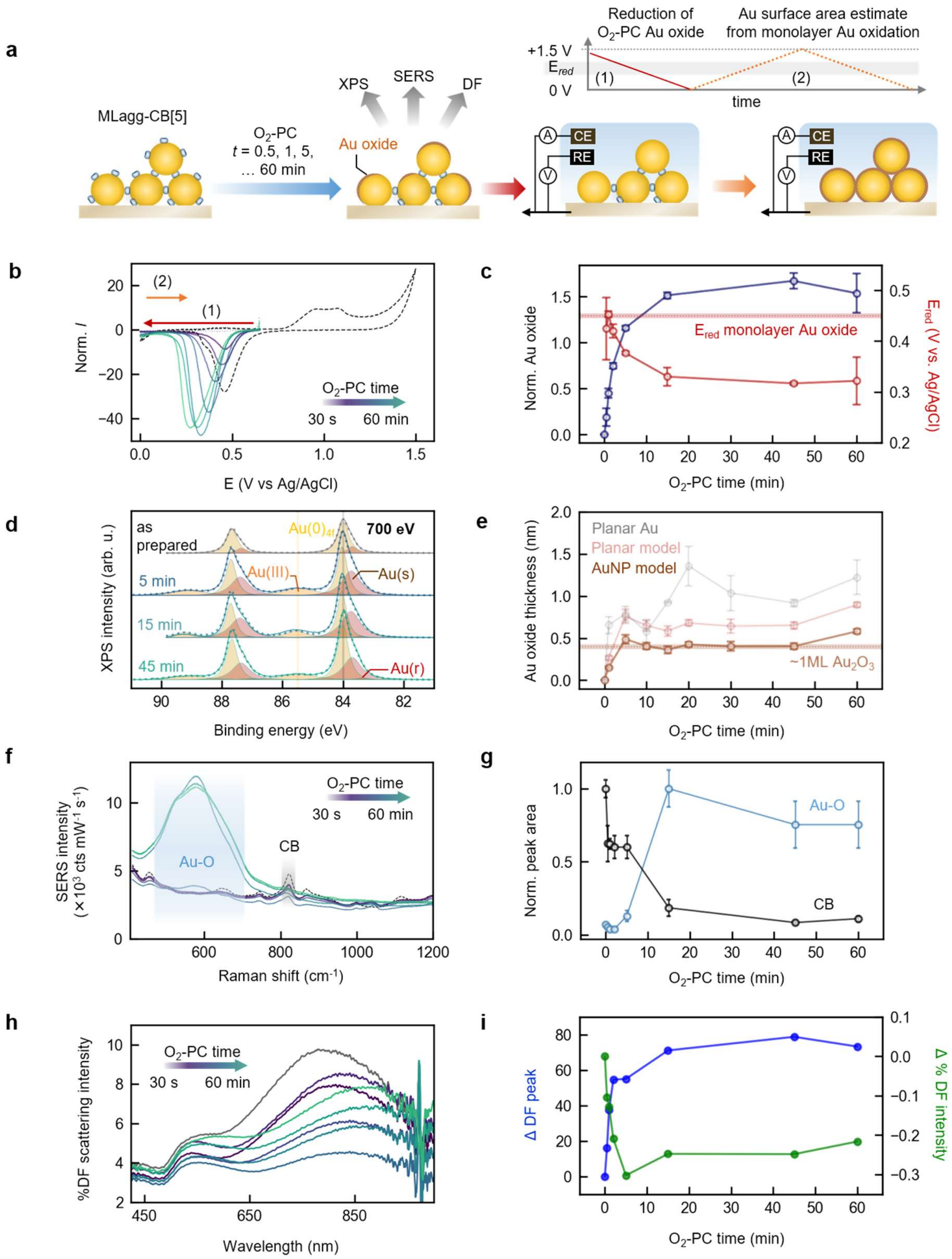

Supplementary Figure 8 | Characterisation of Au oxide formation of MLagg-CB[5] from oxygen plasma cleaning (O_2-PC) treatment. (**a**) Schematic illustrating the O_2-PC treatment of MLagg-CB[5] at various times, followed by XPS, SERS, DF, and electrochemical characterisation of the Au oxide-coated MLagg-CB[5], starting with (1) a linear potential sweep to reduce the Au oxide formed from O_2-PC treatment, followed by (2) an oxidative and reductive potential sweep (0.0 V to +1.5 V and back) to estimate the MLagg-CB[5] surface area from monolayer Au oxide formation. (**b**) Reductive linear sweep voltammograms (1) from OCP ~ +0.7 V to 0.0 V at 100 mV s^{-1} in 1 M potassium phosphate buffer for the reduction of Au oxide formed from O_2-PC at varying treatment times. Also shown is a representative cyclic voltammogram (2) of the monolayer Au oxide formation and reduction of the MLagg-CB[5] (0.0 V to +1.5 V and back at 100 mV s^{-1} in 1 M potassium phosphate buffer). Currents were normalised with respect to respective current density of the monolayer Au oxide

reduction. (**c**) Ratio of the O_2-PC Au oxide reduction current density to the monolayer Au oxide reduction current density (normalised Au oxide, blue) and O_2-PC Au oxide reduction potential (E_{red}, red) vs. O_2-PC treatment time. Data points represent mean ± s.d. (*n*=3 different MLagg-CB[5] samples). Red dotted line highlights the mean E_{red} from monolayer Au oxide reduction. (**d**) Au 4f XPS spectra (solid line) and fits (dotted line) of MLagg-CB[5] (as prepared) and after 5 min, 15 min and 45 min O_2-PC treatment time. Spectra were collected using 700 eV X-ray energy. (**e**) Au oxide thickness estimated from XPS depth profiling data sets on planar Au (grey) and MLagg-CB[5] fitted using planar model (pink) and the AuNP model (brown) after different O_2-PC treatment times. Data points represent the fitted Au oxide thickness, while error bars represent 1σ standard error from the regression, representing the uncertainty of the thickness estimate, not replicate measurements. (**f**) Representative SERS spectra of the MLagg-CB[5] as prepared (black dotted line) and after various O_2-PC treatment times. (**g**) Normalised SERS peak areas of CB[5] (~826 cm^{-1} peak, black) and Au-O (summation of 520, 590, and 660 cm^{-1} peaks, blue) of MLagg-CB[5] after various O_2-PC treatment times. Data points represent the mean ± s.d. (*n*=3 different MLagg-CB[5] samples). (**h**) DF scattering spectra of MLagg-CB[5], as prepared (grey line), and after various O_2-PC treatment times. (**i**) Change in the chain mode peak wavelength and intensity before and after various O_2-PC treatment times.

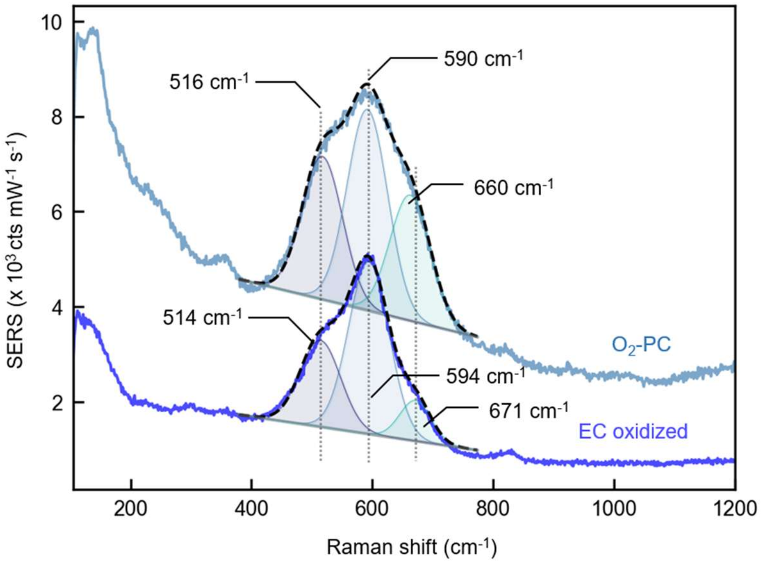

Supplementary Figure 9 | SERS spectra of MLagg-CB[5] after oxidation with O_2-PC and electrochemical potential step. Overlaid SERS spectra of MLagg-CB[5] after 45 min O_2-PC treatment (light blue line) and electrochemical oxidation (+1.5 V vs Ag/AgCl for 60 s in 50 mM potassium phosphate buffer, pH 7.0, blue line). Spectra were measured on dried MLagg samples. Also shown are peak fits of the three main Au-O vibrational bands at 520 cm^{-1}, 590 cm^{-1}, and 660 cm^{-1}. Spectra are not offset.

Supplementary Note 5. Surface transformations during EC-ReSERS

EC-SERS and EC-DF during EC-ReSERS

To study the nanogap transformations in more detail, we examine the spectral evolution of the MLagg through a single oxidation-reduction cycle (**Supplementary Figure 10**). Initially, at 0 V, the SERS spectrum shows that the nanogap is filled with CB[5] molecules, along with Au-Cl at 240 cm^{-1} (**Supplementary Figure 10b, yellow points**). As the potential is ramped from 0 V to +0.45 V, we observe a small oxidative current peak. The SERS spectra show that this is associated with the rise of the Au-Cl line, which also shifts to 265 cm^{-1}, thus suggesting the following surface reaction, where Au atoms are oxidised from Au(0) to Au(I) and coordinate with Cl^- to form Au-Cl:

Au surface oxidation	$Au(0) + Cl^- \rightarrow Au\text{-}Cl_{(surface)} + e^-$	eqn. 1

The corresponding DF spectra show a ~10 nm red shift in the coupled plasmon mode and a decrease in scattering intensity (**Supplementary Figure 10c,d**). Previous works studying the plasmon mode shifts of AuNPs electrochemically cycled in Cl^--containing electrolyte have also reported similar shifts and dampening[48,49]. Simulations of the scattering spectra[49] suggested that these shifts are consistent with the formation of electron-depleted surface Au atoms with a layer of bound Cl^-, or the formation of Au(I/III)-Cl complexes on the surface of the AuNPs[48–50]. However, due to a lack of data characterising the surface chemistry in these previous studies, the surface species was not identified at the time. Our correlated EC-SERS and EC-DF data now suggest that the formation of Au-Cl is indeed the case.

As the potential is increased to +0.9 V, the Au-Cl line reaches a peak, while the CB[5] line (black points) decreases. Au-O lines also start to appear, suggesting that this is a pre-oxide formation stage, where OH^- start to displace other anions such as Cl^- on the Au surface[51,52] (**eqn. 2**). DF spectra show a minor blue shift in the plasmon mode and a continued decrease in intensity. As the potential is further increased to a current peak at V_o~+1.1 V, further oxidation results in the formation of Au_2O_3 (**eqn. 3-4**), alongside a variable fraction of Au(III)-Cl seen at ~345-360 cm^{-1} (**eqn. 5**). A rapid drop in scattering intensity is also observed, consistent with the decrease in electron density as more surface Au atoms are oxidised (**Supplementary Figure 10f**).[50]

Anion displacement:	$Au\text{-}Cl_{(surface)} + 2H_2O \rightarrow Au\text{-}OH_{(surface)} + H_3O^+ + Cl^-$	eqn. 2
	$Au\text{-}OH_{(surface)} + H_2O \rightarrow Au\text{-}O^-_{(surface)} + H_3O^+$	eqn. 3
Further oxidation:	$2Au\text{-}O^-_{(surface)} + 3H_2O \rightarrow Au_2O_{3(surface)} + 2H_3O^+ + 2e^-$	eqn. 4
	$Au\text{-}Cl_{(surface)} + 2Cl^- \rightarrow Au(III)\text{-}Cl_{3(surface)} + 2e^-$	eqn. 5

The oxide persists until the potential is decreased again to V_{or}~+0.4 V (oxide reduction, at the main reduction peak) when the Au(III) states convert to Au-Cl and Au(0), while CB[5] returns inside the nanogap (**eqn. 6-8**). The scattering intensity also increases rapidly to its initial state, along with a sharp ~20 nm blue shift, suggesting the restoration of electron density. This is followed by a ~5 nm red shift as more CB[5] returns to the nanogap (**Supplementary Figure 10g**). An additional line at ~670 cm^{-1}, previously ascribed to Au-O[18,53], persists until V<-0.8V when it is replaced by an enhanced CB[5] signal, after which the cycle repeats.

Reduction to Au-Cl	$Au_2O_{3(surface)} + 4Cl^- + 6H_3O^+ + 2e^- \rightarrow 2Au\text{-}Cl_{(surface)} + 9H_2O$	eqn. 6
	$Au(III)\text{-}Cl_{3(surface)} + 2e^- \rightarrow Au\text{-}Cl_{(surface)} + 2Cl^-$	eqn. 7
Further reduction	$Au\text{-}Cl_{(surface)} + e^- \rightarrow Au(0) + Cl^-$	eqn. 8

To further validate the assignment and electrochemical behaviour of the Au-Cl peak at 240-265 cm^{-1}, we performed additional EC-SERS experiments using substrates that do not contain CB[5]. Specifically, we tested salt-aggregated AuNP films (MLagg-NaCl, **Supplementary Figure 11a-c**) and a MLagg functionalised with nicotinic acid (MLagg-NIA, **Supplementary Figure 11d-f**), both of which exhibit no overlapping peaks near 240-280 cm^{-1} from organic ligands. In both systems, we observed clear potential-dependent evolution of the Au-Cl peak during

the oxidation-reduction cycle: the Au-Cl signal emerges during the anodic sweep, reappears following Au oxide reduction, and desorbs upon further cathodic bias-mirroring the trends observed in MLagg-CB[5]. While there are some differences in peak position and intensity, likely due to variations in available surface binding sites and ligand-Au interactions, the overall behaviour of the Au-Cl signal is consistent across systems. This supports our interpretation that the Au-Cl peak originates from a surface-stabilized covalent adlayer and is not an artifact of spectral overlap with CB[5].

Potential step relaxation

To study the intermediate stages of EC-ReSERS in more detail, the MLagg was oxidised and reduced in a series of ±0.1 V increments, with relaxations to open-circuit potential (OCP) between each potential step (**Supplementary Figure 12-Supplementary Figure 13**). This scheme allows us to probe intermediate states, their stability, and their impact on the surface of the AuNPs. By comparing the SERS spectra during and after the application of a step potential and by tracking the post-step OCP, it can be determined, for example, if the observed peak shifts during the application of a potential are due to transient changes in the ion distribution in the EDL or due to chemisorption of a surface species.

Supplementary Figure 12 shows the SERS spectra during and after each step potential step. Since oxidation does not require additional CB[5], it is performed here in 50 mM buffer without extra Cl^-, while for reduction, 1 mM CB[5] is added to avoid sintering. Before any applied potential, the OCP of the MLagg-CB[5] is 0.2 V, and the SERS spectrum shows the characteristic CB[5] spectrum with some adsorbed Cl^-. Similar to the results from CV, as the potential is stepped in the positive direction, the Au-Cl line increases in intensity and gradually shifts from 240 to 265 cm^{-1}. These peak shifts are notably stable even after relaxation to OCP. After each step potential, the OCP also does not return to the initial state; rather, it gradually increases as the Au-Cl line increases in intensity and shifts to higher energy. These results suggest that the peak shift is not due to a Stark shift solely from the externally applied potential, but due to additional stable changes in the surface chemistry, specifically the formation of a Au-Cl surface compound. The observed increases in both peak intensity and position are consistent with an increase in Au-Cl surface coverage density as the applied potential is increased. After +0.8 V, when the Au-Cl displaces most CB[5] from the gap, the OCP relaxes to ~0.7 V, suggesting a surface chemical potential dominated by a higher coverage of Au-Cl.

As the potential is stepped more positive, Au is further oxidised, and Au(III)-Cl and Au-O lines start to appear by +0.9 V. Here, OH^- ions displace Cl^- to form Au(I/III)-OH . The Au(III)-Cl line shifts from 345 cm^{-1} to 355 cm^{-1} as more Au(III) co-coordinated Cl^- are displaced by OH^-. As OH^- ions are further deprotonated to form oxide ions (O^{2-}), Au(III) ions are pushed out from the surface as O^{2-} penetrates the surface layer in a place-exchange process to alleviate electrostatic repulsion[51,54]. Three distinct Au(III)-O peaks, corresponding to 3 inequivalent O^{2-} positions in the Au_2O_3 lattice, first appear at 480, 560, and 630 cm^{-1} as replicated in DFT calculations (**Supplementary Figure 10f** (inset), **Supplementary Figure 14**). As the potential is increased to +1.3 V, the lines shift to higher energies reaching 520, 590, and 660 cm^{-1}, respectively. By this point, the OCP also gradually shifts from 0.7 to 0.9 V as the surface potential becomes dominated by Au_2O_3.

In the nanogap, the penetration depth of Au_2O_3 is set by the available gap size. As a monolayer (ML) of oxidised Au atoms expands to 0.4 nm thickness[40], for d=0.9 nm nanogaps, this means only 1-2 ML of Au_2O_3 can form on either facet before meeting in the middle to form a plug, as confirmed by XPS depth profiling (**Supplementary Figure 6**). We note that previous *in-situ* EXAFS of electrochemically-grown Au_2O_3 shows that the structure is highly disordered[55]. Remaining Au(III)-Cl species can also contribute to the lattice disorder. Indeed, the formation of a hydrated Au oxide is supported by XPS O 1s spectra, which shows a peak corresponding to OH^- (**Extended Data Figure 1**). Oxidising the MLagg in D_2O also shows 5-10 cm^{-1} shifts in the 520 and 590 cm^{-1} Au-O lines (**Supplementary Figure 15**), while O-D stretching vibrations are also evident at 2500 cm^{-1}. These suggest water molecules are incorporated into the oxide structure, either as absorbed water or as part of the lattice in the form of hydroxyl groups.

In the first phase of the reduction sequence in the presence of CB[5], Au oxide and Au(III)-Cl reduce, forming an Au-Cl intermediate. During this intermediate state, the CB[5] scaffolding molecule starts to rebind into the

nanogap. This rebinding process appears critical for restoring the nanogap architecture; however, the precise structure and role of the Au-Cl species during re-scaffolding have not been completely understood previously.

To address this gap, literature reports provide valuable context for the possible configurations and chemical behaviours of Au-Cl on Au surfaces. Previous STM and DFT studies investigating the low-temperature exposure of Au surfaces with Cl_2 shows that at >0.33 ML surface coverage, Cl atoms form a honeycomb-like superstructure with Au adatoms released from the surface due to adsorbate-induced surface reconstruction[56]. This surface compound has also been characterised as $AuCl_2$ quasi-molecules, which can form 3D agglomerations at room temperature[57]. A similar Au_2Cl_2 surface compound can also form upon incubating planar Au with HCl in room temperature[58]. In fact, Rzeźnicka *et al*. used this Au-Cl superstructure as a template to form a hybrid inorganic-organic layered surface structure with 4,4-bipyridine[58]. The self-assembly of such a 3D surface structure was driven by aurophilic and π–π stacking interactions, and due to the covalent-like Au-Cl bond[59], the organic molecule can also form H-bonding interactions with Au-Cl. Characterisation of the layered structure with SIMS confirming the formation of a surface Au_2Cl_2 (Au-Cl-Au-Au-Cl-Au) chain structure, while Raman and TERS showed a 255 cm^{-1} peak[58].

The 255 cm^{-1} peak observed in Rzeźnicka's work is close to the 265 cm^{-1} peak observed in our data, suggesting the formation of a Au-Cl surface species. Like 4,4-bipyridine, CB[5] has also been demonstrated to form electrostatic and H-bonding interactions with $HAuCl_4$.[60,61] The Raman spectrum of a bulk CB[5]$\cdot x[AuCl_4]^-$ precipitate (**Supplementary Figure 16**) shows peak shifts and intensity changes in the CB[5] lines, reflecting the interaction between the CB[5] molecules and the Au-Cl species. Likewise, the SERS spectra of the MLagg when the nanogap features the Au-Cl species also show similar changes in CB[5] lines such as the CN and CH vibrations (**Supplementary Figure 13a**), suggesting an interaction between CB[5] and the Au-Cl surface species.

These observations support our hypothesis that interactions between CB[5] and the Au-Cl adlayer are a key driving force for the re-scaffolding process, guiding CB[5] molecules back into the nanogap during the Au-Cl stage. Further investigation of the precise structure and dynamics of the Au-Cl surface species is warranted, as literature examples demonstrate its strong potential to interact with organic molecules, an interaction that appears central to efficient nanogap regeneration in our system.

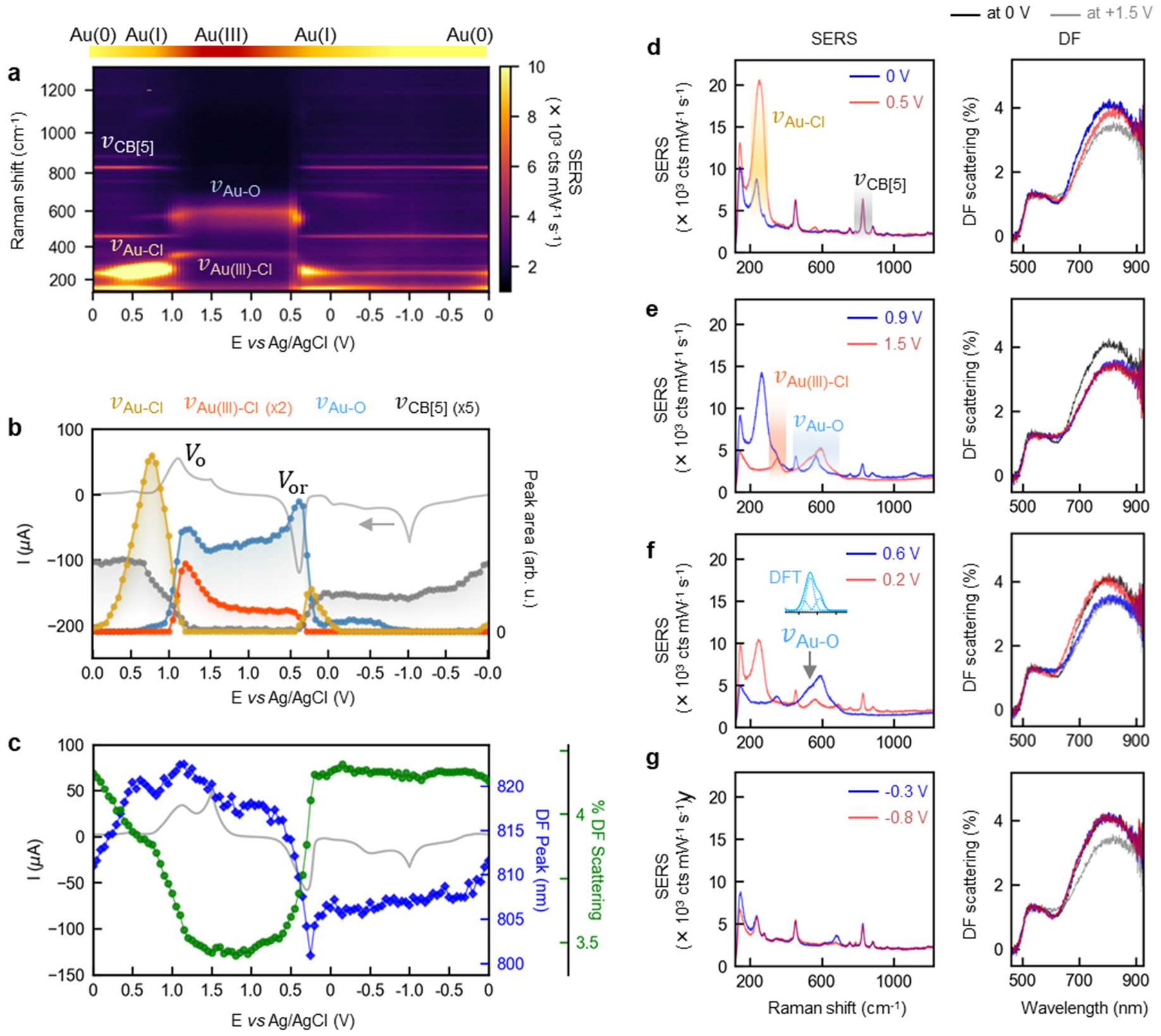

Supplementary Figure 10 | Different chemical transitions in SERS and DF of Au MLagg in an oxidation-reduction cycle. (**a**) SERS spectra (1 s integration time, 785 nm laser excitation with 1 mW power) through a single oxidation-reduction cycle (scan rate 50 mV s^{-1}) in 0.1 mM CB[5] in 50 mM potassium phosphate buffer (pH 7.0). (**b**) Extracted peaks at Au-Cl (245 cm^{-1}, yellow points), Au(III)-Cl (345 cm^{-1}, orange points), Au-O (multiple peaks centred at 590 cm^{-1}, light blue points), and CB[5] (826 cm^{-1}, black points), together with current (grey solid line) at each applied potential. (**c**) Extracted dark-field (DF) spectral peak positions (blue points) and scattering strength (green points) together with current (grey solid line) at each applied potential. (**d-g**) SERS and DF spectra at different points during the ORC. The DF spectra of the MLagg at 0 V (black) and +1.5 V (grey) are also shown for reference. *In-situ* SERS and DF spectra were measured separately on different MLagg samples that underwent the same treatment and ORCs.

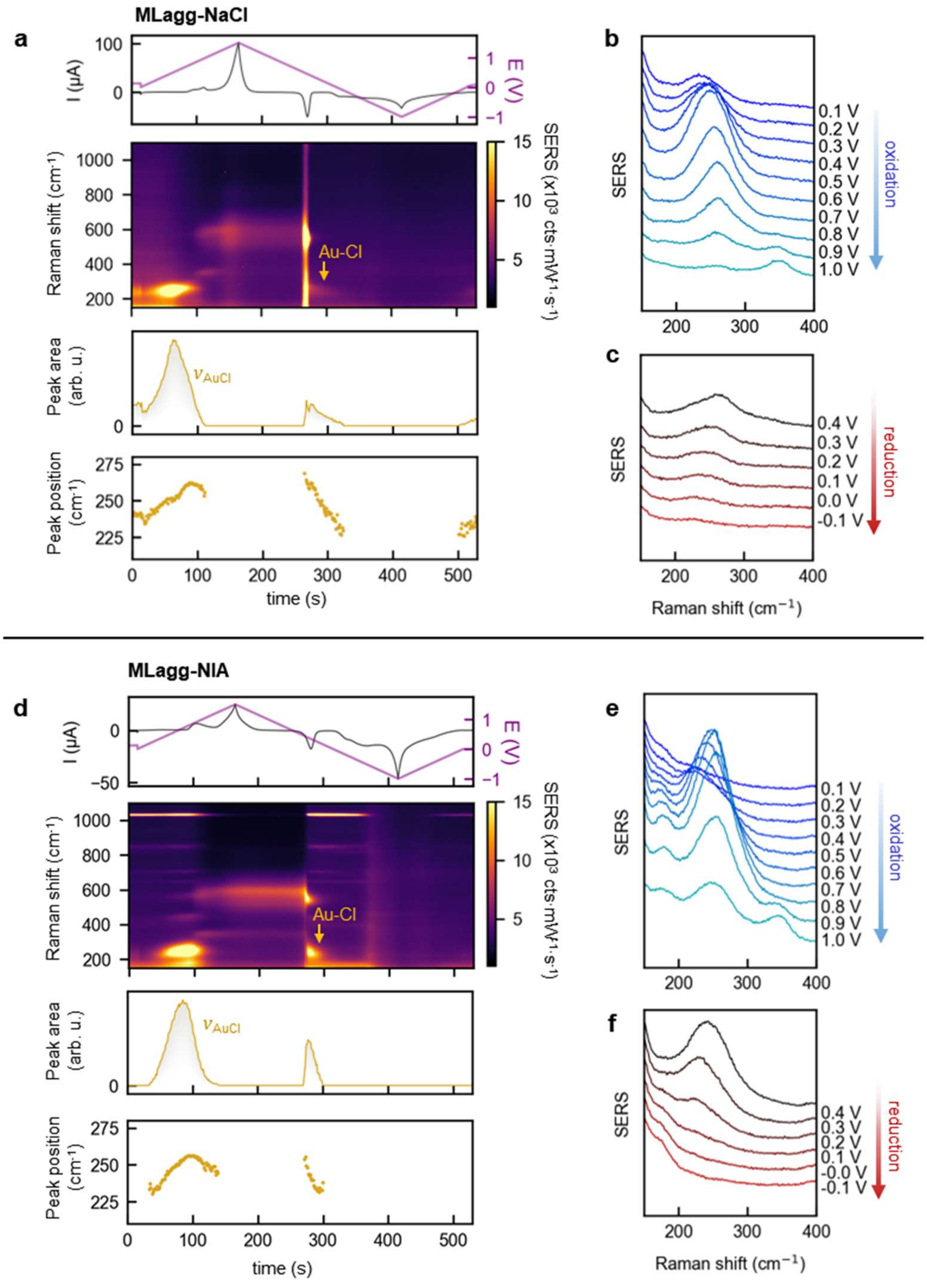

Supplementary Figure 11 | Evolution of Au-Cl in other AuNP aggregate systems. (**a-c**) Salt-aggregated AuNPs (MLagg-NaCl) undergoing a single ORC in 50 mM potassium phosphate buffer and 1 mM KCl at 10 mV·s^{-1}. (**a**) Time-series SERS spectra along with extracted Au-Cl peak area and intensity per SERS spectrum. SERS spectra at low wavenumber region measured at indicated applied potentials during the oxidative (**b**) and reductive (**c**) sweeps. (**d-f**) Nicotinic acid functionalised aggregated AuNPs (MLagg-NIA) undergoing a single ORC in 1 mM nicotinic acid (NIA), 1 mM KCl, and 50 mM potassium phosphate buffer at 10 mV·s^{-1}. (**d**) Time-series SERS spectra along with extracted Au-Cl peak area and intensity per SERS spectrum. SERS spectra at low wavenumber region measured at indicated applied potentials during the oxidative (**e**) and reductive (**f**) sweeps.

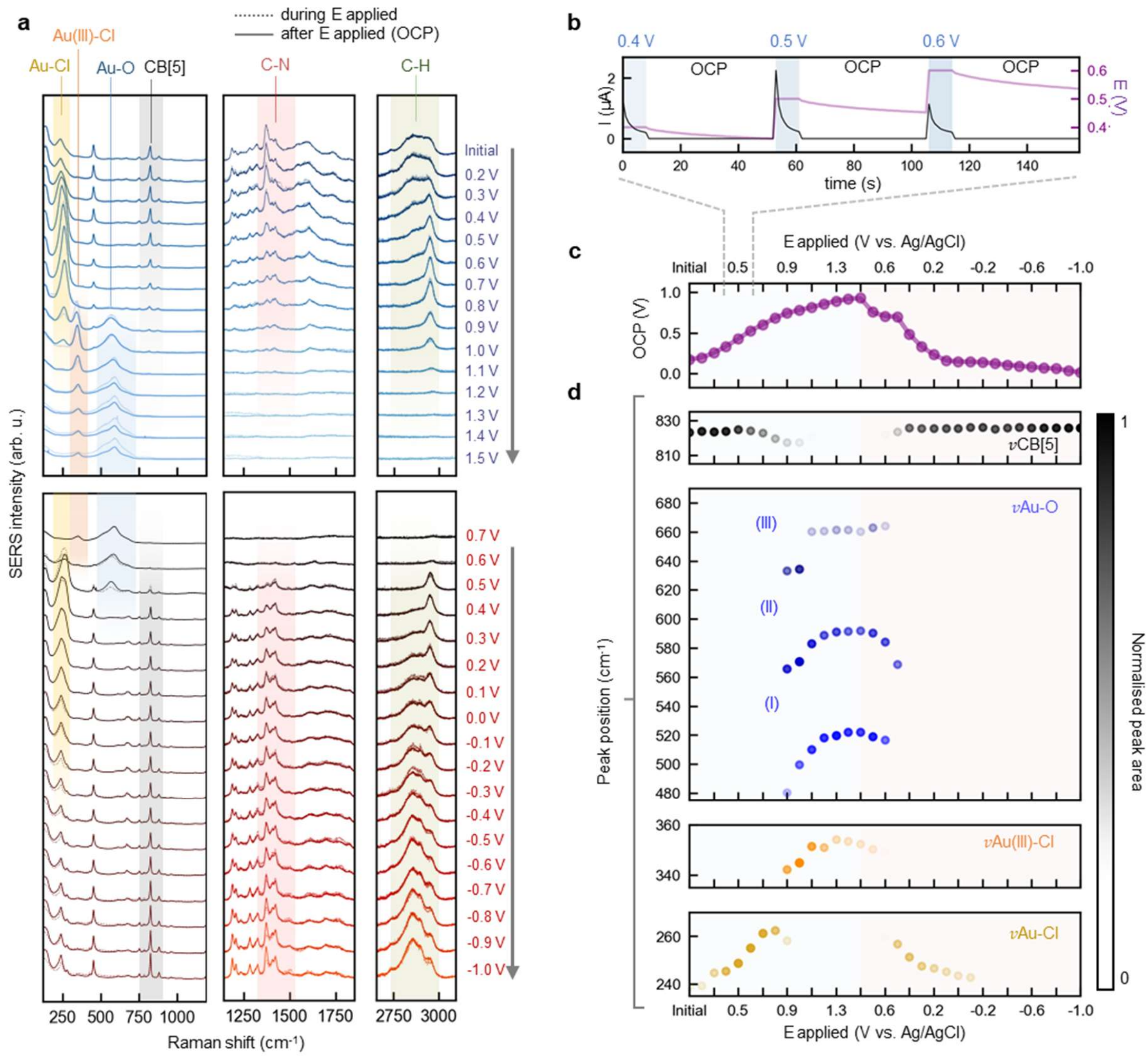

Supplementary Figure 12 | Progression of SERS and OCP after potential step oxidation and reduction of MLagg. (**a**) SERS spectra in different wavenumber regions, showing the progression of different peaks as labelled during positive-going potential steps from 0.2 V to +1.5 V in 50 mM potassium phosphate buffer (top panels) and negative-going potential steps (bottom panels) from +0.7 V to -1.0 V in 1 mM CB[5], 50 mM potassium phosphate buffer. SERS spectra were measured during the applied potential (dotted line) and after relaxation to open-circuit potential (OCP, solid line). (**b**) Representative potential (purple line) and current traces (black line) of the MLagg-CB[5] undergoing sequential potential steps (+0.4 V, +0.5 V, and +0.6 V). Constant potential steps were held for 10 s (blue shaded regions), followed by 45 s relaxation to OCP. (**c**) OCP of the MLagg-CB[5] after an applied step potential. OCP was measured 45 s after the applied potential. (**d**) Peak position (points) and normalised peak area (colour gradient) of the indicated SERS lines after applied step potentials (measured at OCP).

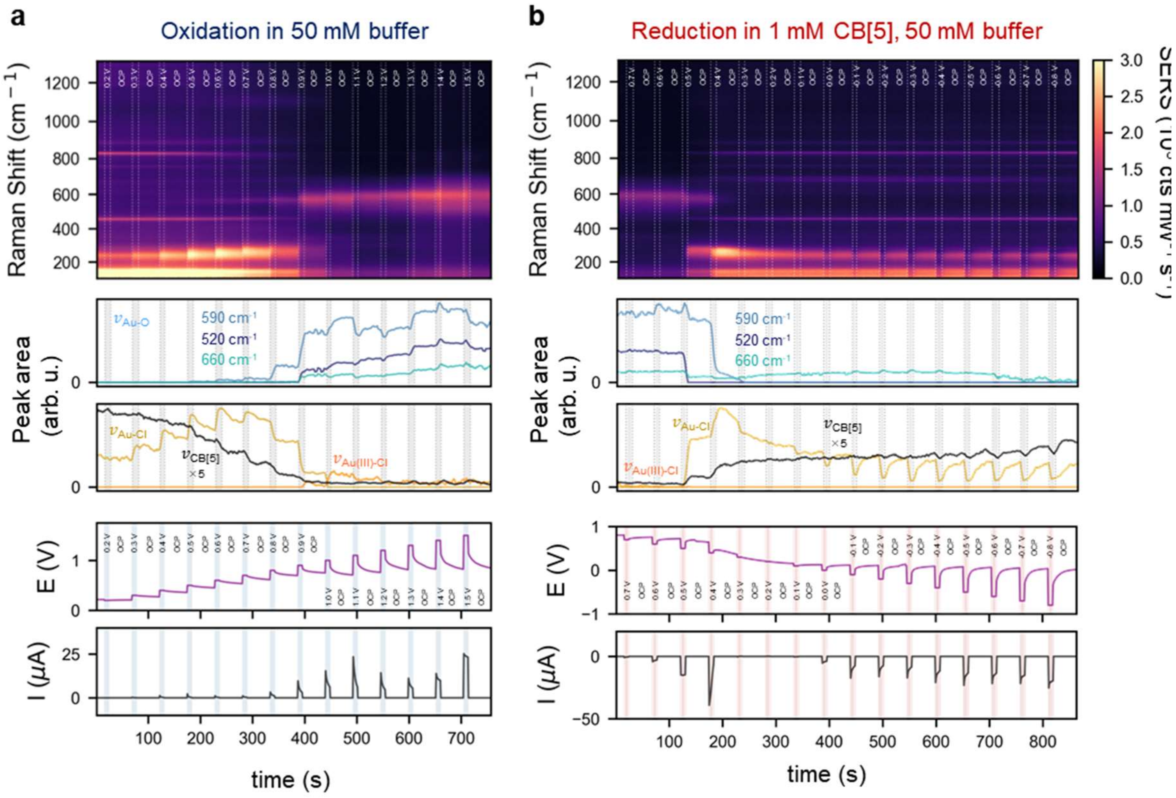

Supplementary Figure 13 | Potential step oxidation and reduction of MLagg-CB[5]. (**a,b**) Time-series SERS spectra from MLagg-CB[5] undergoing sequential potential steps (in increments of 0.1 V) for 10 s, followed by relaxation to open-circuit potential (OCP) for 45 s in (**a**) 50 mM potassium phosphate buffer (pH 7.0) from 0.2 V to +1.5 V and (**b**) 1 mM CB[5] and 50 mM potassium phosphate buffer (pH 7.0) from +0.7 V to -0.8 V. The peak areas of Au-O (520, 590, 660 cm^{-1} peaks, blue lines), CB[5] (~826 cm^{-1}, black lines), Au(III)-Cl (~345 cm^{-1}, orange line), and Au-Cl (~250 cm^{-1}, yellow line) are plotted per SERS spectrum. The applied potentials followed by the measured OCP (purple line) and the corresponding current responses (black line) are plotted *vs* time.

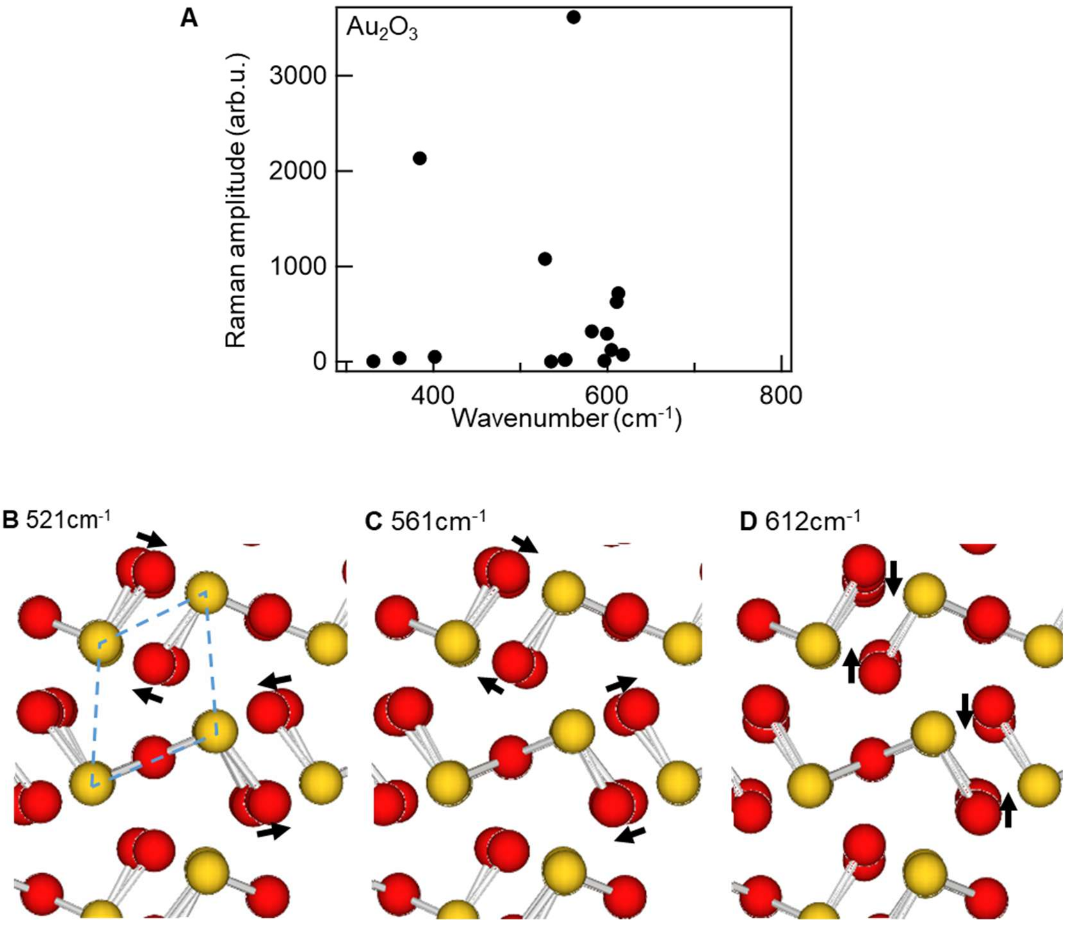

Supplementary Figure 14 | **Main vibrational modes of Au_2O_3 from DFT.** (**A**) Phonon modes and their Raman intensities. (**B-D**) Arrows show movement of O atoms (red) vs Au atoms (gold) for the three main modes at (**B**) 521cm^{-1}, (**C**) 561cm^{-1}, and (**D**) 612cm^{-1}.

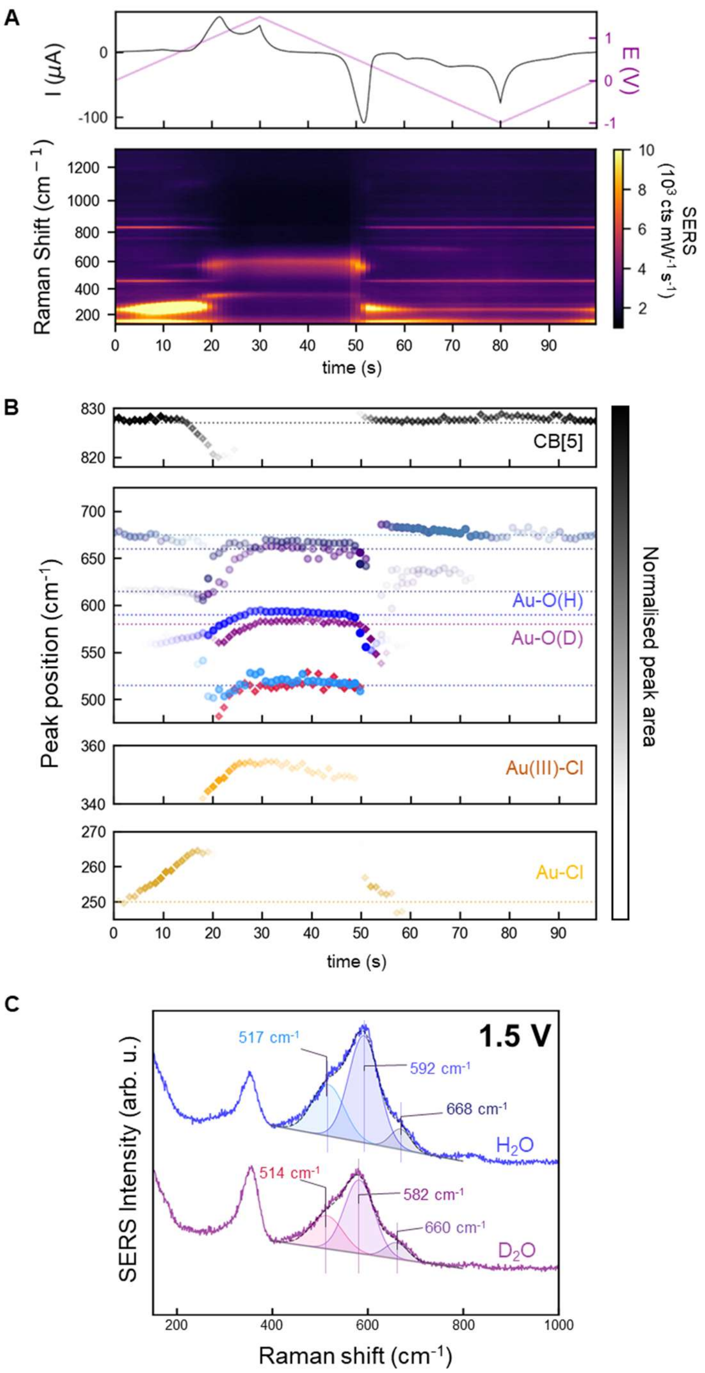

Supplementary Figure 15 | Cyclic voltammetry (CV) and time-series SERS of MLagg-CB[5] in 100 µM CB[5] and 50 mM potassium phosphate buffer (pH 7.0) in H_2O or D_2O. (**a**) Time-series SERS spectra from MLagg-CB[5] cycled between +1.5 V and -1.0 V in 50 mM potassium phosphate buffer (pH 7.0) in H_2O at 50 mV s^{-1} for 5 scans. The applied potential (purple line) and corresponding current response (black line) are plotted with time. (**b**) Peak positions of CB[5] (black), various Au-O lines in H_2O (blue points) or D_2O (pink and purple points), Au(III)-Cl (orange points), and Au-Cl (yellow points) with time. Points correspond to the time-series SERS spectra in (**a**). The shade of each point represents the normalised peak area, with darker points corresponding to higher normalised peak areas. (**c**) SERS spectrum of the MLagg-CB[5] at +1.5 V during the anodic potential sweep in H_2O or D_2O. Three fitted Au-O peaks and the fitted spectrum (black dashed line) are shown. Oxidising the MLagg in D_2O shows 3-10 cm^{-1} shifts to lower energies in the Au-O lines suggesting water molecules are incorporated into the oxide structure, either as absorbed water or as part of the lattice in the form of hydroxyl groups.

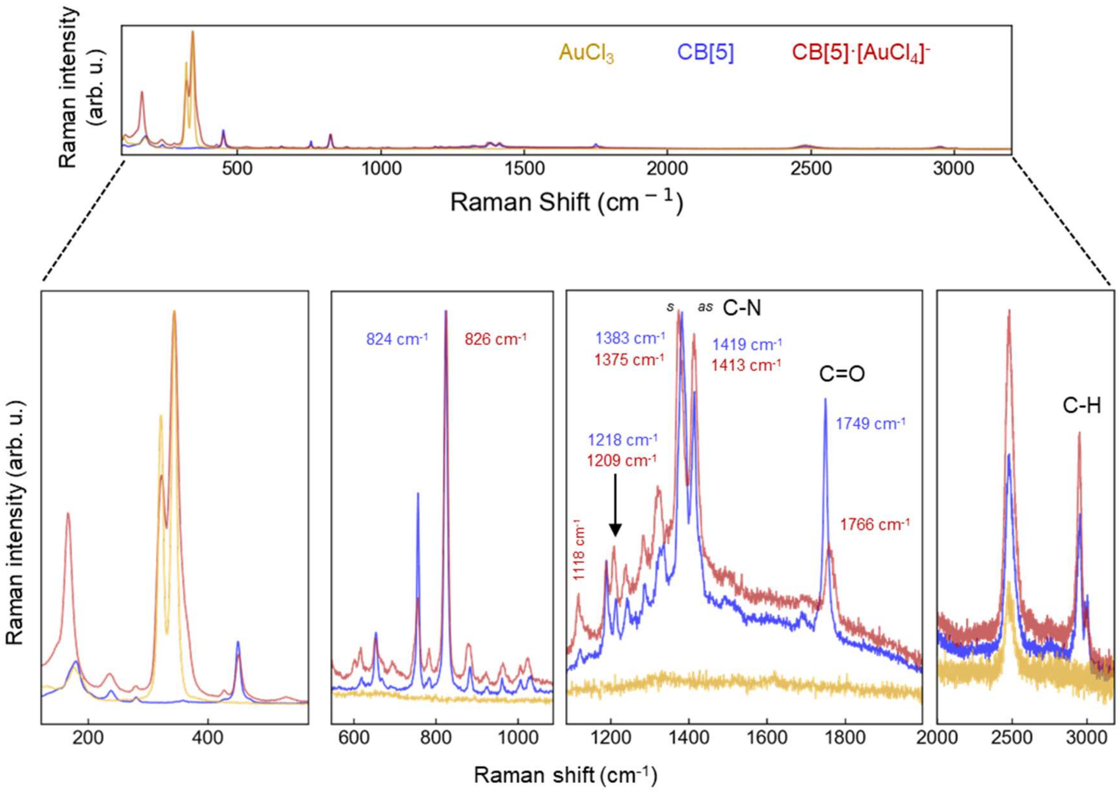

Supplementary Figure 16 | Raman of $AuCl_3$, CB[5] and CB[5]·$[AuCl_4]^-$. (*top*) Full Raman spectral scan of Au(III)Cl_3 (yellow line), CB[5] (blue line) and CB[5]·$[AuCl_4]^-$ (red line). Spectra were collected with 20 s integration time using a 785 nm excitation laser at 12.4 mW power. CB[5] and CB[5]·$[AuCl_4]^-$ spectra were normalised with respect to the ~826 cm^{-1} CB[5] peak, while the Au(III)Cl_3 spectrum was normalised with respect to the Au(III)-Cl ~345 cm^{-1} peak of CB[5]·$[AuCl_4]^-$. (*bottom*) Close-up of different spectral regions, with CB[5] peak shifts highlighted. Peak at 2500 cm^{-1} is an artifact from the sample holder.

Supplementary Note 6. AuNP facets after ORCs

To further investigate how repeated oxidation-reduction cycles (ORCs) reshape the surface morphology of AuNPs, we electrochemically characterised the evolution of Au surface facets. These measurements were conducted on MLagg-CB[5] both with and without CB[5] scaffold molecules. We employed lead underpotential deposition (Pb-UPD) as a facet-sensitive probe, exploiting the fact that Pb atoms preferentially deposit on specific crystallographic facets of gold at distinct potentials[62]. The voltammetric features corresponding to Pb monolayer deposition and stripping thus provides a fingerprint of the surface atomic arrangement and enable us to monitor how this structure evolves over multiple ORCs. SEM images of an as-prepared MLagg confirm that the nanoparticles have different shapes (cuboctahedron, rhombicuboctahedron, pentagonal bipyramid) with surfaces featuring (111), (110) and (100) crystallographic orientations (**Supplementary Figure 17a**). The Pb UPD voltammogram of the as-prepared AuNPs shows that the initial surface is indeed rich in (111) facets with some (110) and (100) character (**Supplementary Figure 17d**). The Au(111) stripping peak is split (3s,3s'), with the higher-potential stripping peak (3s, -230 mV *vs* Ag/AgCl) corresponding to Pb desorption from well-ordered terraces, and the lower potential peak (3s', -250 mV) associated with Pb desorption from terraces less than four atoms wide[63,64]. The dominant 3s peak indicates surfaces of well-ordered (111) terraces with few steps. Additional broad (111) features at higher potentials (4s at 0 mV corresponding to Pb desorption from step edges and 5s at 200 mV for wide, well-ordered terraces) are also consistent with the presence of a mix of (111) terraces and step edges.

After oxidation-reduction cycles, Pb UPD voltammograms reveal evolutions in the Au(111) doublet peak (3s, 3s') as well as the 4s and 5s peaks (**Supplementary Figure 17d,e**). With more ORCs, the 3s/3s' peak ratio decreases. Previous studies on planar Au and AuNPs show that the 3s peak decreases as step density increases, since rougher surfaces disrupt the ordered Pb adlayer structure[63,64]. After cycle 10, the 3s/3s' ratio when CB[5] is present is four-fold larger than without CB[5] during re-scaffolding (**Supplementary Figure 17f**). This suggests that the typical loss of Au(111) domains due to nanoparticle roughening (clearly seen in SEM, **Supplementary Figure 17c**), is greatly reduced when CB[5] is present (as also seen in the retention of distinguishable facets in SEM, **Supplementary Figure 17b**). The scale of roughening is also very different (**Supplementary Figure 17b,c**), showing how CB[5] changes facet stability, preserving Au(111) character and limiting excessive step formation. In contrast, without CB[5], the shift of the 4s peak toward Au(110) and the 5s peak toward Au(554) confirm the loss of large (111) terraces and the transition to a roughened, high-index surface.

Overall these results imply that CB[5] plays a crucial role in preventing excessive roughening, preserving terrace structure, and in stabilizing the AuNP nanogaps, whereas its absence leads to greater surface roughening and nanoparticle fusion. We however note that characterization of Au facets using Pb UPD and SEM provides only insights into *overall* AuNP surface morphology, without directly examining the nanogaps which SERS probes (since these form <5% of the total surface area).

Here, SERS provides much more pertinent information on the nanogaps, revealing details that are not obvious from Pb UPD alone. Cycling in the presence of CB[5] results in stable SERS spectra with a constant background, indicating that the nanogaps remain structurally intact despite the moderate overall surface roughening observed in Pb UPD (**Supplementary Figure 17**). However, in the absence of CB[5], SERS background intensities double after the first cycle, followed by a progressive decrease in SERS intensity with further cycles (**Supplementary Figure 17**). This suggests roughening within the nanogaps leading to greater plasmonic damping and a loss of well-defined hotspot regions as early as cycle 1, whereas Pb UPD, which characterizes the entire AuNP surface, only indicates a degree of roughening comparable to cycling with CB[5]. Unlike Pb UPD, which probes both SERS-active and non-SERS-active regions, SERS selectively detects nanogap changes, revealing structural evolution that may not yet be apparent in the global facet analysis.

Furthermore, the sharp SERS peaks observed in CB[5]-stabilised nanogaps show no evidence of broadening from Au adatoms (or 'picocavities'[65]), suggesting that CB[5] not only preserves the structural integrity of nanogaps but may also enhance the atomic order for the Au facets within them.

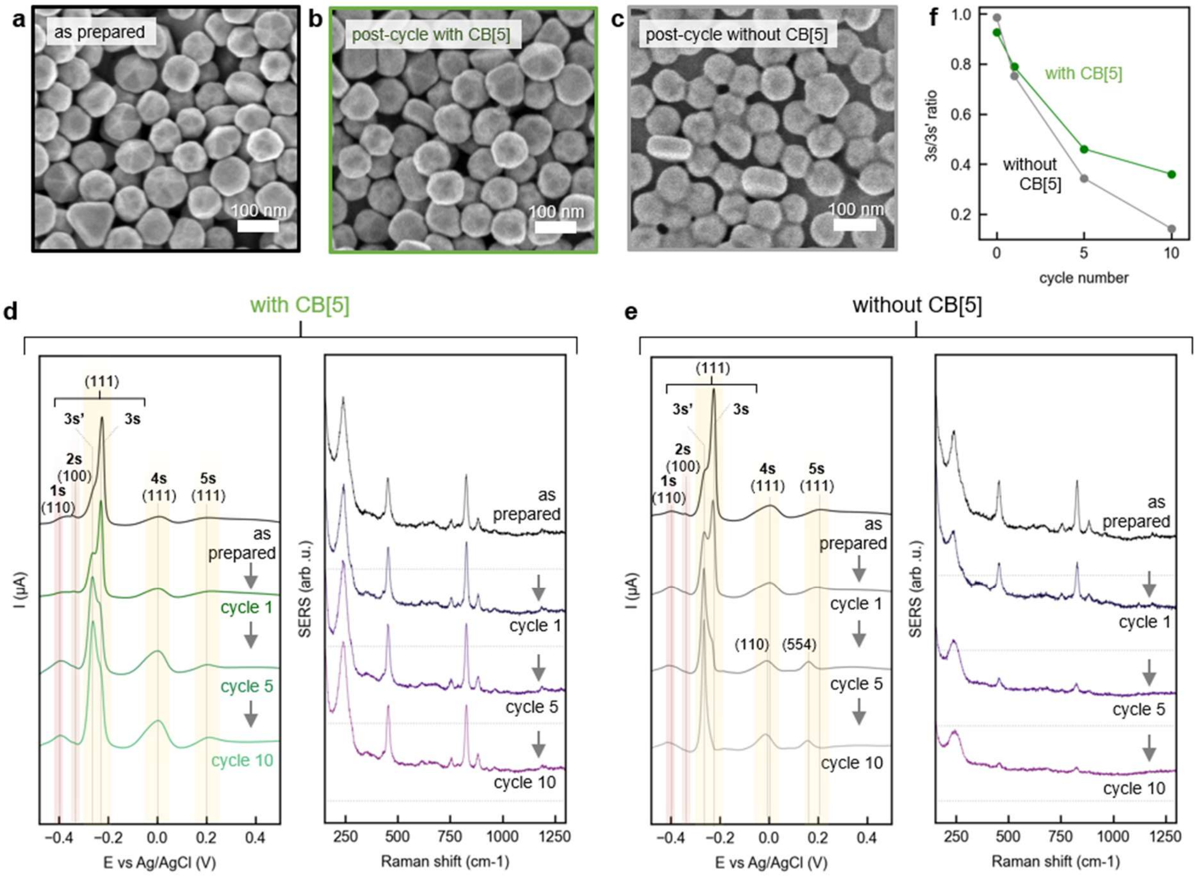

Supplementary Figure 17 | MLagg-CB[5] morphology after multiple ORCs. (a-c) SEMs of (**a**) initial MLagg, and after 10 oxidation and reduction cycles both (**b**) with CB[5], and (**c**) without CB[5]. (**d,e**) Positive sweeps from UPD stripping of Pd monolayers on AuNP MLagg samples and corresponding SERS spectra, taken for increasing numbers of rescaffolding cycles with/out CB[5]. (**f**) Extracted ratio of 3s and 3s' peaks with cycling.

Supplementary Note 7. ORCs with other SERS substrates

To assess whether CB[5] can stabilise other classes of SERS substrates beyond MLagg-CB[5], we investigated the effect of electrochemical cycling on two distinct systems: (1) a NaCl-aggregated thin-film AuNP substrate (MLagg-NaCl), and (2) an electrochemically roughened planar Au electrode (EC-roughened Au). Both systems are known to exhibit dynamic structural changes under potential cycling, often leading to irreproducible SERS signals.

The MLagg-NaCl SERS substrate forms interstitial nanogaps between AuNPs aggregated via ionic screening. These nanogaps, however, are not chemically stabilised and exhibit substantial variation in spacing and orientation. When subjected to oxidation-reduction cycles (ORCs) in buffer alone, the MLagg-NaCl system undergoes progressive sintering of nanogaps, leading to diminished SERS enhancement and loss of signal reproducibility (**Supplementary Figure 18a-c**). However, when CB[5] and Cl^- is introduced into the electrolyte during ORCs (**Supplementary Figure 18d-f**), the MLagg-NaCl demonstrates SERS reproducibility after the first few cycles, with the CB[5], Au oxide, Au-Cl and Au(III)-Cl SERS signals exhibiting consistent signal changes across multiple cycles. The SERS background also decreases and stabilises after the first few cycles, suggesting some degree of structural reorganization occurs within the nanogaps. This suggests that CB[5] plays a role in arresting further sintering, likely through nanogap rebinding and surface passivation, as observed in the MLagg-CB[5] system.

EC-roughened Au SERS substrates feature ill-defined nanoscale geometries, with SERS hotspots typically formed from sharp edges, corners, crevices and protrusions on the rough surface. Since the features are not well-defined nor well-stabilised, the SERS hotspots on this substrate are notably dynamic[66]. During ORCs in buffer, the SERS intensity and background increase each cycle, implying further surface roughening occurs (**Supplementary Figure 19a-c**). We also observe the appearance of transient SERS peaks, indicating the surface undergoes dynamic structural changes at the nanoscale. In contrast, introducing CB[5] and Cl^- during cycling stabilises the SERS signal, with consistent backgrounds and no appearance of transient SERS features (**Supplementary Figure 19d-f**). The CB[5], Au oxide, Au-Cl, and Au(III)-Cl SERS signals are now repeatable each oxidation-reduction cycle. This stabilisation of the SERS suggests that CB[5] also acts as a structural stabiliser, ensuring that dynamic restructuring inherent in electrochemical cycling is minimized. This leads to a consistently enhanced SERS signal and a stable background, supporting reproducible SERS performance, however with weaker signals than the MLagg.

These results extend the applicability of CB[5]-mediated structural stabilisation beyond well-defined nanogap substrates. In both salt-aggregated and EC-roughened Au substrates, addition of CB[5] and Cl^- leads to improved reproducibility of SERS signals under electrochemical cycling. This highlights CB[5] as a versatile molecular stabiliser capable of mitigating nanoscale instabilities across structurally diverse Au SERS platforms.

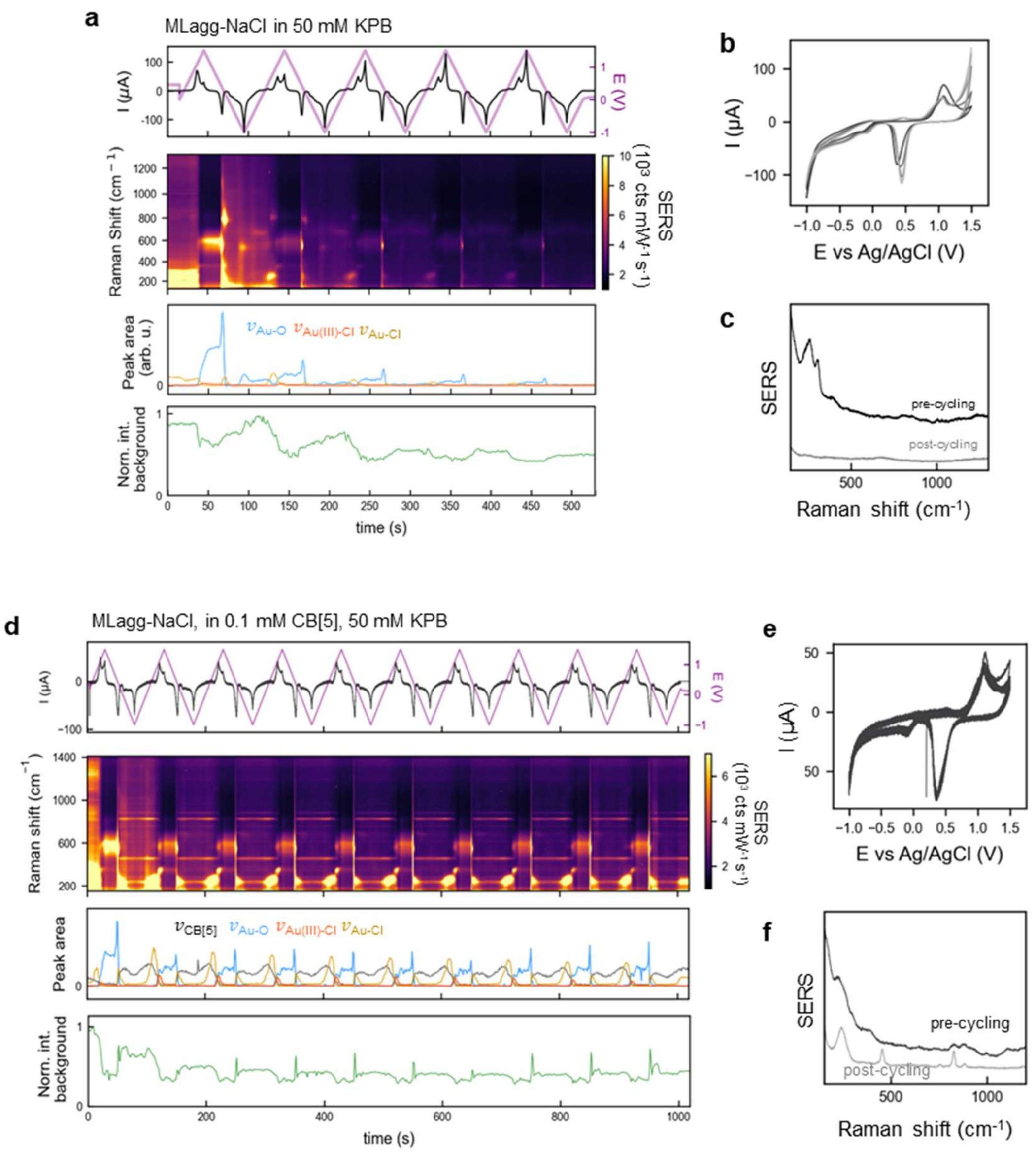

Supplementary Figure 18 | Cyclic voltammetry (CV) and time-series SERS of MLagg-NaCl in potassium phosphate buffer (KPB, pH 7.0) with and without CB[5]. (**a,d**) Applied potential vs Ag/AgCl (purple solid line) and corresponding current response (black solid line) from a MLagg-CB[5] cycled between +1.5 V and -1.0 V in (**a**) 50 mM KPB (pH 7.0) or (**d**) 0.1 mM CB[5] in 50 mM KPB at 50 mV s^{-1} for 5 scans. Time-series SERS spectra during the potential scans is shown, along with the peak areas of CB[5] (826 cm^{-1}, black), Au-O (summation of ~520, ~590, and ~660 cm^{-1} peaks, line), Au(III)-Cl (~345 cm^{-1}, orange), and Au-Cl (~240-270 cm^{-1}, yellow) per SERS spectrum. Bottom panel plots integrated background intensity per spectrum. (**b,e**) Overlaid cyclic voltammograms of the five scans in (**b**) 50 mM KPB or (**e**) 0.1 mM CB[5] in 50 mM KPB. (**c, f**) SERS spectra of the MLagg-CB[5] before and after cycling in (**c**) 50 mM KPB, or (**f**) 0.1 mM CB[5] in 50 mM KPB.

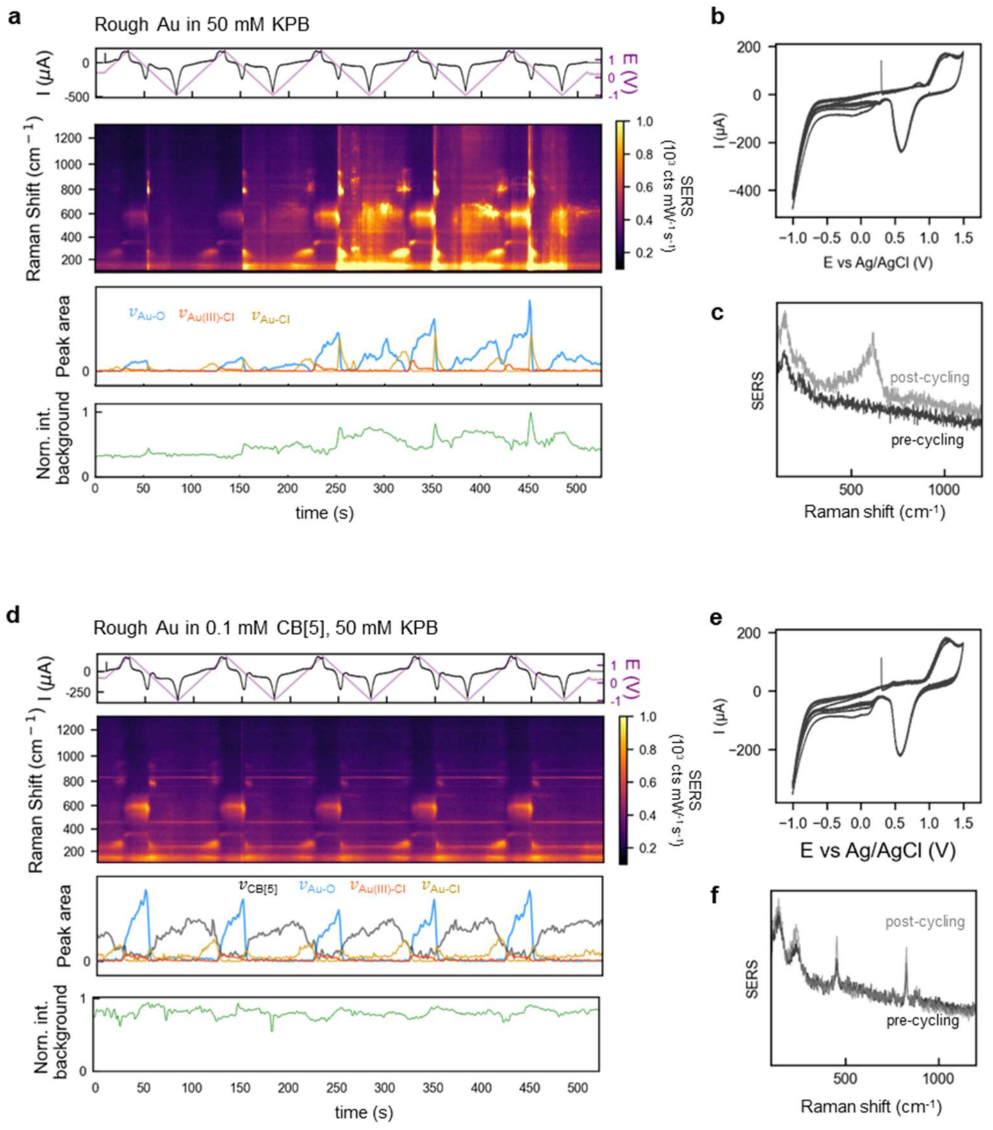

Supplementary Figure 19 | Cyclic voltammetry (CV) and time-series SERS of EC-roughened Au electrode in potassium phosphate buffer (KPB, pH 7.0) with and without CB[5]. (**a,d**) Applied potential vs Ag/AgCl (purple solid line) and corresponding current response (black solid line) from a MLagg-CB[5] cycled between +1.5 V and -1.0 V in (a) 50 mM KPB (pH 7.0) or (d) 0.1 mM CB[5] in 50 mM KPB at 50 mV s^{-1} for 5 scans. Time-series SERS spectra during the potential scans is shown, along with the peak areas of CB[5] (826 cm^{-1}, black), Au-O (summation of ~520, ~590, and ~660 cm^{-1} peaks, blue), Au(III)-Cl (~345 cm^{-1}, orange), and Au-Cl (~240-270 cm^{-1}, yellow) per SERS spectrum. Bottom panel plots integrated background intensity per spectrum. (**b,e**) Overlaid cyclic voltammograms of the five scans in (**b**) 50 mM KPB or (**e**) 0.1 mM CB[5] in 50 mM KPB. (**c, f**) SERS spectra of the MLagg-CB[5] before and after cycling in (**c**) 50 mM KPB, or (**f**) 0.1 mM CB[5] in 50 mM KPB.

Supplementary Note 8. EC-ReSERS Kinetics

To uncover the requirements for effective nanogap re-scaffolding in EC-ReSERS, and to define conditions suitable for practical regeneration cycles, we systematically investigated how electrochemical kinetics influence surface redox transformations and nanogap regeneration. Specifically, we explored how variations in scan rate and buffer concentration affect the formation of key surface intermediates, and their relationship to CB[5] nanogap rebinding following oxidative cleaning. By combining cyclic voltammetry with time-resolved SERS, we identified how mass-transfer limitations and electric double layer (EDL) structure modulate both the redox sequence and the electrostatic environment experienced by CB[5]. These studies reveal trade-offs between redox efficiency and nanogap accessibility, offering insight into how electrochemical parameters influence the success or failure of re-scaffolding. The results guide both mechanistic interpretation and the design of EC-ReSERS protocols for reproducible, high-performance sensing.

Scan rate

The effect of scan rate (v) on MLagg oxidation and reduction kinetics is investigated using cyclic voltammetry (CV) and time-resolved SERS (**Extended Data Figure 3a**). The electrochemical behaviour when cycling in 0.1 mM CB[5] and 50 mM potassium phosphate buffer (pH 7.0) indicates that both oxidation and reduction are mass-transfer limited, as evidenced by the increasing peak current with $v^{1/2}$ (Randles-Sevcik behaviour, **Extended Data Figure 3b,c**), the shift in oxidation (V_o) and reduction (V_{or}) peak potentials (**Extended Data Figure 3d,e**) to more extreme values with increasing v, and the broadening of the peaks with $\log v$ (**Extended Data Figure 3f,g**). The oxidation process is highly dependent on the local pH and Cl^- concentration, as surface water must be deprotonated to form Au-O species, while Cl^- is required for the formation of Au-Cl and Au(III)-Cl intermediates (see **Supplementary Note 5, eqns. 1-8** for chemical reactions). The increasing potential shifts with v confirm that reactant depletion at the electrode surface constrains oxidation kinetics at higher sweep rates, thereby requiring higher overpotentials. Similarly, reduction kinetics are also limited by reactant diffusion, as evident from the increased overpotentials required for Au oxide reduction.

Oxidation: SERS analysis further reveals that the evolution of intermediate species is strongly v-dependent (**Extended Data Figure 3h-j**). During oxidation, a slow scan rate (5 $mV\cdot s^{-1}$) allows the simultaneous rise of Au(III)-Cl and Au-O, followed by a 95% decrease in Au(III)-Cl intensity and conversion to predominantly Au-O (**Extended Data Figure 3h**). At an intermediate scan rate (50 $mV\cdot s^{-1}$), Au(III)-Cl and Au-O also form nearly simultaneously, but Au(III)-Cl persists more prominently, demonstrating only a 70% decrease in SERS intensity while further oxidation to Au-O occurs (**Extended Data Figure 3i**). At the highest scan rate (500 $mV\cdot s^{-1}$), Au(III)-Cl is even more persistent, with only a 35% decrease in intensity, indicating that the limited time for Cl^- displacement by OH^- prevents complete Au oxide formation (**Extended Data Figure 3j**). This trend is consistent with the anodic shift of the oxidation onset potential (V_o), which parallels the shift in peak Au(III)-Cl SERS intensity toward more positive potentials at higher scan rates. These results indicate that at high scan rates, the transformation of Au(III)-Cl to Au-O is incomplete due to insufficient time for reactant exchange, leading to the persistence of Au(III)-Cl species in the nanogap.

At higher scan rates, we also observe a lag between electrochemical current peaks and the corresponding SERS signals, indicating delayed transformations within the nanogaps. This likely reflects more pronounced mass transport limitations into these recessed regions, which are less accessible than the exposed AuNP surfaces probed by CV. This highlights that electrochemical measurements reflect the entire electrode, while SERS selectively reports on the nanogap environment.

Reduction: Mass transfer limitations can also be observed during reduction. During the cathodic sweep, Au(III)-Cl reduction precedes the complete reduction of Au-O. As Au(III)-Cl intensity decreases, there is a corresponding increase in Au-Cl and the rebinding of CB[5] molecules to the nanogaps. At slow scan rates, Au-Cl SERS intensities recover rapidly with a steep rise in CB[5] intensity that surpasses pre-oxidation levels, suggesting that extended diffusion time allows for both enhanced Au-Cl formation and CB[5] re-scaffolding. The slow scan rate also enables more complete Au-O reduction as Au-Cl is formed, which can ensure a favourable CB[5] re-binding environment at the nanogap. At this scan rate, the CB[5] intensity reaches a plateau at 0.25 V, indicating that full rebinding occurs early in the reduction cycle (**Extended Data Figure 3h**, see grey vertical line). At 50 $mV\cdot s^{-1}$, the Au-Cl

intensity during reduction is lower, and the CB[5] signal plateaus at a greater overpotential (~0 V), suggesting that mass-transfer constrains the re-scaffolding process (**Extended Data Figure 3i**). At the highest scan rate, Au-Cl formation is even more limited, and the CB[5] signal reaches a plateau only at -0.50 V, confirming that high scan rates exacerbate reactant depletion effects (**Extended Data Figure 3j**). Notably, a 25% decrease in CB[5] intensity is observed after the first cycle at this scan rate, but subsequent cycles maintain a stable CB[5] signal, suggesting that despite reduced ligand rebinding, a minimum threshold of CB[5] molecules can sufficiently rebind, ensuring nanogap stability.

Effect of CB[5] concentration: For practical applications, the ability to perform EC-ReSERS rapidly is highly desirable, particularly for SERS substrate regeneration in sensing applications. To evaluate whether a faster electrochemical switching process could be implemented, oxidation and reduction were performed using alternating stepped potentials (+1.5 V for oxidation, followed by -0.8 V for reduction) under identical solution conditions (0.1 mM CB[5] in 50 mM buffer). Under these conditions, the SERS signal decreased over repeated cycles, suggesting nanogap sintering (**Supplementary Figure 20a**). This indicates that the oxidation and reduction processes were too rapid for full reactant diffusion and CB[5] rebinding, reinforcing the role of mass-transfer limitations in dictating the optimal cycling conditions.

However, increasing the CB[5] concentration to 1 mM CB[5] during the reduction step allowed the stepped potential process to proceed without significant signal loss across multiple cycles (**Supplementary Figure 20b**), suggesting that higher CB[5] concentrations provide a more efficient means of stabilizing nanogaps, compensating for limited diffusion time at faster potential switching. This further confirms that at lower CB[5] concentrations, mass-transfer constraints hinder effective ligand rebinding during rapid potential stepping, leading to nanostructural destabilization. Thus, while EC-ReSERS at high scan rates or stepped potentials is feasible, it requires conditions that mitigate mass-transfer limitations, either through increased CB[5] availability or possible hydrodynamic conditions. These findings provide a critical framework for optimizing EC-ReSERS for practical applications, balancing cycling speed and substrate stability.

<u>Buffer concentration</u>

The effect of scan rate demonstrates that mass-transfer limitations influence EC-ReSERS kinetics. While cycling at 50 $mV\cdot s^{-1}$ provides a balance between speed and substrate stability, mass transport constraints are still evident. To further investigate these limitations, the impact of buffer concentration on the kinetics and efficiency of EC-ReSERS was investigated by varying the phosphate buffer concentration from 5 mM to 1000 mM, while maintaining a fixed CB[5] (0.1 mM) and Cl^- concentration (~0.5 mM) (**Supplementary Figure 21**). The variation in phosphate concentration alters buffering capacity, ionic strength, and EDL properties, thereby affecting both oxidation and reduction processes.

Oxidation: Electrochemical data indicate that higher buffer concentrations reduce mass-transfer limitations, as evidenced by increased peak currents (i_p), lower overpotentials (V_o and V_{or}), and narrower FWHM for oxidation and reduction peaks (**Supplementary Figure 21a-g**). These improvements in electrochemical kinetics at higher buffer concentrations can be due to the enhanced ion availability and transport. *In situ* SERS data further support this (**Extended Data Figure 5**), showing that during the anodic sweep, adsorption of additional anions (OH^- and PO_4^{3-}) follows Au-Cl formation, with significantly higher SERS intensities for OH^- and PO_4^{3-} at elevated buffer concentrations (**Extended Data Figure 5g,h**). This indicates that at higher ionic strength, the EDL is more compressed (Debye length ~0.3 nm), allowing for greater incorporation and accessibility of multiple anions at the nanogaps. In contrast, at lower buffer concentrations (Debye length ~1-4 nm), Au-Cl adlayer formation dominates, likely due to both the high affinity of Cl^- for Au and the increased EDL overlap at the 0.9 nm nanogaps, which limits the availability of competing anions in the double layer.

Reduction: Reduction also showed strong mass-transfer limitations. At low buffer concentration (5 mM), the reduction peaks for Au(III)-Cl and Au-O are broad and well-separated, indicating strong mass-transfer limitations that cause these processes to occur sequentially (**Extended Data Figure 5a**). In particular, the reduction of Au-O requires protons to facilitate oxide removal, and at low buffer concentrations, the limited proton availability and diffusion further constrain Au-O reduction, delaying its occurrence to more negative potentials. At moderate buffer concentrations (50 mM), a single broad reduction peak is observed, likely due to the merging of the two

reduction steps as mass transport improves but reactant replenishment remains constrained, leading to kinetic overlap (**Extended Data Figure 5d**). In contrast, at high buffer concentrations, two narrow but closely spaced peaks appear, suggesting that mass-transfer limitations are minimized, allowing Au(III)-Cl and Au-O reduction to occur as distinct, rapid processes within a smaller potential window (**Extended Data Figure 5g**). These trends indicate that buffer concentration strongly influences the resolution and broadening of reduction peaks, with lower concentrations leading to sequential, diffusion-limited reduction, while higher concentrations enhance ion transport, enabling sharper, well-defined reduction processes.

CB[5] re-scaffolding*:* Following the reduction of Au(III)-Cl, the formation of Au-Cl and the subsequent rebinding of CB[5] are strongly influenced by buffer concentration, particularly due to EDL effects and competing anion adsorption. At high buffer concentrations, time-resolved SERS shows that as Au-Cl is formed, OH^- and PO_4^{3-} also rebind at the electrode surface (**Extended Data Figure 5g,i**). The compressed EDL in these conditions allows a greater accumulation of these highly charged anions near the surface, introducing stronger electrostatic repulsion, and thereby screening CB[5] from efficiently re-entering the nanogap. The additional anions can also screen the nearby electropositive Au atoms generated from the Au-Cl surface dipole, therefore blocking CB[5] rebinding. As a result, the CB[5] signal after reduction is lower compared to pre-oxidation levels, and with further cycling, this inefficient rebinding leads to a 70% decrease in CB[5] SERS intensity (**j**), most likely due to nanogap sintering.

In contrast, at lower buffer concentrations (5 and 50 mM), significantly less OH^- and PO_4^{3-} rebinding is observed following Au(III)-Cl reduction (**Extended Data Figure 5a,d,c,f**). Instead, due to EDL overlap effects, Au-Cl dominates the surface, providing an environment that is more favourable for CB[5] rebinding. Unlike “hard” anions OH^- and PO_4^{3-}, which introduce strong electrostatic repulsion and charge screening, the Au-Cl adlayer exhibits partial covalent character, reducing the overall surface charge density and making it less repulsive to adsorbate binding. This allows CB[5] to interact more easily with Au-Cl, facilitating efficient rebinding and nanogap reconstruction, thereby maintaining stable SERS signals across multiple cycles. These results highlight that while higher buffer concentrations improve oxidation and reduction kinetics, they can also negatively impact CB[5] rebinding by suppressing favourable Au-Cl interactions and promoting ionic screening effects, ultimately leading to nanogap destabilization and eventual sintering over extended cycling.

High buffer concentration, slow scan rate

To further probe the role of anionic screening in nanogap re-scaffolding, we conducted EC-ReSERS at a slow scan rate (5 $mV\cdot s^{-1}$) in 1 M KPB (**Extended Data Figure 6**). These conditions minimize mass transport limitations and allow CB[5] sufficient time to diffuse to the interface. Despite this, we observed that CB[5] rebinding remained significantly delayed, initiating only after both phosphate and OH^- SERS signals disappeared. Notably, Au-Cl formation is still evident, but now coexisted with stronger and more persistent phosphate signatures compared to faster scan rates. These findings indicate that excess anionic species in the nanogap impede effective CB[5] rebinding not due to transport limitations, but due to an electrostatically unfavourable interfacial environment dominated by competing adsorbates. In contrast, when the surface is primarily covered by Au-Cl, the nanogap environment becomes more conducive to CB[5] re-entry, as the outward-oriented dipole and reduced anion crowding promote favourable dipole-dipole interactions. Thus, successful nanogap regeneration is governed not just by adsorption kinetics, but by the precise electrostatic and chemical composition of the nanogap interface.

Summary

The electrochemical and SERS data demonstrate that both scan rate and buffer concentration play crucial roles in the efficiency and stability of EC-ReSERS cycling. While higher scan rates accelerate oxidation and reduction, they impose mass-transfer limitations that hinder CB[5] rebinding. Similarly, higher buffer concentrations enhance ion transport and mitigate mass-transfer constraints but lead to stronger EDL compression, promoting OH^- and PO_4^{3-} adsorption at the nanogaps, which electrostatically screens CB[5] and contributes to nanogap sintering. Even when diffusion limitations are removed, CB[5] rebinding remains delayed until excess anions desorb from the nanogap. This confirms that it is not diffusion-limited transport, but the unfavourable local interfacial environment that restricts effective re-scaffolding. In contrast, when the surface is predominantly covered by Au-Cl and competing anions are minimized, the nanogap environment becomes electrostatically favourable, facilitating CB[5] rebinding via dipole-dipole interactions. In contrast, moderate conditions (50 $mV\cdot s^{-}$

[1] scan rate and 50 mM buffer) provide a balance between efficient oxidation/reduction and effective CB[5] re-scaffolding, leading to stable cycling performance. These insights highlight the trade-offs between reaction kinetics and nanogap stability in EC-ReSERS, underscoring the need for optimized electrolyte composition and cycling conditions to maintain reproducibility and enhance long-term substrate performance for sensing applications.

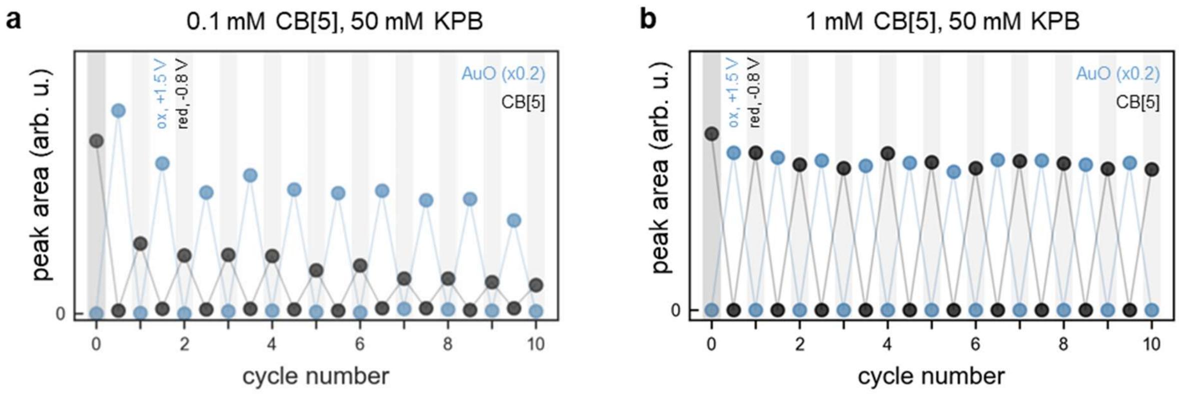

Supplementary Figure 20 | EC-ReSERS with oxidative and reductive step potentials. (**a,b**) Peak areas of Au oxide and CB[5] per cycle of MLagg-CB[5] oxidation (+1.5 V, 15 s) and reduction (-0.80 V, 15 s) in (**a**) 0.1 mM CB[5] and (**b**) 1 mM CB[5] in 50 mM potassium phosphate buffer (pH 7.0).

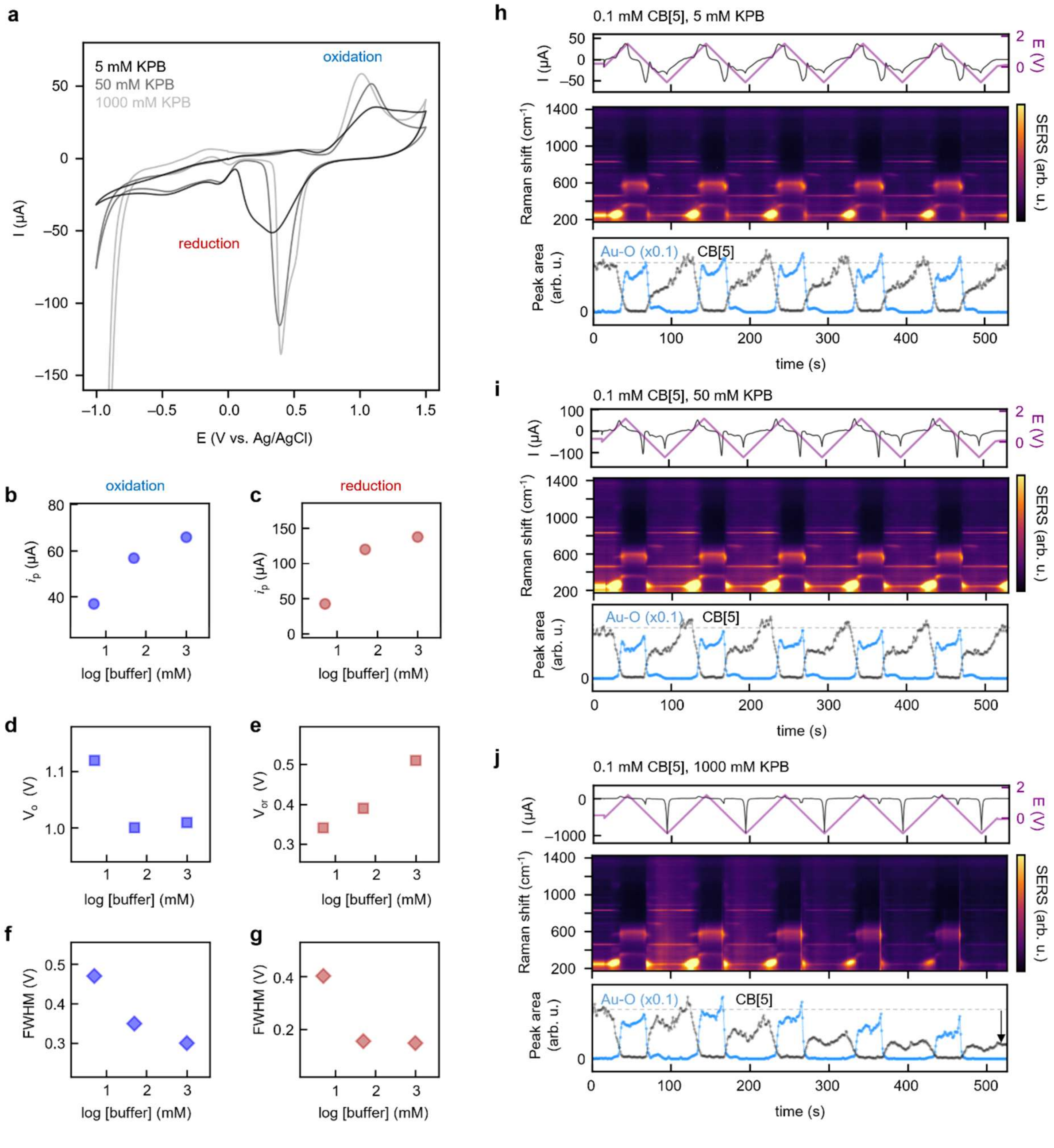

Supplementary Figure 21 | EC-ReSERS kinetics at different buffer concentrations. (**a**) Overlaid cyclic voltammograms of MLagg-CB[5] in 0.1 mM CB[5] and varying buffer concentrations = 5, 50, 1000 mM potassium phosphate buffer (KPB) at pH 7.0. Scan rate = 50 mV·s^{-1}. Effect of scan rate on the oxidation and reduction (**b-c**) peak currents (i_p), (**d-e**) peak potentials and (**f-g**) peak widths (full-width half-maximum, FWHM). Applied potential *vs* Ag/AgCl (purple solid line) and corresponding current response (black solid line) from MLagg-CB[5] cycled between +1.5 V and -1.0 V in 0.1 mM CB[5] and (**h**) 5 mM, (**i**) 50 mM, and (**j**) 1000 mM KPB. Time-series SERS spectra during the potential scans is shown, along with the peak areas of Au-O (summation of 520, 590, and 660 cm^{-1} peaks, blue), and CB[5] (826 cm^{-1}, grey) per SERS spectrum.

Supplementary Note 9. EC-ReSERS in varying electrolytes

To investigate the effect of the supporting electrolyte on the oxidation and reduction of MLagg-CB[5], we performed time-resolved SERS and cyclic voltammetry in different electrolytes with and without CB[5]. EC-ReSERS has been demonstrated previously using potassium phosphate buffer, pH 7.0. Changing the cation to Na^+ by using sodium phosphate buffer (pH 7.0) did not reveal any significant changes in the voltammogram nor in the SERS spectra (**Supplementary Figure 22**). Similarly, changing the anion to sulphate (**Supplementary Figure 23**) or perchlorate (**Supplementary Figure 24**) did not significantly change the nanogap chemistry, although there are some minor spectral and electrochemical differences that can be noted. Previous work studying the oxidation of Au in different electrolytes has shown that as the applied potential is swept anodically, hydroxide ions from the aqueous solvent compete for Au binding sites with the electrolyte anions as the initial step towards Au oxidation[51]. As anions have different binding affinities to Au (in order of $OH^- > Cl^- > SO_4^{2-} > ClO_4^-$)[67], the small shifts in the Au oxidation potential (V_o) are expected.

The cyclic voltammograms in phosphate buffer are also more stable compared to those in Na_2SO_4 and $NaClO_4$, suggesting that there may be fluctuations in local pH when using non-buffer electrolytes, resulting in more prominent shifts in the oxidation and reduction potentials per cycle. In the non-buffered electrolytes, there are also differences in the shape of the Au oxide reduction peak (V_{or}). For phosphate buffer, the reduction peak appears as a single asymmetric peak at +0.4 V, but in Na_2SO_4 and $NaClO_4$, the reduction peak splits into two: the α-oxide peak at higher potentials followed by the β-oxide peak at lower potentials[68,69]. The kinetics of oxidation in the non-buffered electrolytes might favour the sequential formation of different oxide species. Also, the α-/β-oxide splitting is more evident when cycling without CB[5], suggesting that the presence CB[5] and/or small amounts of Cl^- can interfere with the formation of the more stable β-oxide.

The effect of pH was also investigated. Cycling in low pH with different anions generally show similar behaviour. In all cases, when cycling with 0.1 mM CB[5] in acidic conditions (**Supplementary Figure 25-Supplementary Figure 27**), CB[5] lines decrease in intensity after the first cycle but are relatively stable afterwards. The oxidation potential in low pH also shifts to higher potentials compared to that in neutral conditions, which is expected given the low concentration of hydroxide ions at low pH. There also appears to be a higher tendency to form Au(III)-Cl species based on the stronger Au(III)-Cl lines relative to Au-O lines.

Cycling in high pH (100 mM NaOH, pH 13), on the other hand, shows a preference for the formation of Au-O/H compared to Au-Cl species (**Extended Data Figure 4**). In fact, the Au oxidation potential is shifted to lower potentials (~0.5 V compared to 1.0 V in neutral conditions and 1.3 V in acidic conditions) and there is no trace of Au-Cl species from the SERS spectra. The high concentration of OH^- in the electrolyte and the comparably high affinity of OH^- to Au effectively displaces any Cl^- from the surface, resulting in the oxidation and reduction of Au without the formation of any Au-Cl intermediates. Instead, a peak at 380-400 cm^{-1} corresponding to $Au\text{-}OH^-_{(ads)}$ appears at positive potentials, which readily transitions to the formation of Au oxide by +0.5 V. Cycling in these conditions, however, does not appear to be favourable for SERS, as there is a gradual decrease in SERS enhancement after Au oxide reduction even in the presence of CB[5]. Further discussion of the failure of effective re-scaffolding in NaOH is detailed in the **Main Text** and in **Supplementary Note. Electronic and interfacial properties of Au-Cl adlayer.**

In the other extreme, cycling in excess Cl^- was also examined by using NaCl (**Supplementary Figure 28**) and HCl (**Supplementary Figure 29**) as the supporting electrolytes. When Cl^- is in excess, anodic sweeps to +1.5 V result in the favourable formation of Au(III)-Cl species instead of Au oxide, as evidenced by the prominent Au-Cl and Au(III)-Cl SERS peaks and weak Au-O lines. As the electrolyte is not buffered, however, repeated cycling may lead to local pH changes promoting the formation of some Au-OH (550-560 cm^{-1}) by the second cycle. Interestingly, even when CB[5] is not added to the electrolyte, the existing CB[5] molecules on the MLagg appear to remain in the nanogaps despite the oxidation and reduction of Au(III)-Cl species. In this case, repeated oxidation and reduction appears to be highly repeatable as the nanogaps remain stabilised.

The behaviour in high Cl^- background electrolytes contrast sharply with systems of low $[Cl^-]$ where Au oxidation proceeds via Au-OH and Au-O formation, which readily displaces CB[5] from the surface. The absence of significant oxide formation in high Cl^- environments likely prevent this desorption, allowing CB[5] to remain bound throughout cycling. These findings support the hypothesis that CB[5] desorption is primarily driven by the formation of Au-O or Au-OH surface species, which form highly negatively charged environments at the nanogap and introduce repulsive interactions unfavourable for CB[5] carbonyl portal binding at the Au facets. On the contrary, Au-Cl adlayers, which exhibit partial covalent character and favourable interfacial properties, preserve CB[5] attachment and nanogap stability even in the absence of excess CB[5] molecules in solution.

Cycling in highly oxidising and reducing potentials in the presence of excess Cl^- is commonly used as a method to form roughened Au with high SERS activity[3], so to determine whether the retained SERS activity is due to roughening, a control using a MLagg-NaCl substrate was tested (**Supplementary Figure 30**). MLagg-NaCl is prepared from the same AuNPs, but is aggregated using NaCl, thus only native ligands such as citrate remain on the surface. When the MLagg-NaCl was cycled in NaCl, Au(III)-Cl was formed during the anodic sweep, followed by its reduction in the cathodic sweep. However, repeated cycling resulted in a gradual decrease in SERS activity, likely from the sintering of the nanogaps due to the lack of stabilising ligands. This result suggests that any increase in SERS activity from roughening is not enough to compensate for the decrease in SERS activity from sintering.

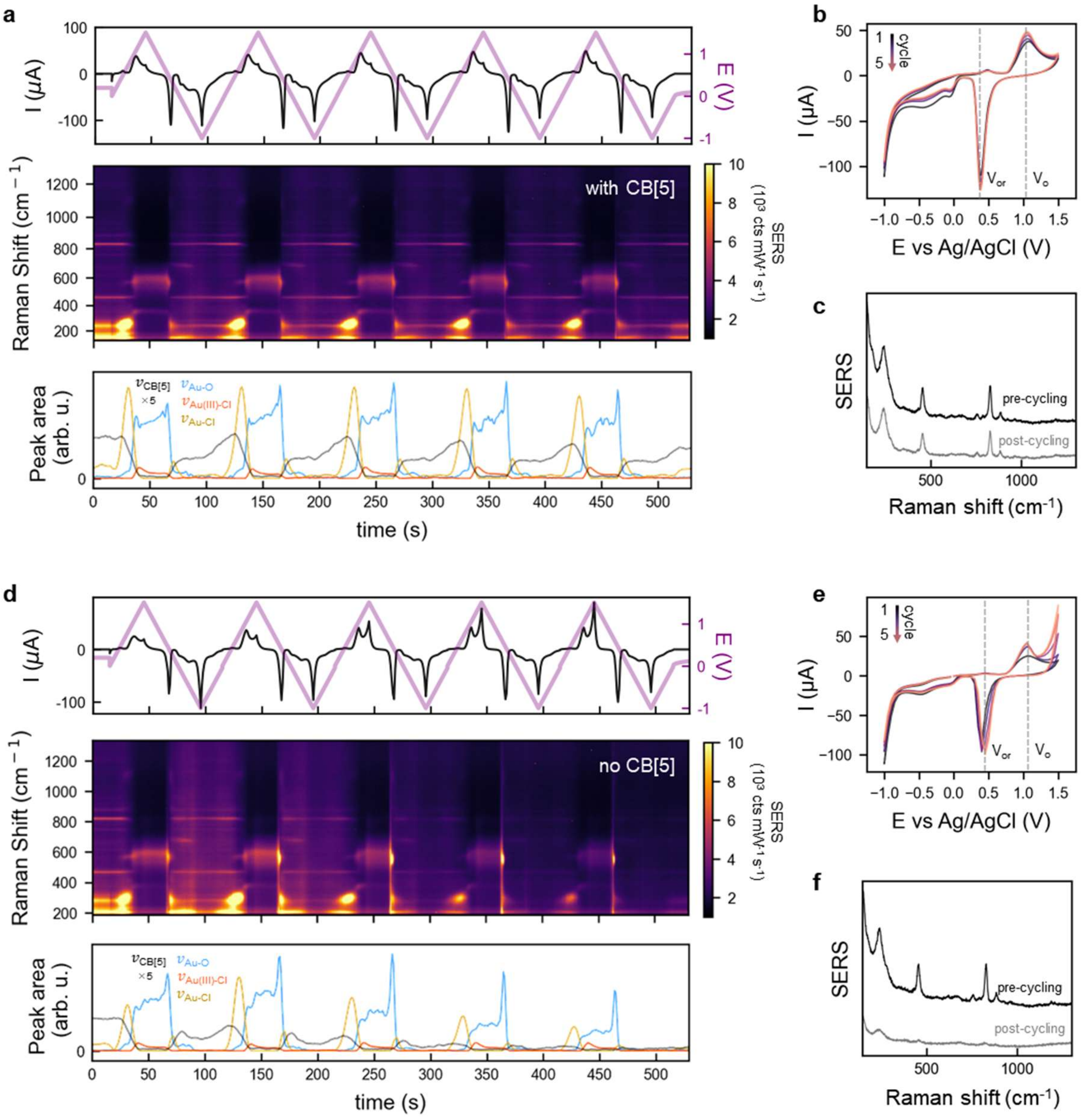

Supplementary Figure 22 | Cyclic voltammetry (CV) and time-series SERS of MLagg-CB[5] in sodium phosphate buffer (pH 7.0) with and without CB[5]. (**a,d**) Applied potential vs Ag/AgCl (purple) and current response (black) for MLagg-CB[5] cycled between +1.5 V and -1.0 V at 50 $mV \cdot s^{-1}$ for five scans, with (**a**) 0.1 mM CB[5] and (**d**) without CB[5], together with time-series SERS and extracted peak areas of CB[5] (826 cm^{-1}, grey) Au-O (520, 590, 660 cm^{-1}, blue), Au(III)-Cl (345 cm^{-1}, orange), and Au-Cl (240-270 cm^{-1}, yellow). (**b,e**) Overlaid cyclic voltammograms corresponding to (**a**) and (**d**). (**c,f**) SERS spectra of MLagg-CB[5] before and after cycling, measured (**c**) with and (**f**) without CB[5].

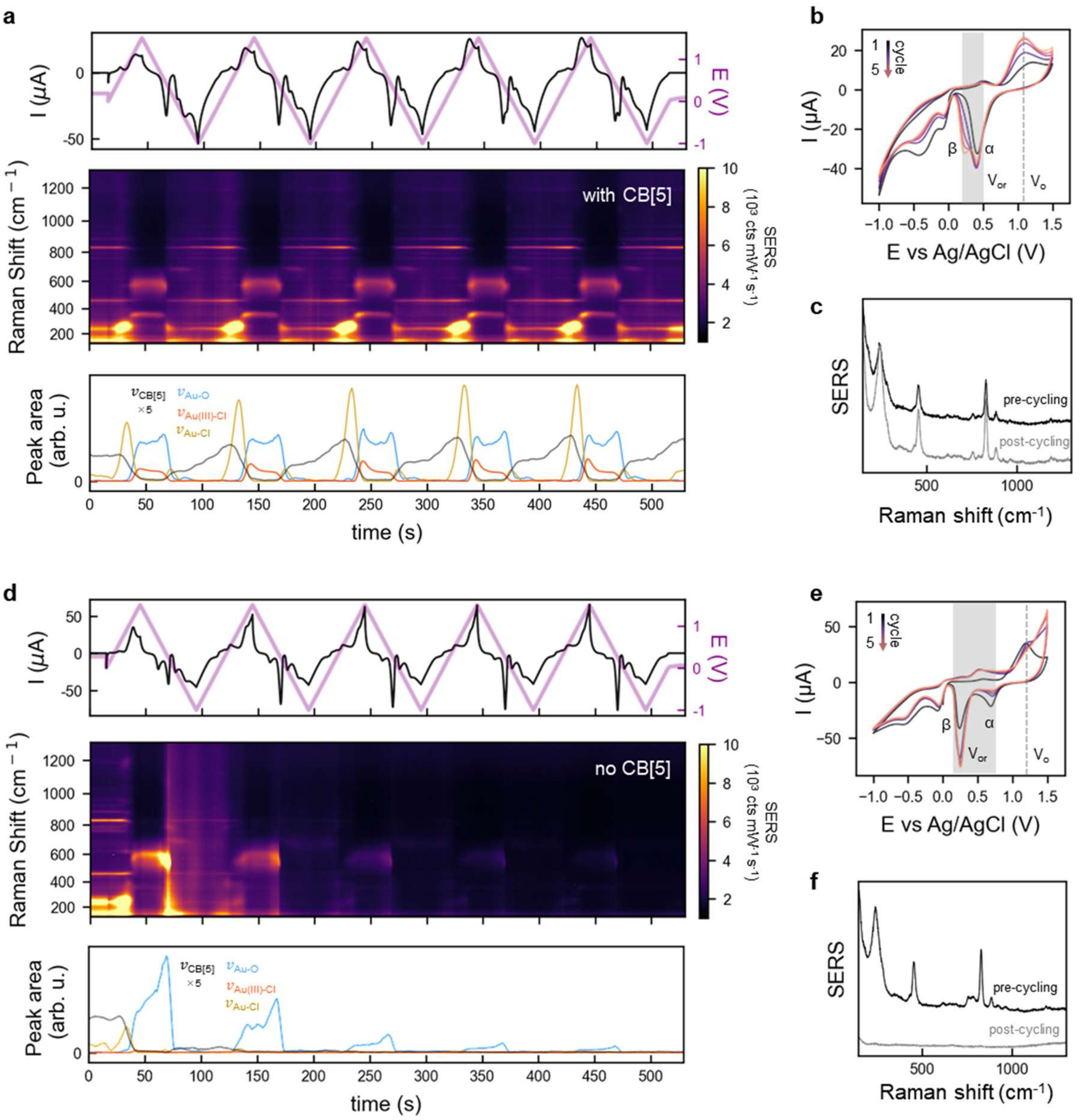

Supplementary Figure 23 | Cyclic voltammetry (CV) and time-series SERS of MLagg-CB[5] in 100 mM Na_2SO_4 (pH 7.0) with and without CB[5]. (**a,d**) Applied potential vs Ag/AgCl (purple) and current response (black) for MLagg-CB[5] cycled between +1.5 V and -1.0 V at 50 mV·s^{-1} for five scans, with (**a**) 0.1 mM CB[5] and (**d**) without CB[5], together with time-series SERS and extracted peak areas of CB[5] (826 cm^{-1}, grey) Au-O (520, 590, 660 cm^{-1}, blue), Au(III)-Cl (345 cm^{-1}, orange), and Au-Cl (240-270 cm^{-1}, yellow). (**b,e**) Overlaid cyclic voltammograms corresponding to (**a**) and (**d**). (**c,f**) SERS spectra of MLagg-CB[5] before and after cycling, measured (**c**) with and (**f**) without CB[5].

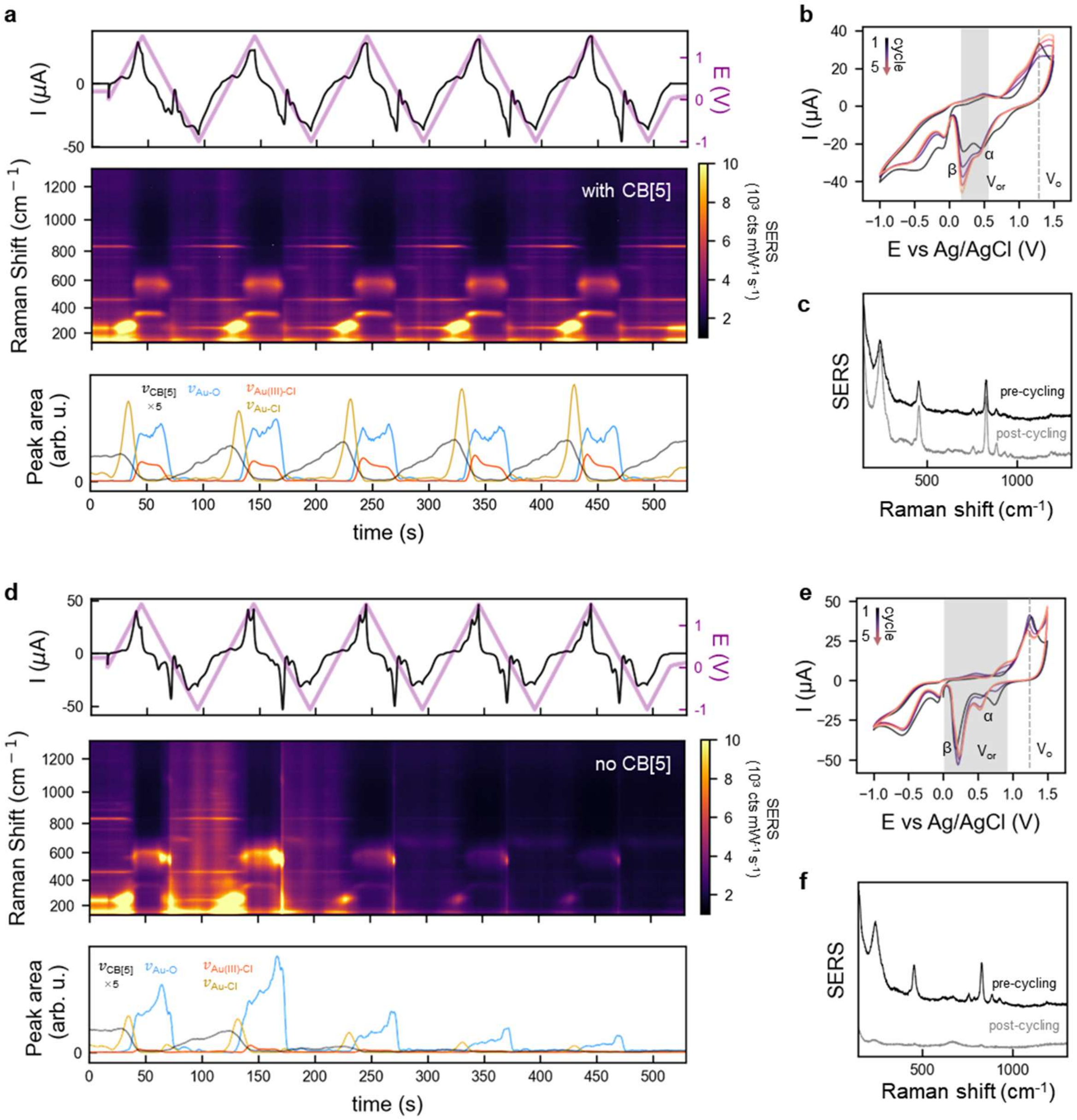

Supplementary Figure 24 | Cyclic voltammetry (CV) and time-series SERS of MLagg-CB[5] in 100 mM $NaClO_4$ (pH 7.0) with and without CB[5]. (**a,d**) Applied potential vs Ag/AgCl (purple) and current response (black) for MLagg-CB[5] cycled between +1.5 V and -1.0 V at 50 mV·s^{-1} for five scans, with (**a**) 0.1 mM CB[5] and (**d**) without CB[5], together with time-series SERS and extracted peak areas of CB[5] (826 cm^{-1}, grey) Au-O (520, 590, 660 cm^{-1}, blue), Au(III)-Cl (345 cm^{-1}, orange), and Au-Cl (240-270 cm^{-1}, yellow). (**b,e**) Overlaid cyclic voltammograms corresponding to (**a**) and (**d**). (**c,f**) SERS spectra of MLagg-CB[5] before and after cycling, measured (**c**) with and (**f**) without CB[5].

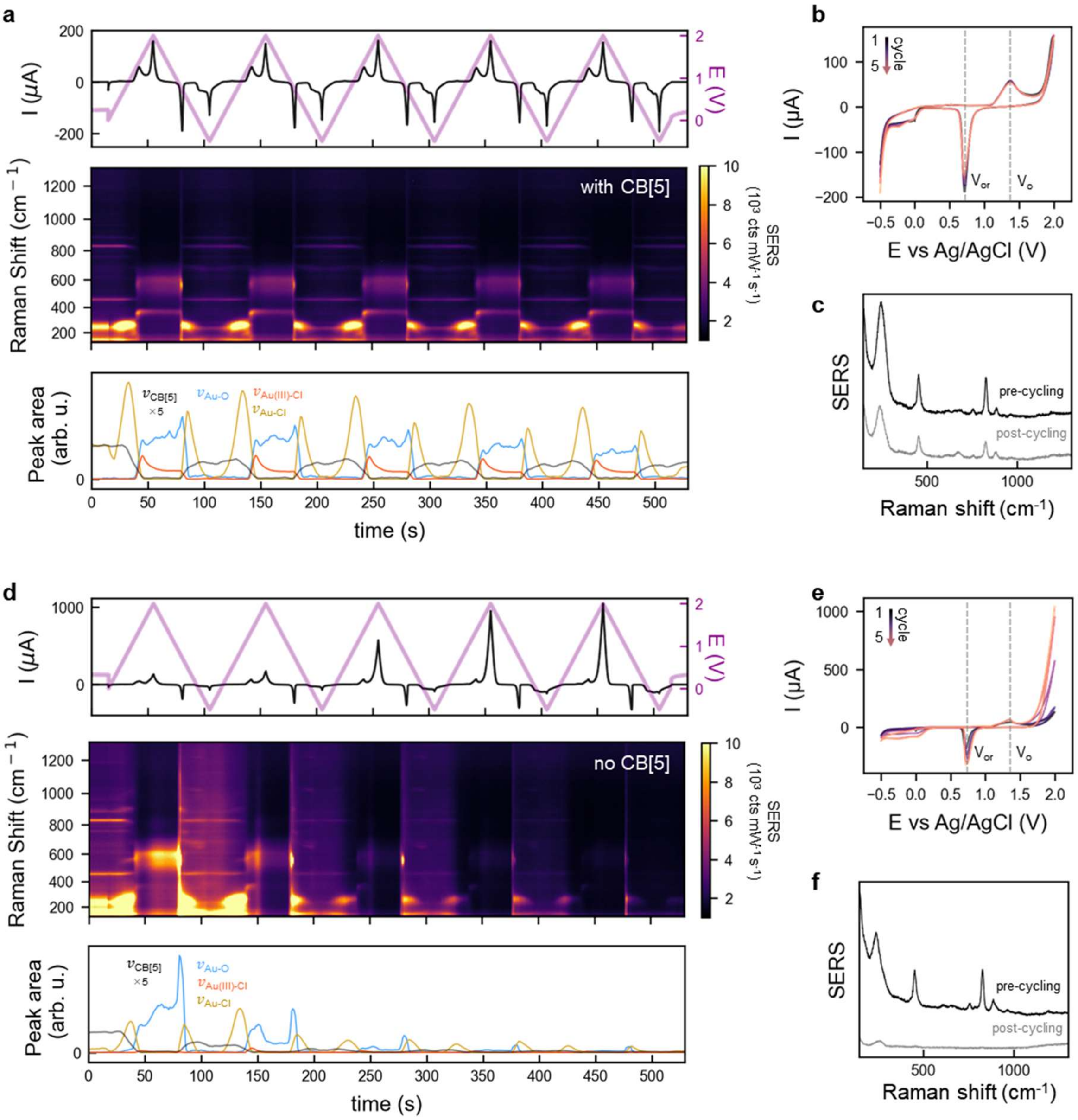

Supplementary Figure 25 | Cyclic voltammetry (CV) and time-series SERS of MLagg-CB[5] in 100 mM H_3PO_4 (pH 1.0) with and without CB[5]. (**a,d**) Applied potential vs Ag/AgCl (purple) and current response (black) for MLagg-CB[5] cycled between +1.5 V and -1.0 V at 50 mV·s^{-1} for five scans, with (**a**) 0.1 mM CB[5] and (**d**) without CB[5], together with time-series SERS and extracted peak areas of CB[5] (826 cm^{-1}, grey), Au-O (520, 590, 660 cm^{-1}, blue), Au(III)-Cl (345 cm^{-1}, orange), and Au-Cl (240-270 cm^{-1}, yellow). (**b,e**) Overlaid cyclic voltammograms corresponding to (**a**) and (**d**). (**c,f**) SERS spectra of MLagg-CB[5] before and after cycling, measured (**c**) with and (**f**) without CB[5].

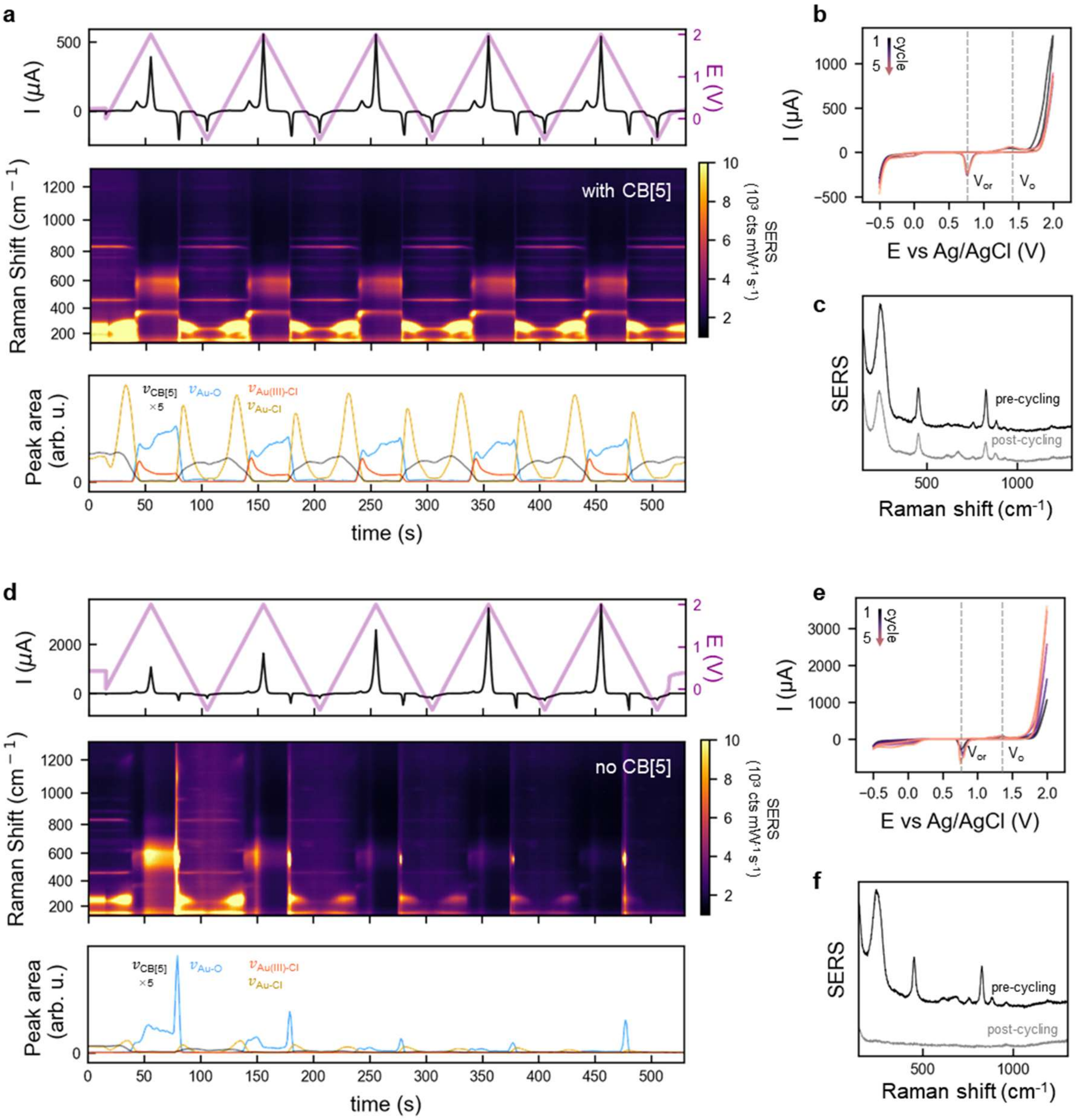

Supplementary Figure 26 | Cyclic voltammetry (CV) and time-series SERS of MLagg-CB[5] in 100 mM H_2SO_4 (pH 1.0) with and without CB[5]. (**a,d**) Applied potential vs Ag/AgCl (purple) and current response (black) for MLagg-CB[5] cycled between +1.5 V and -1.0 V at 50 mV·s^{-1} for five scans, with (**a**) 0.1 mM CB[5] and (**d**) without CB[5], together with time-series SERS and extracted peak areas of CB[5] (826 cm^{-1}, grey), Au-O (520, 590, 660 cm^{-1}, blue), Au(III)-Cl (345 cm^{-1}, orange), and Au-Cl (240-270 cm^{-1}, yellow). (**b,e**) Overlaid cyclic voltammograms corresponding to (**a**) and (**d**). (**c,f**) SERS spectra of MLagg-CB[5] before and after cycling, measured (**c**) with and (**f**) without CB[5].

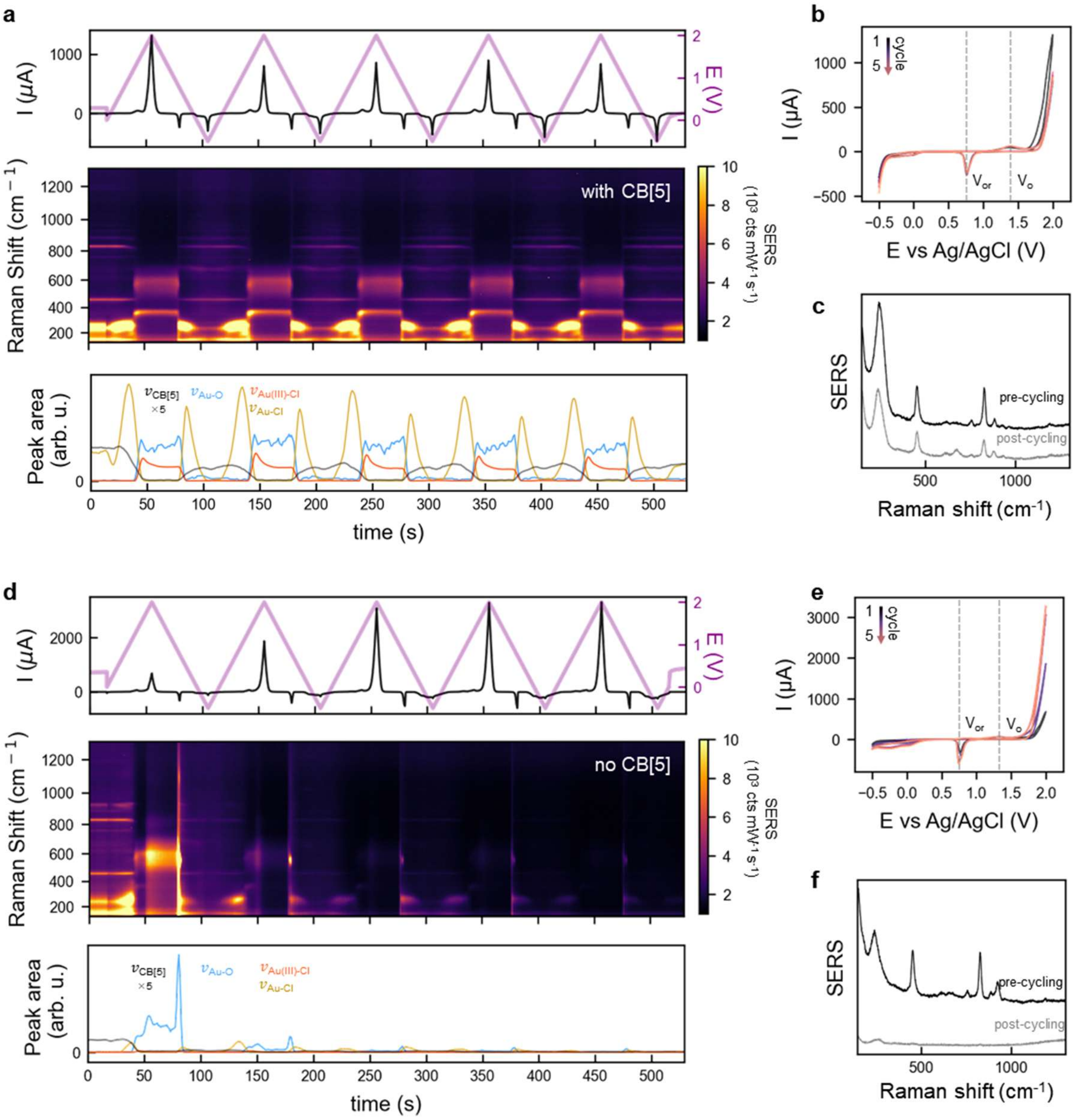

Supplementary Figure 27 | Cyclic voltammetry (CV) and time-series SERS of MLagg-CB[5] in 100 mM $HClO_4$ (pH 1.0) with and without CB[5]. (**a,d**) Applied potential vs Ag/AgCl (purple) and current response (black) for MLagg-CB[5] cycled between +1.5 V and –1.0 V at 50 mV·s^{-1} for five scans, with (**a**) 0.1 mM CB[5] and (**d**) without CB[5], together with time-series SERS and extracted peak areas of CB[5] (826 cm^{-1}, grey), Au–O (520, 590, 660 cm^{-1}, blue), Au(III)–Cl (345 cm^{-1}, orange), and Au–Cl (240–270 cm^{-1}, yellow). (**b,e**) Overlaid cyclic voltammograms corresponding to (**a**) and (**d**). (**c,f**) SERS spectra of MLagg-CB[5] before and after cycling, measured (**c**) with and (**f**) without CB[5].

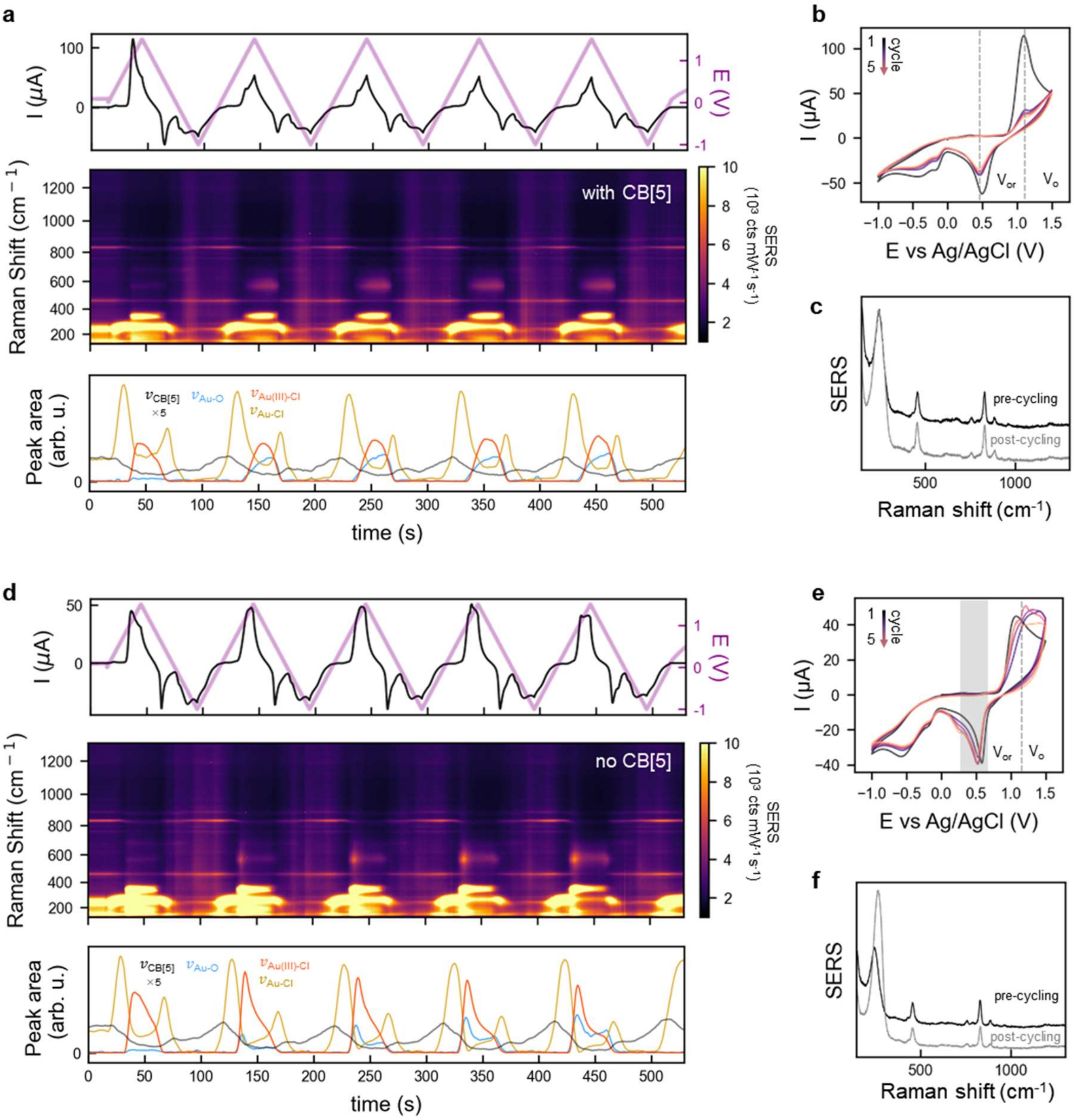

Supplementary Figure 28 | Cyclic voltammetry (CV) and time-series SERS of MLagg-CB[5] in 100 mM NaCl (pH 7.0) with and without CB[5]. (**a,d**) Applied potential vs Ag/AgCl (purple) and current response (black) for MLagg-CB[5] cycled between +1.5 V and –1.0 V at 50 mV·s^{-1} for five scans, with (**a**) 0.1 mM CB[5] and (**d**) without CB[5], together with time-series SERS and extracted peak areas of CB[5] (826 cm^{-1}, grey), Au–O (520, 590, 660 cm^{-1}, blue), Au(III)–Cl (345 cm^{-1}, orange), and Au–Cl (240–270 cm^{-1}, yellow). (**b,e**) Overlaid cyclic voltammograms corresponding to (**a**) and (**d**). (**c,f**) SERS spectra of MLagg-CB[5] before and after cycling, measured (**c**) with and (**f**) without CB[5].

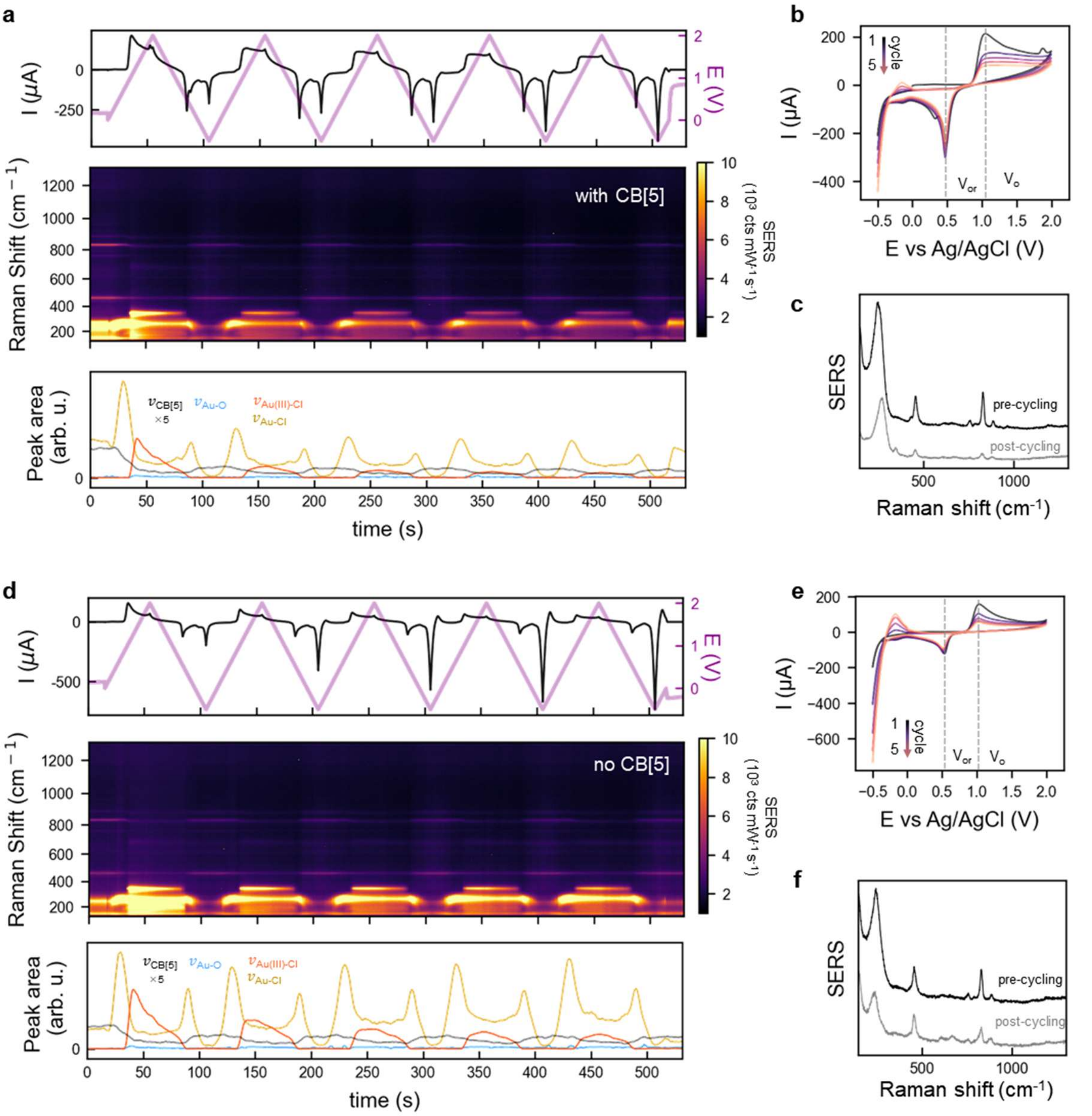

Supplementary Figure 29 | Cyclic voltammetry (CV) and time-series SERS of MLagg-CB[5] in 100 mM HCl (pH 1.0) with and without CB[5]. (**a**,**d**) Applied potential vs Ag/AgCl (purple) and current response (black) for MLagg-CB[5] cycled between +1.5 V and –1.0 V at 50 mV·s^{-1} for five scans, with (**a**) 0.1 mM CB[5] and (**d**) without CB[5], together with time-series SERS and extracted peak areas of CB[5] (826 cm^{-1}, grey), Au–O (520, 590, 660 cm^{-1}, blue), Au(III)–Cl (345 cm^{-1}, orange), and Au–Cl (240–270 cm^{-1}, yellow). (**b**,**e**) Overlaid cyclic voltammograms corresponding to (**a**) and (**d**). (**c**,**f**) SERS spectra of MLagg-CB[5] before and after cycling, measured (**c**) with and (**f**) without CB[5].

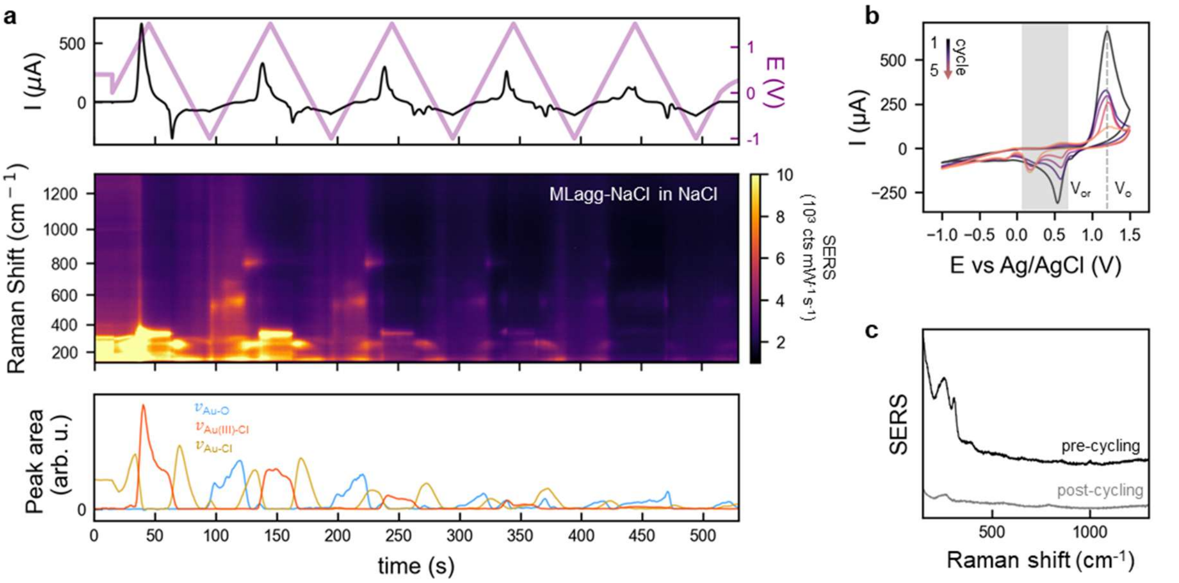

Supplementary Figure 30 | Cyclic voltammetry (CV) and time-series SERS of MLagg-NaCl in 100 mM NaCl (pH 7.0). (**a**) Applied potential vs Ag/AgCl (purple) and current response (black) for a MLagg-NaCl cycled between +1.5 V and -1.0 V at 50 mV·s^{-1} for five scans, together with time-series SERS and extracted peak areas of Au-O (520, 590, 660 cm^{-1}, blue), Au(III)-Cl (345 cm^{-1}, orange), and Au-Cl (240-270 cm^{-1}, yellow). (**b**) Overlaid cyclic voltammograms of the five scans in 100 mM NaCl. (**c**) SERS spectra of the MLagg-NaCl before and after cycling in 100 mM NaCl.

Supplementary Note 10. Electronic and interfacial properties of Au-Cl adlayer

The regeneration and stabilization of nanogap architectures in EC-ReSERS depend on the surface chemistry that emerges during electrochemical cycling. While Cl^- adsorption is a well-established phenomenon in electrochemistry, the precise role of Au-Cl surface species in modulating nanogap stabilisation during ReSERS remains poorly understood. Similarly, hydroxide adsorption offers a contrasting interfacial chemistry that may influence surface potential and electronic structure in distinct ways. To better understand why EC-ReSERS successfully regenerates nanogap hotspots under some conditions but not others, we investigate and characterize the formation of Au-Cl and Au-OH adlayers using complementary spectroelectrochemical methods. This comparison provides insight into how surface bonding, dipole formation, and interfacial structure can influence the re-scaffolding process and CB[5] rebinding during regeneration.

The characterisation of the Au-Cl adlayer is discussed in the **Main Text**. This section presents more in-depth discussion of results presented in the Main Text, as well as supporting experimental results.

Au-Cl adlayer

Au-Cl bond characterisation from literature. Although halogen-metal interactions are generally assumed to be ionic, as in the classical cases of Ag-Cl and Hg-Cl, electrochemical and surface science studies now demonstrate that Au-Cl bonding deviates from this norm, exhibiting significant covalent character[70]. While electrochemical studies show that the electrosorption valency of Cl^- on Au is close to 1, meaning nearly 1 e^- is transferred per Cl^- adsorbed, which is consistent with oxidation of surface Au(0) to Au(I)[71–73], the resulting Au-Cl bond exhibits a small dipole moment (~0.08 to 0.3 D)[67,71,73], far lower than predicted for ionic interactions. Early spectroscopic work by Gao and Weaver reported a well-defined Au-Cl stretch between 245-275 cm^{-1} with a high force constant (1.6×10^5 dyn cm^{-1}), indicating a bond more consistent with covalent than ionic character.[4] Collectively, these studies point to substantial charge redistribution at the interface, consistent with a polar covalent bond between Au and Cl, whether the bond is formed electrochemically from Cl^- adsorption on Au electrodes or from gaseous Cl_2 adsorption onto Au surfaces[70,71,74,75].

Au-Cl work function. The formation of the Au-Cl surface dipole is reflected electrochemically: when the Au-Cl layer is formed, the OCP stabilizes at +0.5 V, a shift from the initial value of 0 V. This shift reflects a change in the electronic potential at the electrode surface. Specifically, when an outward-pointing surface dipole forms ($Au^{\delta+} \rightarrow Cl^{\delta-}$), it raises the vacuum level and increases the work function, or the energy required to remove electrons from the metal.[76] Since the OCP reflects the difference in effective work function between the electrode and the reference electrode,[77] this increase manifests as a more positive OCP. This observation is consistent with early surface science studies using the Anderson retarding potential method, which reported a +0.5 V increase in work function upon Cl adsorption on Au(111), highlighting that both electrochemical (OCP) and vacuum-based methods yield comparable insights into the surface potential effects of Cl adsorption on Au.[78]

CO as a vibration probe of interfacial electric fields. In addition to open-circuit potential (OCP) measurements, we monitored the C≡O stretching mode of adsorbed carbon monoxide (CO) on the MLagg substrate to probe interfacial electric fields. The CO vibrational frequency is known to shift linearly with local electric field strength via the Stark effect[79], providing insight into surface potential variations under different conditions. Tracking the position of the CO band across different applied potentials and after relaxation to OCP allows us to distinguish between transient electrostatic effects arising from EDL charging and persistent fields associated with surface dipoles, such as those formed by Au-Cl.

<u>Origin of CO:</u> Although CO is not intentionally introduced in our experiments, we consistently observe a strong and well-defined CO stretching band (2120 cm^{-1})[79] in the SERS spectra of as-prepared MLagg-NaCl and MLagg-CB[5] samples, as well as in electrochemically-cycled MLagg-CB[5] (**Supplementary Figure 32**). The appearance of the peak in all samples implies that CO is not primarily generated during electrochemical ORCs, nor is it from the CB[5] reagent. The precise source is uncertain but likely arises from the ambient adsorption of trace CO from air during handling or sample preparation as adventitious carbon contamination[80]. Additional adsorbed CO may

also form during electrochemical oxidation of the citrate molecules that initially cap the AuNPs. Although the surface coverage of CO is presumed to be low, its peak appears prominently in the SERS spectrum due to the intrinsically large Raman cross section of the C≡O triple bond.

Stark tuning of CO under applied potential: The C≡O stretching mode exhibits a linear blue-shift with increasingly positive applied potential (**Extended Data Figure 8**). This behaviour is attributed to the vibrational Stark effect, where the energy of a polar bond is modulated by an external electric field[81]. For linearly adsorbed CO on metal surfaces, the applied field alters the electron density along the C≡O bond axis: a more positive interfacial potential pulls electron density away from the antibonding π* orbital, strengthening the bond and shifting the vibrational mode to higher wavenumbers[82]. We observed this field-dependent shift across three buffer concentrations (**Extended Data Figure 8c**). The CO stretching frequency shifts linearly with potential in each case in a manner consistent with those previously reported in the literature[79], but the magnitude of the tuning rate increases with ionic strength: 17 $cm^{-1}\cdot V^{-1}$ for 5 mM buffer, 19 $cm^{-1}\cdot V^{-1}$ for 50 mM, and 23 $cm^{-1}\cdot V^{-1}$ for 1000 mM buffer (all R^2=0.99). This trend reflects how ionic strength and EDL structure modulate the local electric field in the nanogap. At low buffer concentration, the EDL is diffuse and overlaps across the ~0.9 nm gap, producing a weaker field gradient at the adsorption site. As buffer concentration increases, the EDL compresses, and the potential drops more steeply near the metal surface, enhancing the local field and the corresponding Stark response of CO.

Tracking CO position after OCP relaxation: To distinguish transient EDL effects from persistent interfacial fields arising from chemical changes, we tracked CO frequency after stepping to a defined potential and relaxing to OCP (**Extended Data Figure 8b, Supplementary Figure 33**). Following negative potentials (-0.5 to -0.1 V), the CO peak relaxed to ~2120 cm^{-1}, indicating that EDL charging fields dissipate once external bias is removed. In contrast, following positive potentials (+0.1 to +0.5 V), the CO peak remained blue-shifted (2123-2131 cm^{-1}), with the magnitude of the shift increasing with the applied potential. These persistent shifts coincide with the appearance of Au-Cl vibrational features in SERS and a matching elevation in OCP, supporting the formation of a covalent Au-Cl surface dipole that modifies the interfacial electric field in the absence of applied bias.

Buffer concentration effects: While the formation of Au-Cl results in the same elevated OCP values across all buffer concentrations (e.g., +0.1 V, +0.3 V, and +0.5 V following applied steps of the same magnitude), the CO vibrational peak at OCP is offset by ~3 cm^{-1} in 1 M buffer relative to 5 and 50 mM (**Supplementary Figure 33**). This suggests that although the thermodynamic potential is dominated by the Au-Cl surface dipole, the local electric field at the CO adsorption site is further modulated by ionic strength. In high buffer concentrations, the compressed EDL shortens the distance over which the Au-Cl-induced surface potential drops, leading to a sharper potential gradient along the interface. This enhances the electric field experienced by CO, even though the overall surface potential (OCP) remains constant. Thus, the CO Stark shift at OCP reports not only the presence of chemically-induced fields (from Au-Cl), but also the electrostatic shaping of those fields by buffer ions. The observed increase in CO peak position in 1 M buffer reflects a cooperative effect: the Au-Cl adlayer establishes the interfacial dipole, while ionic screening compresses and amplifies the field in the region occupied by the CO molecule.

DF characterisation of Au-Cl formation. Dark-field (DF) scattering spectroscopy provides additional evidence of surface electronic restructuring upon Au-Cl adlayer formation (**Supplementary Figure 31a**). When Au-Cl forms, we observe a reversible ∼5% decrease in scattering intensity and a 5-7 nm redshift in the chain mode resonance. These spectral features are consistent with a modest increase in local refractive index and a decrease in free electron density at the Au surface. Similar optical signatures have been reported by Sannomiya *et al.*, who observed plasmon damping and redshifts in Au nanoparticles during electrochemical cycling in Cl^--containing electrolytes[83–85]. Simulations attributed these shifts to the formation of a “lossy layer” at the metal interface composed of electron-depleted Au and adsorbed Cl^-. This layer was shown to act as an absorbing dielectric interface that modifies the optical properties of the surface without significant morphological change. In our case, we now identify this surface species as the Au-Cl surface dipole. The localized depletion of electron density on surface-bound Au atoms and the polar nature of the Au-Cl bond support the formation of an interfacial layer with reduced metallicity. This manifests optically as a plasmon redshift and intensity damping. Notably, these DF

features are fully reversible, confirming that the observed changes stem from the electronic, not structural, transformation of the surface.

Control: buffer only. Control experiments performed in buffer only conditions (**Supplementary Figure 38a**), where only residual Cl^- is present, show markedly weaker vibrational and optical responses. The Au-Cl peak in SERS is less intense and remains near 240 cm^{-1} and gradually disappears with further potential switching. CO Stark shifts are also smaller, correlating with decreases in Au-Cl intensity. These controls confirm that the robust spectro-electrochemical features we observe result from electrochemically driven Au-Cl adlayer formation.

Au-Cl in EC-ReSERS

After characterising the structural and electronic properties of the Au-Cl adlayer, the next question is to consider its functional role in EC-ReSERS. The first step in nanogap regeneration is the stabilisation of the Au surface itself. Au-Cl adsorption is well documented to lift the native 22×√3 reconstruction of Au(111), a periodic pattern associated with high surface mobility and tensile stress.[71–73] Upon Cl adsorption, surface atoms undergo displacement that flattens the surface and constrains their mobility, producing a more energetically stable, unreconstructed state. Scanning tunnelling microscopy (STM) studies have shown that this structural transition is driven by adsorbate-induced Au displacement and formation of the Au-Cl adlayer[71–73]. By reducing adatom mobility, inter-facet diffusion, and surface relaxation, this process lowers the likelihood of sintering during oxide reduction. Thus, the presence of an Au-Cl adlayer stabilises the atomic lattice, providing the necessary structural precondition for nanogap stabilisation.

At the scale of the nanogap, the orientation of the Au-Cl surface dipole further contributes to stabilisation. The $Au^{\delta+}\rightarrow Cl^{\delta-}$ dipoles point outward from the opposing facets of the gap, producing a mutual dipole-dipole repulsion across the nanogap. This electrostatic effect helps prevent facet collapse at the moment of oxide reduction, when the gap would otherwise be vulnerable to sintering. In this way, Au-Cl acts as a transient electrostatic scaffold, maintaining the physical integrity of the nanogap until stabilising ligands can return.

Beyond its role as a transient electrostatic scaffold, the Au-Cl intermediate state also creates a favourable interfacial environment that facilitates the re-introduction of CB[5] as a steric stabiliser. During the Au-Cl state, the positive polarisation of the Au surface can attract the electron-rich carbonyl portals of CB[5], facilitating oriented rebinding. High-wavenumber SERS shows that on Au-Cl surfaces, C-H stretches between 2700-2850 cm^{-1} are consistently suppressed, while the asymmetric CH_2 stretch at 2950 cm^{-1} is retained or enhanced (**Supplementary Figure 39a**). This selective retention suggests an ordered binding mode, likely mediated by local field effects and interactions between the C-H groups and the Cl of the Au-Cl bond. Prior studies have shown that C-H--Cl-Au dipole-dipole interactions are sufficiently strong to influence supramolecular assembly[86–88], and this likely contributes to CB[5] retention in the nanogap. As CB[5] begins to re-enter at this stage, continued cathodic sweeping reduces the remaining Au-Cl species, facilitating full displacement of Cl^- and allowing CB[5] to dominate the nanogap interface. This ligand exchange completes the regeneration cycle, re-establishing the original CB[5]-stabilized architecture.

Failure at high buffer concentration. Although Au-Cl formation is a prerequisite for successful regeneration, its ability to facilitate CB[5] rebinding is suppressed at high buffer concentration. Under these conditions, the EDL becomes strongly compressed, leading to two key effects. First, the field associated with the Au-Cl dipole is screened and confined to the immediate surface region. Second, the nanogap becomes crowded with competing buffer anions, particularly phosphate. These effects disrupt the normally favourable interactions between Au-Cl and CB[5]. Excess anions can screen the partially positive Au atoms that would otherwise attract the electron-rich carbonyl portals of CB[5], while electrolyte cations and anions can shield dipole-dipole interactions between $Cl^{\delta-}$ and the C-H groups of CB[5]. As a result, the electrostatic guidance that normally promotes oriented CB[5] rebinding is weakened, preventing effective re-scaffolding.

Failure with Au-OH adlayers. A second mode of failure arises under alkaline conditions, where Au-Cl is absent and the surface is instead dominated by Au-OH. When +0.2 V is applied in 100 mM NaOH (containing 0.1 mM CB[5] and 0.5 mM Cl^-), electrochemical and spectroscopic signatures indicate the formation of a stable Au-OH

adlayer (**Supplementary Figure 31b**). Higher potentials were avoided as significant Au oxidation was observed beyond +0.2 V (**Supplementary Figure 38b**). Here, the Au-OH vibrational mode shifts from 375 to 400 cm^{-1} upon application of +0.2 V and remains present after OCP relaxation, which stabilizes at +0.1 V (starting from -0.1 V). These results indicate the formation of a surface-bound OH^- layer, but the nature of this interface is distinct from that of the Au-Cl adlayer.

Previous studies on the Au-OH surface layer report that the Au-OH bond is predominantly ionic in character. DFT analysis showed that OH^- interacts with Au through weak orbital overlap and strong Pauli repulsion, leading to minimal e^- sharing[89]. Thus, the electron density remains localized on OH^-, with little perturbation of the Au lattice. The result is a negatively charged interface with OH^- remaining strongly hydrated[67]. The result is a negatively charged, strongly hydrated interface that is disordered and lacks the ordered superstructure typical of Au-Cl. The disordered nature of the layer along with strong solvent screening therefore contributes directly to the weak reported surface dipole (0.02 D).[67] Moreover, the same disorder can contribute to the system's optical response. DF scattering measurements show a repeatable 20% decrease in scattering intensity upon Au-OH formation, with no consistent resonance shift. This contrasts with the modest damping and clear redshift observed for Au-Cl. The optical behaviour suggests that Au-OH forms a strongly lossy, disordered interface, where hydration, ionic character, and disordered structure lead to significant plasmon damping.

These interfacial properties directly impair CB[5] rebinding. Surface potential measurements of Au-OH also show that the surface is strongly negative (-110 mV), creating an environment that is electrostatically unfavourable for the electronegative portals of CB[5]. Formation of the Au-OH adlayer results in a suppression of all C-H modes, replaced by a single broad and lower-intensity peak (**Supplementary Figure 39c,d**). This indicates either loss of CB[5] from the hotspot or a disordered binding orientation. While CB[5] signals may transiently reappear following reduction, SERS intensities plateau at lower values and progressively degrade with repeated ReSERS cycles, indicating incomplete nanogap regeneration. This likely reflects partial CB[5] binding at nanogap edges rather than full re-entry into the confined gap centre, leaving key regions vulnerable to sintering.

These results demonstrate that the Au-Cl adlayer forms an ideal interface that actively supports CB[5] rebinding and nanogap stabilization. Its outward-oriented dipole and ability to engage both C-H and carbonyl interactions make it uniquely suited as an intermediate during re-scaffolding. Failure arises when the role of Au-Cl is compromised. At high buffer concentration, the Au-Cl adlayer still forms, but its fields are screened and crowded by excess ions, preventing CB[5] from effectively rebinding to the nanogap. Under alkaline conditions, Au-Cl is absent and replaced by a disordered, ionic Au-OH adlayer, which produces a strongly negative interface that is electrostatically incompatible with CB[5] rebinding. In both cases, re-scaffolding fails, leaving nanogaps vulnerable to sintering. These findings establish Au-Cl not merely as a correlated surface species but as the functional intermediate that enables reproducible nanogap regeneration.

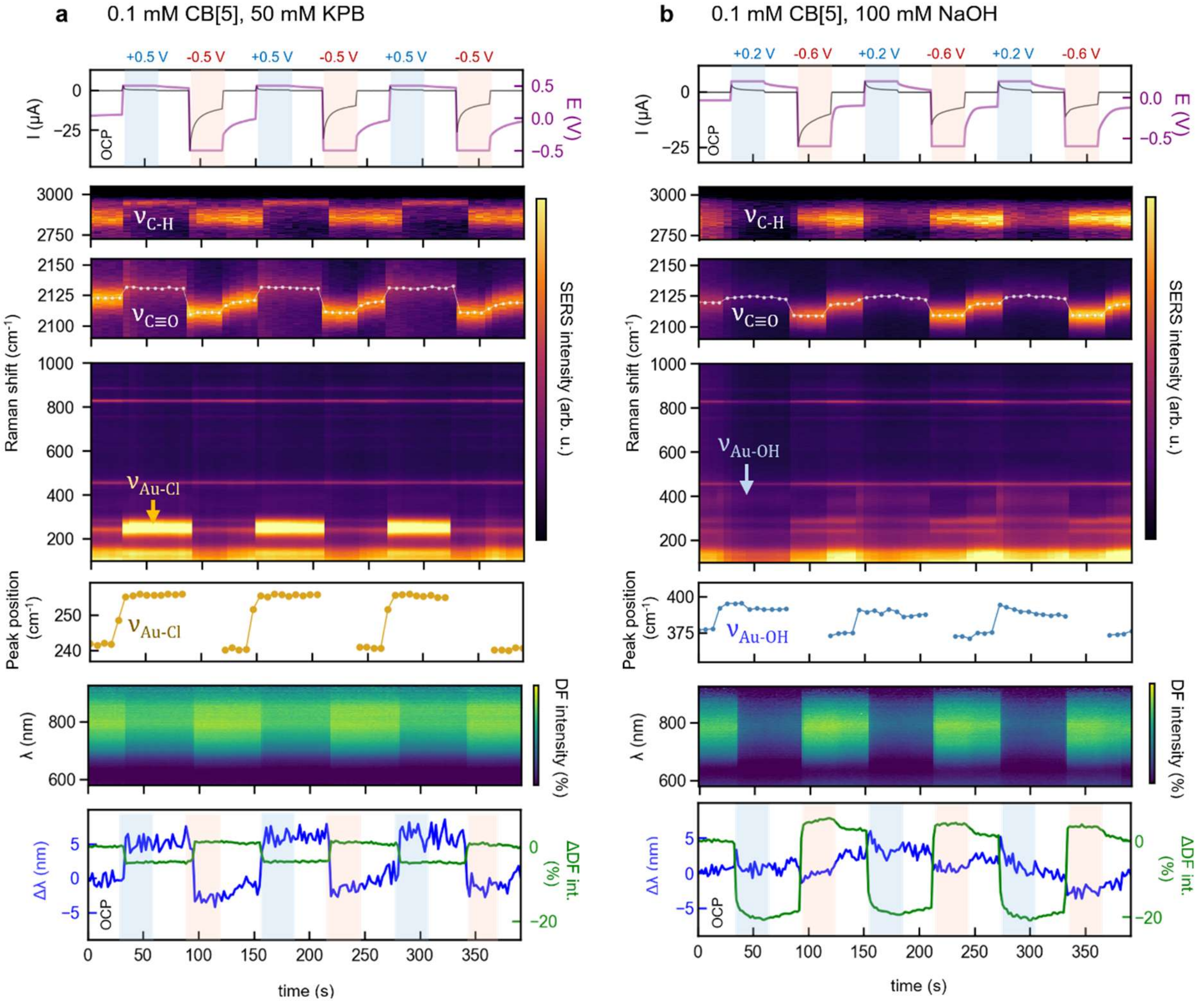

Supplementary Figure 31 | Electrochemical control of Au-Cl and Au-OH- adlayer formation. (**a-b**) Time-series current, potential, and SERS spectra of MLagg-CB[5] undergoing potential switching between anodic and reducing potentials in 0.1 mM CB[5] and (**a**) 50 mM potassium phosphate buffer (pH 7.0) or (**b**) 100 mM NaOH (pH 13). Between each applied potential, the system is allowed to relax to OCP. Wavenumber ranges of interest are highlighted in the time-series SERS spectra: the high wavenumber CB[5] C-H stretching modes, the carbon monoxide (C≡O) stretching mode with the peak position tracked (white line), and the lower wavenumber range showing CB[5] signature vibrations and the Au-Cl or Au-OH stretching modes. Au-Cl or Au-OH peak positions are tracked per time series SERS spectrum. The bottom panel shows the corresponding dark field scattering spectra as the MLagg-CB[5] undergoes the same potential switching. The changes in chain mode peak amplitude and position are plotted relative to the initial amplitude and position.

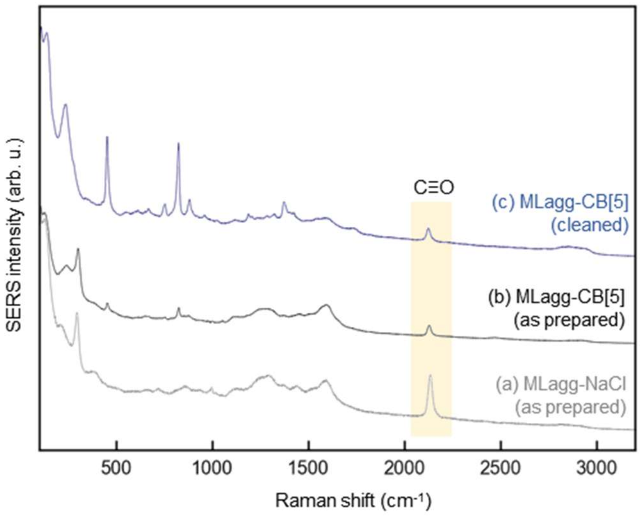

Supplementary Figure 32 | CO adsorbed on MLagg. Extended SERS spectra of (**a**) citrate-capped AuNPs aggregated with NaCl (MLagg-NaCl), (**b**) citrate-capped AuNPs aggregated with CB[5] (as prepared, prior to electrochemical cleaning), and (**c**) MLagg-CB[5] after electrochemical cycling with CB[5]. The peak at 2120 cm^{-1} is assigned to linearly adsorbed CO on Au.

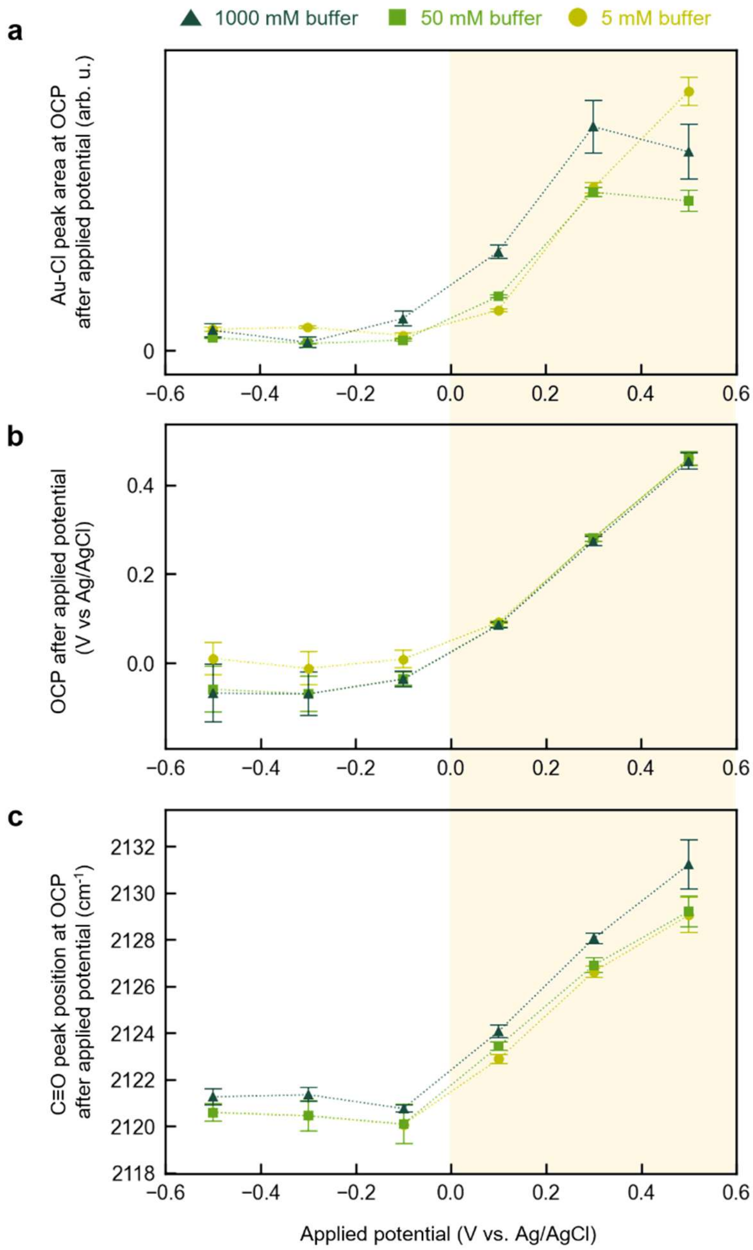

Supplementary Figure 33 | CO Stark shifts with Au-Cl adlayer stabilisation at OCP. SERS and OCP measurements taken after stepping the MLagg-CB[5] substrate to various applied potentials (x-axis) followed by relaxation to OCP, in 0.1 mM CB[5], 0.5 mM Cl^-, and 5 mM, 50 mM, or 1000 mM potassium phosphate buffer (pH 7.0). (**a**) Peak area of Au-Cl SERS peak measured after OCP relaxation, showing potential-dependent formation and stabilisation. (**b**) Measured OCP after the applied potential step. (**c**) Position of the CO vibrational mode measured at OCP after the applied potential step. Points are plotted as mean±1s.d. from measurements on *n*=3 different MLagg-CB[5] substrates.

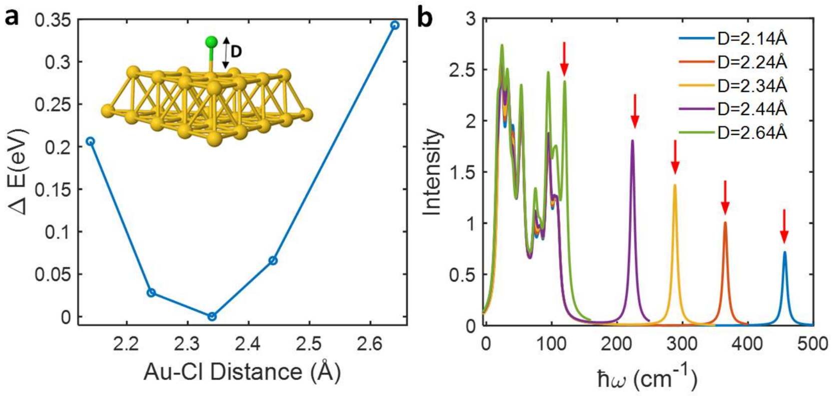

Supplementary Figure 34 | Calculated Raman scattering spectrum of Au-Cl. (**a**) Total energy differences for different Cl-Au distances, indicating the stability of each configuration. (**b**) Raman scattering spectra for different Cl-Au distances, showing changes in the Au-Cl peak position (indicated by red arrows).

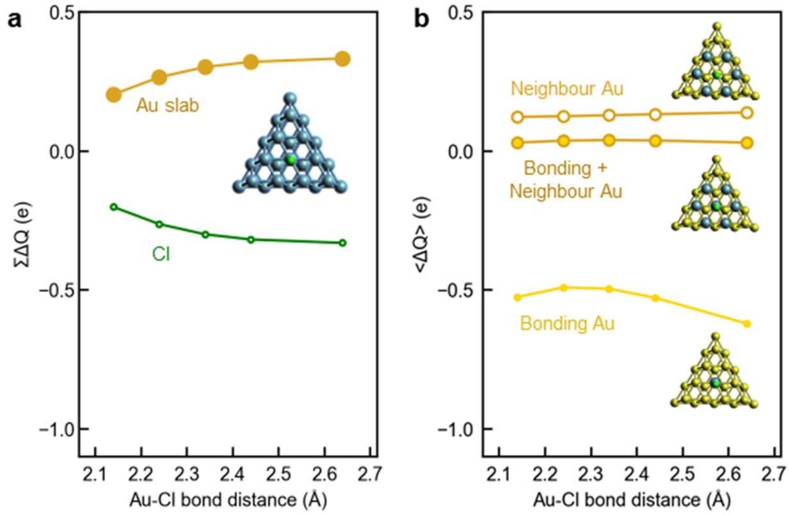

Supplementary Figure 35 | Charge transfer on Cl and selected Au atoms. (**a**) Mulliken charge (ΔQ) on Cl (green atom) in Au-Cl bond and sum of charges on entire Au slab (Au atoms highlighted in blue). (**b**) Average charge per Au atom in a selected cluster of Au atoms highlighted in blue: (*bottom*) the Au atom in the Au-Cl bond, (*top*) six neighbouring Au atoms surrounding the bonding Au, and (*mid*) all seven Au atoms (bonding and neighbouring).

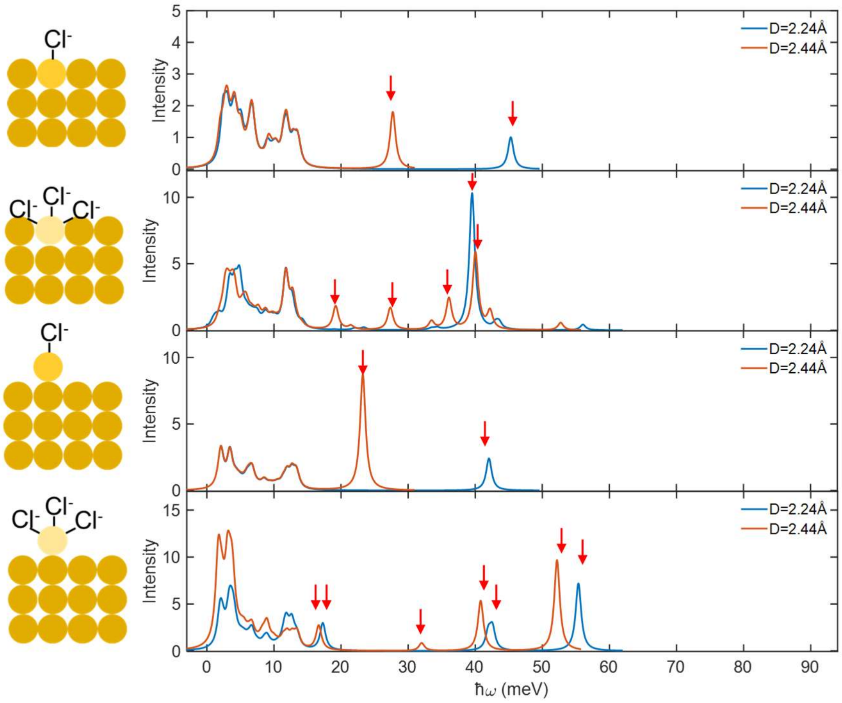

Supplementary Figure 36 | Calculated Raman scattering spectrum of Au-Cl configurations. Raman scattering spectra for different configurations of Cl on Au. The ground state Cl-Au distance is 2.24 Å, while the 2.44 Å distance corresponds to a configuration that is 0.003 eV higher in energy than the ground state. The baseline Cl-Au distance for all configurations is D = 2.24 Å. When labelled as D = 2.44 Å, it indicates that only one Cl-Au bond distance is increased to 2.44 Å, while others remain at the optimum distance. The next figure displays an example of vibrational modes associated with chlorine, highlighted by red arrows.

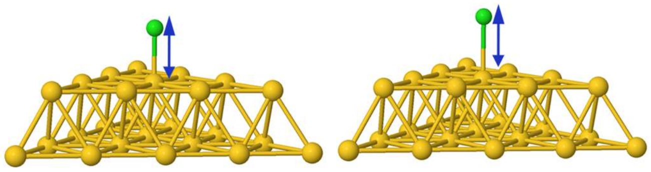

Supplementary Figure 37 | Vibrational modes of Au-Cl with D = 2.24 Å (left) and D = 2.44 Å (right).

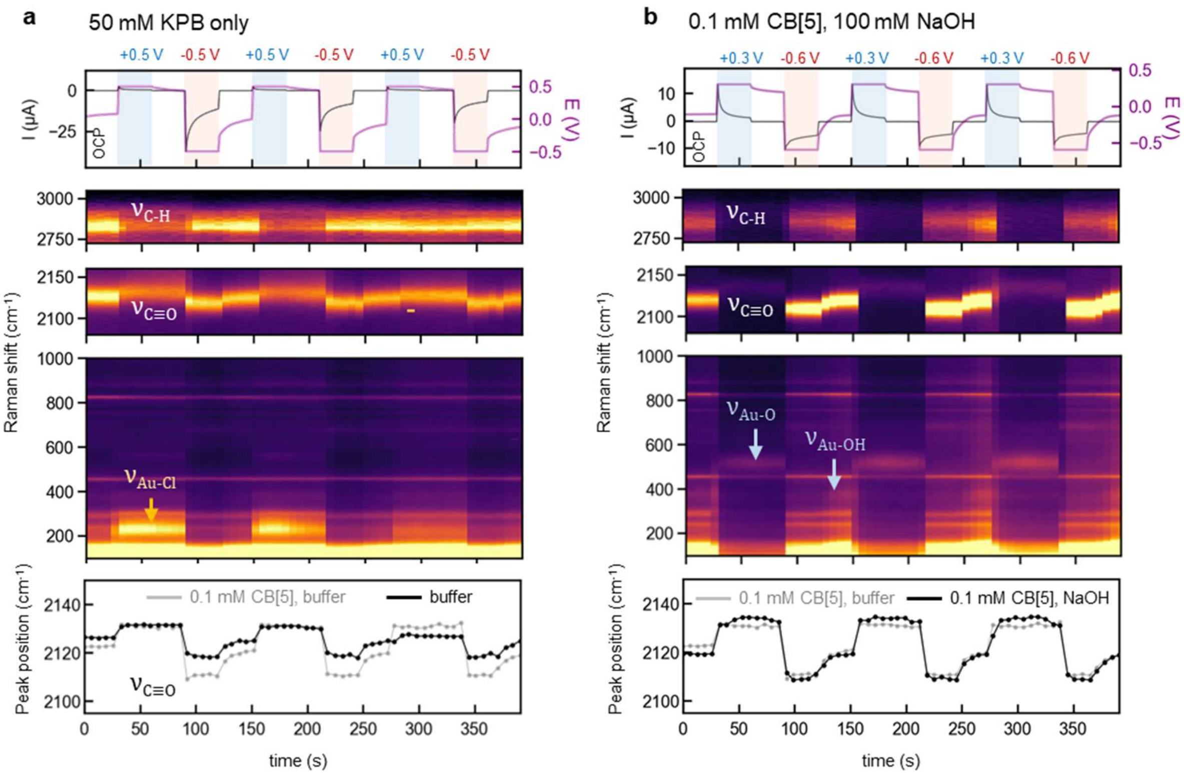

Supplementary Figure 38 | Formation of adlayers in different electrolytes. (**a-b**) Time-series current, potential, and SERS spectra of MLagg-CB[5] undergoing potential switching between anodic and reducing potentials in (**a**) 50 mM potassium phosphate buffer (pH 7.0) only or (**b**) 0.1 mM CB[5] and 100 mM NaOH (pH 13). In (b) the MLagg-CB[5] is polarized to a more positive anodic potential, resulting in the onset of Au oxide formation. Between each applied potential, the system is allowed to relax to OCP. Wavenumber ranges of interest are highlighted in the time-series SERS spectra: the high wavenumber CB[5] C-H stretching modes, the carbon monoxide (C≡O) stretching mode, and the lower wavenumber range showing CB[5] signature vibrations and the Au-Cl or Au-OH stretching modes. The peak position of the C≡O stretching mode is tracked in the bottom panel with the peak positions in 0.1 mM CB[5], buffer plotted for comparison.

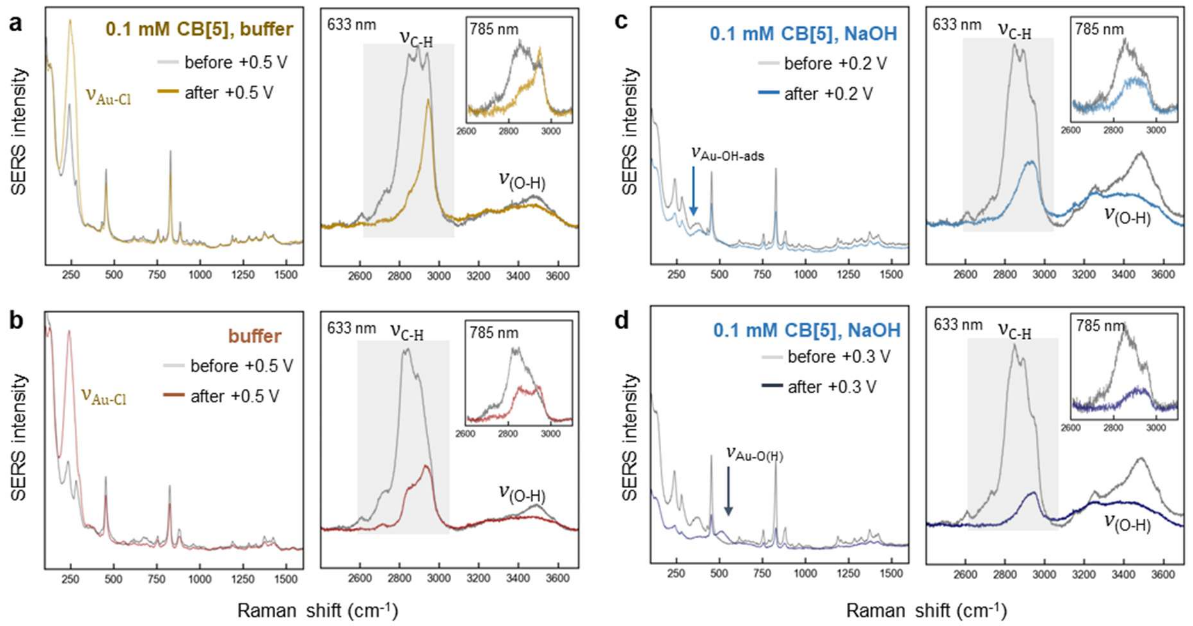

Supplementary Figure 39 | SERS spectra before and after adlayer formation. (a-d) SERS spectra of MLagg-CB[5] before and after the electrochemical formation of an adlayer (Au-Cl or Au-OH) in (a) 0.1 mM CB[5], 50 mM potassium phosphate buffer (pH 7.0), (**b**) 50 mM potassium phosphate buffer (pH 7.0), and (**c-d**) 0.1 mM CB[5] and 100 mM NaOH (pH 13.0). Fingerprint SERS spectra region was measured with 2 mW 785 nm laser excitation at 10 s integration time. High wavenumber SERS was measured with both 633 nm (10 s integration) and 785 nm (inset) laser excitation. Some variation in relative SERS intensity before and after adlayer formation was noted when the spectra were measured in 633 nm and 785 nm laser excitations. This can be due to the expected red shift (away from laser line) in the plasmon resonance of the MLagg-CB[5] after adlayer formation.

Supplementary Note 11. XPS and SERS characterisation of Au-Cl

To investigate the chemical nature of the Au-Cl species formed at the AuNP surface during ReSERS, we performed detailed surface analysis using X-ray photoelectron spectroscopy (XPS) and SERS on MLagg-CB[5] samples that exhibit strong Au-Cl vibrational signatures. Au-Cl adlayers are known to form electrochemically and from the gas phase[4,71–73], and the Au-Cl bond has been widely described as covalent in nature[4,70]. However, characterisation of the electronic structure of the Au-Cl adlayer has remained limited due to instrumental resolution limits in lab-based XPS[90,91]. To resolve the nature of this highly surface-localised Au-Cl adlayer, we combine low-energy, surface-sensitive XPS with SERS, enabling correlation between electronic structure and vibrational signatures.

<u>XPS and SERS of MLagg Au-Cl adlayer</u>

We present XPS analysis alongside SERS measurements to confirm the presence of a covalent-like Au-Cl layer on the MLagg surface (**Extended Data Figure 7**). DFT analysis suggests that formation of a Au-Cl adlayer induces significant charge transfer between the bonding Au and Cl and even induces charge redistribution from surrounding non-bonding Au atoms (**Supplementary Figure 35**). As a result of Au-Cl bonding, the bonding and nearby Au atoms exhibit a cumulative positive Mulliken charge (+0.2-0.3), balancing the partial negative charge on Cl (-0.3). To potentially detect this shift in Au charge distribution, we analyse an MLagg sample prepared from Ch-ReSERS, which exhibits a SERS Au-Cl vibrational mode at 260 cm^{-1}. XPS analysis at low photon energy (700 eV) reveals Au 4f peaks at: 84.0 eV/87.7 eV corresponding to Au(0), surface Au(s), and reconstruction Au(r) peaks at slightly lower binding energies, and a small feature above the Au(0) peaks at 84.8 eV/88.4 eV. The 84.8 eV/88.4 eV feature appears below the Au oxide peaks at 85.4 eV/89.1 eV, suggesting that the peaks can be attributed to Au with a partial positive charge, consistent with a covalent Au-Cl interaction. However, we note that the 84.8 eV peak is not unique to Au-Cl, as it is also present in the as-prepared MLagg sample, which lacks an Au-Cl SERS peak, and even in a planar Au reference sample. Thus, the 84.8 eV peak alone is not a definitive marker for Au-Cl and may arise from other Au-ligand interactions or even adventitious contamination. In addition, our DFT suggests that different Au atoms can exhibit different charges depending on whether they are the bonding or neighbouring Au atoms. Thus, as surface Au atoms in sub-monolayer Au-Cl can adopt multiple charged states, it can be challenging to assign a single Au 4f peak to Au-Cl.

To more reliably identify the Au-Cl surface species, we turn to the Cl 2p XPS. Across all MLagg samples exhibiting SERS Au-Cl peaks, Cl 2p XPS consistently shows two distinct pairs of peaks: one at 197.6 eV/199.2 eV, corresponding to weakly bound chloride ions (Cl^-), and another at 200.2 eV/201.8 eV, which is typically attributed to $Cl^{\delta-}$ such as that in a covalent bond[92]. Since no new Cl-based organic bonds are expected, the higher-BE Cl peak most likely represents charge transfer between Au and Cl, resulting in Cl bearing a partial negative charge. Notably, in the HCl-treated MLagg sample (without CB[5]), the high-BE Cl peaks appear at a slightly lower binding energy (199.6 eV and 201.2 eV), suggesting CB[5] interactions slightly shift the Au-Cl electronic environment via dipole-dipole interactions. Alternatively, this can also reflect differences in adlayer density, as this can similarly result in different degrees of charge transfer between Au and Cl.[73,91]

XPS evidence of the charge redistribution in Au-Cl adlayers has been previously difficult to measure due to the limited resolution available in lab-based XPS[90,91]. Previous measurements of Au-Cl adlayers primarily measured only Cl^- signals (197.6 eV/199.2 eV) in the Cl 2p spectra and were only able to detect minor shifts in this peak upon higher-density Au-Cl adlayer formation[91]. The absence of strong shifts in the Cl 2p spectra of these previous measurements can be attributed to limited spectral resolution or x-ray induced photoreduction of the Au-Cl adlayer, which can result in the dominance of Cl^- signals. Energy-tuned measurements of the MLagg-CB[5] support this interpretation: when the Cl 2p spectrum was recorded at different photon energies (450, 700, and 1100 eV, **Supplementary Figure 40**), the higher-BE peak at 200.2/201.8 eV became progressively less prominent with increasing energy. This trend reflects the reduced surface sensitivity at higher excitation energies, where the deeper probe volume diminishes the relative contribution of the true surface Au-Cl adlayer and enhances signals from weakly bound or photoreduced Cl^-. By contrast, low-energy measurements (450-700 eV) consistently revealed a distinct higher-BE Cl peak that can be assigned to $Cl^{\delta-}$, confirming the covalent nature of the Au-Cl adlayer.

XPS and Raman of $AuCl_{(s)}$ and $AuCl_{3(s)}$

As a reference, we compare the Au-Cl adlayer with bulk $AuCl_{(s)}$. In $AuCl_{(s)}$, Cl atoms are configured in a linear Cl-Au-Cl-Au molecular chain arrangement[93], where each Cl is coordinated to 1-2 Au atoms. These two types of electronic environments are reflected in two sets of high Cl 2p binding energies (199.7 eV/201.3 eV and 200.7 eV/202.3 eV). These peaks are higher than Cl^- (197.6 eV/199.2 eV), indeed confirming that the Au-Cl bonds in $AuCl_{(s)}$ exhibit delocalization of electron density and metal-ligand orbital overlap. The two sets of Au 4f peaks in $AuCl_{(s)}$ (85.2 eV/88.8 eV and 85.8 eV/89.5 eV) are also at higher BE than surface Au-Cl (84.8 eV/89.1 eV), consistent with stronger electron withdrawal from Au due to dual Cl coordination in the bulk lattice. Furthermore, the Raman spectrum of $AuCl_{(s)}$ exhibits a strong Au-Cl vibrational mode at 290 cm^{-1}, whereas the Au-Cl adlayer in the MLagg shows a mode at 260 cm^{-1} indicating that bulk Au-Cl bonds are stronger and more delocalized than those at the surface. This is consistent with previously calculated force constants for Au-Cl bonds in a surface Au-Cl adlayer (1.25-1.6 x 10^5 dyn cm^{-1}) versus the bulk $AuCl_{(s)}$ (1.75 x 10^5 dyn cm^{-1})[10,74]. These comparisons confirm that surface-bound Au-Cl is distinct from bulk $AuCl_{(s)}$, where Cl is more polarized from bonding with dual-coordinated Au atoms.

To further clarify the electronic and chemical distinction between surface-bound Au-Cl species and higher-valent gold chlorides, we also examined the XPS and Raman spectra of solid $AuCl_{3(s)}$, which exists as a planar dimeric molecule, Au_2Cl_6, with each Au(III) atom coordinated to three Cl ligands, including two bridging chlorides[6]. XPS analysis of $AuCl_{3(s)}$ reveals characteristic high binding energy Au 4f peaks at 86.0 eV and 89.7 eV, consistent with a higher valent Au(III) state. The corresponding Cl 2p peaks at 200.7 eV and 202.3 eV reflect strong Au-Cl charge transfer and are higher than those of free chloride, consistent with covalent-like bonding between Cl and Au. This shift supports the view that even in Au(III) complexes, the Au-Cl interaction retains substantial covalent character, consistent with previous calculations that show the terminal Cl and bridging Cl bear charges of -0.22 and -0.33 respectively[91]. Notably, X-ray exposure during XPS also induces the gradual emergence of lower binding energy Au 4f features (~84.0 and 84.8 eV), likely due to partial photoreduction of $AuCl_{3(s)}$ to Au(0) and intermediate Au-Cl species.

Raman spectroscopy of $AuCl_{3(s)}$ reveals intense bands at 322 and 345 cm^{-1}, corresponding to symmetric and asymmetric Au-Cl stretching modes within the Au_2Cl_6 dimer, alongside a bending mode near 178 cm^{-1}. The higher-frequency stretching modes reflect stronger bonding, as expected for Au(III)-Cl interactions, which involve higher bond order and stronger metal-halide bonding than those of lower-valent Au species.

Summary

XPS and SERS support the existence of a surface Au-Cl layer, where the Au-Cl bond exhibits covalent character. The Au carries a partial positive charge, leading to a small shift in Au 4f above Au(0), while Cl 2p reinforces the distinction between covalent Au-Cl and weakly bound Cl^-. CB[5] further modifies the Au-Cl environment, shifting its binding energy slightly higher. These findings confirm that Au-Cl bonding in MLagg is distinct from the Au-Cl bonds formed in $AuCl_{(s)}$ and $AuCl_{3(s)}$, and instead exists as a distinct surface-stabilized covalent interaction with surface Au.

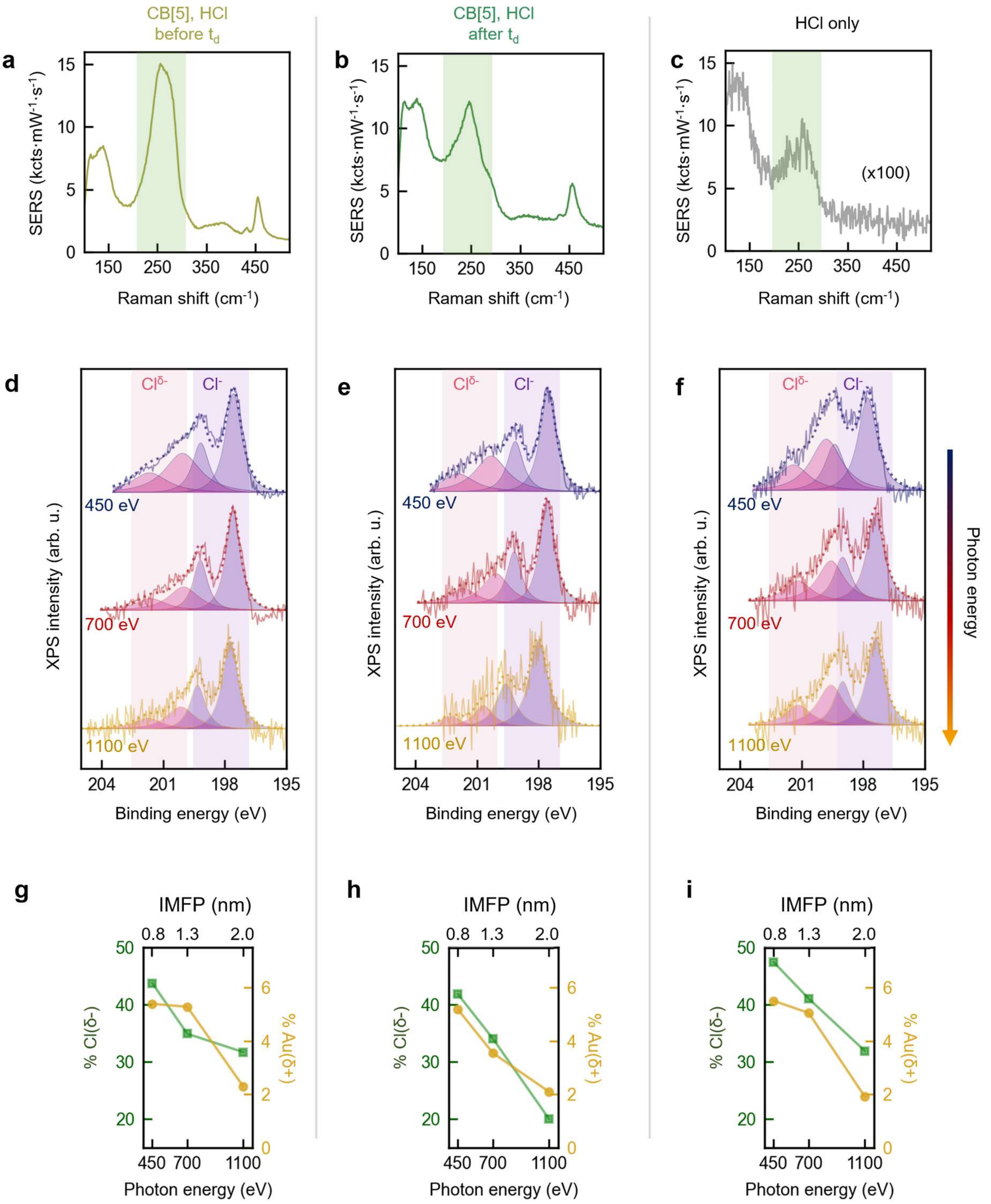

Supplementary Figure 40 | XPS depth profile of MLagg-CB[5] samples with Au-Cl. (**a-c**) SERS spectra of MLagg-CB[5] samples after various treatments, (**d-e**) Cl 2p XPS spectra and peak fits measured at various photon energies (450, 700, and 1100 eV). (**g-i**) Ratios in % of Cl(δ-) 2p and Au(δ+) 4f as a function of photon energy or approximate inelastic mean free path (IMFP).

Supplementary Note 12. Au-Cl formation and reduction in EC-ReSERS

Understanding the redox processes underlying Au-Cl adlayer formation is critical to elucidating the mechanistic basis of electrochemical regeneration in EC-ReSERS. Here, we combine CV with time-resolved SERS to investigate the evolution of the Au-Cl vibrational signature (240-270 cm^{-1}) and its correspondence with oxidation and reduction currents. Our first objective is to assign specific redox currents to the formation and reduction of the Au-Cl adlayer and correlate these processes with shifts in the Au-Cl SERS band. Our second objective is to evaluate the extent of Au-Cl formation relative to Au oxide formation. By comparing the integrated charge associated with Au-Cl formation/reduction to that of Au oxide, we assess whether the Au-Cl coverage approaches monolayer levels or remains sub-monolayer. This, in turn, allows us to explore whether greater Au-Cl coverage improves the subsequent re-binding of CB[5] molecules and thereby promotes more effective nanogap re-scaffolding.

<u>*Au-Cl Redox Processes*</u>

To deconvolute the redox currents specifically associated with Au-Cl adlayer formation and reduction, we performed CV and time-resolved SERS within a limited potential window (-0.80 V to +0.5 V) that avoids Au oxide formation. MLagg-CB[5] substrates were cycled in 0.1 mM CB[5] (with associated ~0.5 mM Cl^-) and 50 mM potassium phosphate buffer (pH 7.0) and compared with the full EC-ReSERS cycling range to highlight the isolated contribution of Au-Cl transformations (**Supplementary Figure 41a**). A control CV of the bare FTO electrode was also recorded, which confirms that the cathodic current peak at -0.6 V is due to oxygen reduction reaction (ORR) background and not AuNP processes.

During the anodic sweep to +0.5 V, the MLagg-CB[5] exhibits two well-defined anodic peaks at +0.15 V and +0.36 V, absent in the FTO control (**Supplementary Figure 41a**). These are assigned to distinct stages in Au-Cl adlayer formation, supported by corresponding changes in the Au-Cl SERS peak (**Supplementary Figure 41b**). As the potential is swept anodically, the Au-Cl peak at ~240 cm^{-1} undergoes an increase in intensity and progressive peak shift: first to ~250 cm^{-1} at 0.15 V, then further to ~270 cm^{-1} at 0.36 V. This sequence suggests an increase in Au-Cl coverage and structural ordering, consistent with a phase-like transition from a sparse adlayer to a more dense, ordered adlayer[75], as previously established from second-harmonic generation (SGH) measurements during the formation of Au-Cl adlayers[73]. Previous electrochemical[72,73] and DFT analysis[75] of Au-Cl adlayers on Au also showed that the magnitude of the Au-Cl surface dipole decreases with increasing coverage due to inter-adsorbate repulsion. To compensate for this repulsion, the Au-Cl adlayer has been shown to exhibit electro-compressibility, where increased packing density induces greater partial charge transfer from Cl to Au.[73,91] This can be associated with changes in the Au-Cl surface structure, where Au atoms can reconstruct and incorporate into the surface layer[86,90,94]. Our DFT data suggests that less negative charges on Cl from greater covalent bonding character with Au are associated with shorter Au-Cl bond distances, which in turn result in a shift in the Au-Cl peak position to higher frequencies. Thus, the observed SERS evolution now provides spectroscopic evidence of electro-compressibility, where increased adlayer coverage compresses the Au-Cl bond and enhances its covalent character as the potential becomes more positive.

During the cathodic sweep, two corresponding reduction peaks are observed at +0.30 V and -0.20 V, which align with downward shifts in the Au-Cl peak from 270 cm^{-1}→250 cm^{-1} and then from 250 cm^{-1}→240 cm^{-1}, respectively. These reversals are attributed to partial reduction of Au-Cl, consistent with a progressive loss of adlayer density and order as the surface returns toward a less-coordinated or fully reduced Au(0) state.

To probe how Cl^- concentration influences adlayer formation dynamics, additional experiments were conducted by incrementally increasing Cl^- concentration with additions of 1 mM and 10 mM KCl. Under higher [Cl^-], the Au-Cl peak more readily increases in intensity and shifts toward ~270 cm^{-1}. This indicates that elevated Cl^- concentrations accelerate the formation and ordering of the Au-Cl adlayer, likely by increasing surface site saturation and facilitating denser adsorbate packing.

These results reveal that Au-Cl formation proceeds via at least two redox-accessible stages, each associated with distinct Au-Cl vibrational shifts and current features. The potential-limited cycling approach enables a clean assignment of these redox events, independent of Au oxide formation, and highlights the influence of Cl^- availability on adlayer formation.

Au-Cl during EC-ReSERS

To investigate Au-Cl during EC-ReSERS cycling, we analysed the evolution of Au-Cl-associated redox currents and SERS signals across a range of scan rates. Unlike the potential-limited regime, EC-ReSERS involves extended anodic potentials that induce oxidation to Au-Cl, Au(III)-Cl, and Au oxide. On the reverse sweep, the reduction pathway proceeds first through oxide and Au(III)-Cl reduction, before Au-Cl reforms and subsequently reduces. The cathodic sequence implies a different environment for Au-Cl formation and reduction during the re-scaffolding phase, potentially influencing its function in enabling CB[5] rebinding.

In the anodic sweep, the two distinct Au-Cl oxidation peaks at +0.15 V and +0.36 V are consistently observed and attributed to progressive formation of the Au-Cl adlayer (**Supplementary Figure 42a**). However, the integrated oxidation charge associated with Au-Cl (Q^{ox}_{Au-C}) is modest at only 4-8% of the charge associated with the formation of Au oxide (Q^{ox}_{AuOx}), far below the 50% expected for complete monolayer coverage (**Supplementary Figure 42b**). This suggests that only a fraction of the surface undergoes oxidation to form an Au-Cl adlayer before further oxidation occurs. The limited formation of Au-Cl is primarily due to constraints on Cl^- adsorption, as pre-adsorbed CB[5] molecules restrict the number of available surface sites for Cl^- binding, which can be further constrained by inter-adsorbate repulsion. Even at different scan rates, the maximum Au-Cl SERS intensity (normalized to the initial CB[5] intensity) remains relatively constant, suggesting saturation of binding sites (**Supplementary Figure 42b**). SERS measurements also confirm that CB[5] intensity remains relatively stable as Au-Cl forms (**Extended Data Figure 5**), and only decreases with higher applied potentials, where Au-Cl intensity decreases and other anions such as hydroxide and phosphate start to bind. As a result, Au-Cl formation is limited to less than a monolayer before further oxidation occurs.

Further oxidation of Au-Cl to Au(III)-Cl and Au oxide is also strongly scan rate dependent. The $Q^{ox}_{Au-Cl}/Q^{ox}_{AuOx}$ ratio decreases for lower scan rates, from 8% at 500 $mV{\cdot}s^{-1}$, to only 4% at 5 $mV{\cdot}s^{-1}$. This trend can be explained by the increase in the Au oxide charge (Q^{ox}_{AuOx}) at slower scan rates, which allows oxidation to progress more fully, facilitating the conversion of Au-Cl into Au(III). Indeed, time-resolved SERS shows that oxidation at slow scan results in a sequential transformation of Au-Cl to Au(III)-Cl/Au oxide, and finally to predominantly Au oxide (**h**). In contrast, at fast scan rates, further oxidation is kinetically constrained, preventing complete conversion of Au-Cl to Au(III)-Cl or Au oxide (**j**).

In the cathodic sweep, the opposite trend is observed, with $Q^{red}_{Au-Cl}/Q^{red}_{AuOx}$ ratio decreasing as scan rate increases (**Supplementary Figure 42d,** 14% at 5 $mV{\cdot}s^{-1}$ *vs* 2% at 500 $mV{\cdot}s^{-1}$). This suggests that Cl^- diffusion controls Au-Cl recovery, as Cl^- must reach the surface before a Au-Cl adlayer can re-form after Au(III)-Cl and Au oxide reduction. At slow scan rates, there is more time for Cl^- to rebind, leading to higher Au-Cl reduction charge and stronger Au-Cl SERS intensity. In contrast, at fast scan rates, Cl^- diffusion is limited, reducing Au-Cl formation and resulting in a weaker reduction signal.

However, care must be taken in interpreting the $Q^{red}_{Au-Cl}/Q^{red}_{AuOx}$ ratio. The broad reduction peak near +0.4 V, traditionally associated with Au oxide reduction, likely includes overlapping contributions from Au(III)-Cl → Au-Cl and even partial Au-Cl reduction (**Supplementary Figure 41a**). Thus, the charge assigned to “Au oxide” encompasses multiple redox events. In contrast, the discrete -0.20 V peak captures only the later-stage, clearly resolved Au-Cl reduction. Therefore, the calculated $Q^{red}_{Au-Cl}/Q^{red}_{AuOx}$ ratio underestimates the true proportion of Au-Cl involved in the redox process. While this limits the quantitative precision of current-based comparisons, the observed scan rate trends and corresponding SERS signals still provide insight into the kinetics of Au-Cl regeneration and its role in substrate recovery.

This variation in Au-Cl formation during re-scaffolding has functional consequences. Because CB[5] is fully displaced during the oxidation sweep, its re-binding depends critically on the interfacial environment created during reduction. At slow scan rates, the extended diffusion time allows Cl- to rebind and stabilize the surface as Au-Cl, creating a neutral, covalent-like adlayer that facilitates CB[5] re-entry. At the same time, the extended diffusion time also enables effective Au oxide reduction while the Au-Cl adlayer is formed, therefore ensuring the surface is predominantly characterised by the Au-Cl adlayer. Notably, even at sub-monolayer Au-Cl coverage, the Au-Cl surface dipole can already have a significant impact on interfacial properties. SERS data confirm a correlation between Au-Cl peak intensity during reduction and CB[5] recovery, with both signals reaching higher values at lower scan rates (**Supplementary Figure 42d**). Indeed, during slow scan rates, CB[5] more effectively rebinds in the nanogap during re-scaffolding, reaching an intensity that is greater than its initial intensity (**Supplementary Figure 42d,** normalized CB[5] signal > 1). In contrast, at fast scan rates, Cl^- diffusion is limited, resulting in weaker Au-Cl formation and reduced CB[5] rebinding.

These results support a direction-dependent formation of Au-Cl in EC-ReSERS. During oxidation, Au-Cl formation is limited by competition with CB[5], and its transient presence may serve as a precursor to further oxidation rather than a stabilizing layer. However, during reduction, Au-Cl acts as a critical intermediate that enables CB[5] re-scaffolding, particularly under kinetically favourable conditions where Cl^- can reach the surface in sufficient time and concentration. The correlation between Au-Cl and CB[5] intensities highlights the functional importance of this adlayer in governing nanogap regeneration and SERS signal reproducibility.

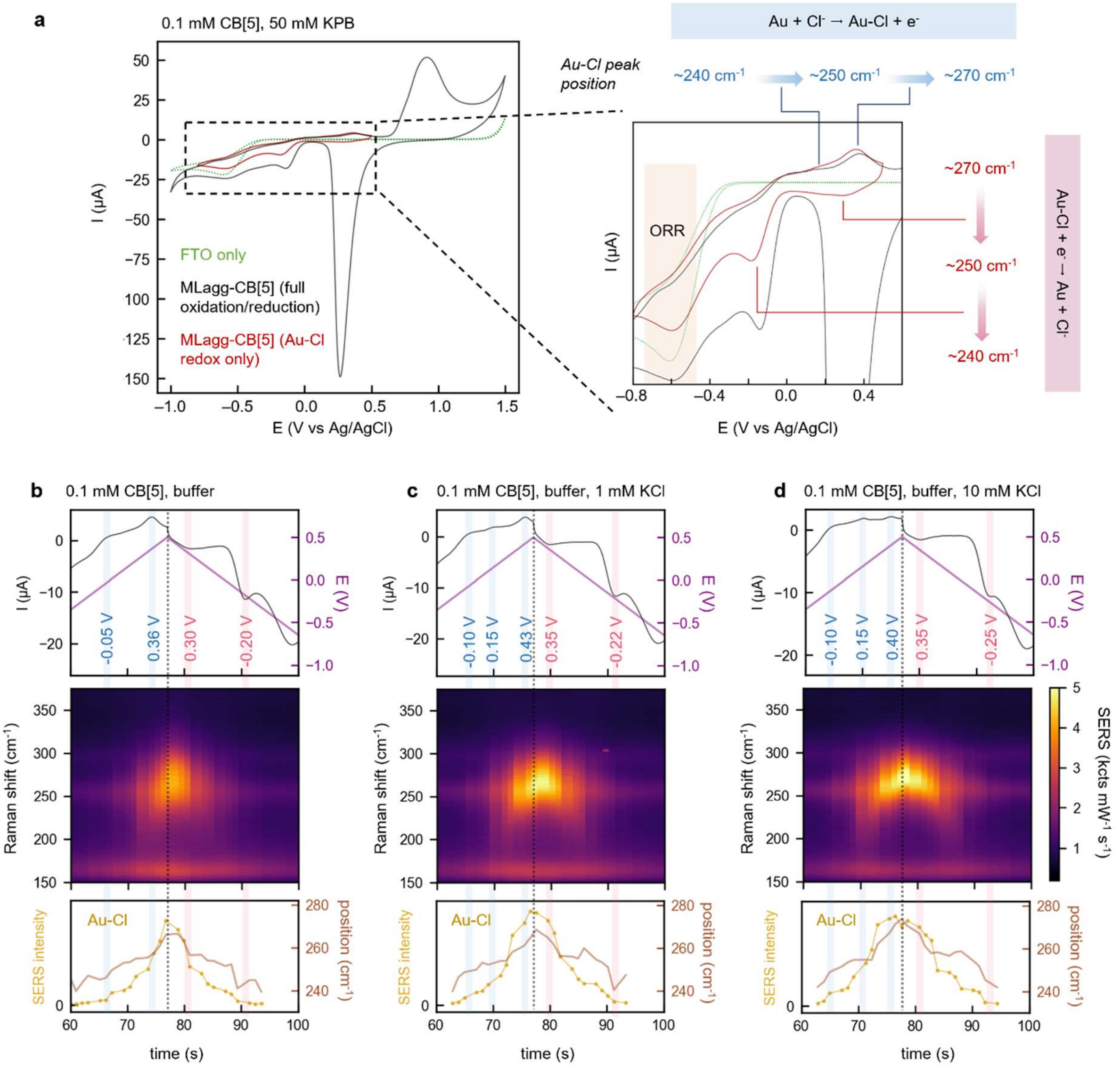

Supplementary Figure 41 | Formation and reduction of Au-Cl adlayer. (**a**) Cyclic voltammograms (CV) of MLagg-CB[5] and bare FTO in 0.1 mM CB[5], 50 mM potassium phosphate buffer (pH 7.0), with scan rate of 50 mV s^{-1}. Zoom-in panel highlights region where Au-Cl formation and reduction occur, with corresponding SERS Au-Cl peak position shifts annotated per oxidation and reduction peak. (**b-d**) Applied potential vs Ag/AgCl (purple solid line) and corresponding current response (black solid line) from MLagg-CB[5] cycled between +05 V and -0.8 V in (**a**) 0.1 mM CB[5], 50 mM potassium phosphate buffer, (**c**) with additional 1 mM KCl, or (**d**) with additional 10 mM KCl at 50 mV s^{-1}. Time-series SERS spectra during the potential scans is shown, along with the peak area (yellow line) and peak position (brown) of Au-Cl. Blue and pink lines highlight oxidative and reductive current peaks associated with Au-Cl.

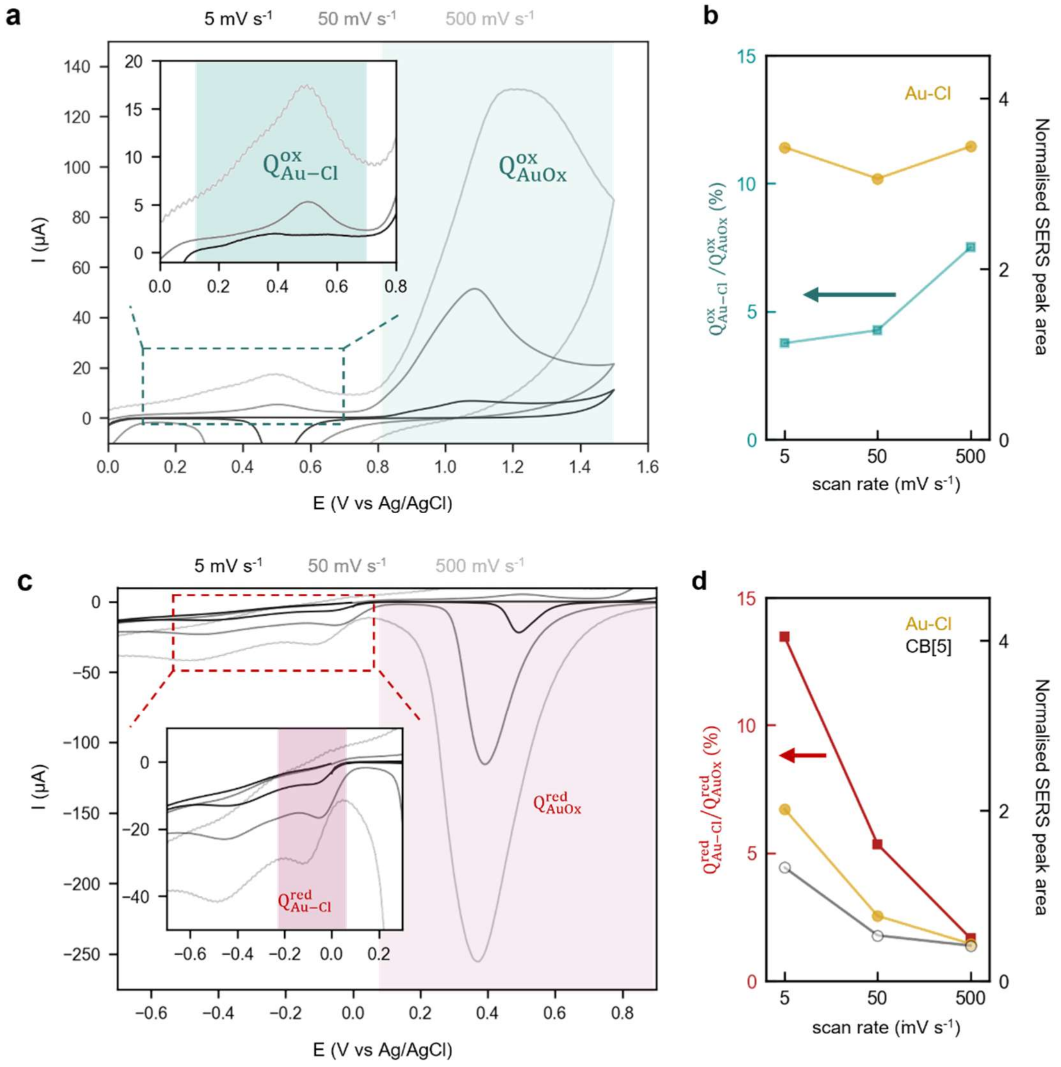

Supplementary Figure 42 | Formation and reduction of Au-Cl adlayer during EC-ReSERS at varying scan rates. Zoomed-in region of the cyclic voltammogram of MLagg-CB[5] in 0.1 mM CB[5], 50 mM potassium phosphate buffer pH 7.0 highlighting the (**a**) anodic current peaks corresponding to Au-Cl formation and (**c**) cathodic peak corresponding to Au-Cl reduction at varying scan rates (v=5, 50, 100 mV·s^{-1}). The integrated charge ratio (%) of (**b**) Au-Cl : Au oxide formation ($Q^{ox}_{Au-Cl}/Q^{ox}_{AuOx}$) or (**d**) reduction ($Q^{red}_{Au-Cl}/Q^{red}_{AuOx}$) are plotted with the corresponding normalized Au-Cl SERS intensity *vs* log v. SERS peak area normalized with respect to initial CB[5] peak area. In (**d**) the normalized SERS peak area of the rebinding CB[5] peak is also plotted.

Supplementary Note 13. Au-Cl adlayer destabilisation with OH^-

The Au-Cl and Au-OH adlayers formed on the MLagg exhibit distinct interfacial dipoles that modulate the surface electronic structure that strongly influence molecular binding and nanogap stabilization. Au-Cl, formed under anodic potentials in the presence of Cl^-, generates a covalent-like surface dipole that raises the work function and produces a near-neutral interfacial field. In contrast, Au-OH adlayers formed under mildly oxidizing alkaline conditions are more ionic and disordered in nature. The resulting interface presents a more negatively charged and hydrated surface, which raises the work function to a lesser extent and introduces an electrostatically repulsive field. These properties hinder CB[5] access to the nanogap and often lead to incomplete re-scaffolding and structural degradation over repeated cycles.

We have previously shown that the Au-Cl and Au-OH adlayers can be formed and subsequently reduced electrochemically; we also find that these adlayers can be modulated through solution-phase chemical changes. We investigated the effect of adding hydroxide (OH^-) ions to Au-Cl-coated MLagg substrates held under open-circuit conditions in phosphate buffer (**Supplementary Figure 43a**). Initially, the system stabilizes at an OCP between +0.5 and +0.7 V depending on the applied anodic potential (+0.5 or +0.7 V). The high stabilised OCP reflects the presence of the Au-Cl adlayer, confirmed by SERS in a stable high intensity Au-Cl peak at 260 cm^{-1} (**Supplementary Figure 43b**). Upon addition of a first aliquot of NaOH, the OCP dropped gradually to approximately +0.3 V. At this stage, the SERS spectrum showed partial loss of the Au-Cl signal alongside the emergence of a broad band at ~400 cm^{-1}, attributable to the formation of an Au-OH adlayer. Following the addition of a second NaOH aliquot, the OCP dropped further to ~0 V, accompanied by a more complete loss of the Au-Cl peak and strengthening of the Au-OH signature. This second transition was marked by further evolution in the CO peak Stark shift, indicating a more negative surface field consistent with a Au-OH interface. In parallel, the CB[5] spectral profile shifted significantly, with the previously suppressed lower wavenumber C-H peaks re-emerging. This spectral reorganization confirms a substantial transformation of the surface environment both in terms of interfacial electrostatics and local binding configurations.

We also repeated these measurements on Au-Cl adlayers formed in 0.1 mM CB[5] in 100 mM HCl at pH 1 (**Supplementary Figure 44**). Similar behaviour was observed, though with distinct pH effects. The initial OCP of +0.7 V decreased sharply to 0 V following the first NaOH addition, accompanied by an immediate loss of the Au-Cl peak and appearance of the Au-OH band. However, within tens of seconds, the Au-Cl SERS peak re-emerged while the Au-OH band diminished, and the OCP rose slightly, stabilizing near +0.4 V. This reversal likely reflects the higher proton activity in HCl, which neutralizes OH^- and restores the conditions favourable for Cl^- rebinding. A second NaOH addition overcame this neutralisation, resulting in a stable OCP near 0 V and a final surface spectrum characterized by the disappearance of Au-Cl and restoration of the Au-OH band. Notably, all NaOH additions were not enough to significantly neutralise all protons, as the pH of the bulk solution remained at pH 2.

In both buffered and acidic environments, the addition of OH^- destabilizes the Au-Cl adlayer through direct ligand exchange and competition for surface sites. This destabilization manifests as a sharp drop in OCP and a loss or weakening of the Au-Cl SERS band, accompanied by the appearance of features attributable to Au-OH. These spectral and electrochemical signatures reflect the collapse of the Au-Cl layer and a corresponding shift in the interfacial field to a more negatively charged environment dominated by hydroxide binding or exposed Au(0) sites. Crucially, these changes are not confined to the surface species themselves; they result in changes to the local electronic structure, reorganizing adsorbed molecules such as CB[5] and modulating vibrational Stark shifts in co-adsorbed CO. The resulting patterns closely mirror those observed during electrochemical modulation of the adlayer, confirming that the underlying changes in dipole structure and work function are qualitatively similar whether triggered by applied potential or solution chemistry. These observations highlight the central role of surface dipoles in governing interfacial properties and demonstrate that electronic properties of AuNP surfaces can be tuned through both electrochemical and solution-based control.

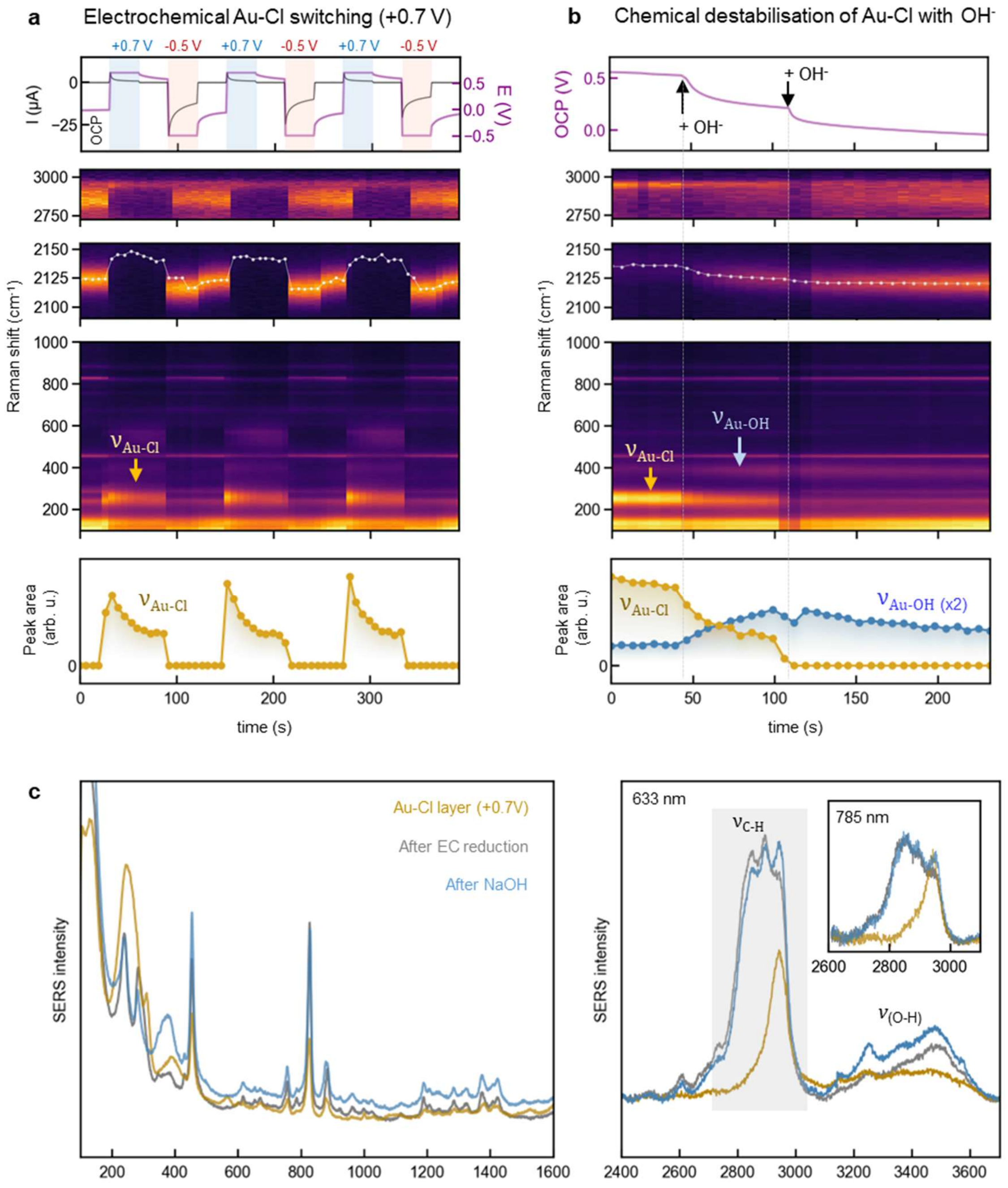

Supplementary Figure 43 | Electrochemical formation of Au-Cl (in buffer) and its destabilisation with OH^-. (**a**) Time-series current, potential, and SERS spectra of MLagg-CB[5] undergoing potential switching between anodic (+0.7 V) and reducing potentials (-0.5 V) in 0.1 mM CB[5] and 50 mM potassium phosphate buffer (pH 7.0). Between each applied potential, the system was allowed to relax to OCP. Wavenumber ranges of interest are highlighted in the time-series SERS spectra: the high wavenumber CB[5] C-H stretching modes, the carbon monoxide (C≡O) stretching mode with the peak position tracked (white line), and the lower wavenumber range showing CB[5] signature vibrations and the Au-Cl or Au-OH stretching modes. The peak area of the Au-Cl stretching mode at 240-270 cm^{-1} is tracked per SERS spectrum. (**b**) An MLagg-CB[5] with an electrochemically formed Au-Cl adlayer (+0.7 V) in 0.1 mM CB[5] and buffer is treated with aliquots of 1 M NaOH. Time-series SERS spectra and OCP measurements were tracked, and Au-Cl and Au-OH peak areas extracted per SERS spectrum. (**c**) SERS spectra of MLagg-CB[5] after adlayer formation, reduction, and adlayer destabilisation with NaOH.

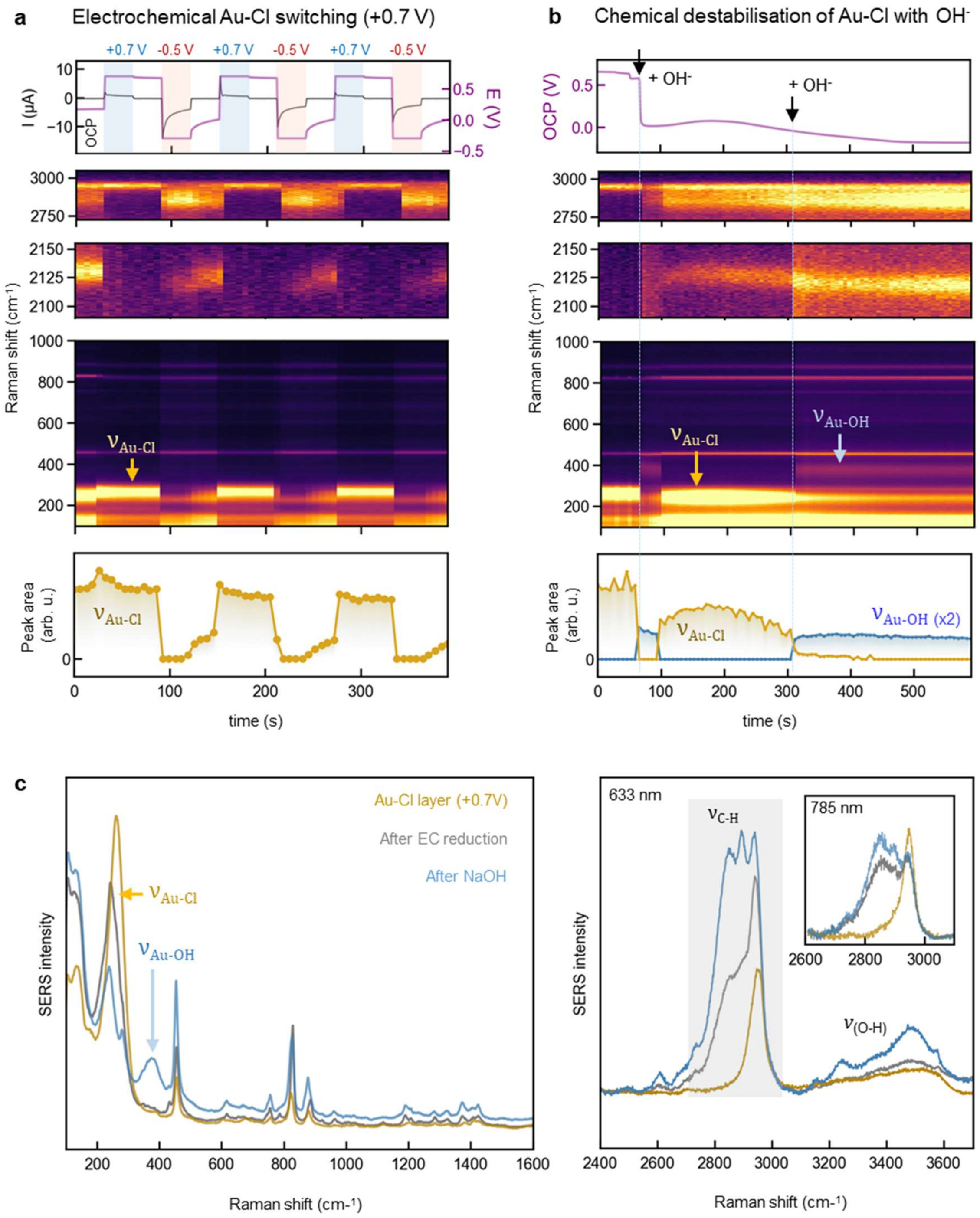

Supplementary Figure 44 | Electrochemical formation of Au-Cl (in HCl) and its destabilisation with OH^-. (**a**) Time-series current, potential, and SERS spectra of MLagg-CB[5] undergoing potential switching between anodic (+0.7 V) and reducing potentials (-0.5 V) in 0.1 mM CB[5] and 100 mM HCl (pH 1). Between each applied potential, the system was allowed to relax to OCP. Wavenumber ranges of interest are highlighted in the time-series SERS spectra: the high wavenumber CB[5] C-H stretching modes, the carbon monoxide (C≡O) stretching mode, and the lower wavenumber range showing CB[5] signature vibrations and the Au-Cl or Au-OH stretching modes. The peak area of the Au-Cl stretching mode at 240-270 cm^{-1} is tracked per SERS spectrum. (**b**) An MLagg-CB[5] with an electrochemically formed Au-Cl adlayer (+0.7 V) in 0.1 mM CB[5] and HCl is treated with aliquots of 1 M NaOH. Time-series SERS spectra and OCP measurements were tracked, and Au-Cl and Au-OH peak areas extracted per SERS spectrum. (**c**) SERS spectra of MLagg-CB[5] after adlayer formation, reduction, and adlayer destabilisation with NaOH.

Supplementary Note 14. Surface Transformations in Ch-ReSERS

The kinetics of EC-ReSERS demonstrate that oxidation and reduction pathways are highly dependent on Cl^- availability, pH, and mass-transfer effects, all of which influence the transformation between Au(III)-Cl, Au-Cl, and Au-O species. These same factors also govern the behaviour of Ch-ReSERS, where chemical treatment with CB[5], acid, and Cl^- drives the conversion of surface-bound Au oxide into Au(III)-Cl and subsequently Au-Cl species, in the absence of an externally applied potential. This section investigates the chemical pathways of Ch-ReSERS across a range of Cl^- concentrations and pH values and further characterizes the full sequence of transformations involved: from initial oxide dissolution to the formation of a metastable Au-Cl-stabilized state, its spontaneous collapse, and the eventual regeneration of CB[5]-stabilized nanogaps. Time-resolved spectro-electrochemical data are used to track these stages and provide mechanistic insight into the interfacial chemistry that governs nanogap re-scaffolding.

Role of H^+ and Cl^- in the intermediate stage of Ch-ReSERS

After oxidative cleaning and the concomitant formation of a Au oxide layer on the MLagg, it was previously demonstrated[35] that the nanogaps can be re-scaffolded upon incubation with CB[5] and an acid such as HCl or H_2SO_4. However, the chemistry underlying the process has not yet been fully elucidated. To investigate this process, time-resolved SERS and open-circuit potential (OCP) measurements during chemical re-scaffolding have revealed the changes in the nanogap chemistry as the MLagg transforms from an oxide-coated surface to one regenerated with CB[5] scaffolds (see **Supplementary Table 2** for chemical reactions).

Time-series SERS spectra and OCP measurements of oxidised MLaggs treated with 1 mM CB[5] and 0.5 M HCl or 0.5 M H_2SO_4 are shown in **Supplementary Figure 45**. While the intermediate stages show differences in the kinetics of the transformation, there are also notable similarities in the intermediate species involved in both processes. In both cases, the decrease in the Au oxide band centred at 590 cm^{-1} (blue lines in **Supplementary Figure 45**) coincides with the appearance of peaks at ~345/365 cm^{-1} (orange line) corresponding to Au(III)-Cl stretching vibrations (**Supplementary Figure 3** and **Supplementary Table 1**). These changes are followed by the gradual appearance of CB[5] (826 cm^{-1}) and Au-Cl lines (265 cm^{-1}). In the MLaggs treated with CB[5]-HCl, the Au-O band decreases rapidly, and the OCP also immediately drops from an initial 0.9-1.0 V (indicating the surface is dominated by Au oxide) to 0.7 V, which is consistent with the formation of a Au-Cl adlayer[95]. MLaggs treated with CB[5]-H_2SO_4, on the other hand, show slower decreases in the Au-O lines along with a slow rise in Au(III)-Cl. It should be noted that the CB[5] reagent contains HCl and KCl from crystallisation, so the solution of CB[5]-H_2SO_4 also contains ~5 mM Cl^-. During the transition, weak (bi)sulfate ion peaks at ~1000 cm^{-1} are also observed, but the prominence of the Au(III)-Cl peak (despite the relatively low concentration of Cl^- in the system), along with its simultaneous rise with the decrease in the Au-O band suggest that the transition process is dominated by the interaction of Cl^- with Au oxide. This is consistent with previous work investigating the decomposition of Au oxide with electrolytes, where it was demonstrated that Au oxide decomposes only after treatment HCl and not H_2SO_4.[96] However, since chloride ions are in a limiting concentration, the decomposition of Au oxide occurs more slowly than in treatments with excess Cl^-. The OCP during the transition also remains at 1.0 V, suggesting that the surface still contains a substantial amount of Au oxide relative to Au-Cl species. Similar behaviour is observed when using another acid such as $HClO_4$.

Aside from chloride ions, acidic conditions also facilitate the Au oxide dissolution process. Treatment of an oxidised MLagg with CB[5] and excess Cl^- in neutral conditions (0.5 M NaCl) also demonstrates decomposition of Au oxide with a rise in Au(III)-Cl, but at a slower rate. To systematically study the effect of pH and Cl^-, oxidised MLaggs were treated with 1 mM CB[5] and different solutions of two pH levels (pH 1.00 and pH 2.85 using $H_3PO_4/H_2PO_4^-$) and two chloride ion levels ([Cl^-] = 0.1 M and 0.5 M). At a fixed pH of 2.9 (**Supplementary Figure 46**), the Au oxide decomposition rate is generally slow, with the various Au-O bands evolving during the intermediate stage. At lower [Cl^-], the three main Au-O bands decrease to a plateau, while at higher [Cl^-], the 520 and 660 cm^{-1} Au-O bands decrease at a relatively faster rate, leaving the 550 cm^{-1} line as the remaining peak, which has also been attributed to Au-OH bond vibrations[17]. At lower pH and at both levels of [Cl^-] (**Supplementary Figure 47**), there is a similar rapid decrease in the 520 and 660 cm^{-1} Au-O lines, with the 550 cm^{-1} line remaining throughout the intermediate stage. The combined results suggest that protons catalyse Au oxide decomposition,

likely through the protonation of oxide lattice O^{2-} to OH^-, indicated by the remaining 550 cm^{-1} line during the intermediate stage[17]. Further protonation can lead to the release of OH^- as H_2O [17]. Looking more closely at the Au(III)-Cl line, there are two peaks at 345 cm^{-1} and 365 cm^{-1}, with the latter indicating that Au(III) is also coordinated to OH^-. This suggests incomplete release of OH^- despite the highly acidic conditions, possibly from the local depletion of protons from the initial protonation of O^{2-}, especially considering the solution remains unstirred throughout the re-scaffolding process.

Stages and Mechanism of Ch-ReSERS

Upon treatment of an oxidised MLagg with CB[5] and 0.5 M HCl (**Supplementary Table 2**), a high local concentration of Au(III)-Cl species is generated at the surface, arising from the chemical dissolution of the pre-formed Au oxide layer. The Au(III)-Cl species can follow two parallel pathways: (1) they can desorb from the surface as a soluble complex such as [$AuCl_4^-$] to be integrated into the EDL or diffuse away into the bulk solution, or (2) stay adsorbed to the surface to undergo comproportionation with exposed Au(0) atoms to form an Au-Cl adlayer. Initially, the second pathway appears to dominate, as we observe a rapid rise in the Au-Cl SERS peak (265 cm^{-1}) and a stabilisation of the OCP to +0.7 V, accompanied by a simultaneous decrease in the Au(III)-Cl peak at 340 cm^{-1}. These spectral changes are consistent with comproportionation of Au(III)-Cl to form Au-Cl. A weak CB[5] signal also becomes detectable at this stage, suggesting initial binding of CB[5] onto a limited number of exposed binding sites or regions with favourable electrostatics at the Au-Cl interface. High wavenumber C-H lines of bound CB[5] at this stage show the characteristic enhancement of the high C-H line associated with binding at the Au-Cl environment (**Supplementary Figure 54**).

After initial dissolution, the system enters a metastable stage where the OCP remains at +0.7 V. The observed stability of the intermediate state in Ch-ReSERS may be attributed to a surface dipole formed by the Au-Cl adlayer, which elevates the work function and thus maintains a high OCP. However, unlike in electrochemical systems where Au-Cl can be uniformly formed by anodic oxidation on a pre-coated CB[5] surface, the Au-Cl layer in Ch-ReSERS is generated *in situ* via comproportionation between surface Au(0) and Au(III)-Cl species generated from the chemical dissolution of surface oxide. In this case, CB[5] is introduced simultaneously with HCl and competes for binding with Cl^- as the adlayer forms. Consequently, the resulting Au-Cl interface is more heterogeneous and more dynamic. Indeed, SERS data suggests it is not static; rather, the slow but steady rise in the Au-Cl peak during the intermediate stage suggests that the interface is sustained by continued reaction of Au(III)-Cl with surface Au(0) to generate Au-Cl. This redox balance maintains the interfacial electronic structure in a high work function state.

SERS and electrochemical data reveal that this intermediate state can abruptly collapse. In multiple experiments (**Supplementary Figure 45-47**), a shift in the Au-Cl peak from 265 cm^{-1} to 240 cm^{-1} occurs within seconds, coinciding with a rapid OCP drop toward 0 V and the reappearance of CB[5] spectral signatures (**Supplementary Figure 48**). This behaviour suggests a fast, cooperative transformation of the surface, where the Au-Cl surface dipole collapses and triggers both the electronic potential and interfacial chemical structure to reorganize. While the precise trigger for the transition remains not fully clarified, closer inspection of the onset of the transition reveals a distinct sequence: as the OCP exhibits an initial sharp drop from +0.7 V to +0.6 V, the Au(III)-Cl peak diminishes rapidly while the Au-Cl peak rapidly rises. As the Au(III)-Cl is nearly depleted, Au-Cl reaches in maximum intensity while the OCP drops further to 0.5 V. At this point, the Au-Cl peak red shifts and decreases in intensity while the OCP continues to drop further to 0 V.

The initial sharp drop in OCP from +0.7 V to +0.6 V, where there is a rapid change in Au(III)-Cl and Au-Cl, marks a critical point. This point marks the start of a burst of rapid comproportionation, where Au(III)-Cl reacts with Au(0) sites to form Au-Cl. Notably, the driving factor behind this does not appear to be the overall depletion of local Au(III)-Cl species through diffusion to the bulk. UV-Vis measurements, while inherently limited in sensitivity, show that $AuCl_4^-$ is detectable in the bulk (within ~3 mm from the MLagg surface, and at >1 μM) only after the OCP drop (**Supplementary Figure 50**). This suggests that $AuCl_4^-$ is likely confined near the interface during the intermediate stage and only becomes fully diffusible once the surface structure destabilizes. Although we cannot fully rule out the presence of $AuCl_4^-$ at concentrations below the UV-Vis detection limit during the earlier stage, the observed signal surge after the OCP drop nonetheless supports the idea of $AuCl_4^-$ release. The local

enrichment of $AuCl_4^-$ near the surface during this stage likely occurs since the Au-Cl adlayer generates a slightly net positive surface, therefore electrostatically attracting $AuCl_4^-$ in its EDL.

As $AuCl_4^-$ is still locally available, the likely driving factor behind the transition point is the formation of a critical Au-Cl adlayer density. The Au-Cl adlayer acts as an electron-withdrawing overlayer, depleting the underlying Au surface of electron density and raising the work function, which stabilizes the system at +0.7 V. However, as the adlayer density increases, electrostatic repulsion between neighbouring Au-Cl units intensifies. While the adlayer initially accommodates this repulsion through electro-compressibility, allowing local rearrangement or bond-length adjustments to alleviate strain[73,91], this capacity eventually saturates at a critical density. The instability can be exacerbated by the presence of other species at the surface, such as CB[5] and Au(III)-Cl. At this point, further increases in coverage or local strain (exacerbated by surface heterogeneities such as steps, defects, or grain boundaries) can destabilize the adlayer structure. The electronic and structural instability will make it favourable for electron transfer from the underlying Au(0) to Au-Cl. This local reduction event collapses the surface dipole, resulting in a local change in work function that immediately propagates across the entire Au surface via electronic conduction. As a result, a global shift in OCP occurs from +0.7 V to 0.6 V, even though the initiating event was local in nature. The decreased work function facilitates rapid electron transfer from Au(0) to any remaining surface-bound Au(III)-Cl, triggering a comproportionation cascade that accelerates Au-Cl formation.

Once the Au-Cl SERS intensity reaches its maximum, the surface has effectively exhausted the supply of surface Au(III)-Cl, leaving the adlayer at its densest and most electron-withdrawing state. At this point, although the Au-Cl SERS intensity reaches a peak, the OCP does not stabilize but instead continues to decrease rapidly, reflecting the dynamic instability of this high-coverage Au-Cl state. The steep decline in OCP indicates that the adlayer can no longer maintain its electronic structure, and electron transfer from the underlying Au(0) to the Au-Cl adlayer itself becomes thermodynamically favourable. This final reduction cascade collapses the Au-Cl adlayer, marked by a rapid decrease and red shift of the Au-Cl SERS line, and a continued drop in OCP toward 0 V, and the increase of CB[5] signals indicating nanogap re-scaffolding. Concurrently, in-situ dark-field scattering measurements reveal a blue-shift of the chain mode plasmon peak from 810 to 795 cm^{-1} and an increase in scattering intensity from 5.2% to 5.6%, consistent with a more metallic surface emerging at the end of the OCP drop (**Supplementary Figure 49**).

During the Au-Cl layer collapse, the local Au surface becomes transiently electron-deficient due to the preceding electron transfer that reduced the Au-Cl adlayer. This local positive polarization enhances the electrostatic attraction between the Au surface and the electronegative carbonyl portals of CB[5], facilitating rapid rebinding and stabilization of the surface. This immediate ligand rebinding helps re-establish the nanogap structure and completes the regeneration cycle.

Overall, the intermediate stage of Ch-ReSERS represents a metastable, high-OCP state maintained by the Au-Cl adlayer. This adlayer not only establishes a surface dipole that elevates the local work function but also actively involved in surface redox reactions. At critical adlayer density, electrostatic repulsion and strain induce electronic instability, triggering local electron transfer that propagates rapidly across the Au surface via electronic conduction. This global reduction cascade collapses the Au-Cl adlayer and reorganizes both the surface chemistry and electronic potential, enabling rapid CB[5] rebinding. These insights establish the Au-Cl adlayer as a dynamic, redox-active surface feature that facilitates the regeneration cycle through its capacity to modulate local electron density, surface potential, and ligand interactions.

Reducing agents

To regenerate an oxidised MLagg with CB[5] by chemical means, an alternative approach to CB[5]-HCl treatment is the direct addition of reducing agents such as ascorbic acid (AA) and $NaBH_4$. **Supplementary Figure 51a,b** show the time-series SERS spectra of an oxidised MLagg-CB[5] before and after the addition of CB[5]+reducing agent at *t*=0 s. Immediately after the addition of the reducing agent, the Au-O peaks centred at 590 cm^{-1} decrease with the simultaneous increase in CB[5] peaks (826 cm^{-1} and 451 cm^{-1}). No appreciable Au-Cl intermediates were detected, suggesting that the reducing agents act directly on Au oxide. OCP measurements also show a rapid

decrease in potential, dropping almost immediately to ~0 V after the addition of CB[5]-AA, while the potential settles to -1.0 V with CB[5]-$NaBH_4$, highlighting the highly reducing conditions of the solution.

The SERS, DF, and XPS spectra of the MLaggs rescaffolded using different methods (CB[5]-HCl, -AA, or -NaBH4) are shown in **Supplementary Figure 51c-g.** While the SERS spectra of all rescaffolded MLaggs feature the characteristic CB[5] peaks at 826 cm^{-1} and 450 cm^{-1}, the spectra of the MLaggs regenerated with reducing agents feature additional peaks related to adsorbed oxygen species (670 cm^{-1}, 520 cm^{-1}). The Au 4f spectra of the MLaggs suggest all Au oxide is reduced to metallic Au. The O 1s spectra of the MLaggs regenerated with reducing agents indicate that different adsorbed oxygen species are indeed on the surface, consistent with the SERS spectra. Previous work investigating the decomposition of Au oxides on planar Au substrates also report the presence of residual adsorbed oxygen species after the chemical decomposition of oxides [97].

DF scattering spectra of the MLagg as prepared and after cleaning and re-scaffolding using either HCl, AA or $NaBH_4$ show that the regenerated MLaggs exhibit decreases in the plasmonic chain mode amplitude. However, the MLaggs regenerated using reducing agents exhibit the greatest decreases in scattering intensity, from 22% for $NaBH_4$ and 58% for AA, compared to 11% when using HCl. The rapid rate at which Au oxide reduction occurs upon addition of the reducing agent relative to the diffusion of CB[5] into the nanogaps can result in more pronounced sintering. Thus, repeated regeneration using this approach is not viable.

Supplementary Table 2 | Summary of Ch-ReSERS stages. Key spectro-electrochemical observations were tracked per growth stage, with the SERS peak position and intensity changes noted. (Arrows indicate changes in peak intensity: (↑) intensity increase, (↓) intensity decrease, (–) no change in intensity, (↑↑) sharp increase, (↓↓) decrease to become undetectable). For simplicity, the primary Au(III)-Cl species is denoted as $AuCl_3$, although dimers (Au_2Cl_6) or $AuCl_4^-$ complexes can also exist. Au(III) can also be coordinated with a mix of Cl^- and OH^-

**Stage**		**Observations**					**Reaction / Dominant surface species**
		**SERS**				**OCP**	
		**Au-O**	**Au(III)-Cl**	**Au-Cl**	**CB[5]**		
Oxidised MLagg		Broad,<br>520, 560, 660 cm^{-1}				0.9 V	Au_2O_3
Treat with CB[5], HCl		↓<br>(w) 550 cm^{-1}	↑<br>340, 365 cm^{-1}	↑<br>265 cm^{-1}	↑<br>826 cm^{-1}	0.9-<br>0.7 V	**Chemical dissolution of Au oxide and redox of Au(III)-Cl species (represented here primarily as $AuCl_3$)**<br>$Au_2O_3 + 6HCl \rightarrow 3H_2O + 2AuCl_{3(ads)}$ or<br>$Au_2O_3 + 8HCl \rightarrow 3H_2O + 2HAuCl_{4(ads)}$<br>[$\nu_{Au(III)\text{-}Cl}$ = 345 cm^{-1}]<br>*Partial hydrolysis or incomplete ligand exchange:*<br>$AuCl_3 + OH^- \rightarrow Au\text{-}Cl_2(OH)^-$<br>[$\nu_{Au(III)\text{-}Clx(OH)y}$ 365 cm^{-1}, 550 cm^{-1}]<br>*Comproportionation*: $AuCl_3 + Au(0) \rightarrow 2Au\text{-}Cl + Cl^-$<br>[$\nu_{Au\text{-}Cl}$ 265 cm^{-1}]<br>CB[5] binds to limited free Au(0) sites
Intermediate stage	I	– 550 cm^{-1}	↓<br>340, 365 cm^{-1}	↑<br>265 cm^{-1}	– 826 cm^{-1}	0.7 V	**Surface $AuCl_3$ decreasing from comproportionation**<br>*Comproportionation*: $AuCl_3 + Au(0) \rightarrow 2Au\text{-}Cl + Cl^-$<br>[$\nu_{Au\text{-}Cl}$ 270 cm^{-1}]
	II	– 550 cm^{-1}	**near plateau**<br>340, 365 cm^{-1}	**near plateau**<br>265 cm^{-1}	– 826 cm^{-1}	0.7 V	
Transition stage, t_d (OCP drop)	I	– 550 cm^{-1}	↓↓ 340 cm^{-1}<br>– 365 cm^{-1}	↑↑<br>265 cm^{-1}	– 826 cm^{-1}	0.7-<br>0.5 V	**$AuCl_{3(ads)}$ depleted, now primarily $AuCl_2OH_{(ads)}$**<br>*Comproportionation*: $AuCl_2OH + Au(0) \rightarrow 2Au\text{-}Cl + OH^-$
	II	↓↓<br>550 cm^{-1}	↓↓<br>365 cm^{-1}	↓<br>265 → 240 cm^{-1}	↑<br>826 cm^{-1}	0.5-<br>0.2 V	**Surface Au(III) species (Au(III)-Cl and Au-O) depleted**<br>*Comproportionation*: $AuCl_2OH + Au(0) \rightarrow 2Au\text{-}Cl + OH^-$<br>*Hydrolysis*: $AuCl_2OH + OH^- \rightarrow AuCl(OH)_2$<br>**Surface now primarily Au(0) and Au-Cl (unstable)**<br>**CB[5] competitively starts to bind to Au(0) patches, decreasing Au-Cl coverage (265 → 240 cm^{-1})**
	III			↓<br>240 cm^{-1}	↑<br>826 cm^{-1}	0.2-<br>0 V	**Au-Cl coverage decreases further, CB[5] continues to bind to free Au(0) sites**
Post-transition				– 240 cm^{-1}	– 826 cm^{-1}	0 V	**Equilibrium Au-Cl and CB[5] binding**

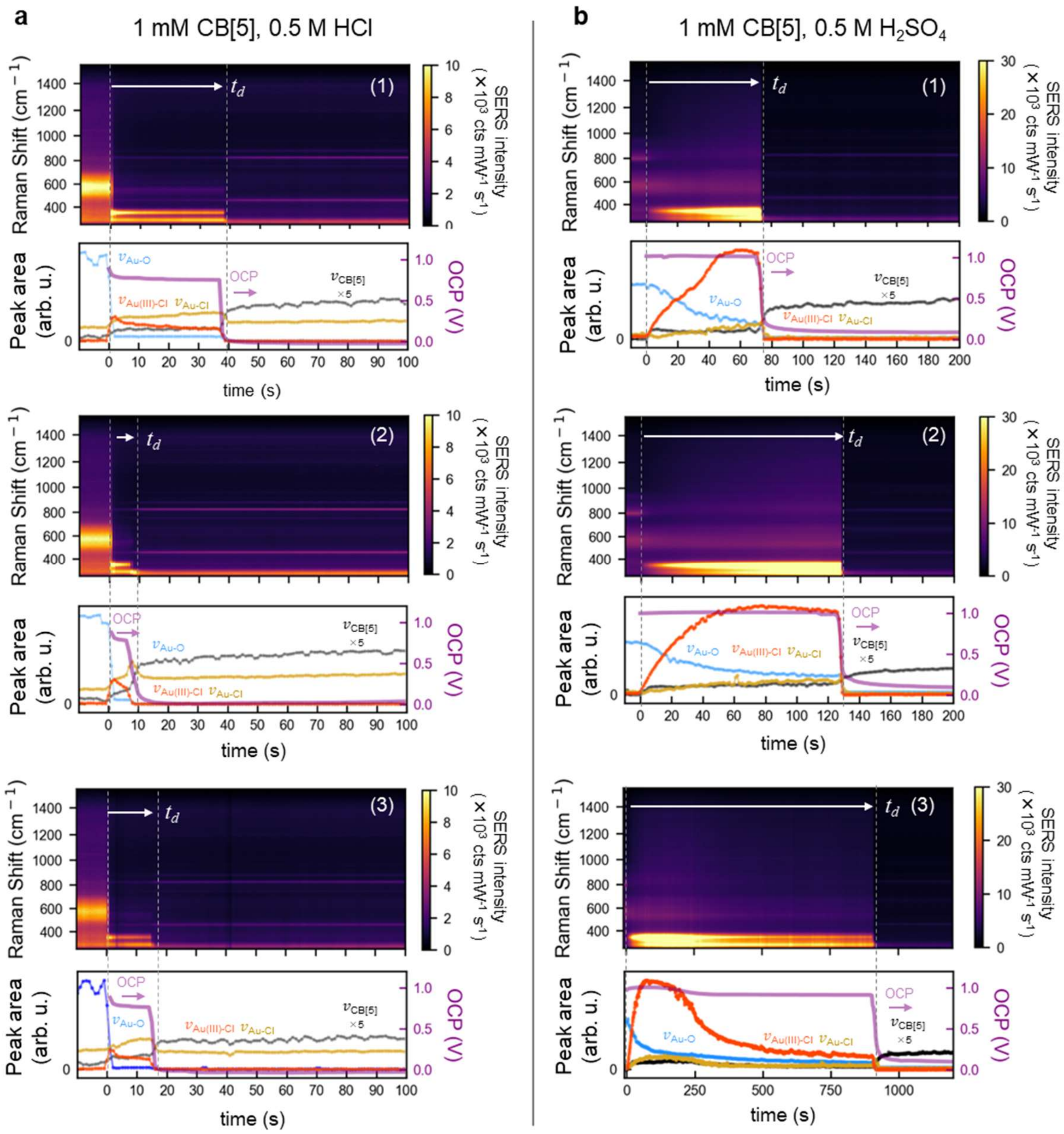

Supplementary Figure 45 | Replicate time-series SERS and OCP measurements of MLagg-CB[5] during Ch-ReSERS with HCl and H_2SO_4. (**a,b**) Triplicate (1-3) time-series SERS spectra from MLagg-CB[5] initially oxidised with 45 min oxygen plasma cleaning, followed by incubation with 100 µL solution of (**a**) 1 mM CB[5] in 0.5 M HCl (pH 0.3) or (**b**) 1 mM CB[5] in 0.5 M H_2SO_4 (pH 0.3) at t=0. At the onset of solution incubation, the open-circuit potential (OCP, vs. Ag/AgCl) was monitored along with SERS. The peak areas of Au-O lines (summation of 520, 590, 660 cm^{-1} lines, blue), CB[5] (826 cm^{-1}, black), Au(III)-Cl (345 cm^{-1}, orange), and Au-Cl (240-270 cm^{-1}, yellow) were tracked per time-series SERS spectrum. Note the different time scales between (**a**) and (**b**). (**b3**) is also on a different time scale from (**b1,b2**).

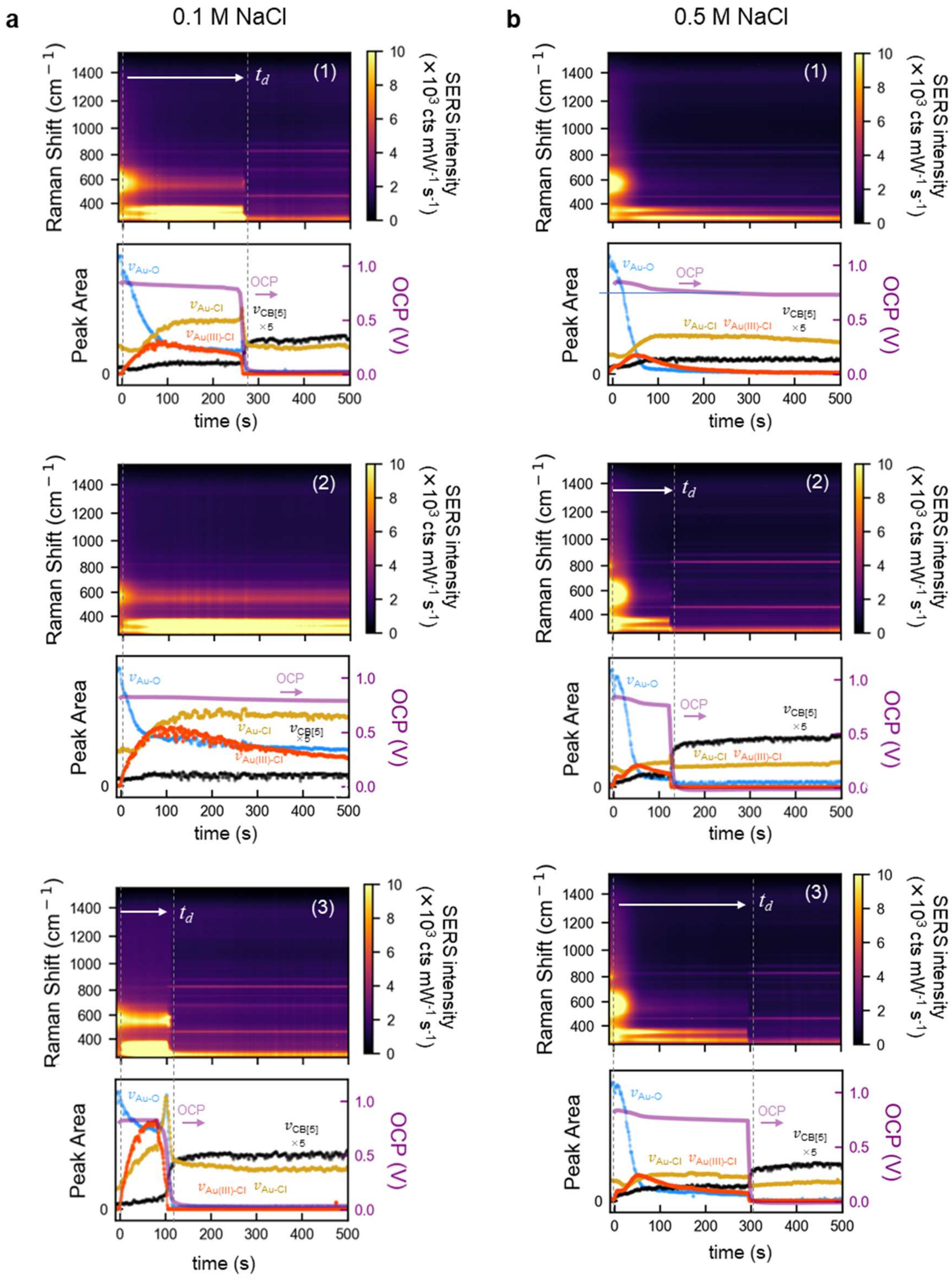

Supplementary Figure 46 | Replicate time-series SERS and OCP measurements of MLagg-CB[5] during Ch-ReSERS at pH 2.9 (0.5 M H_3PO_4/KH_2PO_4) with 0.1 M or 0.5 M NaCl. Triplicate (1-3) time-series SERS spectra from MLagg-CB[5] initially oxidised with 45 min oxygen plasma cleaning, followed by incubation with 100 µL solution of 1 mM CB[5] in 0.5 M H_3PO_4-/KH_2PO_4 (pH 2.9) with (**a**) 0.1 M NaCl or (**b**) 0.5 M NaCl at t=0. At the onset of solution incubation, the open-circuit potential (OCP, vs. Ag/AgCl) was monitored along with SERS. The peak areas of Au-O lines (summation of 520, 590, 660 cm^{-1} lines, blue), CB[5] (826 cm^{-1}, black), Au(III)-Cl (345 cm^{-1}, orange), and Au-Cl (240-270 cm^{-1}, yellow) were tracked per time-series SERS spectrum. In some cases, no t_d transition was observed within the time range investigated.

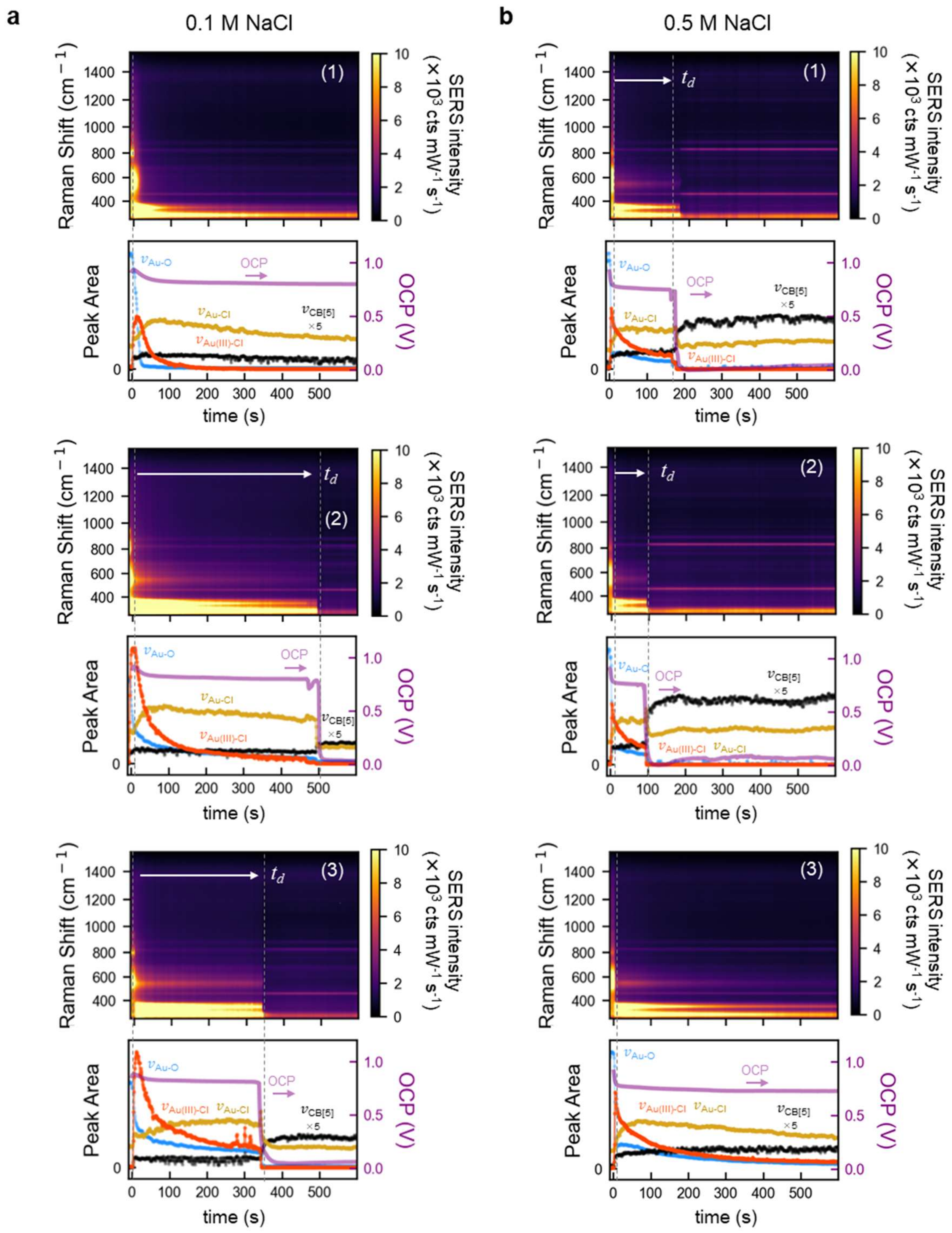

Supplementary Figure 47 | Replicate time-series SERS and OCP measurements of MLagg-CB[5] during Ch-ReSERS at pH 1.0 (0.5 M H_3PO_4) with 0.1 M or 0.5 M NaCl. Triplicate (1-3) time-series SERS spectra from MLagg-CB[5] initially oxidised with 45 min oxygen plasma cleaning, followed by incubation with 100 µL solution of 1 mM CB[5] in 0.5 M H_3PO_4 (pH 1.0) with (**a**) 0.1 M NaCl or (**b**) 0.5 M NaCl at t=0. At the onset of solution incubation, the open-circuit potential (OCP, vs. Ag/AgCl) was monitored along with SERS. The peak areas of Au-O lines (summation of 520, 590, 660 cm^{-1} lines, blue), CB[5] (826 cm^{-1}, black), Au(III)-Cl (345 cm^{-1}, orange), and Au-Cl (240-270 cm^{-1}, yellow) were tracked per time-series SERS spectrum. In some cases, no t_d transition was observed within the time range investigated.

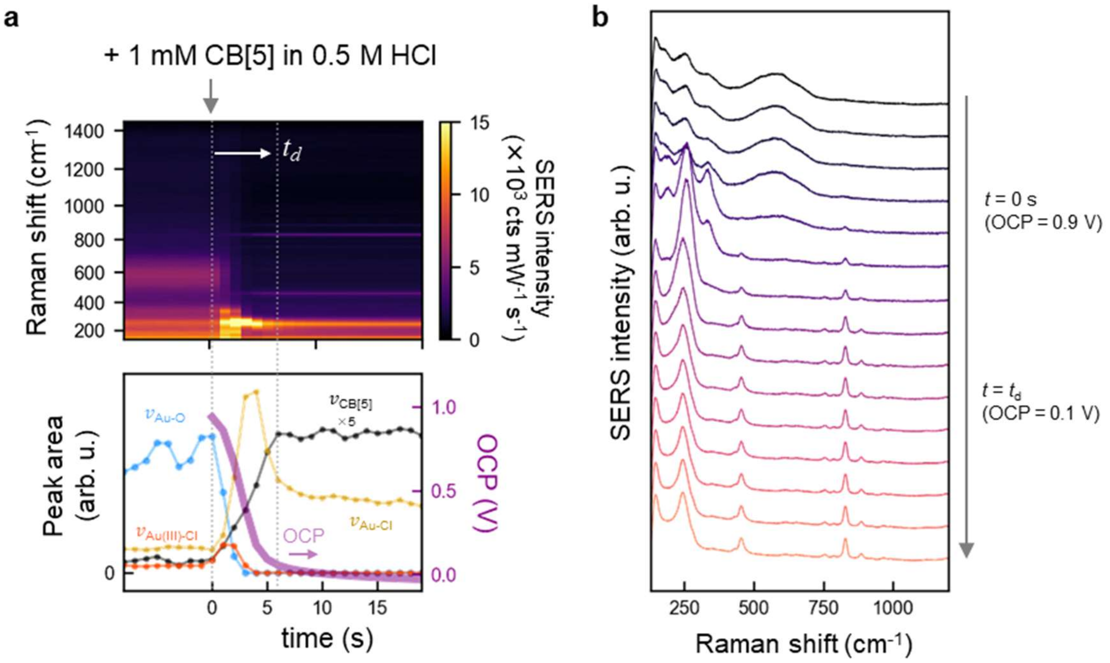

Supplementary Figure 48 | Time-series low wavenumber SERS and OCP measurements of oxidised MLagg-CB[5] during CB[5]-HCl treatment. (**a**) Time-series SERS spectra (1 s integration time, 785 nm excitation laser with 1 mW power) from MLagg-CB[5] initially oxidised with 45 min oxygen plasma cleaning, followed by incubation with 100 µL solution of 1 mM CB[5] in 0.5 M HCl at t=0. At the onset of solution incubation, the open-circuit potential (OCP, vs. Ag/AgCl) was monitored along with SERS. The peak areas of Au-O lines (summation of 520, 590, 660 cm^{-1} lines, blue), CB[5] (826 cm^{-1}, black), Au(III)-Cl (345 cm^{-1}, orange), and Au-Cl (240-270 cm^{-1}, yellow) were tracked per time-series SERS spectrum. (**b**) Time-series SERS spectra near the t_d transition. Spectra are offset for clarity.

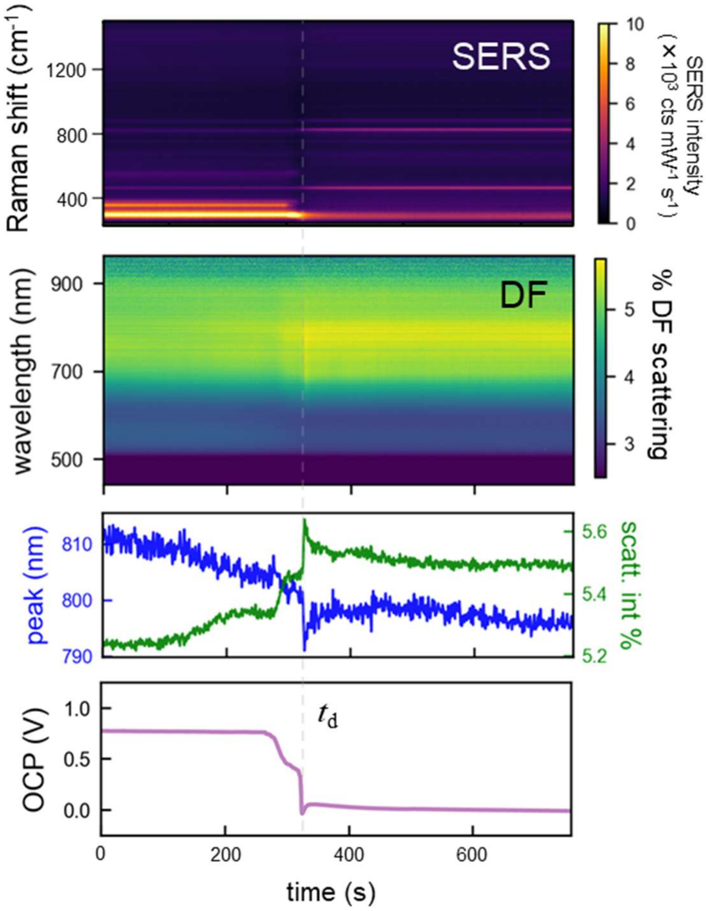

Supplementary Figure 49 | Time-series SERS, DF, and OCP measurements of oxidised MLagg-CB[5] during CB[5]-HCl treatment. Time-series SERS spectra (1 s integration time, 785 nm excitation laser with 1 mW power) and DF spectra (0.5 s integration time) from MLagg-CB[5] initially oxidised with 45 min oxygen plasma cleaning, followed by incubation with 100 µL solution of 1 mM CB[5] in 0.5 M HCl. The MLagg-CB[5] chain-mode peak wavelength position (blue) and amplitude (green) are plotted per DF spectrum with time. Throughout solution incubation, the open-circuit potential (OCP, vs. Ag/AgCl) was also monitored. Due to instability in the time-series DF measurements during injection of the CB[5]-HCl solution, the time frame shown is limited to points near t_d.

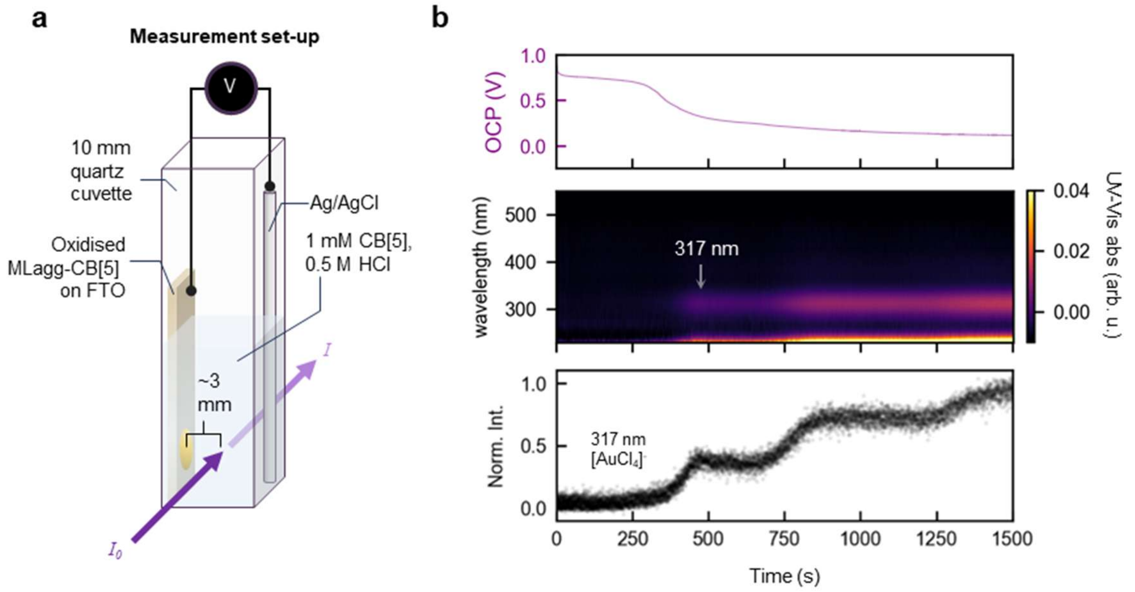

Supplementary Figure 50 | Time-series UV-Vis and OCP measurements of 1 mM CB[5] 0.5 M HCl solution during Ch-ReSERS. (**a**) Spectro-electrochemical measurement set-up. (**b**) Time-series OCP measurements and UV-Vis absorbance of the Ch-ReSERS at a measurement spot ~3 mm away from the oxidised MLagg. The absorbance peak of $AuCl_4^-$ at 317 nm is tracked in the time-series spectrum.

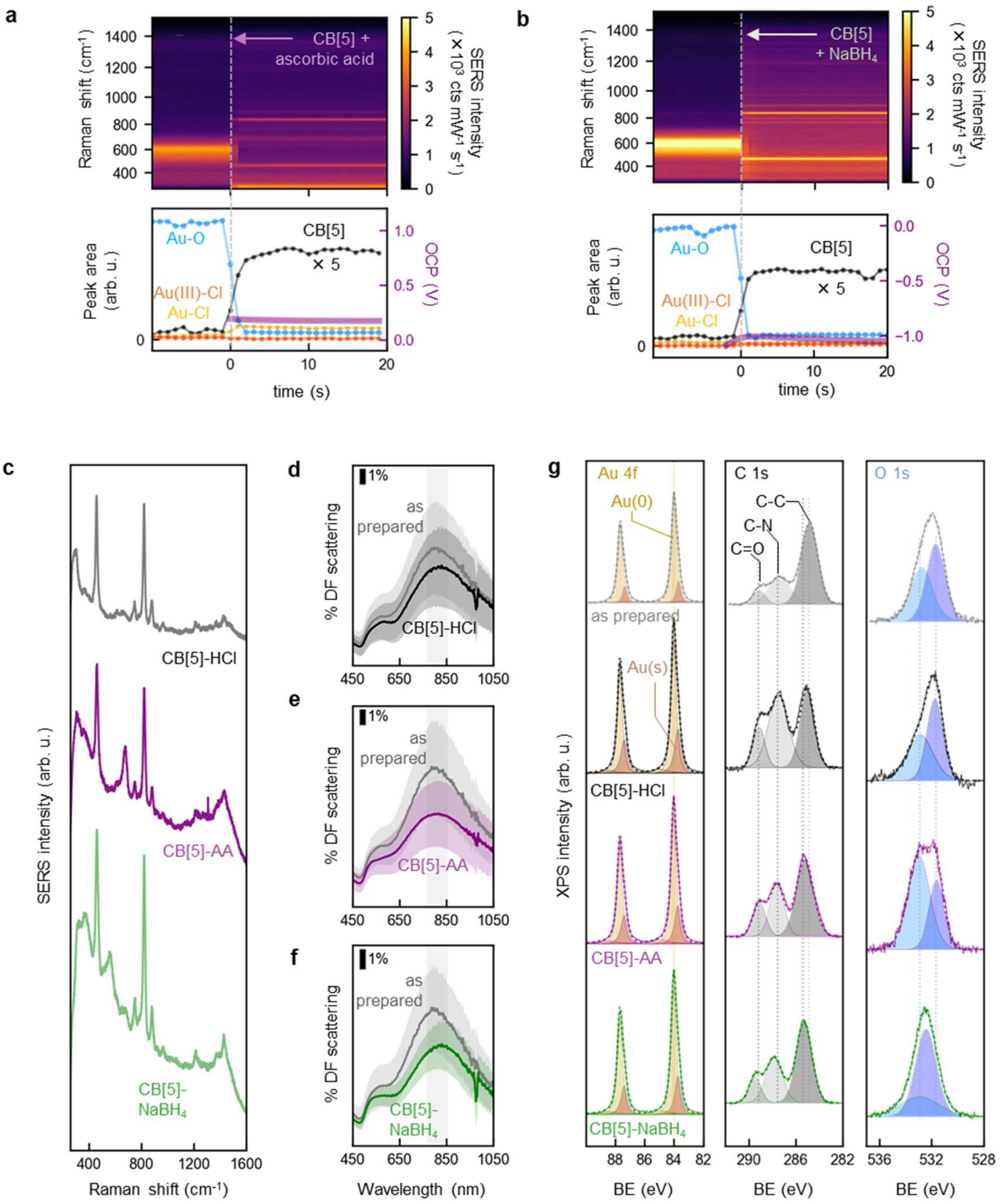

Supplementary Figure 51 | Ch-ReSERS of MLagg-CB[5] with CB[5] and reducing agents. Time-series SERS of oxidised MLagg-CB[5] (45 min O_2 plasma) incubated at t = 0 with 1 mM CB[5] and (**a**) 100 mM ascorbic acid (AA) or (**b**) 35 mM $NaBH_4$. OCP (vs Ag/AgCl) was monitored in parallel. Extracted peak areas of Au-O (520, 590, 660 cm^{-1}, blue), CB[5] (826 cm^{-1}, black), Au(III)-Cl (345 cm^{-1}, orange), and Au-Cl (240-270 cm^{-1}, yellow) are shown. (**c**) SERS spectra of oxidised MLagg-CB[5] regenerated with CB[5]-HCl (black), CB[5]-AA (purple), or CB[5]-$NaBH_4$ (green). DF spectra of MLagg-CB[5] (**d**) as-prepared (grey) and after regeneration with (**e**) CB[5]-HCl (black), (**f**) CB[5]-AA (purple), or (**g**) CB[5]-$NaBH_4$ (green); solid lines and shaded regions represent mean ±1 s.d. of n = 150 spectra across the substrate. (**h**) Au 4f, C 1s, and O 1s XPS spectra (solid lines), fits (dotted lines), and components of MLagg-CB[5] as-prepared and after regeneration with CB[5]-HCl (black), CB[5]-AA (purple), or CB[5]-$NaBH_4$ (green). All XPS spectra normalised to maximum intensity.

Supplementary Note 15. Ch-ReSERS destabilisation with OH^-

To further investigate the interplay between interfacial dipoles and redox dynamics, we examined whether the Au-Cl adlayer formed during Ch-ReSERS could be chemically destabilized by OH^-. In this variant of the Ch-ReSERS experiment, oxidized MLagg substrates were treated with 1 mM CB[5] and 0.5 M HCl to form Au-Cl and Au(III)-Cl stabilized at an OCP of ~+0.7 V. Rather than allowing the system to undergo a spontaneous OCP drop as in standard Ch-ReSERS, we instead perturbed the interface with additions of NaOH. Each addition triggered an immediate and reproducible drop in OCP, accompanied by spectroscopic signatures closely resembling those observed in the unperturbed, spontaneous Ch-ReSERS cascade.

Upon addition of a small amount of NaOH (final bulk pH ~1) (**Extended Data Figure 9**), the Au-Cl band shifted from 260 cm^{-1} to 240 cm^{-1} as the OCP dropped, consistent with a disruption of an ordered adlayer. Simultaneously, the CB[5] signal increased in intensity, while Au-OH bands at 382 cm^{-1} emerged, which is typically not observed in standard Ch-ReSERS where the system transitions directly to Au(0). These effects suggest that even minor additions of OH^- can disrupt the local interfacial equilibrium even as the bulk solution equilibrates to strongly acidic conditions. At the surface, the introduction of OH^- may locally displace Cl^- or coordinate to oxidized Au sites, tipping the equilibrium toward the formation of a Au-OH adlayer, therefore modifying the interfacial field and binding environment around CB[5]. This surface reorganization explains the abrupt spectral and electrochemical transitions observed, despite the still strongly acidic bulk environment. Indeed, while the bulk pH of the solution remains strongly acidic, the interfacial Au-OH species are known to form as intermediates during the oxidation and reduction of Au, even in acidic media[47,98,99] and are likely stabilized via coordination to oxidised Au (i.e., Au(I)-OH or Au(III)-OH). To validate the assignment of the Au-OH peak, use of deuterated analogues (DCl and NaOD) during Ch-ReSERS perturbation (**Supplementary Figure 52**) showed the formation of a peak at 378 cm^{-1}, a 4 cm^{-1} red shift consistent with deuterium substitution (Au-OD).

Interestingly, when smaller aliquots of NaOH were added (**Supplementary Figure 53**), the system exhibited oscillatory behaviour. The first NaOH addition caused an immediate OCP drop (**Supplementary Figure 53**, point 2), loss of Au(III)-Cl and Au-Cl peaks, and the appearance of the Au-OH band alongside an increase in CB[5] intensity. However, over time, the OCP began to rise again, and a reversal in surface speciation occurred (**Supplementary Figure 53**, point 2'): the Au-OH band rapidly disappeared, and the Au-Cl peak re-emerged and shifted back toward 260 cm^{-1}. This process repeated upon a second NaOH addition (**Supplementary Figure 53**, point 3), producing another drop in OCP, a renewed increase in Au-OH intensity, and a shift in the Au-Cl peak to 240 cm^{-1}. Again, a delayed OCP increase followed, accompanied by the decline of Au-OH and the return of high-intensity, higher-frequency Au-Cl features (**Supplementary Figure 53**, point 3').

These oscillations in surface speciation and OCP reflect a dynamic exchange between Cl^- and OH^- at the Au interface, modulated by solution composition and local concentration gradients. Notably, throughout these cycles, the CB[5] intensity remained largely stable following its initial rise after the first OCP drop. This suggests that the CB[5] molecules, once rebound to the nanogap, are not displaced by the ongoing Cl^-/OH^- exchange, but instead experience fluctuations in their local binding environment.

Importantly, these perturbations also provide insight into the fate of residual Au(III)-Cl present before NaOH addition. Although the Au(III)-Cl signal was already decreasing, its complete disappearance following the OCP drop points to an additional redox-driven transformation. The destabilization of the Au-Cl adlayer by OH^- leads to the collapse of the interfacial dipole and a decrease in the surface work function, as reflected in the OCP drop. This shift effectively raises the Fermi level of the AuNP surface relative to the redox potential of Au(III)-Cl, enabling thermodynamically favourable electron transfer from the electrode to the remaining Au(III) species. As a result, Au(III)-Cl is reduced either to Au-Cl or all the way to Au(0), via direct electron transfer or surface-mediated comproportionation. The rapid loss of Au(III)-Cl features thus reflects not just a rearrangement of surface ligands, but a redox activation event made possible by the lowering of the surface work function. This emphasizes how changes in surface dipole structure directly modulate interfacial redox chemistry through Fermi level alignment.

The solution-based destabilisation of Au-Cl reinforces the interpretation that the adlayer governs both the surface dipole and local redox accessibility. Its collapse not only shifts the interfacial field and reorganizes bound ligands but also lowers the work function sufficiently to activate residual redox reactions. This is evident in the rapid disappearance of Au(III)-Cl signals, which likely reflects electron transfer enabled by Fermi level realignment rather than simple ligand exchange. While the spontaneous collapse in standard Ch-ReSERS arises from the collapse of redox equilibrium, the NaOH-triggered collapse demonstrates an extrinsic, chemically induced pathway. In both cases, the destabilisation of the Au-Cl layer initiates a structural and electronic reorganization, marked by OCP drop, CB[5] rebinding, and redox re-equilibration. However, the reversibility observed in the OH-induced case through its oscillatory shifts in Au-Cl and Au-OH speciation has no analogue in the spontaneous system, highlighting the unique ability of OH^- to modulate the surface dipole and redox chemistry in real time via interfacial ligand exchange.

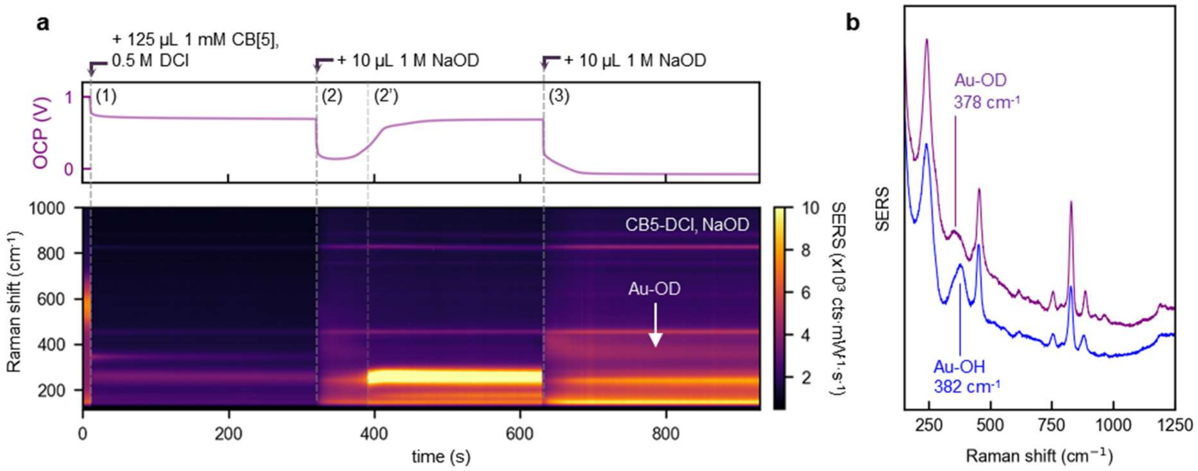

Supplementary Figure 52 | Au-Cl destabilisation by OD^-. (**a**) OCP and time series SERS spectra of an oxidised MLagg-CB[5] treated with 1 mM CB[5] and 0.5 M DCl (pH 1) at point (1) and allowed to equilibrate. After some time, multiple aliquots of 1 M NaOD are dropped into the electrolyte solution at points (2) and (3), resulting in shifts in the equilibrium. A spontaneous transition point occurs at (2'). (**b**) Final SERS spectra of MLagg-CB[5] after CB[5] treatment with DCl and NaOD (purple line) or HCl and NaOH (blue line). The Au-OH/D peak is highlighted.

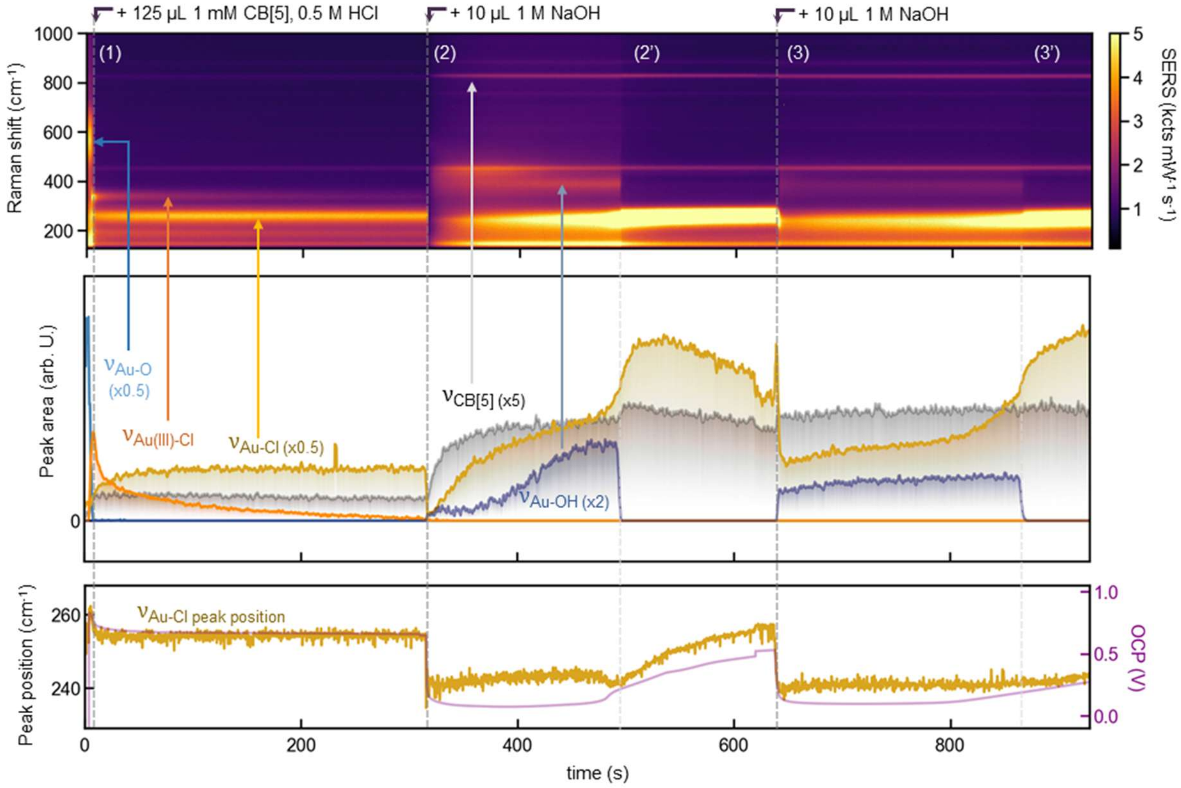

Supplementary Figure 53 | Au-Cl destabilisation by OH⁻ with oscillatory behaviour. Time series SERS spectra of an oxidised MLagg-CB[5] treated with 1 mM CB[5] and 0.5 M HCl at point (1) and allowed to equilibrate. After some time, multiple aliquots of 1 M NaOH are dropped into the electrolyte solution at points (2) and (3), resulting in shifts in the equilibrium. Spontaneous transition points occur at (2') and (3'). The peak areas of CB[5] (826 cm^{-1}, grey line) Au-O (sets of peaks centred at 590 cm^{-1}, blue line), Au(III)-Cl (345 cm^{-1}, orange line), Au-Cl (240-270 cm^{-1}, yellow line), and Au-OH (400 cm^{-1}, dark blue line). The evolution of the peak Au-Cl line is also plotted alongside the measured open-circuit potential evolution (purple line).

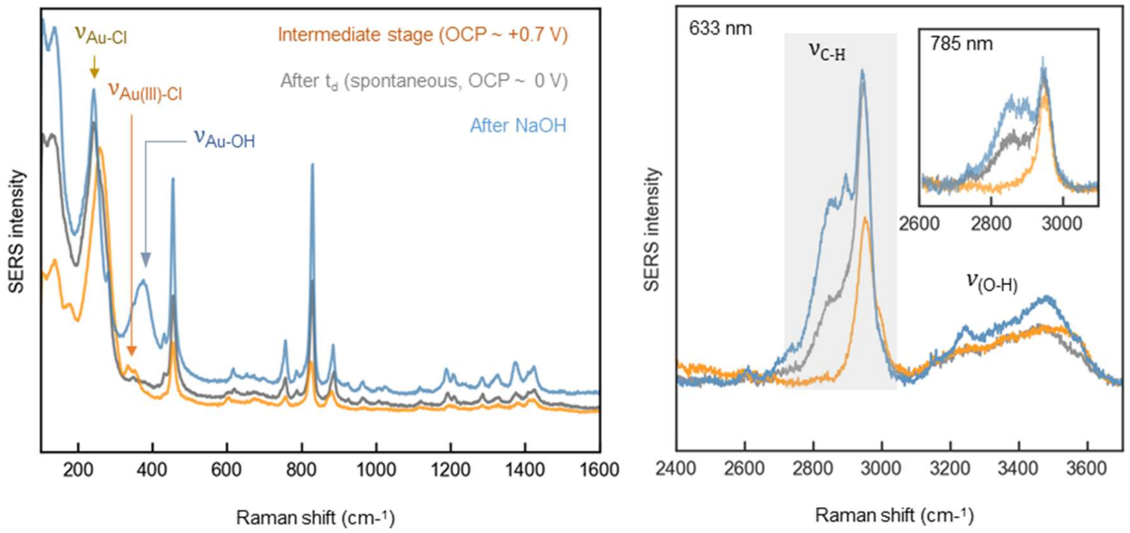

Supplementary Figure 54 | SERS spectra of MLagg-CB[5] during different stages of Ch-ReSERS. SERS spectra of MLagg-CB[5] after the addition of 1 mM CB[5] and 0.5 M HCl during the intermediate state (OCP ~ 0.7 V), after a spontaneous OCP drop, and after induced destabilisation with NaOH treatment. High wavenumber modes were measured with 633 nm and 785 nm laser excitation (inset).

Supplementary Note 16. Ch-ReSERS with citrate

Our Ch-ReSERS studies above provide detailed insight into the redox transitions of surface-bound Au species, highlighting a stepwise transformation from Au(III)-Cl precursors to an intermediate Au-Cl adlayer before reduction to metallic Au(0). Spectro-electrochemical measurements revealed that the Au-Cl adlayer forms a distinct surface dipole that modifies the electronic structure of the Au substrate, as evidenced by an increase in the work function upon its formation. This adlayer not only serves as the key Au(I) intermediate in the reduction sequence but also modulates subsequent ligand adsorption dynamics. Our *in situ* SERS and OCP measurements of Ch-ReSERS enabled direct observation of these dynamic surface-confined processes that are otherwise challenging to resolve with other techniques.

The relevance of Au surface redox chemistry extends beyond Ch-ReSERS, as similar Au(III)-Cl→Au(I)-Cl→Au(0) transformations underlie other important Au-based processes, including the colloidal synthesis of AuNPs. In the classical Turkevich synthesis, citrate functions both as a reducing agent and as a stabilizing ligand, driving the reduction of Au precursors and capping the resulting colloidal AuNPs to control their stability. Notably, work by Rodríguez-González *et al.* demonstrated that the electrochemical potential of a Au electrode immersed in the synthesis mixture remains stable at positive values (+0.7 V) until a rapid drop to 0 V coincides with the completion of particle growth[100]. Since both the colloidal AuNP surface and the Au electrode are immersed in the same chemical environment during synthesis, the same underlying Au redox transformations govern both interfaces, supporting the use of electrode-based measurements as a proxy for colloidal AuNP surface states. The OCP drop observed during AuNP synthesis also parallels the OCP drop observed during Ch-ReSERS experiments. These observations suggest that both systems share similar interfacial redox transitions centred on Au-Cl chemistry.

By substituting CB[5] with citrate in Ch-ReSERS (**Extended Data Figure 10**), we now probe a case where citrate can interact with Au(III)-Cl, Au-Cl, and Au(0) surface species. While our system does not replicate the full complexity of colloidal nanoparticle formation, it enables real-time tracking of surface redox transformations and citrate binding using SERS and electrochemical measurements. Our results show that when an oxidized MLagg is treated with HCl and citrate, the OCP stabilizes at approximately 0.5-0.6 V. During this period, the initial Au oxide SERS signal decreases while a distinct Au-Cl vibrational band emerges at ~260 cm^{-1}. Unlike our previous experiments with CB[5], where a prominent Au(III)-Cl intermediate is detected immediately upon chemical dissolution of Au oxide, no Au(III)-Cl signal is observed during this stage. This suggests that the addition of citrate promotes faster reduction of Au(III) to Au-Cl and Au(0), bypassing the accumulation of detectable Au(III)-Cl species. This observation aligns with prior studies that identified the reduction of Au(I) to Au(0) as the rate-determining step in the redox transformations in AuNP formation[101].

Approximately 10 s later, the OCP drops to ~0 V, similar to the transformation observed in Ch-ReSERS. This transition is accompanied by a shift in the Au-Cl peak from 260 to 240 cm^{-1}, the disappearance of the Au-O band, and a sharp, transient increase in the citrate vibrational band at 1020 cm^{-1}, which subsequently plateaus. We interpret this event as the reductive collapse of the Au-Cl adlayer, which then permits citrate to access and bind to the now-reduced Au surface. The transient spike in citrate signal likely reflects a combination of rapid binding kinetics and a local electromagnetic field enhancement as the surface becomes Au(0). During the OCP drop, we observe a transient appearance of a Au(III)-Cl signal, which we attribute to the disproportionation of remaining surface-bound Au-Cl into Au(0) and Au(III)-Cl. The resulting Au(III) is then rapidly reduced by citrate, completing the redox cascade to a fully metallic, citrate-stabilized surface. This event corresponds to the OCP collapse and the sudden appearance of citrate vibrational bands, indicating rapid surface reorganization. These findings mirror earlier results by Rodriguez-Gonzalez et al., who proposed that the OCP drop during AuNP synthesis reflects the point at which citrate displaces Cl^-, thereby passivating further growth.[100] While that study inferred this transition from electrochemical measurements, our Ch-ReSERS data now provide direct spectroscopic evidence of a similar transformation.

Our results also relate closely to the hypotheses of Polte et al. and Paraniello et al., who proposed that surface layers containing $AuCl_4^-$ mediate redox reactions during nanoparticle growth, though they were unable to identify

the exact nature of these surface-bound intermediates.[102–104] In our system, we observe the formation and eventual depletion of a surface-bound Au-Cl adlayer, a species that may reflect the type of intermediate they hypothesized. Although Ch-ReSERS is not a direct analogue of colloidal synthesis, it offers a view of surface chemistry transitions that are otherwise challenging to access during AuNP growth. These findings reinforce the utility of the MLagg system as a model platform for studying dynamic ligand exchange, redox processes, and surface restructuring relevant for processes such as AuNP synthesis.

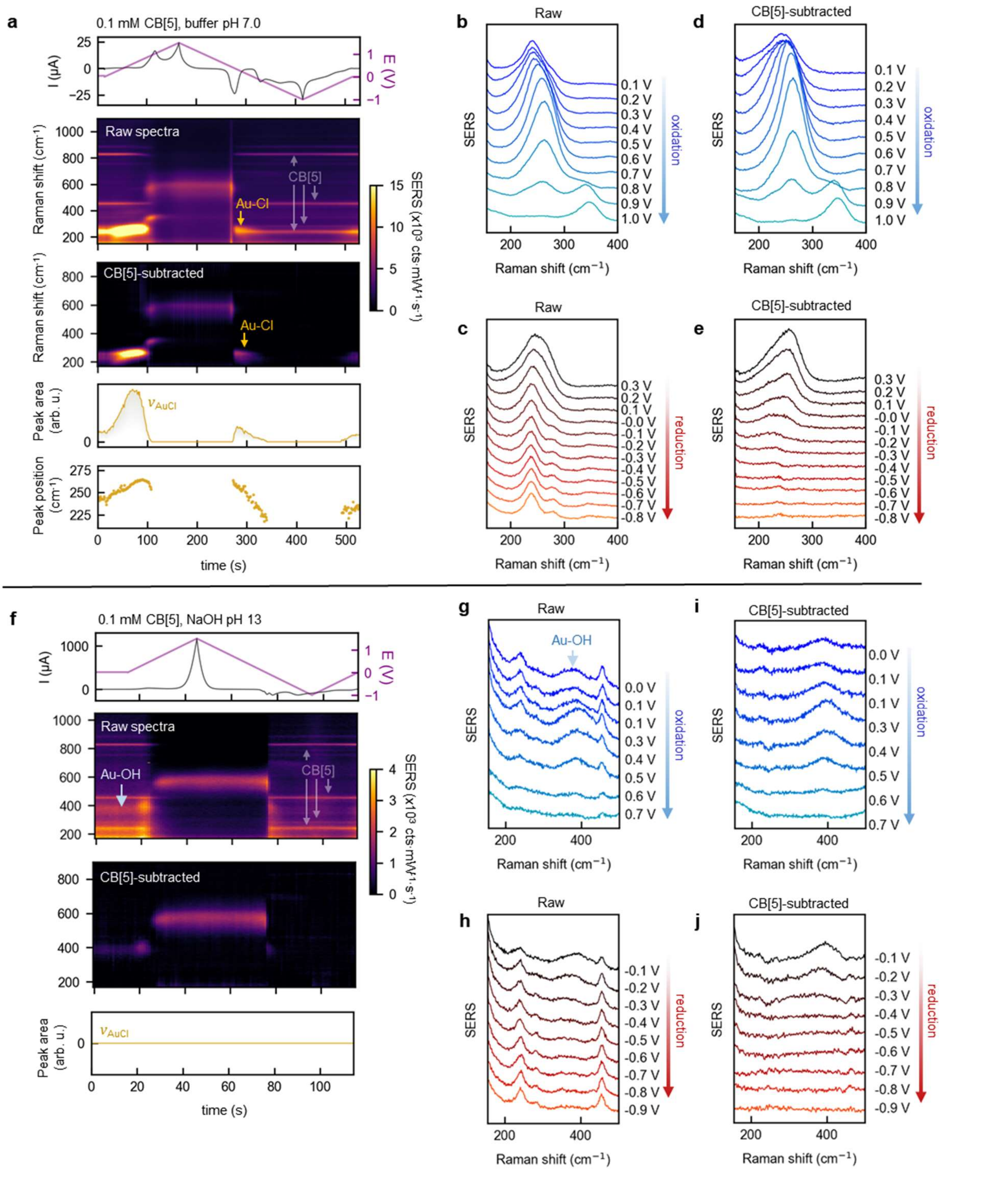

Supplementary Figure 55 | CB[5] SERS spectrum correction for Au-Cl peak extraction. Correction method demonstrated for MLagg-CB[5] undergoing a single oxidation-reduction cycle (ORC) in (**a-e**) 0.1 mM CB[5], 0.5 mM Cl^-, and potassium phosphate buffer (pH 7.0) at 10 mV s^{-1} scan rate. (**a**) Raw time-series SERS spectra and CB[5]-subtracted SERS spectra. The lower panels show the Au-Cl peak area and position extracted from the CB[5]-subtracted SERS spectra. (**b-c**) Raw SERS spectra at low wavenumber region measured at indicated applied potentials during the oxidation and reduction sweeps. (**d-e**) Corresponding CB[5]-subtracted SERS spectra at the same applied potentials. For comparison, the CB[5] subtraction method was applied to an MLagg-CB[5] system undergoing an ORC in (**f-j**) 0.1 mM CB[5], 0.5 mM Cl^- and 100 mM NaOH (pH 13). (**f**) Raw and CB[5]-subtracted time-series SERS spectra are shown with extracted Au-Cl peak area. (**g-h**) Raw SERS spectra at indicated applied potentials during oxidation and reduction sweeps, along with (**i-j**) corresponding CB[5]-subtracted spectra.

Supplementary References

1. Watling, K., Hope, G. A. & Woods, R. SERS Investigation of Gold Dissolution in Chloride and Cyanide Media. *J. Electrochem. Soc.* **152**, D103 (2005).
2. Loo, B. H. In situ identification of halide complexes on gold electrode by surface-enhanced Raman spectroscopy. *J. Phys. Chem.* **86**, 433–437 (1982).
3. Gao, P., Patterson, M. L., Tadayyoni, M. A. & Weaver, M. J. Gold as a ubiquitous substrate for intense surface-enhanced Raman scattering. *Langmuir* **1**, 173–176 (1985).
4. Gao, P. & Weaver, M. J. Metal-adsorbate vibrational frequencies as a probe of surface bonding: Halides and pseudohalides at gold electrodes. *J. Phys. Chem.* **90**, 4057–4063 (1986).
5. Chan, M. Y., Leng, W. & Vikesland, P. J. Surface-Enhanced Raman Spectroscopy Characterization of Salt-Induced Aggregation of Gold Nanoparticles. *ChemPhysChem* **19**, 24–28 (2018).
6. Hargittai, M. & Schulz, A. Molecular Structure, Bonding, and Jahn−Teller Effect in Gold Chlorides: Quantum Chemical Study of AuCl3, Au2Cl6, AuCl4-, AuCl, and Au2Cl2 and Electron Diffraction Study of Au2Cl6. *J Am Chem Soc* **6**, 1449-1458, (2001).
7. Li, Z. L., Wu, T. H., Niu, Z. J., Huang, W. & Nie, H. D. In situ Raman spectroscopic studies on the current oscillations during gold electrodissolution in HCl solution. *Electrochem. Commun.* **6**, 44–48 (2004).
8. Tossell, J. A. The speciation of gold in aqueous solution: A theoretical study. *Geochim. Cosmochim. Acta* **60**, 17–29 (1996).
9. Forneris, B., Hiraishi, J., Miller, F. A. & Uehara, M. Roman and infrared spectra of I2Cl6 and Au2Cl6. *Spectrochim. Acta Part Mol. Spectrosc.* **26**, 581–591 (1970).
10. Husson, E., Quy Dao, N. & Breitinger, D. K. Vibrational spectra and normal coordinate analyses of the gold halides AuX (X = Cl, Br and I). *Spectrochim. Acta Part Mol. Spectrosc.* **37**, 1087–1092 (1981).
11. Braunstein, P. & Clark, R. J. H. The preparation, properties, and vibrational spectra of complexes containing the AuCl2-, AuBr2-, and AuI2- ions. *J. Chem. Soc. Dalton Trans.* 1845–1848 (1973) doi:10.1039/DT9730001845.
12. Pan, P. & Wood, S. A. Gold-chloride complexes in very acidic aqueous solutions and at temperatures 25-300 °C: A laser Raman spectroscopic study. *Geochim. Cosmochim. Acta* **55**, 2365–2371 (1991).
13. Murphy, P. J. & LaGrange, M. S. Raman spectroscopy of gold chloro-hydroxy speciation in fluids at ambient temperature and pressure: A re-evaluation of the effects of pH and chloride concentration. *Geochim. Cosmochim. Acta* **62**, 3515–3526 (1998).
14. Li, C. Y. *et al.* In Situ Monitoring of Electrooxidation Processes at Gold Single Crystal Surfaces Using Shell-Isolated Nanoparticle-Enhanced Raman Spectroscopy. *J. Am. Chem. Soc.* **137**, 7648–7651 (2015).
15. Yang, S. & Hetterscheid, D. G. H. Redefinition of the Active Species and the Mechanism of the Oxygen Evolution Reaction on Gold Oxide. *ACS Catal.* **10**, 12582–12589 (2020).
16. Desilvestro, J. & Weaver, M. J. Surface structural changes during oxidation of gold electrodes in aqueous media as detected using surface-enhanced Raman spectroscopy. *J. Electroanal. Chem. Interfacial Electrochem.* **209**, 377–386 (1986).
17. Pfisterer, J. H. K. *et al.* Role of OH Intermediates during the Au Oxide Electro-Reduction at Low pH Elucidated by Electrochemical Surface-Enhanced Raman Spectroscopy and Implicit Solvent Density Functional Theory. *ACS Catal.* **10**, 12716–12726 (2020).
18. Yeo, B. S., Klaus, S. L., Ross, P. N., Mathies, R. A. & Bell, A. T. Identification of Hydroperoxy Species as Reaction Intermediates in the Electrochemical Evolution of Oxygen on Gold. *ChemPhysChem* **11**, 1854–1857 (2010).
19. Baker, T. A., Xu, B., Liu, X., Kaxiras, E. & Friend, C. M. Nature of oxidation of the Au(111) surface: Experimental and theoretical investigation. *J. Phys. Chem. C* **113**, 16561–16564 (2009).
20. Diaz-Morales, O., Calle-Vallejo, F., de Munck, C. & Koper, M. T. M. Electrochemical water splitting by gold: evidence for an oxide decomposition mechanism. *Chem. Sci.* **4**, 2334 (2013).

21. Zhumaev, U. *et al.* Electro-oxidation of Au(1 1 1) in contact with aqueous electrolytes: New insight from in situ vibration spectroscopy. *Electrochimica Acta* **112**, 853–863 (2013).

22. Pireaux, J. J., Liehr, M., Thiry, P. A., Delrue, J. P. & Caudano, R. Electron spectroscopic characterization of oxygen adsorption on gold surfaces. II. Production of gold oxide in oxygen DC reactive sputtering. *Surf. Sci.* **141**, 221–232 (1984).

23. Baker, T. A., Friend, C. M. & Kaxiras, E. Atomic oxygen adsorption on Au(111) surfaces with defectsc. *J. Phys. Chem. C* **113**, 3232–3238 (2009).

24. Griffiths, M. J. & Barrow, R. F. Observations on the electronic spectra of CuO, AgO and AuO isolated in rare gas matrices. *J. Chem. Soc. Faraday Trans. 2 Mol. Chem. Phys.* **73**, 943–951 (1977).

25. Wang, X. & Andrews, L. Infrared spectrum and structure of the gold dihydroxide molecule. *Chem. Commun.* **2**, 4001–4003 (2005).

26. Takechi-Haraya, Y. *et al.* Current Status and Challenges of Analytical Methods for Evaluation of Size and Surface Modification of Nanoparticle-Based Drug Formulations. *AAPS PharmSciTech* **23**, 150 (2022).

27. Sibug-Torres, S. M. *et al.* In situ electrochemical regeneration of nanogap hotspots for continuously reusable ultrathin SERS sensors. *Nat. Commun.* **15**, 2022 (2024).

28. Grys, D. *et al.* Eliminating irreproducibility in SERS substrates. *J. Raman Spectrosc.* **52**, 412–419 (2021).

29. Taylor, R. W. *et al.* Precise Subnanometer Plasmonic Junctions for SERS within Gold Nanoparticle Assemblies Using Cucurbit[*n*]uril "Glue". *ACS Nano* **5**, 3878–3887 (2011).

30. Arul, R. *et al.* Giant mid-IR resonant coupling to molecular vibrations in sub-nm gaps of plasmonic multilayer metafilms. *Light Sci. Appl.* **11**, 281 (2022).

31. Klyushin, A. Y., Rocha, T. C. R., Hävecker, M., Knop-Gericke, A. & Schlögl, R. A near ambient pressure XPS study of Au oxidation. *Phys. Chem. Chem. Phys.* **16**, 7881–7886 (2014).

32. Higo, M. *et al.* Formation and decomposition of gold oxides prepared by an oxygen-dc glow discharge from gold films and studied by X-ray photoelectron spectroscopy. *Thin Solid Films* **699**, 137870 (2020).

33. Juodkazis, K., Juodkazyte, J., Jasulaitiene, V., Lukinskas, A. & Šebeka, B. XPS studies on the gold oxide surface layer formation. *Electrochem. Commun.* **2**, 503–507 (2000).

34. Higo, M. *et al.* Reaction monitoring of gold oxides prepared by an oxygen-dc glow discharge from gold films in various aqueous solutions by a surface plasmon resonance-based optical waveguide sensing system and X-ray photoelectron spectroscopy. *Anal Sci* **36**, 1081-1089, (2020).

35. Grys, D.-B. *et al.* Controlling Atomic-Scale Restructuring and Cleaning of Gold Nanogap Multilayers for Surface-Enhanced Raman Scattering Sensing. *ACS Sens.* **8**, 2879–2888 (2023).

36. Fadley, C. S., Baird, R. J., Siekhaus, W., Novakov, T. & Berstrom, S. A. L. Surface analysis and angular distributions in x-ray photoelectron spectroscopy. *J. Electrom Spectrosc. Relat. Phenom.* **4**, 93–137 (1974).

37. Cimino, A., Gazzoli, D. & Valigi, M. XPS quantitative analysis and models of supported oxide catalysts. *J. Electron Spectrosc. Relat. Phenom.* **104**, 1–29 (1999).

38. Jablonski, A. Evaluation of procedures for overlayer thickness determination from XPS intensities. *Surf. Sci.* **688**, 14–24 (2019).

39. Powell, C. J. & Jablonski, A. *NIST Electron Effective-Attenuation-Length Database - Version 1.3*. (National Institute of Standards and Technology, Gaithersburg, MD, 2011).

40. Kim, Y., Collins, R. W. & Vedam, K. Fast scanning spectroelectrochemical ellipsometry: In-situ characterization of gold oxide. *Surf. Sci.* **233**, 341–350 (1990).

41. Wu, C., Yin, M., Brien, S. O. & Koberstein, J. T. Quantitative Analysis of Copper Oxide Nanoparticle Composition and Structure by X-ray Photoelectron Spectroscopy. *Chem. Mater.* **18**, 6054–6058 (2006).

42. Ono, L. K. & Roldan Cuenya, B. Formation and Thermal Stability of Au 2 O 3 on Gold Nanoparticles: Size and Support Effects. *J. Phys. Chem. C* **112**, 4676–4686 (2008).

43. Tremiliosi-Filho, G., Dall'Antonia, L. H. & Jerkiewicz, G. Growth of surface oxides on gold electrodes under well-defined potential, time and temperature conditions. *J. Electroanal. Chem.* **578**, 1–8 (2005).

44. Cherevko, S., Topalov, A. A., Zeradjanin, A. R., Katsounaros, I. & Mayrhofer, K. J. J. Gold dissolution: Towards understanding of noble metal corrosion. *RSC Adv.* **3**, 16516–16527 (2013).

45. Rouya, E., Cattarin, S., Reed, M. L., Kelly, R. G. & Zangari, G. Electrochemical Characterization of the Surface Area of Nanoporous Gold Films. *J. Electrochem. Soc.* **159**, K97–K102 (2012).

46. Tremiliosi-Filho, G., Dall'Antonia, L. H. & Jerkiewicz, G. Growth of surface oxides on gold electrodes under well-defined potential, time and temperature conditions. *J. Electroanal. Chem.* **578**, 1–8 (2005).

47. Diaz-Morales, O., Calle-Vallejo, F., De Munck, C. & Koper, M. T. M. Electrochemical water splitting by gold: evidence for an oxide decomposition mechanism. *Chem. Sci.* **4**, 2334 (2013).

48. Dahlin, A. B., Zahn, R. & Vörös, J. Nanoplasmonic sensing of metal–halide complex formation and the electric double layer capacitor. *Nanoscale* **4**, 2339 (2012).

49. Sannomiya, T., Dermutz, H., Hafner, C., Vörös, J. & Dahlin, A. B. Electrochemistry on a Localized Surface Plasmon Resonance Sensor. *Langmuir* **26**, 7619–7626 (2010).

50. Mulvaney, P. Surface Plasmon Spectroscopy of Nanosized Metal Particles. *Langmuir* 788–800 (1996) doi:10.1021/la9502711.

51. Angerstein-Kozlowska, H., Conway, B. E., Hamelin, A. & Stoicoviciu, L. Elementary steps of electrochemical oxidation of single-crystal planes of Au. Part II. A chemical and structural basis of oxidation of the (111) plane. *J. Electroanal. Chem.* **228**, 429–453 (1987).

52. Kasian, O. *et al.* Electrochemical dissolution of gold in presence of chloride and bromide traces studied by on-line electrochemical inductively coupled plasma mass spectrometry. *Electrochimica Acta* **222**, 1056–1063 (2016).

53. Chen, H. C. *et al.* In situ creation of surface-enhanced raman scattering active au-auo x nanostructures through electrochemical process for pigment detection. *ACS Omega* **3**, 16576–16584 (2018).

54. Burke, L. D. & Nugent, P. F. The electrochemistry of gold: I. The redox behaviour of the metal in aqueous media. *Gold Bull.* **30**, 43–53 (1997).

55. Weiher, N. Combined in situ and ex situ Studies of an Electrochemical Interface: Investigation of Anodic Oxide Layers on Gold. (2003).

56. Gao, W. *et al.* Chlorine Adsorption on Au(111): Chlorine Overlayer or Surface Chloride? *J. Am. Chem. Soc.* **130**, 3560–3565 (2008).

57. Andryushechkin, B. V. *et al.* Self-Organization of Gold Chloride Molecules on Au(111) Surface. *J. Phys. Chem. C* **117**, 24948–24954 (2013).

58. Rzeźnicka, I. I. *et al.* Chlorine adlayer-templated growth of a hybrid inorganic–organic layered structure on Au(111). *Surf. Sci.* **652**, 46–50 (2016).

59. Baker, T. A., Friend, C. M. & Kaxiras, E. Nature of Cl bonding on the Au(111) surface: Evidence of a mainly covalent interaction. *J. Am. Chem. Soc.* **130**, 3720–3721 (2008).

60. Lin, R.-L. *et al.* Selective Recovery and Detection of Gold with Cucurbit[n]urils (n = 5–7). *Inorg. Chem.* **59**, 3850–3855 (2020).

61. Wu, H. *et al.* High-Efficiency Gold Recovery Using Cucurbit[6]uril. *ACS Appl. Mater. Interfaces* **12**, 38768–38777 (2020).

62. Mayet, N., Servat, K., Kokoh, K. B. & Napporn, T. W. Probing the Surface of Noble Metals Electrochemically by Underpotential Deposition of Transition Metals. *Surfaces* **2**, 257–276 (2019).

63. Hamelin, A. Underpotential deposition of lead on single crystal faces of gold. *J. Electroanal. Chem. Interfacial Electrochem.* **165**, 167–180 (1984).

64. Jeyabharathi, C., Zander, M. & Scholz, F. Underpotential deposition of lead on quasi-spherical and faceted gold nanoparticles. *J. Electroanal. Chem.* **819**, 159–162 (2018).

65. Baumberg, J. J. Picocavities: a Primer. *Nano Lett.* **22**, 5859–5865 (2022).

66. Tian, Z.-Q., Ren, B. & Wu, D.-Y. Surface-Enhanced Raman Scattering: From Noble to Transition Metals and from Rough Surfaces to Ordered Nanostructures. *J. Phys. Chem. B* **106**, 9463–9483 (2002).

67. Chen, A. & Lipkowski, J. Electrochemical and Spectroscopic Studies of Hydroxide Adsorption at the Au(111) Electrode. *J. Phys. Chem. B* **103**, 682–691 (1999).

68. Burke, L. D. & Nugent, P. F. The electrochemistry of gold: II The electrocatalytic behaviour of the metal in aqueous media. *Gold Bull.* **31**, 39–50 (1998).

69. Yang, S. & Hetterscheid, D. G. H. Redefinition of the Active Species and the Mechanism of the Oxygen Evolution Reaction on Gold Oxide. *ACS Catal.* **10**, 12582–12589 (2020).

70. Baker, T. A., Friend, C. M. & Kaxiras, E. Nature of Cl bonding on the Au(111) surface: Evidence of a mainly covalent interaction. *J Am Chem Soc* **130**, 3720-3721, (2008).

71. Magnussen, O. M. Ordered Anion Adlayers on Metal Electrode Surfaces. *Chem. Rev.* **102**, 679–726 (2002).

72. Shi, Z. & Lipkowski, J. Chloride adsorption at the Au(111) electrode surface. *J. Electroanal. Chem.* **403**, 225–239 (1996).

73. Lipkowski, J., Shi, Z., Chen, A., Pettinger, B. & Bilger, C. Ionic adsorption at the Au(111) electrode. *Electrochimica Acta* **43**, 2875–2888 (1998).

74. Gao, P. & Weaver, M. J. Metal-adsorbate vibrational frequencies as a probe of surface bonding: halides and pseudohalides at gold electrodes. *J. Phys. Chem.* **90**, 4057–4063 (1986).

75. Ávila, M., Juárez, M. F. & Santos, E. Role of the Partial Charge Transfer on the Chloride Adlayers on Au(100). *ChemElectroChem* **7**, 4269–4282 (2020).

76. Wang, G. *et al.* Effect of molecular desorption on the electronic properties of self-assembled polarizable molecular monolayers. *J. Colloid Interface Sci.* **419**, 39–45 (2014).

77. Boettcher, S. W. *et al.* Potentially Confusing: Potentials in Electrochemistry. *ACS Energy Lett.* **6**, 261–266 (2021).

78. Spencer, N. D. & Lambert, R. M. Chlorine chemisorption and surface chloride formation on Au(111). *Surf. Sci.* **107**, 237–248 (1981).

79. Chang, X. *et al.* Understanding the complementarities of surface-enhanced infrared and Raman spectroscopies in CO adsorption and electrochemical reduction. *Nat. Commun.* **13**, (2022).

80. Turetta, N., Sedona, F., Liscio, A., Sambi, M. & Samorì, P. Au(111) Surface Contamination in Ambient Conditions: Unravelling the Dynamics of the Work Function in Air. *Adv. Mater. Interfaces* **8**, 2100068 (2021).

81. Wright, D. *et al.* Vibrational Stark Effects: Ionic Influence on Local Fields. *J. Phys. Chem. Lett.* **13**, 4905–4911 (2022).

82. Wasileski, S. A., Koper, M. T. M. & Weaver, M. J. Field-Dependent Electrode−Chemisorbate Bonding: Sensitivity of Vibrational Stark Effect and Binding Energetics to Nature of Surface Coordination. *J. Am. Chem. Soc.* **124**, 2796–2805 (2002).

83. Ali, A. H. & Foss, C. A. Electrochemically Induced Shifts in the Plasmon Resonance Bands of Nanoscopic Gold Particles Adsorbed on Transparent Electrodes. *J. Electrochem. Soc.* **146**, 628–636 (1999).

84. Sannomiya, T., Dermutz, H., Hafner, C., Vörös, J. & Dahlin, A. B. Electrochemistry on a Localized Surface Plasmon Resonance Sensor. *Langmuir* **26**, 7619–7626 (2010).

85. Dahlin, A. B., Zahn, R. & Vörös, J. Nanoplasmonic sensing of metal–halide complex formation and the electric double layer capacitor. *Nanoscale* **4**, 2339 (2012).

86. Rzeźnicka, I. I. *et al.* Chlorine adlayer-templated growth of a hybrid inorganic–organic layered structure on Au(111). *Surf. Sci.* **652**, 46–50 (2016).

87. Wu, H. *et al.* High-Efficiency Gold Recovery Using Cucurbit[6]uril. *ACS Appl. Mater. Interfaces* **12**, 38768–38777 (2020).

88. Lin, R.-L. *et al.* Selective Recovery and Detection of Gold with Cucurbit[*n*]urils (*n* = 5–7). *Inorg. Chem.* **59**, 3850–3855 (2020).

89. Koper, M. T. M. & Van Santen, R. A. Interaction of H, O and OH with metal surfaces. *J. Electroanal. Chem.* **472**, 126–136 (1999).

90. Andryushechkin, B. V. *et al.* Self-Organization of Gold Chloride Molecules on Au(111) Surface. *J. Phys. Chem. C* **117**, 24948–24954 (2013).

91. Gao, W. *et al.* Chlorine Adsorption on Au(111): Chlorine Overlayer or Surface Chloride? *J. Am. Chem. Soc.* **130**, 3560–3565 (2008).

92. Wang, D.-W., Wu, K.-H., Gentle, I. R. & (Max) Lu, G. Q. Anodic chlorine/nitrogen co-doping of reduced graphene oxide films at room temperature. *Carbon* **50**, 3333–3341 (2012).

93. Janssen, E. M. W., Folmer, J. C. W. & Wiegers, G. A. The preparation and crystal structure of gold monochloride, AuCl. *J. Common Met.* **38**, 71–76 (1974).

94. Baker, T. A., Friend, C. M. & Kaxiras, E. Effects of chlorine and oxygen coverage on the structure of the Au(111) surface. *J. Chem. Phys.* **130**, 084701 (2009).

95. Juodkazis, K., Juodkazytė, J., Juodienė, T. & Lukinskas, A. Determination of Au(III) in the surface layers formed anodically on the gold electrode. *J. Electroanal. Chem.* **441**, 19–24 (1998).

96. Higo, M. *et al.* Reaction monitoring of gold oxides prepared by an oxygen-dc glow discharge from gold films in various aqueous solutions by a surface plasmon resonance-based optical waveguide sensing system and X-ray photoelectron spectroscopy. *Anal. Sci.* **36**, 1081–1089 (2020).

97. Higo, M. *et al.* Formation and decomposition of gold oxides prepared by an oxygen-dc glow discharge from gold films and studied by X-ray photoelectron spectroscopy. *Thin Solid Films* **699**, 137870 (2020).

98. Angerstein-Kozlowska, H., Conway, B. E., Tellefsen, K. & Barnett, B. Stochastically-gated surface processes involving anions in oxidation of Au: time-resolution of processes down to 0.25% coverages and 50 μs time-scales. *Electrochimica Acta* **34**, 1045–1056 (1989).

99. Pfisterer, J. H. K. *et al.* Role of OH Intermediates during the Au Oxide Electro-Reduction at Low pH Elucidated by Electrochemical Surface-Enhanced Raman Spectroscopy and Implicit Solvent Density Functional Theory. *ACS Catal.* **10**, 12716–12726 (2020).

100. Rodríguez-González, B., Mulvaney, P. & Liz-Marzán, L. M. An Electrochemical Model for Gold Colloid Formation via Citrate Reduction. *Z. Für Phys. Chem.* **221**, 415–426 (2007).

101. Gao, Y. & Torrente-Murciano, L. Mechanistic insights of the reduction of gold salts in the Turkevich protocol. *Nanoscale* **12**, 2740–2751 (2020).

102. Polte, J. *et al.* Mechanism of Gold Nanoparticle Formation in the Classical Citrate Synthesis Method Derived from Coupled In Situ XANES and SAXS Evaluation. *J. Am. Chem. Soc.* **132**, 1296–1301 (2010).

103. Wuithschick, M. *et al.* Turkevich in New Robes: Key Questions Answered for the Most Common Gold Nanoparticle Synthesis. *ACS Nano* **9**, 7052–7071 (2015).

104. Mulvaney, P. Surface Plasmon Spectroscopy of Nanosized Metal Particles. *Langmuir* **12**, 788–800 (1996).
